# Revisiting the Middle and Upper Palaeolithic archaeology of Gruta do Caldeirão (Tomar, Portugal)

**DOI:** 10.1371/journal.pone.0259089

**Published:** 2021-10-27

**Authors:** João Zilhão, Diego E. Angelucci, Lee J. Arnold, Francesco d’Errico, Laure Dayet, Martina Demuro, Marianne Deschamps, Helen Fewlass, Luís Gomes, Beth Linscott, Henrique Matias, Alistair W. G. Pike, Peter Steier, Sahra Talamo, Eva M. Wild

**Affiliations:** 1 Institució Catalana de Recerca i Estudis Avançats (ICREA), Barcelona, Spain; 2 Departament d’Història i Arqueologia, Facultat de Geografia i Història, Universitat de Barcelona, Barcelona, Spain; 3 UNIARQ–Centro de Arqueologia da Universidade de Lisboa, Faculdade de Letras de Lisboa, Universidade de Lisboa, Lisboa, Portugal; 4 Dipartimento di Lettere e Filosofia, Università degli Studi di Trento, Trento, Italy; 5 Department of Earth Sciences, Environment Institute, Institute for Photonics and Advanced Sensing (IPAS), School of Physical Sciences, University of Adelaide, Adelaide, South Australia, Australia; 6 UMR 5199 PACEA, Université de Bordeaux, Bordeaux, France; 7 UMR 5608 TRACES, Université de Toulouse, Toulouse, France; 8 Department of Human Evolution, Max Planck Institute for Evolutionary Anthropology, Leipzig, Germany; 9 Oxford Radiocarbon Accelerator Unit, University of Oxford, Oxford, United Kingdom; 10 Department of Archaeology, University of Southampton, Southampton, United Kingdom; 11 Isotope Physics, Universität Wien, Vienna, Austria; 12 Department of Chemistry "Giacomo Ciamician", Alma Mater Studiorum, University of Bologna, Bologna, Italy; University at Buffalo - The State University of New York, UNITED STATES

## Abstract

Gruta do Caldeirão features a *c*. 6 m-thick archaeological stratification capped by Holocene layers ABC-D and Ea, which overlie layer Eb, a deposit of Magdalenian age that underwent significant disturbance, intrusion, and component mixing caused by funerary use of the cave during the Early Neolithic. Here, we provide an updated overview of the stratigraphy and archaeological content of the underlying Pleistocene succession, whose chronology we refine using radiocarbon and single-grain optically stimulated luminescence dating. We find a high degree of stratigraphic integrity. Dating anomalies exist in association with the succession’s two major discontinuities: between layer Eb and Upper Solutrean layer Fa, and between Early Upper Palaeolithic layer K and Middle Palaeolithic layer L. Mostly, the anomalies consist of older-than-expected radiocarbon ages and can be explained by bioturbation and palimpsest-forming sedimentation hiatuses. Combined with palaeoenvironmental inferences derived from magnetic susceptibility analyses, the dating shows that sedimentation rates varied in tandem with the oscillations in global climate revealed by the Greenland oxygen isotope record. A steep increase in sedimentation rate is observed through the Last Glacial Maximum, resulting in a *c*. 1.5 m-thick accumulation containing conspicuous remains of occupation by people of the Solutrean technocomplex, whose traditional subdivision is corroborated: the index fossils appear in the expected stratigraphic order; the diagnostics of the Protosolutrean and the Lower Solutrean predate 24,000 years ago; and the constraints on the Upper Solutrean place it after Greenland Interstadial 2.2. (23,220–23,340 years ago). Human usage of the site during the Early Upper and the Middle Palaeolithic is episodic and low-intensity: stone tools are few, and the faunal remains relate to carnivore activity. The Middle Palaeolithic is found to persist beyond 39,000 years ago, at least three millennia longer than in the Franco-Cantabrian region. This conclusion is upheld by Bayesian modelling and stands even if the radiocarbon ages for the Middle Palaeolithic levels are removed from consideration (on account of observed inversions and the method’s potential for underestimation when used close to its limit of applicability). A number of localities in Spain and Portugal reveal a similar persistence pattern. The key evidence comes from high-resolution fluviatile contexts spared by the site formation issues that our study of Caldeirão brings to light—palimpsest formation, post-depositional disturbance, and erosion. These processes. are ubiquitous in the cave and rock-shelter sites of Iberia, reflecting the impact on karst archives of the variation in climate and environments that occurred through the Upper Pleistocene, and especially at two key points in time: between 37,000 and 42,000 years ago, and after the Last Glacial Maximum. Such empirical difficulties go a long way towards explaining the controversies surrounding the associated cultural transitions: from the Middle to the Upper Palaeolithic, and from the Solutrean to the Magdalenian. Alongside potential dating error caused by incomplete decontamination, proper consideration of sample association issues is required if we are ever to fully understand what happened with the human settlement of Iberia during these critical intervals, and especially so with regards to the fate of Iberia’s last Neandertal populations.

## 1. Introduction

### 1.1. Research design

**Fig 1:**
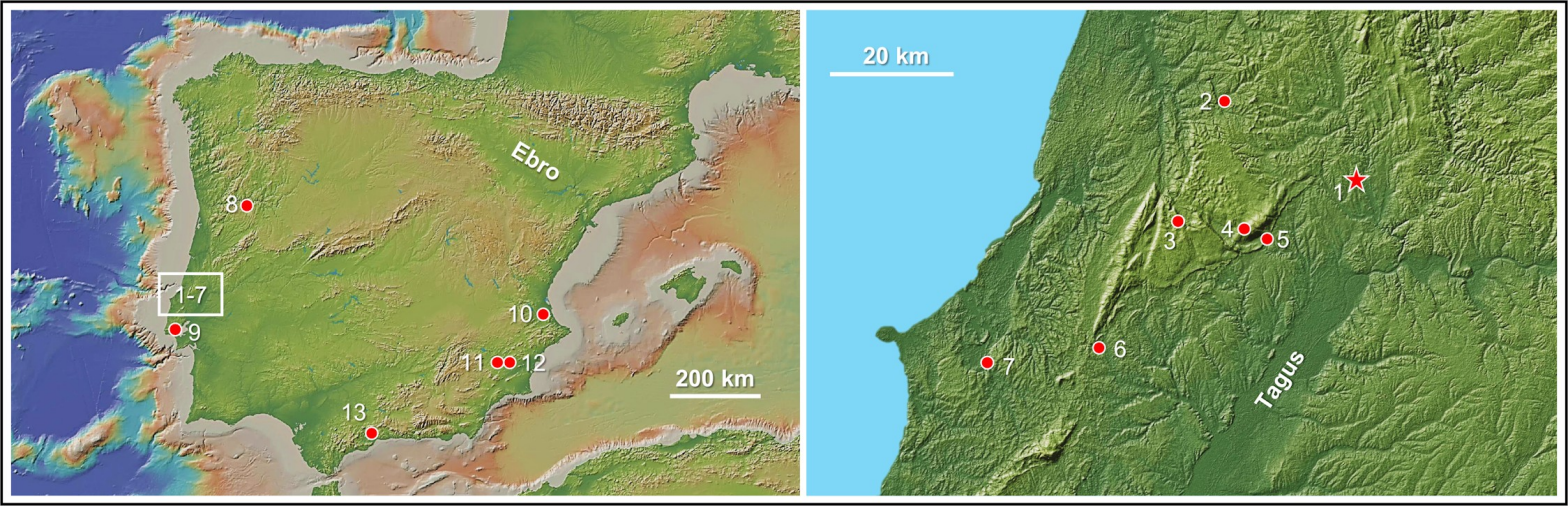
Location of the sites mentioned in the text. 1. Gruta do Caldeirão. 2. Abrigo do Lagar Velho; 3. Lapa do Anecrial; 4. Lapa do Picareiro; 5. Gruta da Oliveira; 6. Gato Preto; 7. Gruta Nova da Columbeira; 8. Cardina/Salto do Boi; 9. Pego do Diabo; 10. Cova de Malladetes; 11. Cueva Antón; 12. Fica Doña Martina and Abrigo de La Boja (Rambla Perea). 13. Cueva Bajondillo. Relief map: Global Multi-Resolution Topography Synthesis (https://www.gmrt.org/GMRTMapTool/). Reproduced from [5] under a CC BY 4.0 license.

**Fig 2:**
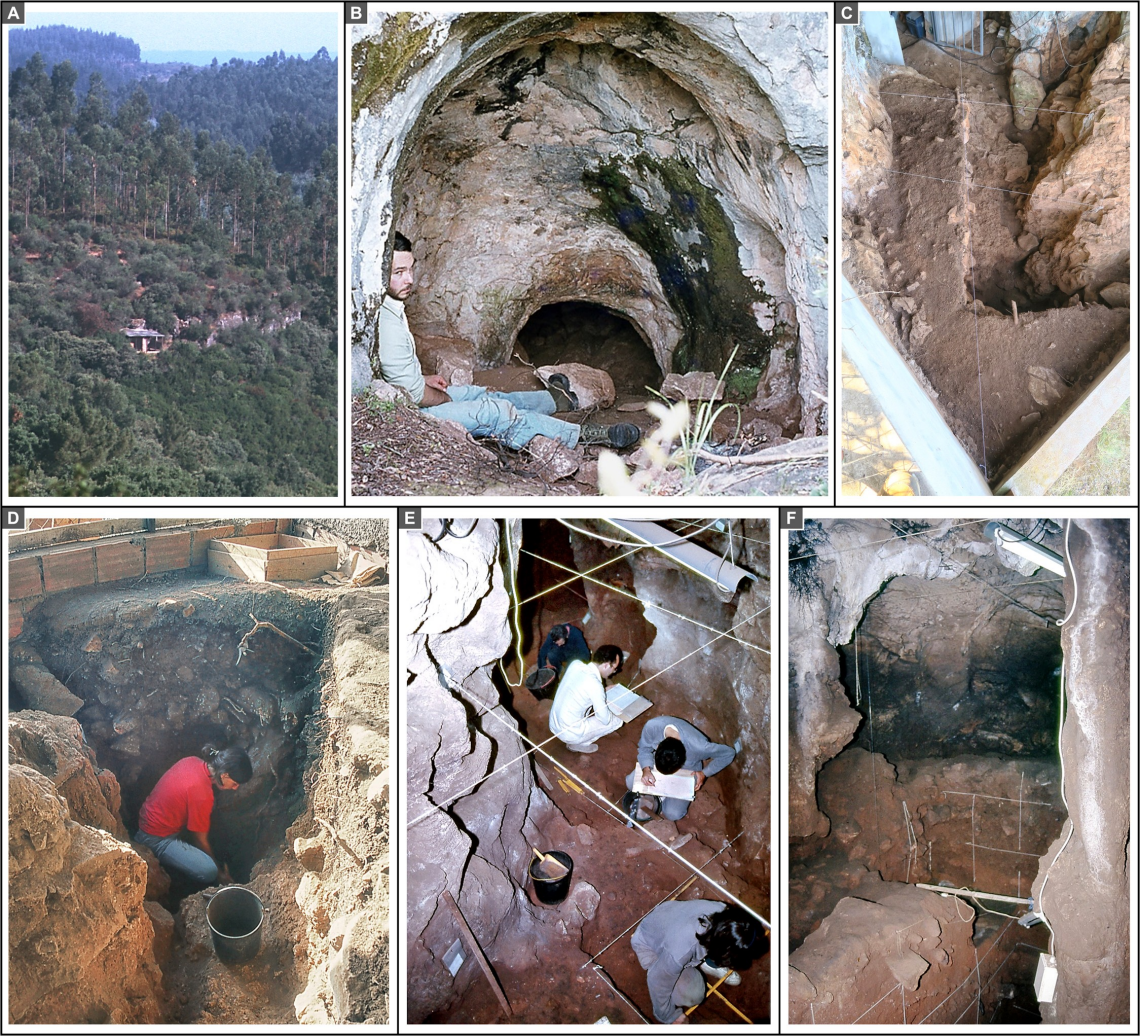
The excavation. **A.** Overview of the small limestone escarpment where Gruta do Caldeirão is located, taken with a zoom lens from the town of Pedreira, 765 m to SSE (1986). **B.** The cave entrance at the time of discovery (1980). **C.** The Entrance trench at the end of the 1979–88 fieldwork. **D.** The Entrance trench during excavation (1987). **E.** Excavation of the Upper Solutrean deposit in the Corridor area (1985). **F.** The Back Chamber towards the end of the excavation (1987); the staining of the wall at the bottom end of the chamber denotes the elevation reached by layer ABC-D, the thick black earth deposit of recent Holocene age that originally capped the stratigraphic succession.

Gruta do Caldeirão (39°38’49.11" N, 8°24’58.32" W) is a karst cavity located in the municipality of Tomar, 132 m asl (above modern sea level). The bedrock is Jurassic limestone, and the cave opens on a low, south-facing cliff located 400 m upstream of a short side valley of the Nabão river, which is a sub-tributary of the Tagus (Figs 1 and 2). Between 1979 and 1988, the site was archaeologically explored under the umbrella of a research project whose principal aim was to provide a chronostratigraphic framework for the Upper Palaeolithic of Portugal [1, 2]. The approach to the interpretation of finds and stratigraphy used through the excavation and initial publication of results hailed from the emerging application of the taphonomic perspective to the study of karst archives (e.g., [3]) and was supported by AMS (Accelerator Mass Spectrometry) dating of bone samples, then in its early stages of development (e.g., [4]). This critical approach to issues of site formation and assemblage integrity shed new light on a number of controversial issues.

Caldeirão yielded one the few funerary contexts of the Cardial culture (west Mediterranean Europe’s first farmers), documenting its long-term contemporaneity with the last hunter-gatherers of the Tagus valley and prompting the formulation of the “maritime pioneer colonisation” model for the Mesolithic-Neolithic transition in southern France and Iberia [6–8]. Its thick Solutrean deposit (layers Fa-Ja) yielded diagnostic stone tool types (unifacial points, laurel-leaves, shouldered or tanged points) in the anticipated stratigraphic order, contradicting functional explanations of their presence/absence [9] and supporting the traditional subdivision of the technocomplex into Proto, Lower, Middle and Upper phases [10]. Underlying the Solutrean, a sequence of Early Upper and Middle Palaeolithic horizons was identified (layers Jb-P), among which layer K, dated from a red deer phalanx to 27,700 ± 600 BP (OxA-1941), was initially thought to represent the most recent Mousterian. This result suggested contemporaneity with other Middle Palaeolithic cave site occupations dated to the same time range, namely at Gruta Nova da Columbeira (Bombarral) (Fig 1, no. 7) [11], and was consistent with the earliest reliable radiocarbon age then available for the Upper Palaeolithic of Portugal: 28,120 +860/-780 BP (ICEN-732), for Aurignacian level 2 of the small cave of Pego do Diabo (Loures) (Fig 1, no. 9). This chronological evidence indicated that the Portuguese Upper Palaeolithic had begun significantly later than elsewhere in Europe, leading to the inclusion of Caldeirão among the list of sites providing support for the “Ebro Frontier” model of the Middle-to-Upper Palaeolithic transition in Iberia (henceforth, the “Transition”) [12–19].

At the time of initial formulation [12], Ebro Frontier stemmed from the observation that, after more than a century of research, occurrences of the Protoaurignacian and the Early Aurignacian (a.k.a. Aurignacian I) remained unknown in Portugal as well as in central, eastern, and southern Spain. Such an absence was all the more remarkable because it concerned the Early Aurignacian not just as a defined assemblage structure or differentiated unit within a stratified sequence of occupations but also as isolated or out-of-context finds (e.g., index fossils, such as the split-based bone point, retrieved in mixed or reworked deposits). The model posited that the corresponding chronostratigraphic slot was occupied by a late-persisting Middle Palaeolithic and that, given known associations between human types and technocomplexes, such latest Middle Palaeolithic Iberians were Neandertal people. At the time, these interpretations questioned prevailing ideas that, because of a putative, biologically based inferiority in cognition or adaptation, the demise of Neandertals would have swiftly followed the first appearance of Upper Palaeolithic modern humans at their doorstep [20, 21].

Subsequent research has questioned some of the premises underpinning the association of Caldeirão with the Ebro Frontier pattern. Firstly, contra initial assessments, revision of the lithic assemblage from the open-air site of Gato Preto (Rio Maior; Fig 1, no. 6) concluded that it represented a genuine manifestation of the Evolved Aurignacian (a.k.a. Aurignacian II) [22]. This revised assessment implied an age in excess of 36,000 years ago for the Gato Preto assemblage, consistent with its TL (thermoluminescence) dating to 38,100 ± 3900 years ago, and, therefore, a start date for the Upper Palaeolithic of Portugal significantly earlier than the time horizon suggested by the single radiocarbon age then available for layer K of Caldeirão. Secondly, the excavation of the Lagar Velho rock-shelter (Fig 1, no.2) showed that, due to site function or raw material availability factors, lithic assemblages akin to Caldeirão layer K’s could occur within well-dated Gravettian sequences [23, 24]. Thirdly, the Columbeira ages were shown to be vastly underestimated [25].

These developments called for a revision of the evidence from Caldeirão, one that otherwise might profit from the significant technical and methodological progress made by archaeological science since the 1980s. Over the last decade, we have therefore undertaken a thorough revision of the pre-Magdalenian levels of the deposit:

- Based on the extant profiles, the succession, for which grain-size and geochemical analyses had been produced in the 1980s and 1990s, was the object of detailed stratigraphic and geoarchaeological description.
- The taxonomy and the paleoclimate significance of the micromammal assemblages were reassessed [26].
- Building on the results of a previous study intent on characterizing the technology of quartzite reduction in the uppermost (Magdalenian and Solutrean) levels of the sequence [27], stone tool refitting work was carried out on the finds from the Early Upper and Middle Palaeolithic levels.
- The Early Upper Palaeolithic objects of personal ornamentation made on marine shell were directly dated and analysed for their technology of production and the composition of associated pigment residues.
- Additional samples of animal bone and tooth from the 1979–88 field seasons were dated by radiocarbon and, in 2017, samples from the basal levels exposed in the extant profiles were taken for OSL (optically stimulated luminescence) dating.
- The finds made in the trench opened in the extant cave porch, which remained unpublished, were thoroughly examined, and its sequence was correlated with that of the interior excavation.

**Fig 3:**
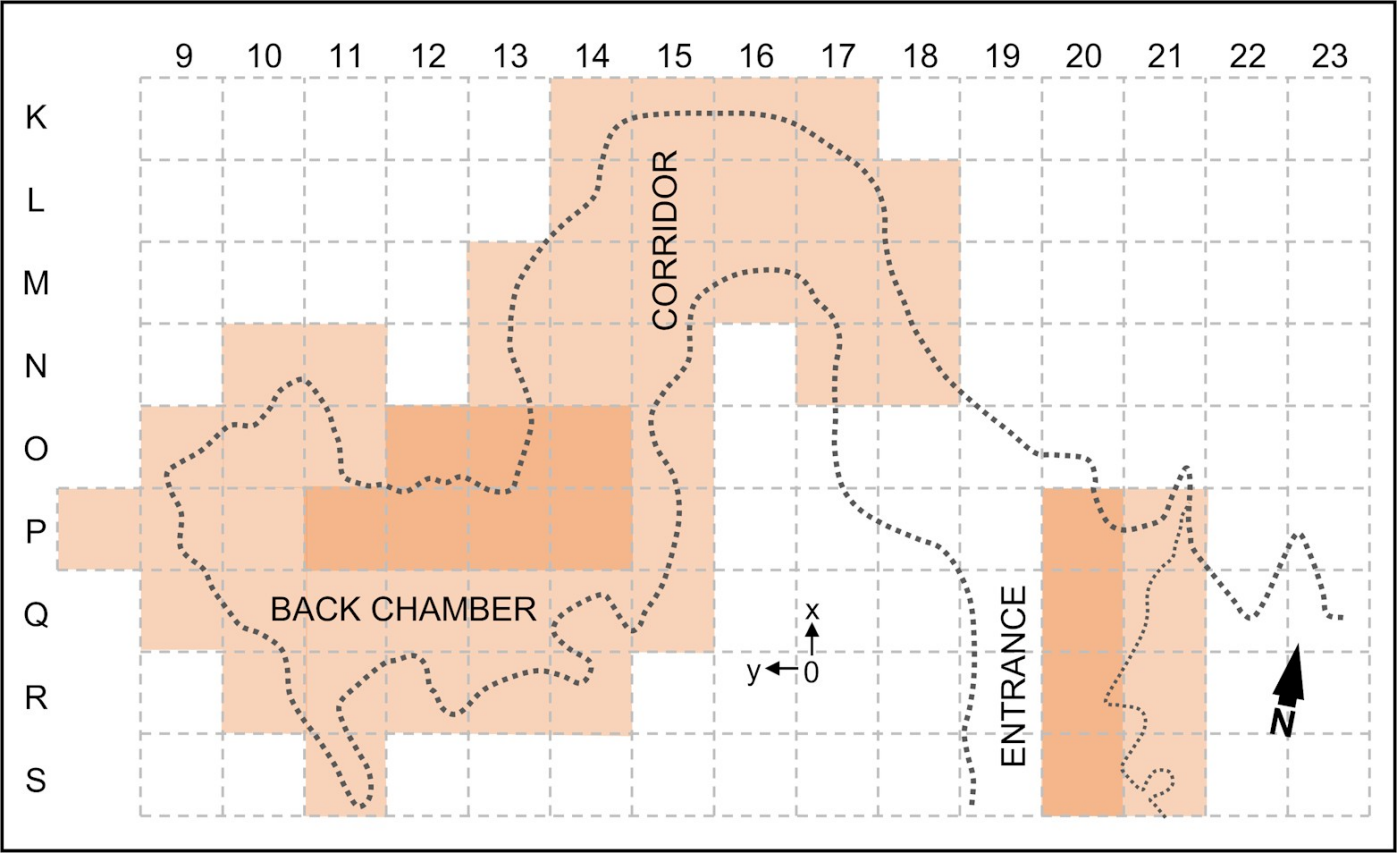
Plan and grid. Plan of the cave at the elevation of the sedimentary infill prior to excavation, and one-square-meter grid used during the 1979–88 field seasons. The excavated grid units are highlighted; the darker shading indicates those where the Early Upper and Middle Palaeolithic levels were reached.

In this paper we present the key findings of this re-evaluation, bringing together the evidence from the three areas of the cave (Entrance, Corridor, and Back Chamber; Figs 2 and 3). We focus on the results of our dating program and the implications of the site’s revised chronology for our understanding of the Transition in Iberia. Detailed explanation of the approach and analytical protocols is given in the Materials and Methods section and in the S1 File.

Throughout, individual dating results will be quoted with their 68.3% confidence intervals, in the format “age ± standard deviation,” following standard reporting conventions of radiocarbon and luminescence dating laboratories. However, the 95.4% confidence interval of these ages is used when comparing their equivalent time ranges in calendar years. Note that ages are given in years or thousands of years (ka) Before Present (BP), i.e., counting back from: 1950 CE, in the case of radiocarbon; the time of sample collection, in the case of OSL (2017 CE); 2000 CE, in the case of the Greenland oxygen isotope record’s stadial (GS, Greenland Stadial) and interstadial (GI, Greenland Interstadial) boundaries [28].

### 1.2. Site description and excavation history

In plan, the cave is a meandering passage developed along two perpendicular axes, roughly oriented N-S and E-W and featuring broadly parallel walls. This morphology made it possible to divide the site into different sections, as shown in Figs 2 and 3. The extant intersection with the cliff face forms an almost oval cave mouth, *c*. 2.2 m-high and *c*. 1.4 m-wide (Fig 2B), opening exteriorly onto a *c*. 4 m-wide triangular platform. The excavation of the latter’s shallow, recent sediment cover in columns 21–22 of the grid revealed that this enlarged porch was created by retreat of the cliff face and attendant loss to erosion of the northern wall and roof of the original entrance; upon reaching the Pleistocene deposit, the trench revealed that the configuration of this sector is in line with that observed inside (Figs 2C, 2D and 3).

Immediately inward of the mouth, the cave features a slumping roof that descends to within 60 cm of the infilling sediment at a narrow point where the passage features a 90° angle. This initial stretch of the cave interior was not excavated. Thereafter, the passage featured a nearly horizontal sedimentary floor, and the roof rose again until another narrow was reached, in squares N-O/13-15 of the grid; here, roof saliences lay within *c*. 40 cm of the ground. The section of the site located between (and including) the two narrows is called the Corridor (squares K-O/13-17; Fig 2E). It opens at a right angle onto the Back Chamber, which includes squares N-S/8-12 and P-Q/13-15 of the excavation grid (Fig 2F). This last section is *c*. 7.5 m long and is where the cave reaches its widest point (at the surface of the infill, the walls were *c*. 3 m apart). The floor of the Back Chamber was, on average, *c*. 2 m below the roof, which features boulder- or speleothem-cluttered chimneys that may have once been open to the surface; for instance, the chimney above squares P-Q/14-15 reaches 1.30 m above the site’s datum, just slightly below the elevation of the rocky platform extending northward of the cliff edge, where pit caves of speleological interest are known to exist.

A survey carried out in the summer of 1979 found fossilised horse teeth on the surface of the Back Chamber’s infill, indicating the presence of a buried Pleistocene deposit. The excavation of the site began here, in the following winter. By 1982, a 3×3 m trench (squares P-Q/11-13) had been taken down to the Solutrean, 2.5 m below the surface, and a test had been opened in square M14 of the Corridor. Eventually, the intervening squares were excavated, and a continuous, square-angled stratigraphic profile spanning L15>16 to Q>R11 was recorded down to the elevation of the uppermost Solutrean unit, layer Fa. In the final phase of the project, between 1985 and 1988, the post-Solutrean loose sediments of the Back Chamber were completely removed for safe excavation of the underlying, consolidated deposit (carried out in row P of the grid and in square O12), while the Corridor was surface-excavated down to the base of the Solutrean (except for squares O/13-14, which reached the base of the Early Upper Palaeolithic).

**Fig 4:**
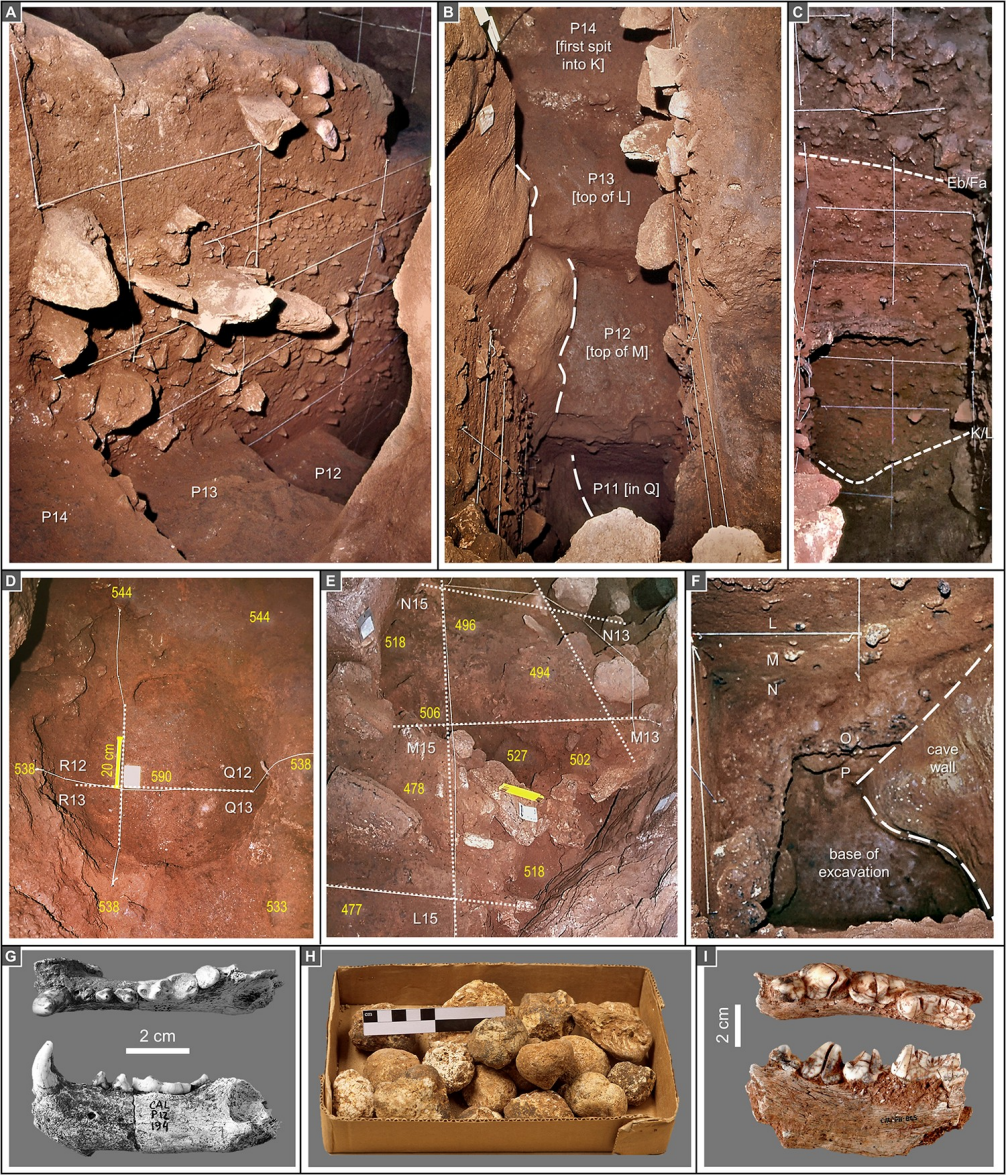
Stratigraphy and disturbance (features and agents). **A.** The P>Q14-11 profile at the end of the excavation, seen from the angle between Corridor and Back Chamber. **B.** Zenithal view of the stepped base of the Back Chamber trench at the end of the excavation; the long-dash lines mark the contour of the cave wall; the stratigraphic depth reached in each grid unit is indicated. **C.** The P11>10 profile; the short-dash lines denote the stratigraphic succession’s two major discontinuities. **D-E.** The large, *c*. 50 cm-deep burrows penetrating layers Fa-Ja in squares Q-R/12-13 and L-N/13-15; elevations are in cm below datum. **F.** The base of the P11>10 profile seen in oblique view taken from the opposite edge of grid unit P11; note how, along the south and west walls of the trench, layer O formed a thick, hard crust. **G.** *Meles meles* (badger) left mandible (P12-194/sc358, layer Fa). **H.** Sample of the large concentration of hyaena coprolites retrieved in the NE corner of square P11 (P11sc907, layer M). **I.** *Crocuta crocuta* (spotted hyaena), right mandible (P11-865, layer L).

**Fig 5:**
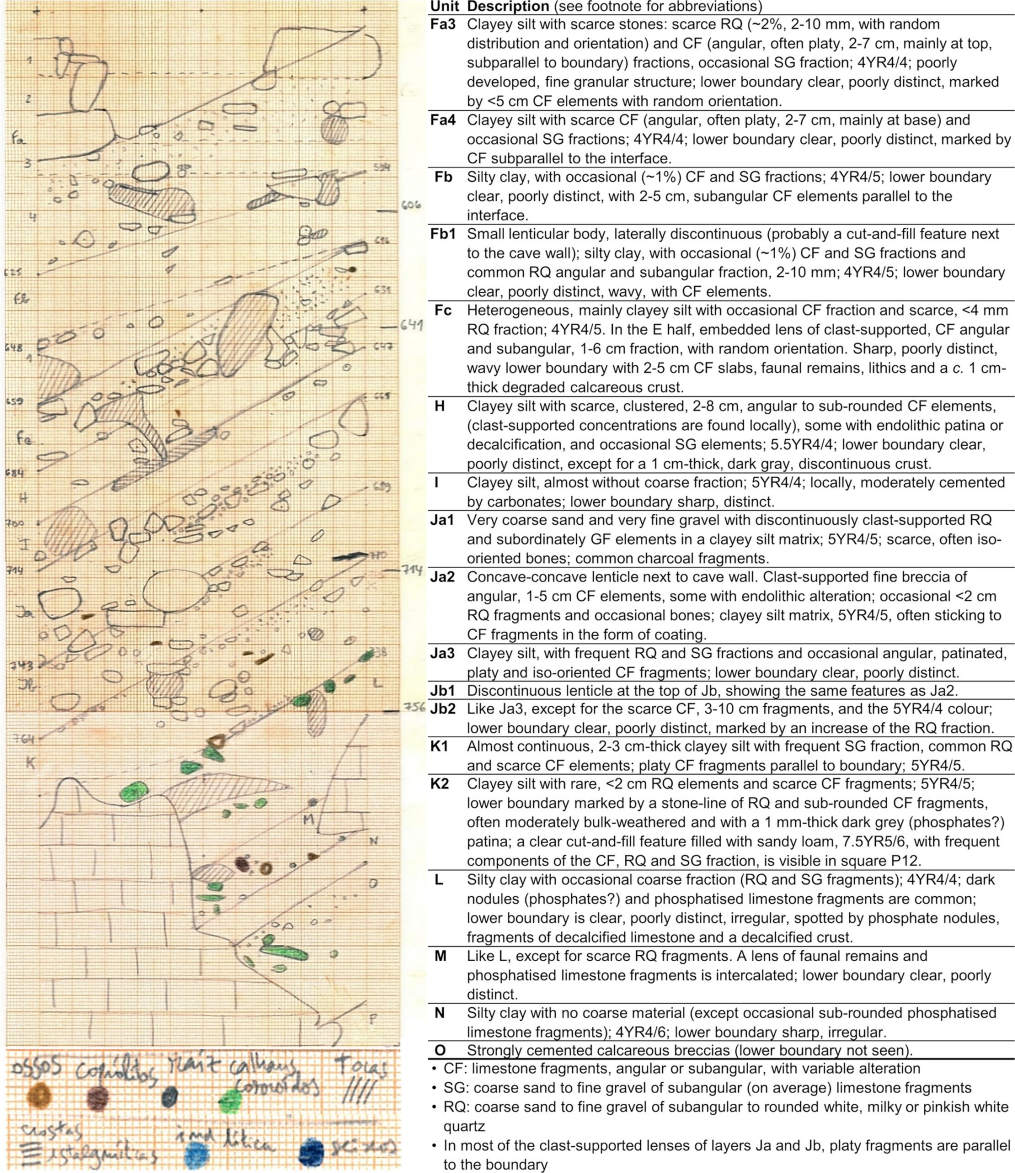
The Pleistocene succession. Illustration (original field record, hand-drawn at 1:10; elevations in cm below datum) and geological description of the P>O11 (North) profile of square P11 extant at the end of the 1979–88 excavations (completed with observations made in P11>10 and P>Q11-13).

**Fig 6:**
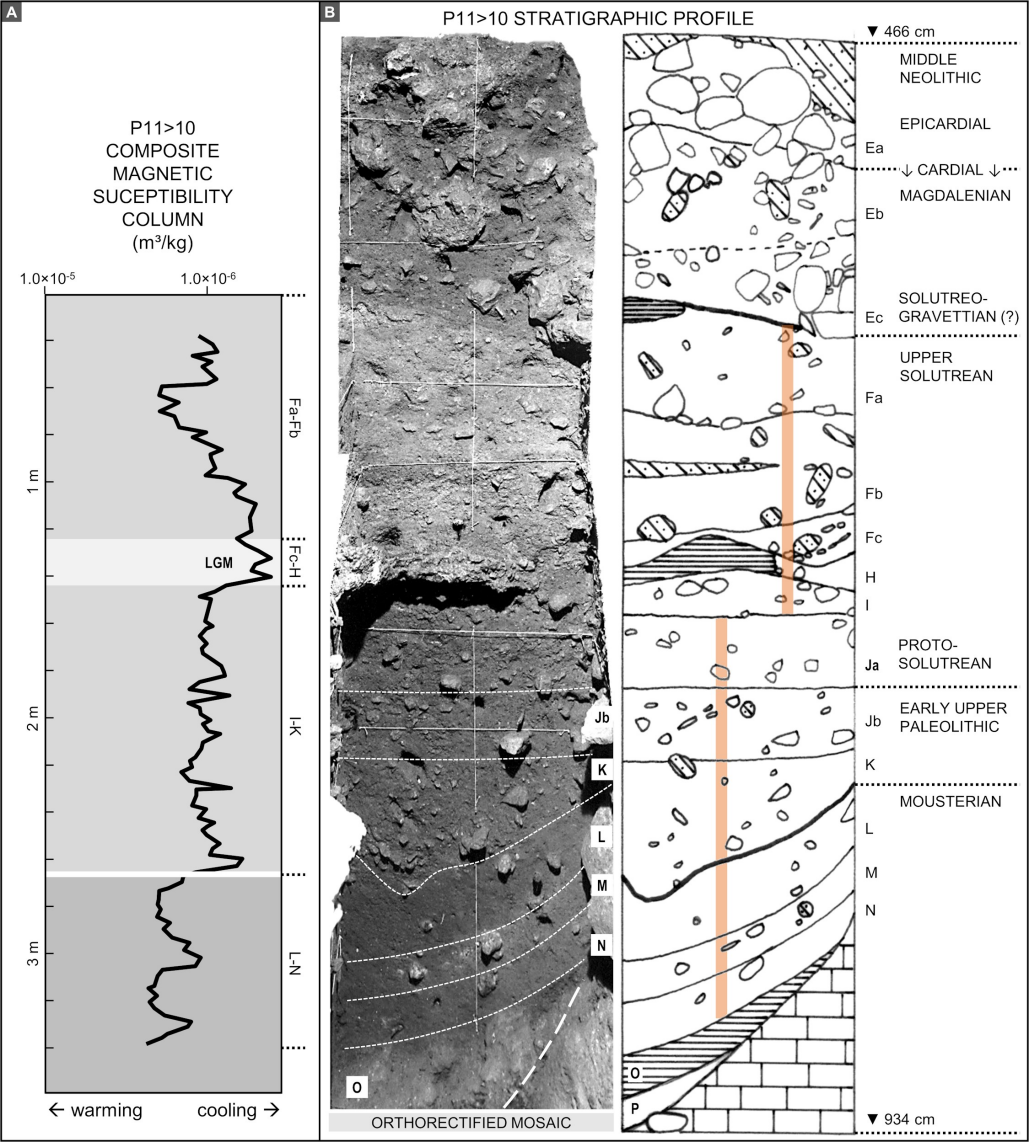
The archaeological sequence. **A.** Magnetic susceptibility curve (log scale) [2, 29]. **B.** Orthorectified photo mosaic and stratigraphic interpretation of the P11>10 profile (based on an original field record drawn at 1:10; elevations are below datum); the colour bands indicate the position of the magnetic susceptibility sampling columns.

The project’s limited exploration of the basal Middle Palaeolithic is explained by research priorities, the thickness of the succession, the morphology of the passage, and a conservation mandate: that a substantial part of the infill be preserved for future investigations. In the Back Chamber and Corridor, the Middle Palaeolithic deposit was exposed in squares O/13-14 and P13, and its uppermost stratigraphic unit was excavated in square P12; only in square P11 could it be probed over a thickness of *c*. 1.25 m, down to *c*. 6 m below the surface of the infill (Figs 4–6). In the Entrance, the Middle Palaeolithic deposit was shallow across most of the trench, but in S20 could be tested for *c*. 1 m without reaching bedrock (Fig 2C and 2D). New excavations targeting the site’s Transition levels are planned for 2021–22.

## 2. Results

### 2.1. Back chamber and corridor

#### 2.1.1. Stratigraphic succession

As best seen in square P11, the cave’s sedimentary infill can be divided into three parts separated by two major discontinuities: one between layers Eb and Fa and the other between layers K and L, both clearly apparent in profile view (Fig 4C). The matrix is mostly clayey in the lower part (layers L to P), mostly silty in the middle part (layers Fa to K), and mostly sandy in the upper part (layers ABC-D to Eb).

Inside the cave, higher-up in the walls and roof, there are small, thin, and isolated plaques of a cemented, fossiliferous breccia that contain rabbit teeth and bones. They testify to an earlier, Middle Pleistocene cycle of sedimentary accumulation, followed by an episode of karst reactivation and attendant erosion that almost completely removed an older deposit prior to the accumulation of the extant infill. Substantial portions of this breccia are preserved in the Entrance, where it was left exposed at the basis of the excavated trench in squares P-R/20 (Fig 2C).

Layer ABC-D was excavated in totality. It was a loose, dark, and organic matter-rich cave earth deposit, traversed by roots and supported by a dense network of tree and shrub rootlets entering the cave via the porch and the roof’s chimneys. At the bottom end of the Back Chamber, this deposit was up to 1.5 m thick. Across the site, layer ABC-D was extensively tunnelled by burrowing animals, mostly badgers (in places their underground nesting chambers remained intact, forming large voids within the body of the layer). The archaeological content consisted of an unstratified mix of abundant human remains as well as pottery, stone, bone, and metal tools documenting continued funerary usage of the place between the Late Neolithic and Early Medieval times [1, 6, 30].

Layer Ea is a dense accumulation of large boulders. It is *c*. 50 cm-thick at the bottom end of the Back Chamber and wedges out at the intersection with the Corridor. The boulders reflect an episode of roof collapse and upward development of the cave’s chimneys. The brown, sandy matrix is in part made up of fines derived from the surface of the underlying, uppermost Pleistocene unit: layer Eb, which, at the time of formation of layer Ea, remained exposed as the cave floor in the Corridor, upslope. Abundant human remains, pottery, stone tools, and body ornaments document funerary usage of the place by bearers of the Epicardial culture (later part of the Early Neolithic of west Mediterranean Europe) [1, 6, 30].

Layer Eb is a reddish-brown sand with some éboulis. This deposit is significantly bioturbated, especially along the walls. In the Back Chamber, badgers were probably the agent; skeletal remains were retrieved in underlying layer Fa (Fig 4G), and the disturbance features penetrating deeply into underlying units (Fig 4D) are comparable in shape and size to the hollows seen in layer ABC-D. In the Corridor (Fig 4E), the abundant rabbit bones suggest that denning by lynx must also be contemplated, even though study of the lagomorph remains indicates that they were primarily accumulated by humans [31]. Reflecting the bioturbation, the artefact and bone assemblages are heterogeneous. The lithics and the fauna are mostly Magdalenian but include some upwardly displaced Solutrean items. In the Back Chamber, the most conspicuous components are decorated potsherds, objects of personal ornamentation, and the associated human skeletal remains documenting funerary usage of the place by people of the Cardial culture. These finds were originally laid down on the surface of the Magdalenian deposit and were eventually incorporated subsurface by post-depositional processes [1, 6, 30]. This pattern implies a major hiatus in deposition during the Early Holocene.

The dip of the stratification changes significantly, from only *c*. 5–10° in Holocene layers ABC-D and Ea to *c*. 15–25° in the Solutrean and underlying layers. This change suggests that the passage continues beyond the Back Chamber, via a siphon-like, completely filled narrow, and that inward dispersal of sediments along a free slope ceased after the Solutrean. The cluttering-up of the inferred narrow must therefore have been completed during the accumulation of Magdalenian layer Eb, and probably began with the formation of a basal subdivision of layer Eb that could only be differentiated in the P11>10 profile and probably stands for the external edge of a deposit thickening inwards, in columns 8–10 of the grid and beyond (labelled as “Ec” in Fig 6B).

Detailed description of the middle and lower parts of the Back Chamber succession is provided in Fig 5. These units are much less affected by erosion and bioturbation, except at the K/L interface, where a large cut-and-fill feature is to be seen; at that elevation, the disturbance must have been caused by hyaenas, whose activity is documented by skeletal remains and coprolites in layers L and M (Fig 4H and 4I), and by digested bones in layer K [32, 33]. In general, these parts of the infill correspond to the relatively monotonous succession of a fine, reddish sediment with variable amounts of coarser (>2 mm) elements. The deposit is massive (neither sedimentary features nor aggregation can be recognised), moist, moderately firm, and devoid of organic matter. Coarse components—mostly, limestone fragments—vary laterally in all units, and are especially abundant near the wall. No significant changes are apparent in the sedimentary sources of the inwashed material. Such relative homogeneity makes for layer boundaries of poor distinction: inter-layer variation is quantitative only, reflecting changes in the abundance and composition of the coarse fraction. Boundary recognition is based on such changes and, often, the presence at the boundary itself of sedimentary structures indicative of hiatuses (e.g., rills or stone lines).

The Eb/Fa and K/L discontinuities are clear in geometry and texture (Figs 4C and 6). Both separate finer sediment below the boundary from coarser sediment above it. Owing to moderate carbonate precipitation during a short hiatus in sedimentation, resulting in the laterally variable cementation of the then-extant surface, a minor but quite visible discontinuity exists at the Fc/H boundary. This hiatus was followed by the accumulation of the chaotic-like slope sediment forming layers Fa-Fc, atop which there was a 5 cm-thick stalagmite crust of limited extent (cut by the excavation but visible in the P-Q11>10 profile). Despite the overall thickness (*c*. 1 m) of the package, layers Fa-Fc accumulated over a rather short time span, as indicated by their homogeneous archaeological content (see below).

Water-related sedimentary features and fabric are obvious in layers Fa-K, indicating that erosion outside the cave was affecting soils, the exposed sedimentary cover and, partly, the local calcareous bedrock. Between sedimentation pulses, short periods of stabilisation and subsequent accumulation of secondary carbonate or phosphate are nonetheless apparent. In layers Ja, Jb, and K, these alternations are revealed by their differentiation into subunits, visible in profile view (Fig 5) and indicative of sediment inwash occurring as pulses (sometimes with an increase of energy, as seen at the base of layer Jb). The sedimentary facies of such subunits relate to slope dynamics with intermediate sediment concentration (e.g., runoff or overland flow), while higher-up, in layers Fa-Fc, the high sediment concentration denotes a shift in the dynamics of the accumulation [34].

Layers O-Fc represent a cycle of climate worsening modulated by small-scale fluctuations [29], followed by a cycle that reverses the trend but becomes impossible to read beyond the top of layer Fa because of bioturbation (Fig 4). After a phase of strong secondary carbonate precipitation that partially cemented layer O, the inwash of soil-sediment from the outside indicates the onset of slope instability in the cave’s surroundings, which gave rise to the accumulation of layers N-L. These erosive pulses, recorded in the cave’s sediments as correlative deposits, alternated with short phases of stability during which secondary carbonate accumulation and phosphate enrichment occurred. Through time, erosive processes outside gradually increased, as revealed in layers K-Fc by the inwash of coarser components (e.g., quartz sand and fine gravel) derived from two sources: the Late Pliocene/Early Quaternary alluvium that covers the karst outcrop in which the cave is located; and the partial erosion and downslope redeposition of the cave’s outwardly located sedimentary floors.

#### 2.1.2. Archaeological sequence

**Fig 7:**
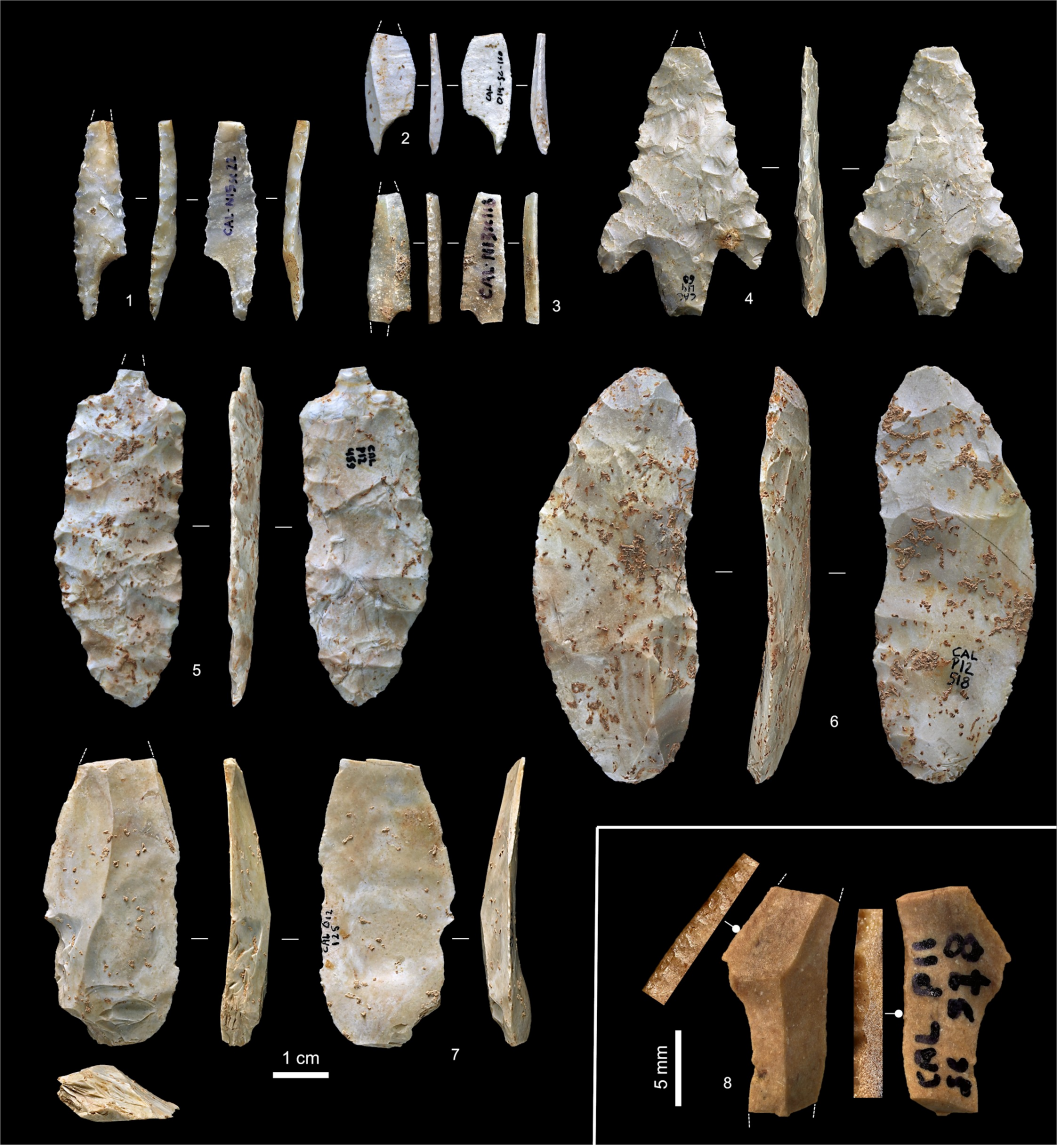
Solutrean diagnostic artefacts. **1–3.** Shouldered points with impact fractures from layer ABC-D (N15sc22; Franco-Cantabrian subtype; upwardly displaced) and basal layer Eb of the Corridor area (O14sc160 and N13sc118; Mediterranean subtype; Solutreogravettian?). **4.** Parpallò point with impact fracture, layer Fa (L14-60; in burrowed area); **5.** Broken laurel-leaf modified into perforator, layer H (P12-459); **6.** Endscraper on ventrally thinned blank, layer I (P12-518). **7.** Vale Comprido point with distal impact fracture, layer Ja (O12-125). **8.** Dufour bladelet (mesial fragment), with zoom-in views of its direct (left side) and inverse (right side) retouch, layer Ja (P11sc978).

The content of layers Fa-Ja illustrates the phasing of the Solutrean as originally defined [10, 35, 36] and since corroborated in both south-western France and southern Iberia [16, 37, 38]. At Caldeirão, the phasing is manifested by the stratigraphically ordered appearance of the technocomplex’s index fossils [2] (Fig 7). The upward displacement of such diagnostics, including bifacial thinning flakes, is, however, significant: out of a total of 69 items, 26% were found in layer Eb and 4% ended-up in layer ABC-D (Table 1).

**Table 1:** Solutrean diagnostics.

	ABC-D	Eb	Fa	Fb	Fc	H	I	Ja	TOTAL
Shouldered point (Mediterranean)	–	3	–	–	–	–	–	–	3
Shouldered point (Cantabrian)	1	–	2	–	–	–	–	–	3
Shouldered point fragment	–	–	1	–	–	–	–	–	1
Parpallò point	–	–	1	–	–	–	–	–	1
Parpallò point fragment	–	–	–	1	–	–	–	–	1
Laurel-leaf	–	–	–	–	–	1*	–	–	1
Laurel-leaf fragment	–	3**	–	2	–	–	–	–	5
Bifacial piece fragment	–	1	–	–	1	1	–	–	3
Foliate preform	–	1	1	–	1	–	–	–	3
Foliate fragment	–	–	2	–	–	–	–	–	2
Vale Comprido point	–	1	–	–	–	–	–	3	4
Vale Comprido point fragment	1	–	–	–	–	–	–	–	1
Unifacial point	–	1	–	1	–	–	1	–	3
Point fragment	1	1	–	–	–	1	1	–	4
Blade with flat retouch	–	–	–	–	–	–	–	–	1
Solutrean endscraper	–	1	–	1	1	3	1	–	7
TOTAL	3	13	7	5	3	6	3	3	43
Bifacial thinning flake***	–	5	17	–	3	1	–	–	26

Stratigraphic distribution

* transformed into perforator after breakage

** includes the basal half of a laurel-leaf point found in Fb

*** twenty of these (1 in Eb, 17 in Fa, 2 in Fc) derive from the reduction of the foliate preform in Fa.

Reflecting the stratigraphic depth reached by some of the larger burrows excavated in the Corridor during the post-Fa hiatus (Fig 4E), the set of displaced finds includes items indicative of all phases: Vale Comprido points, laurel-leaves, and shouldered points. A corollary of this evidence is that the original assemblages’ other retouched tool types and debitage must have suffered comparable losses. Therefore, traditional typological counting would be too biased for meaningful inter-site comparative analysis. For this reason, presentation of the typology and stratigraphic distribution of Solutrean stone tools must be restricted to the diagnostic finds and, here, full counts are provided only for the basal, Early Upper and Middle Palaeolithic levels of the sequence.

Layer Ja yielded three Vale Comprido points in association with Dufour bladelets (Fig 7, nos. 7 and 8), as is typical for the Protosolutrean [2, 37, 39, 40]. Layers I and H yielded items featuring the characteristic flat, invasive retouch seen in the Lower and the Middle Solutrean, both unifacially (Fig 7, no. 6) and bifacially (Fig 7, no.5). Layers Fa-Fc yielded the typical Upper Solutrean mix of barbed-and-tanged (Parpallò) and shouldered points (Fig 7, nos. 1–4). The latter are also well represented among the upwardly displaced material and include both the Franco-Cantabrian (made by flat, invasive retouch) and the Mediterranean (backed) varieties. The small size of the latter (Fig 7, nos. 2–3) is consistent with the Solutreogravettian, which is the terminal phase of the technocomplex in Mediterranean Spain and Portugal but, at Caldeirão, does not occur as a separate stratigraphic unit; its representation at the site can only be inferred from such diagnostic items.

**Fig 8:**
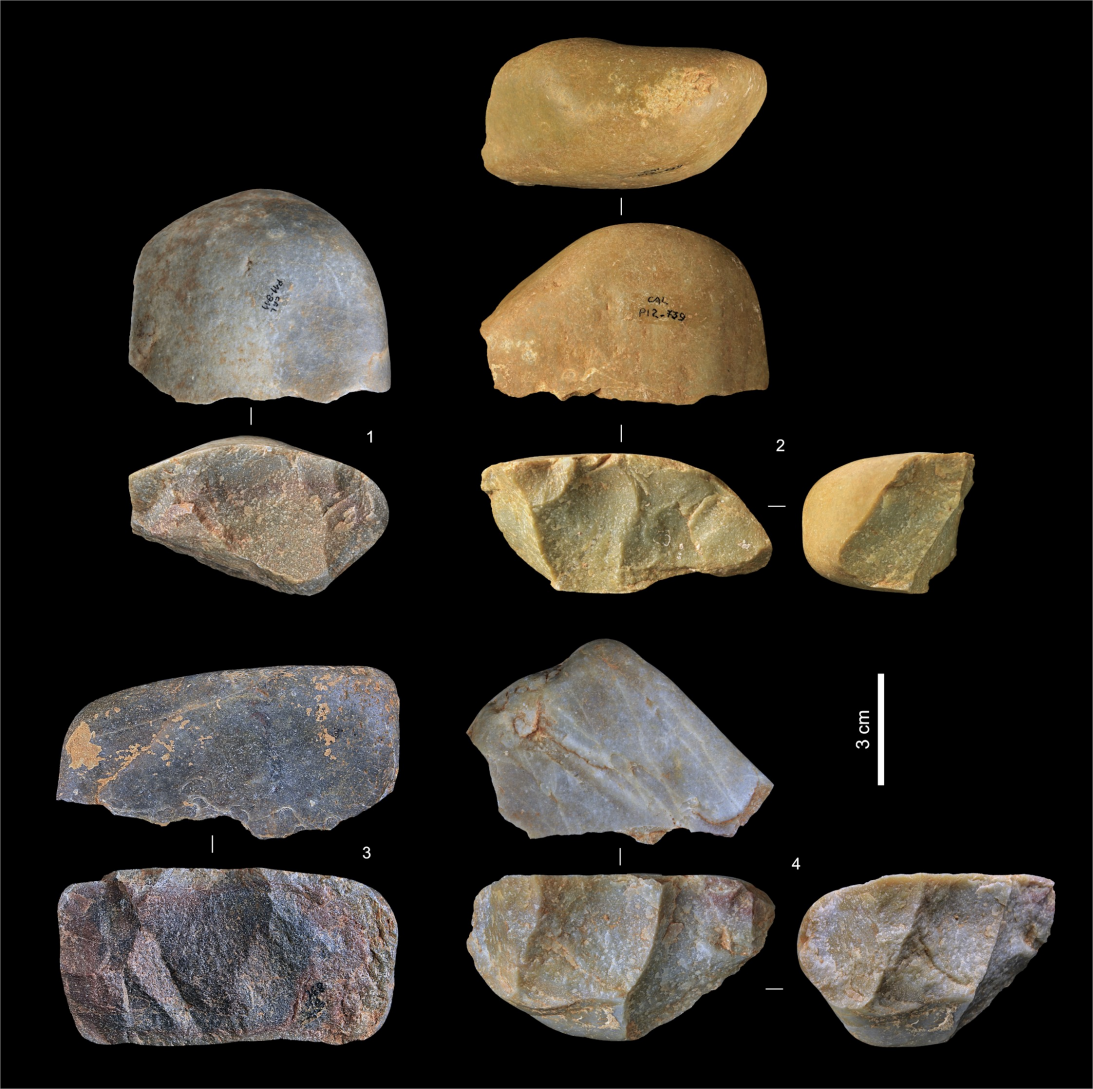
Quartzite cores from layers Jb-L. **1.** P11-811, layer K. **2.** P12-739, layer K (possibly also used or re-used as a hammerstone). **3.** P11-831, layer L. **4.** P11-796, layer K.

**Fig 9:**
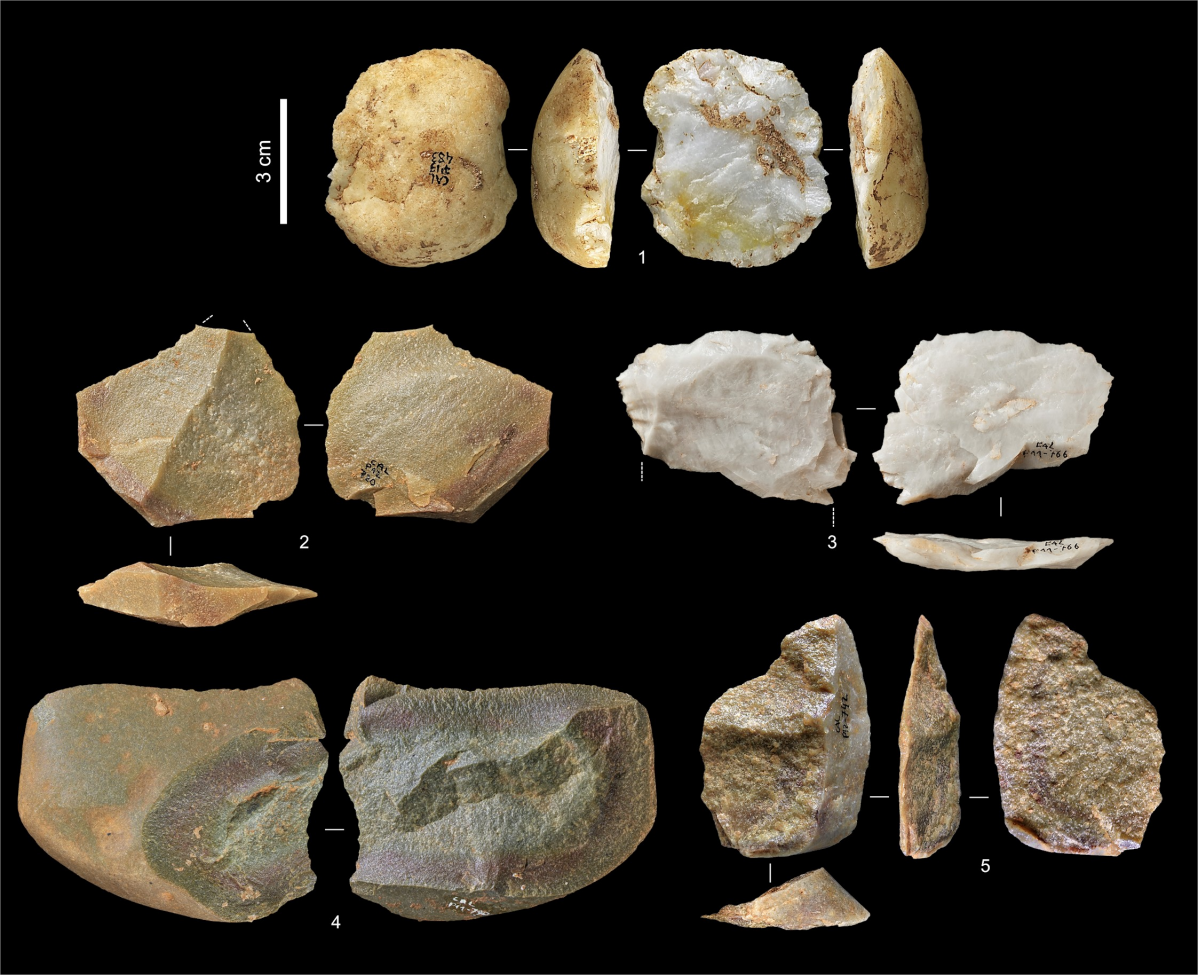
Non-chert débitage from layers Jb-L. **1.** Cortical flake, quartz (P13-483, layer L). **2.** Flake with distal-marginal micro-denticulation of the edges (use-wear?), quartzite (P12-720, layer K). **3.** Levallois flake, distal fragment, quartz (P11-766, layer K). **4.** Flake, quartzite (P11-790, layer K). **5.** Flake, quartzite (P12-742, layer K).

**Fig 10:**
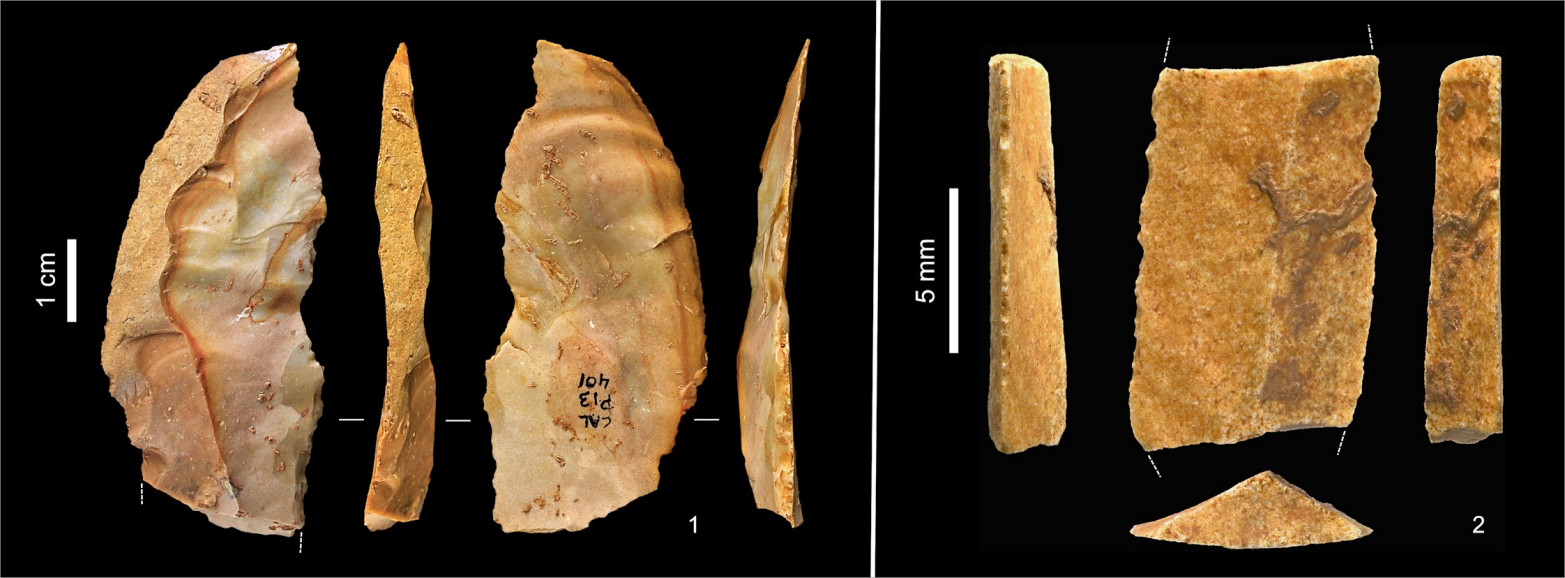
Chert tools from layers Jb-L. **1.** Unilaterally retouched, naturally backed blade (P13-401, top of layer K). **2.** Dufour bladelet, mesial fragment (P11sc980, top of layer L).

**Fig 11:**
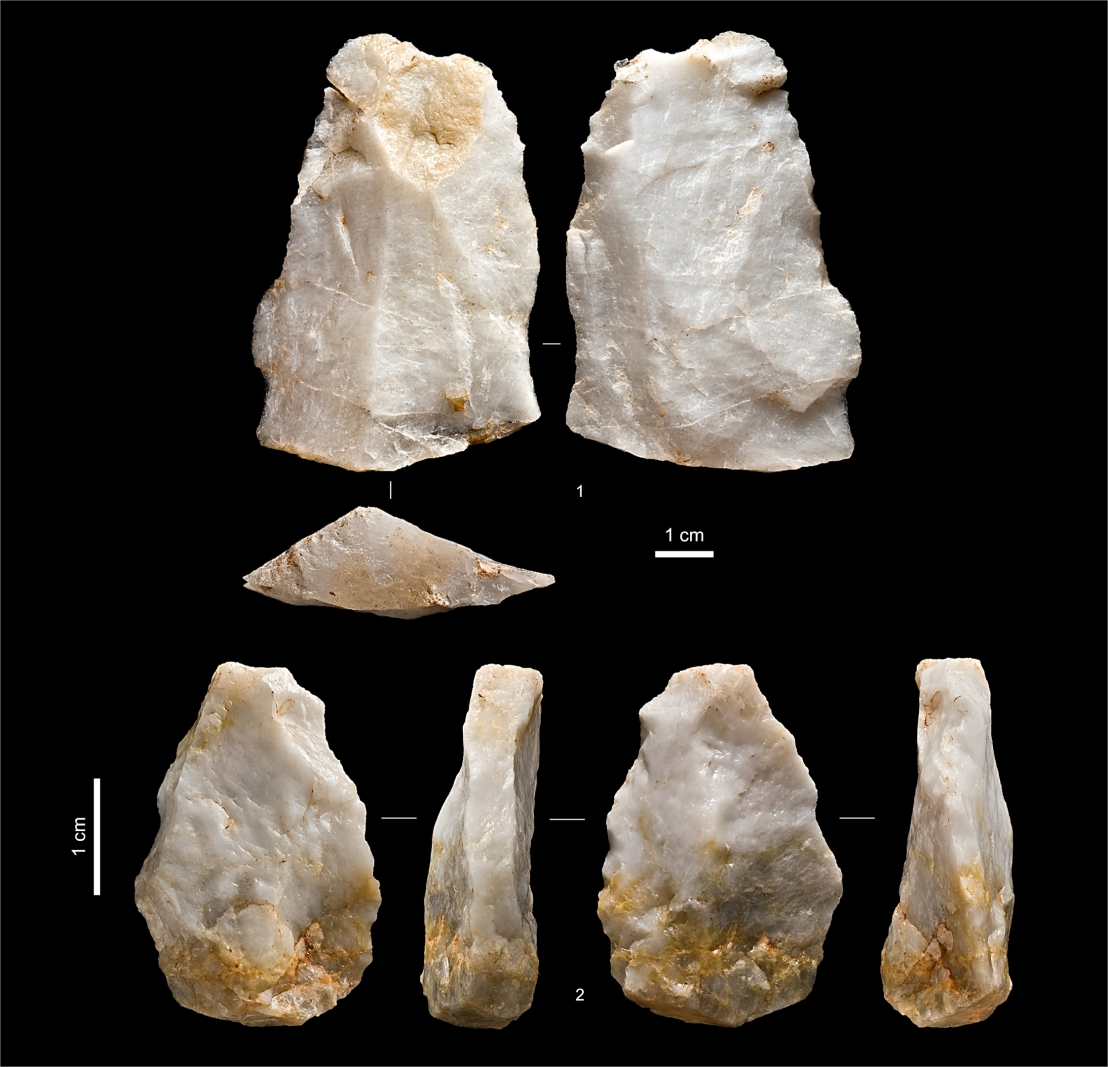
Quartz tools from layers K-L. **1.** Sidescraper (bilateral, inverse retouch; O13-361, surface of layer L). **2.** Sidescraper (bilateral, alternate retouch; P11sc852-3, base of layer K).

Layers Jb and K yielded small stone tool assemblages with no culture-stratigraphic diagnostics; they are dominated by the products and by-products of the expedient exploitation of quartz and quartzite cobbles (Figs 8 and 9; Tables 2 and 3). The nine type-list items in layer Jb include a notched bladelet, a cortically backed, continuously retouched blade (Fig 10, no. 1), and a splintered piece (from a techno-economic perspective, a core rather than a tool [2]); all the others are atypically modified specimens. Pretty much the same applies to layer K, where, despite the slightly higher numbers, chert is even scarcer, which helps to explain why type-list tools are even less numerous: three atypically retouched pieces, a denticulate, and a sidescraper (Fig 11, no. 2), the latter two of quartz and found in square P11, in the layer’s basal spit. A Levallois flake on quartz (Fig 9, no. 3) had the same provenance as those quartz tools. In addition, while layer Jb yielded a few items indicative of the presence of an Upper Palaeolithic technology (namely, three bladelets, one of chert and two of hyaline quartz), layer K yielded none; its two quartzite blades are partly cortical items so classified on account of length/width ratios that accidentally fall in the category (i.e., those two items are blades metrically but not technologically; they were extracted from cores like those illustrated in Fig 8, not from prismatic blade cores).

**Table 2:** Back chamber stone tools (layers Jb-P).

		Chert	Quartz	Quartzite	Hyaline Quartz	Total
Layer Jb	Core	2	1	1	–	4
	Bladelet	1	–	–	2	3
	Flake	1	3	5	–	9
	Flake fragment	4	4	8	1	17
	Retouched tool	7	–	1	–	8
	Chippage	4	7	6	3	20
	Chunk	–	–	3	–	3
	Manuport	–	1	1	–	2
	Total	19	16	25	6	66
Layer K	Core	–	2	5	–	7
	Blade	–	–	2	–	2
	Bladelet	–	1	–	–	1
	Flake	4	9	14	–	27
	Levallois flake	–	1	–	–	1
	Flake fragment	5	5	14	–	24
	Retouched tool	1	2	2	–	5
	Chippage	4	6	4	1	15
	Chunk	–	2	5	–	7
	Manuport	–	1	1	–	2
	Total	14	29	47	1	91
Layer L	Core	–	–	1	–	1
	Flake	–	2	2	–	4
	Flake fragment	–	2	4	–	6
	Retouched tool	1	1	–	–	2
	Chippage	1	1	2	–	4
	Chunk	1	–	3	–	4
	Total	3	6	12	–	21
Layer M	Flake	1	–	1	–	2
	Chippage	–	1	–	–	1
	Total	1	1	1	–	3
Layer N	Flake	–	–	1	–	1
	Levallois flake	1	1	–	–	2
	Chunk	1	–	–	–	1
	Total	2	1	1	–	4
Layer O	Flake	1	–	1	–	2
	Levallois flake	1	–	–	–	1
	Small flake	1	–	–	–	1
	Flake fragment	2	–	–	–	2
	Retouched tool	–	–	1	1	2
	Chippage	2	1	–	–	3
	Chunk	–	1	–	–	1
	Total	7	2	2	1	12
Layer P	Levallois flake	1	–	–	–	1
	Small flake	–	1	–	–	1
	Flake fragment	1	1	1	–	3
	Chippage	1	–	–	–	1
	Chunk	1	–	–	–	1
	Total	4	2	1	–	7

Inventory per techno-economic category (Transition and Middle Palaeolithic levels).

**Table 3:** Back chamber lithic typology (layers Jb-P).

	Layer Jb		Layer K			Layer L		Layer O	
	Chert	Quartzite	Chert	Quartz	Quartzite	Chert	Quartz	Quartz	Quartzite
Atypical endscraper	1	–	–	–	–	–	–	–	–
Endscraper on retouched flake	–	–	–	–	–	–	–	–	–
Unilaterally retouched blade	1	–	–	–	–	–	–	–	–
Notched piece	–	–	–	–	–	–	–	–	–
Denticulated piece	–	–	–	1	–	–	–	–	1
Splintered piece	1*	–	–	–	–	–	–	–	–
Simple sidescraper	–	–	–	1	–	–	–	–	–
Double sidescraper	–	–	–	–	–	–	1	–	–
Notched bladelet	1	–	–	–	–	–	–	–	–
Dufour bladelet	–	–	–	–	–	1	–	–	–
Atypically retouched piece	4	1	1	–	2	–	–	1**	–
Retouched piece fragment	–	–	–	–	–	–	–	–	–
Total	8	1	1	2	2	1	1	1	1

Retouched tool-type counts (Transition and Middle Palaeolithic levels)

* counted as core in Table 2

** hyaline quartz.

**Fig 12:**
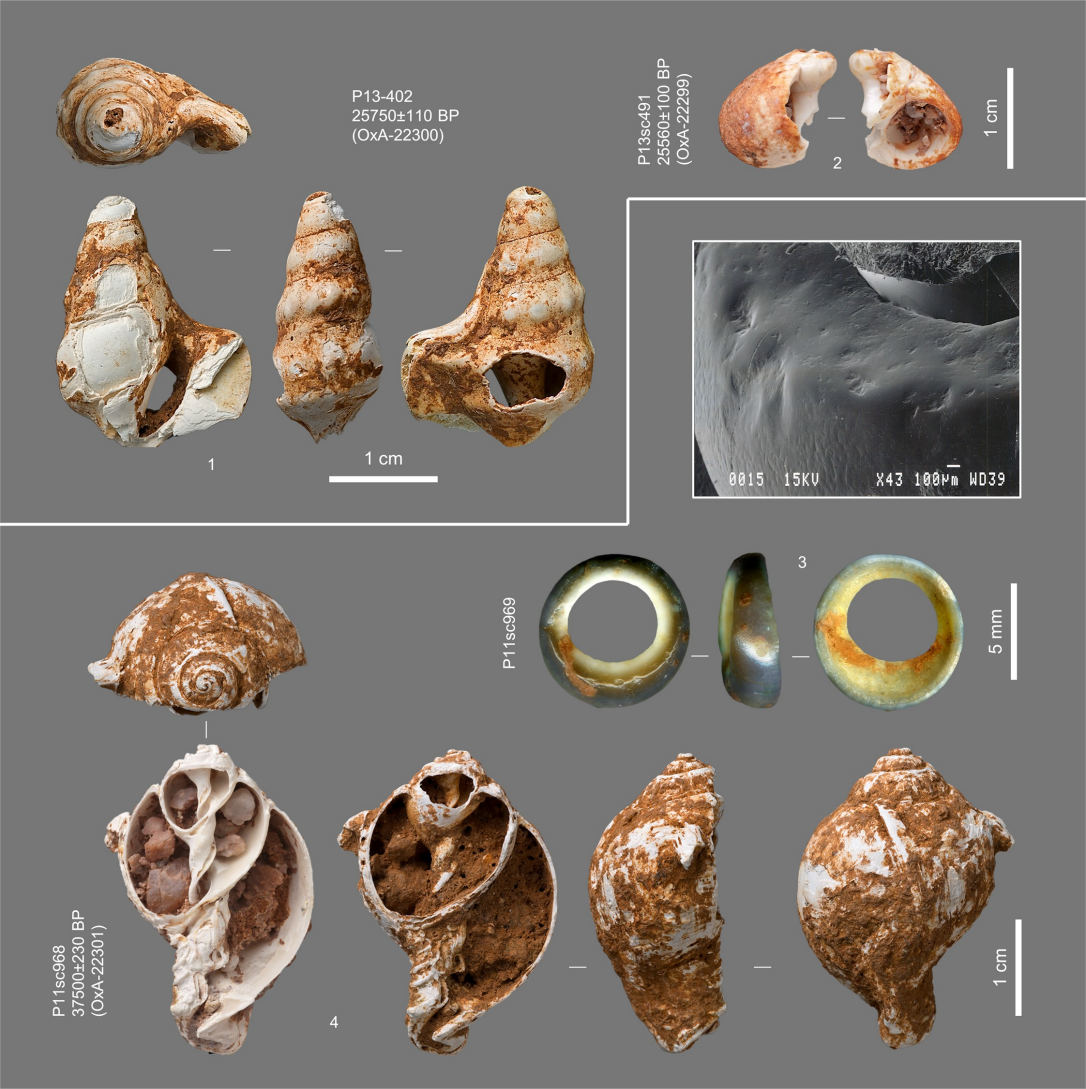
Personal ornaments in layers Jb-K and their radiocarbon ages. **1.** Perforated *Aporrhais pespellacani* shell. **2.** Broken *Littorina obtusata* shell. **3.** Worn-down *Sparus auratus* tooth (undated) and SEM image of the natural wear of the occlusal surface. **4.** Eroded *Semicassis saburon* shell (leftmost ventral view taken after removal of the infill, showing the characteristic coarse sand/small pebble matrix of its layer of provenance). Nos. 1 and 2 are from layer Jb, nos. 3 and 4 are from layer K.

The *Semicassis saburon* shell illustrated in Fig 12 (no. 4) is a sieve find from the eastern half of the K1 spit of square P11, previously published as coming from layer Jb [2, 18]. However, K1 was a horizontal spit that cut across the Jb/K boundary (see Materials and Methods for an explanation of why this was so). Because (a) at the time of excavation, body ornaments were thought to be exclusive to the Upper Palaeolithic and (b) layer K was then thought to belong in the Middle Palaeolithic, the uncertainty was resolved in favour of layer Jb at the time of original publication. Since, the shell’s infill has been excavated to look for micro-samples of charcoal that might be amenable to radiocarbon dating. As the matrix revealed in the process matches the coarse sand/small pebble lens forming the surficial lens of layer K across the Back Chamber trench, the item’s stratigraphic assignment is herein corrected.

The *Sparus auratus* tooth from layer K (Fig 12, no. 3) is the only fish remain found in the excavation. Likely, it is an item of personal ornamentation. However, its SEM (Scanning Electron Microscopy) analysis showed no evidence of human modification: the ring-like shape results from wear in the living animal. Likewise, the ventral erosion of the *Semicassis saburon*, which would have enabled use as a pendant (e.g., via threading around the exposed columella), results not from human modification but from natural abrasion; along the shores of coastal Portugal, this naturally modified morphology is commonly observed among beached shells of the taxon. Likewise, no evidence was found that the perforations seen on the other two marine shells completing the ensemble of personal ornaments from Caldeirão’s pre-Solutrean levels—the *Aporrhais pespelecani* and the *Littorina obtusata* from layer Jb (Fig 12, nos. 1 and 2)—are anything but natural. The only human modification seen in all three concerns the presence of ochre, as detailed in S1 File.

In layer L, quartzite (57.1%) and quartz (28.6%) predominate, and among its 21 items only two were diagnostic: a mesial fragment of a chert Dufour bladelet (Fig 10, no. 2), and a double sidescraper made on a large quartz blank (Fig 11, no. 1). The former comes from square P11 and was identified when lab-sorting the water-sieved sediment residue from the unit’s uppermost spit; clearly, it represents an Upper Palaeolithic item removed while exposing the K/L boundary in that square, where layer L was first encountered in testing and the boundary’s décapage was therefore approximate. A provenance issue also exists with the quartz sidescraper, but in this case one akin to the *Semicassis saburon* shell discussed above. That quartz sidescraper lay at the K/L boundary in square O13 and, through the excavation of the site, finds made during the last, fine-décapage stage of the exposure of stratigraphic interfaces were by convention recorded as belonging in the unit above the interface. Because (a) sidescrapers are characteristically Middle Palaeolithic type-list tools, (b) another quartz sidescraper had been retrieved at the base of layer K in P11 (see above), and (c) layer K was initially thought to belong in the Middle Palaeolithic, whether this item came from K or L was of little consequence at the time. These reasons explain why the item was labelled as “layer K” when first published and thereafter [2, 18]. However, the excavation records make it clear that this item lay on the exposed surface of layer L, and there is no evidence to suggest that a décapage error occurred (S1L Fig in S1 File).

**Fig 13:**
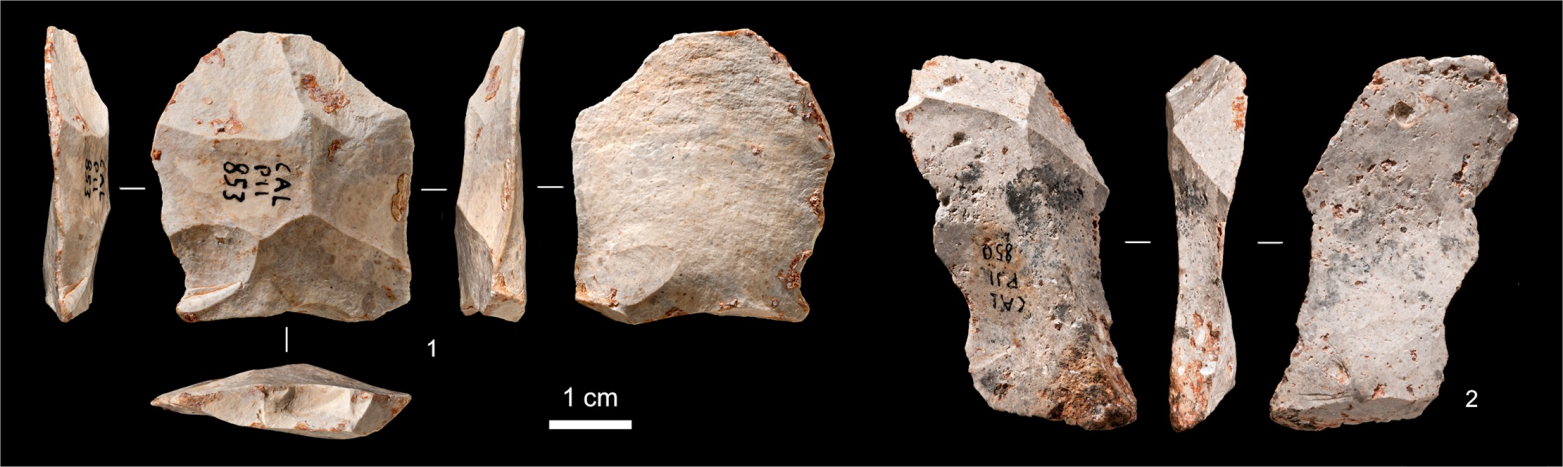
Chert flakes from layer O. **1.** Levallois flake (P11-853). **2.** Regular flake (P11-850).

Downward of layer L, the trench was restricted to square P11 and, as the sloping cave wall encroached on the north side of the square, the workable area became increasingly small, which eventually forced the excavation to halt at the elevation of layer P (Figs 4F and 6B). Thus, the limited number of artefacts in layers M-P (three in layer M, four in layer N, twelve in layer O, seven in layer P; Tables 2 and 3) remains broadly in proportion to the volume of excavated sediment. The layer M artefacts are two flakes and a chip, but a total of four Levallois flakes (three of chert, one of quartz) were retrieved in each one of layers N, O and P (Fig 13), further supporting the Middle Palaeolithic nature of the archaeology found below the K/L boundary.

#### 2.1.3. Radiocarbon dating

**Fig 14:**
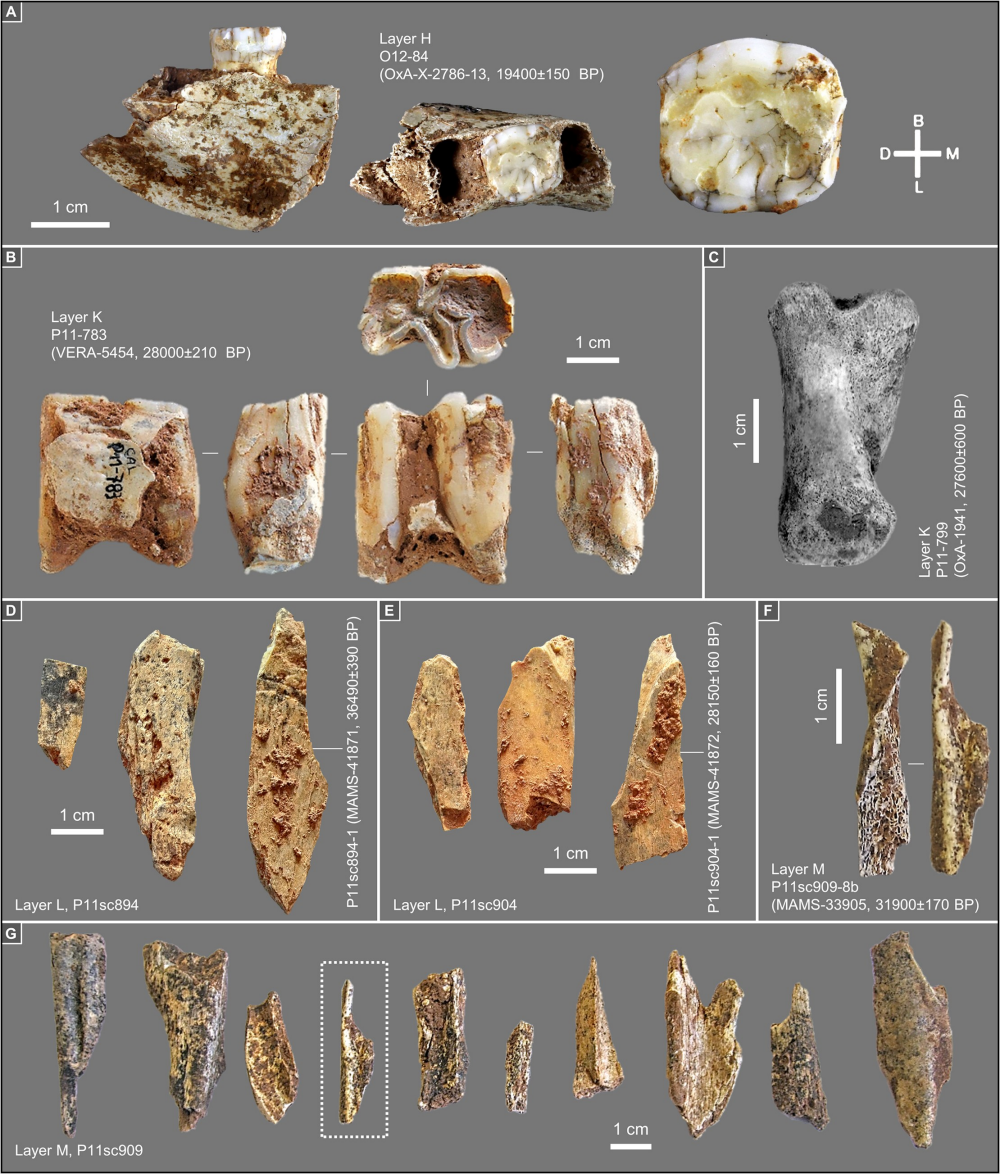
Back chamber’s upper and middle palaeolithic bone samples submitted for radiocarbon dating. **A.** Human mandibular fragment with dm2. **B.** Horse tooth (mandibular M1 or M2). **C.** Unfused red deer first phalange. **D-G.** Non-plotted shaft fragments (the items that yielded enough collagen are associated with age result plus inventory and lab numbers; the fragment indicated by the dotted rectangle in **G** is illustrated in **F**): spit L1 of layer L (**D**); spit L3 of layer L (**E**); spit M1 of layer M (**F, G**).

**Fig 15:**
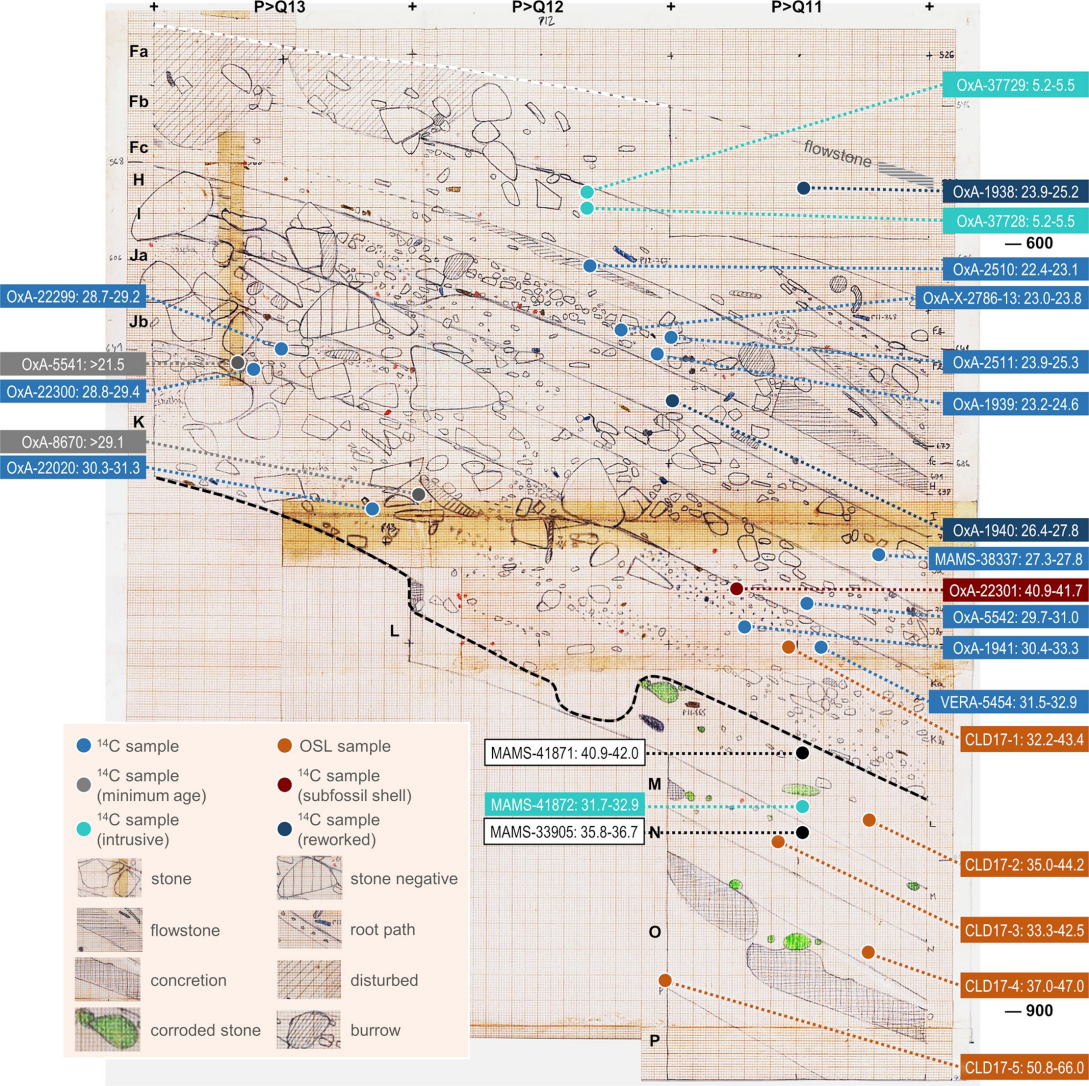
Stratigraphic provenance and significance of the back chamber’s pleistocene dating samples. OSL samples were taken from the P>Q13-11 profile at the indicated positions (except for the layer O sample, which was taken from the P11>12 profile at x = 30 and is herein represented as a projection). All twenty-five single-item, AMS-dated radiocarbon samples are projected using their y- and z-axis coordinates (five non-plotted items are assigned to the centroid of their square/spit provenience). The colour codes reflect the significance attached to radiocarbon results in terms of accuracy and site formation process (MAMS-33905 and MAMS-41871 are not coded because their interpretation remains open). The lab numbers are associated with the 95.4% probability intervals of the samples’ calendar ages. The original field record was hand-drawn at 1:10. Elevations are in cm below datum.

The description above reveals a few culture-stratigraphic anomalies in the Back Chamber sequence, mostly either side of its two major discontinuities (Eb/Fa and K/L). Correlative age-depth anomalies at those elevations are also apparent in the radiocarbon dating record (Tables 4 and 5; Figs 14 and 15): some results appear too young while others appear too old. Discrepancies between radiocarbon age and provenance can conceivably relate to issues of methodological reliability, décapage error, post-depositional disturbance, or interpretation of a sample’s archaeological significance, and will be dealt with in the Discussion section. In the following, we summarise the chronology derived from the samples that, as per the colour coding in Fig 15, are unproblematic.

**Table 4:** AMS radiocarbon dating of the Pleistocene deposit in back chamber, corridor, and entrance: Samples.

Culture	Layer	Sample #	x	y	z	Spit	Material	Taxon	Description	Result received	Reference (a)
Solutrean	Eb	O13sc91	–	–	–	E4	tooth	*Equus* sp.	lower molar	23/02/2021	This work (b)
	Fa	P11-241	20	50	577		bone	*Cervus elaphus*	metatarsal, distal fragment	22/08/1990	Zilhão (1997)
	Fb	O12-57	8	68	580	F3	bone	*Homo sapiens*	left metacarpal 4	16/11/2018	This work
		P12-251	65	68	586	F11	bone	*Homo sapiens*	manual middle phalanx 2–4	16/11/2018	This work
	Fc	P12-401	70	68	607		bone	*Cervus elaphus*	left metatarsal, proximal	22/08/1990	Zilhão (1997)
	H	O12-84	14	80	632	H1 burrow	bone	*Homo sapiens*	left mandible with dm2	16/11/2018	This work (c)
		P12-537	50	94	641		bone	*Capra pyrenaica*	naviculo-cuboid	22/08/1990	Zilhão (1997)
		P12-521	63	99	635		bone	–	rib, proximal fragment	22/08/1990	Zilhão (1997)
	I	P12-574	85	100	660		bone	*Cervus elaphus*	first phalange	22/08/1990	Zilhão (1997)
	Ja	P11-736	91	81	721	North profile	tooth	*Equus* sp.	lower molar	23/02/2021	This work
EUP	Jb	P13-403	57	33	648	J6	bone	*Cervus elaphus*	distal metapodial	20/06/1995	Zilhão (1997) (d)
		P13sc491	–	–	–	J4	shell	*Littorina obtusata*	broken shell bead	04/05/2010	This work
		P13-402	63	38	649	J6	shell	*Aporrhais pespelecani*	shell bead	04/05/2010	This work
		P11-701	5	52	738	profile	bone	–	metapodial fragment	20/06/1995	Zilhão (1997)
	K	P12-705	72	2	697	K5	burnt bone	–	shaft fragment	16/11/2000	This work
		P13-491	32	84	703	K5	bone	*Capra pyrenaica*	scapula fragment	20/06/1995	Zilhão (1997)
										27/02/2010	This work
		P11-799	3	29	748	K3	bone	*Cervus elaphus*	unfused first phalange	22/08/1990	Zilhão (1997)
		P11-783	93	58	757	K3	tooth	*Equus* sp.	molar (M1 or M2)	06/12/2011	This work
		P11sc968	–	–	–	K1East	shell	*Semicassis saburon*	broken shell bead	04/05/2010	This work
Mousterian	L	P11sc894-1	–	–	–	L1	bone	–	shaft fragment	05/10/2019	This work
		P11sc904-1	–	–	–	L3	bone	–	shaft fragment	05/10/2019	This work
	M	P11sc909-8	–	–	–	M1	bone	–	shaft fragment	08/02/2018	This work

Provenience, description and referencing

(a) for fac-simile reproduction of the original field documents and associated stratigraphic reasoning concerning samples O13sc91, O12-84 and P13-403, see S1I-1K Fig in S1 File

(b) though retrieved in an apparently undisturbed area of Magdalenian layer Eb, the sample lay adjacent to a large burrow feature and must be reworked from the underlying Solutrean deposit

(c) found in a small burrow adjacent to the cave wall, the sample likely belongs in H, but downward migration from immediately overlying layer Fc cannot be excluded

(d) originally published by mistake as coming from K, but in fact found at the base of Jb, at the same elevation and within a few cm of the P13-401 naturally backed, continuously retouched blade (Fig 10, no. 1) and the P13-402 *Aporrhais pespelecani* shell (Fig 12, no. 1).

**Table 5:** AMS radiocarbon dating of the pleistocene deposit in back chamber and corridor: Results.

Sample #	Lab # (a)	Sample mass (mg)	Collagen mass (mg)	% collagen	δ^13^C	δ^15^N	%C	%N	C:N	^14^C Age (BP)	cal BP (b)	cal BP (c)	Notes
O13sc91	MAMS-38336	195	6	3.1	-21.2	1.9	45.8	15.6	3.43	20077 ± 100	23930–24192	23824–24325	(d,e)
P11-241	OxA-1938 (AC)	–	19.9	–	–	–	20.1	–	–	20400 ± 270	24207–24927	23874–25178	
O12-57	OxA-37729 (AG)	112	11.9	10.6	-19.74	7.84	–	–	–	6374 ± 34	5310–5459	5224–5469	
P12-251	OxA-37728 (AG)	120	4.3	3.6	-20.25	8.21	–	–	–	6350 ± 34	5231–5365	5219–5464	
P12-401	OxA-2510 (AI)	1200	7.3	0.6	-21.8	–	33.3	–	–	18840 ± 200	22543–22941	22360–23111	
O12-84	OxA-X-2786-13 (AG)	250	2.6	0.7	-18.2	–	–	–	3.5	19400 ± 150	23135–23721	23036–23759	
P12-537	OxA-1939 (AC)	920	13.6	1.5	–	–	22.1	–	–	19900 ± 260	23441–24261	23234–24603	
P12-521	OxA-2511 (AI)	960	8.0	0.8	-21.1	–	30.0	–	–	20530 ± 270	24317–25039	23922–25331	
P12-574	OxA-1940 (AC)	1340	19.9	1.5		–	20.1	–	–	22900 ± 380	26544–27650	26379–27789	
P11-736	MAMS-38337	339	3.7	1.1	-20.4	7.1	44.4	15.3	3.4	23437 ± 140	27474–27757	27343–27816	(d,e)
P13-403	OxA-5541 (AI)	2000	4.2	0.2	-20.1	–	38.1	–	–	18060 ± 140	–	–	(f)
P13sc491	OxA-22299 (OX)	–	–	–	1.9	–	–	–	–	25560 ± 100	28787–29052	28659–29160	
P13-402	OxA-22300 (OX)	–	–	–	1.1	–	–	–	–	25750 ± 110	28937–29228	28786–29447	
P11-701	OxA-5542 (AI)	1220	24.6	2.0	-19.6	–	38.9	–	–	26020 ± 320	30020–30733	29696–31038	
P12-705	OxA-8670 (RR)	–	–	–	-21.4	–	–	–	–	25220 ± 200	–	–	(f)
P13-491	OxA-5521 (AI)		4.8	0.8	-21.1	–	37.5	–	–	23040 ± 340	–	–	(f)
	OxA-22020 (AF)	–	10.7	1.0	-21.8	–	41.4	–	3.1	26790 ± 260	30792–31179	30346–31272	(e)
P11-799	OxA-1941 (AC)	1460	21.7	1.5	–	–	20.7	–	–	27600 ± 600	31068–32741	30446–33311	
P11-783	VERA-5454	–	–	0.3	-17.0 ± 1.3	–	–	–	–	28000 ± 210	31611–32212	31481–32893	(d,g,h)
	VERA-5454_UF2	–	–	0.3	-22.4 ± 0.6	–	–	–	–	27130 ± 200	–	–	(d,e,g)
	VERA-5454_2	–	–	0.6	-20.6 ± 1.5	–	–	–	–	28390 +250/-240	32107–32964	31787–33282	(d,g,h)
	VERA-5454_2UF1	–	–	0.2	-22.6 ± 0.9	–	–	–	–	27470 ± 230	–	–	(d,e,g)
	VERA-5454_2UF2	–	–	0.3	-23.9 ± 0.9	–	–	–	–	27620 ± 250	–	–	(d,e,g)
P11sc968	OxA-22301 (OX)	–	–	–	1.1	–	–	–	–	37500 ± 230	41087–41469	40916–41682	(i)
P11sc894-1	MAMS-41871	593	8.6	1.5	-20.2	6.4	42.7	15.5	3.2	36490 ± 390	41192–41793	40886–42028	(e)
P11sc904-1	MAMS-41872	589	8.6	1.5	-20.7	4.6	43.7	15.5	3.3	28150 ± 160	31791–32774	31721–32903	(e)
P11sc909-8	MAMS-33905	504	3.8	0.8	-18.5	7.9	29.4	11.1	3.1	31900 ± 170	36085–36402	35844–36669	(e)

Age measurements. Calibration was carried out with Calib 8.1 for Windows using the IntCal20 Northern Hemisphere curve for the bone samples and the Marine20 curve for the shell samples [41–43]

(a) OxA results given with pre-treatment code (see Materials and Methods)

(b) 68.3% confidence interval

(c) 95.4% confidence interval

(d) enamel and dentine mix

(e) ultrafiltration pre-treatment

(f) incomplete decontamination, minimum age only (see text)

(g) δ^13^C values were determined with the AMS system, and VERA-5454, VERA-5454UF2, VERA-5454_2, and VERA-5454_2UF1/UF2 are sub-samples of the same specimen, where UF1 is the >30kDa fraction and UF2 is <30kDa fraction

(h) possibly minimum ages only

(i) subfossil specimen.

The results obtained for layers Fc and H are age-depth consistent. OxA-2510 (18,840 ± 200 BP) places layer Fc in the 22.4–23.1 ka interval. OxA-1939 (19,900 ± 260 BP) and OxA-2511 (20,530 ± 270 BP) are statistically indistinguishable and place layer H in the 23.2–25.3 ka interval. OxA-X-2786-13 (19,400 ± 150 BP; 23.0–23.8 ka) is of intermediate age, statistically distinct from OxA-2510 for Fc but indistinguishable from OxA-1939 for H. OxA-X-2786-13 is from human mandibular fragment O12-84 (Fig 14A) and was retrieved alongside a concentration of rabbit bones within a small pocket against the cave wall. This pocket appeared when the Fc/H interface was exposed. Given this provenance, downward migration of O12-84 from immediately overlying layer Fc cannot be excluded. However, the evidence from the field records (S1J Fig in S1 File) pleads in favour of the sample not being intrusive and belonging indeed in the layer H find horizon; given the low collagen yield, it cannot be excluded that the measured age is somewhat of an underestimation, as per its OxA-X label.

The ages obtained for layer H provide a *terminus ante quem* for layers I and Ja that is consistent with the composition of their stone tool assemblages. For Protosolutrean layer Ja, one would expect a result younger than the *terminus post quem* set by the age of the preceding phase of the Portuguese Upper Palaeolithic, the Terminal Gravettian. This latter phase has been AMS-dated using charcoal samples from high-resolution sequences to the 25.0–26.0 ka interval (e.g., the Lagar Velho rock-shelter OxA-8420 result of 21,180 ± 240 BP [23]). Yet, set against this expectation, the OxA-1940 and MAMS-38337 results for, respectively, layers I (22,900 ± 380 BP; 26.4–27.8 ka) and Ja (23,437 ± 140 BP; 27.3–27.8 ka) are too old, by one to two millennia. For layer Ja, the result strongly suggests that we are dealing with a palimpsest situation akin to that identified at Lapa do Anecrial, a cave located *c*. 30 km to the West (Fig 1, no. 3) [44]. This suggestion will be explored in the Discussion section below, alongside possible explanations for the layer I date.

After calibration, the two shell ornaments from layer Jb are slightly younger than the animal bone sample from the same unit. Prior to calibration, however, all three results overlap. This outcome may be due to the reservoir effect off the Portuguese coast being less important at that time than the average oceanic value used for calibration with the Marine20 curve (which, for a raw radiocarbon result of 26,000 BP, implies correction to an age *c*. 900 years younger than if the IntCal20 curve were used instead) [42]. Alternatively, layer Jb could be chronologically heterogeneous and represent at least two separate episodes of sediment accumulation, as indeed suggested by the analysis of extant profiles (Fig 5). The amount of time comprised between the extremes of the combined age interval—28.7–31.0 ka, i.e., less than two-and-a-half millennia—falls in the range of the Middle Gravettian. At the Lagar Velho rock-shelter, the living floors exposed at the elevation of the well-known child burial that have been dated to the same period show a near exclusive recourse to the expedient knapping of locally available quartzite cobbles [23, 24]. Broadly coeval with Caldeirão layers Jb and K, these logistical occupations of Lagar Velho yielded no culture-stratigraphically diagnostic lithics either; the associated radiocarbon results are about a millennium younger than Caldeirão’s, but they are on burnt bone and therefore likely to underestimate the true age of the occupations [45].

OxA-1941 (Fig 14C) and OxA-22020 are statistically indistinguishable and place layer K within the 30.4–33.3 ka interval that, elsewhere in south-western Europe, corresponds to the Early Gravettian. VERA-5454 and VERA-5454_2 are two measurements made on different sub-samples of the same equid tooth (Fig 14B); the results are statistically indistinguishable and overlap with OxA-1941. However, both of the VERA results were obtained with a modified Longin collagen extraction method, and their assessment for accuracy by using ultrafiltration for sub-samples of the tooth was inconclusive. The higher molecular weight fraction of VERA-5454 (UF1) failed, and the lower molecular weight fraction (VERA-5454_UF2), where one would expect to find any unremoved contaminating material, yielded a statistically younger result. In a second trial, both ultrafiltrated fractions, VERA-5454_2UF1 and VERA-5454_2UF2, could be successfully dated; the results obtained are identical but statistically younger than the result obtained with the standard treatment. Given the low collagen yields, one possible explanation for the younger ages of the ultrafiltrated samples is that the applied blank corrections are underestimations. In these circumstances, the accuracy of the non-ultrafiltrated measurements cannot be guaranteed. VERA-5454 will nonetheless be retained as valid because the Longin-based results for this equid tooth are mutually consistent, are not younger than the corresponding ultrafiltration results, agree with the OxA ages, and the yield of VERA-5454_2 (0.6%) was acceptable for a mixed dentine/enamel tooth sample.

The interpretation of the radiocarbon results available for layers L and M is complex. More results might have helped to clarify the issue, but the additional bone samples from the Middle Palaeolithic of the Back Chamber submitted for dating—another four from layer L, another ten from layer M, and another five from layer N—all failed to yield enough collagen. MAMS-41872 is indistinguishable from the results for overlying layer K and, clearly, reflects either décapage error or the intrusive nature of the dated sample. The other two results (MAMS-33905 and MAMS-41871) are in reverse stratigraphic order; under the assumption that both are accurate measurements, one must have undergone post-depositional displacement. We will come back to this issue at length in the Discussion section below.

#### 2.1.4. Luminescence dating

The final single-grain OSL ages obtained for the Caldeirão samples are shown in Table 6. The lowermost sample collected from layer O (Sample CLD17-5) yields an OSL age of 58.4 ± 3.8 ka, providing finite constraints on some of the oldest excavated deposits in the chamber. OSL ages of 42.0 ± 2.5 ka to 37.9 ± 2.3 ka are obtained for the overlaying layers N (CLD17-4), M (CLD17-3) and L (CLD17-2). The weighted mean OSL ages of layers M and L appear slightly inverted (37.9 ± 2.3 ka and 39.6 ± 2.3 ka, respectively); however, these two ages are statistically indistinguishable at 1σ. The layer K sample (CLD17-1) produces a single-grain OSL age of 37.7 ± 2.8 ka, providing a bracketing (*terminus ante quem*) constraint on the culturally significant K/L boundary.

**Table 6:** Single-grain OSL dating results.

Sample	Layer	Grain size (μm)	Water content (a)	Gamma dose rate (b)	Beta dose rate (c)	Cosmic dose rate (d)	Internal dose rate (e)	Total dose rate (Gy/ka) (f)	Number of grains (g)	Age model (h)	D_e_ (Gy) (f)	Age (ka) (f,i)
CLD17-1	K	212–250	9.9	0.85 ± 0.03	1.29 ± 0.07	0.05 ± 0.01	0.03 ± 0.01	2.23 ± 0.11	100/900	MAM-3	84.1 ± 4.2	37.7 ± 2.8
CLD17-2	L	212–250	10.7	1.19 ± 0.04	1.72 ± 0.09	0.05 ± 0.01	0.03 ± 0.01	2.99 ± 0.15	180/1100	CAM	118.4 ± 2.7	39.6 ± 2.3
CLD17-3	M	212–250	10.6	1.30 ± 0.05	1.69 ± 0.08	0.05 ± 0.01	0.03 ± 0.01	3.08 ± 0.15	127/900	CAM	116.6 ± 3.4	37.9 ± 2.3
CLD17-4	N	212–250	11.6	1.20 ± 0.05	1.74 ± 0.09	0.05 ± 0.01	0.03 ± 0.01	3.02 ± 0.15	108/800	CAM	126.8 ± 3.5	42.0 ± 2.5
CLD17-5	O	212–250	12.3	1.19 ± 0.05	1.52 ± 0.08	0.05 ± 0.01	0.03 ± 0.01	2.79 ± 0.14	129/900	CAM	162.9 ± 5.7	58.4 ± 3.8

Dose rate estimates, D_e_ summary statistics, age model results, and corresponding single-grain OSL ages (1σ error) for the samples collected from the Back Chamber

(a) long-term water content expressed as % of dry mass of mineral fraction and assigned relative uncertainties of ± 20%

(b) gamma dose rate calculated from *in situ* field gamma spectrometry measurements made at each sample position with a NaI:Tl detector using the “energy windows” method detailed in [46, 47]

(c) beta dose rates calculated using a Risø GM-25-5 low-level beta counter [48], after making allowance for beta dose attenuation due to grain-size effects and HF etching [49, 50]

(d) cosmic-ray dose rates calculated using the approach of [51] and assigned a relative uncertainty of ± 10%.

(e) the assumed internal alpha + beta dose rate for quartz, with an assigned relative uncertainty of ± 30%, based on the intrinsic ^238^U and ^232^Th contents published by [52–56], and an a-value of 0.04 ± 0.01 [57, 58]

(f) mean ± total uncertainty (68% confidence interval), calculated as the quadratic sum of the random and systematic uncertainties

(g) number of D_e_ measurements that passed the SAR rejection criteria and were used for D_e_ determination / total number of D_e_ values analysed

(h) age model used to calculate the sample-averaged D_e_ value for each sample (CAM = central age model; MAM-3 = three-parameter minimum age model); the MAM-3 D_e_ estimates have been calculated after adding, in quadrature, a relative error of 20% to each individual D_e_ measurement error to approximate the underlying dose overdispersion observed in “ideal” (well-bleached and unmixed) sedimentary samples from this site (i.e., samples CLD17-2 to CLD17-4; see S1D Table in S1 File), and from global overdispersion datasets [59]

(i) total uncertainty includes a systematic component of ± 2% associated with laboratory beta-source calibration.

**Fig 16:**
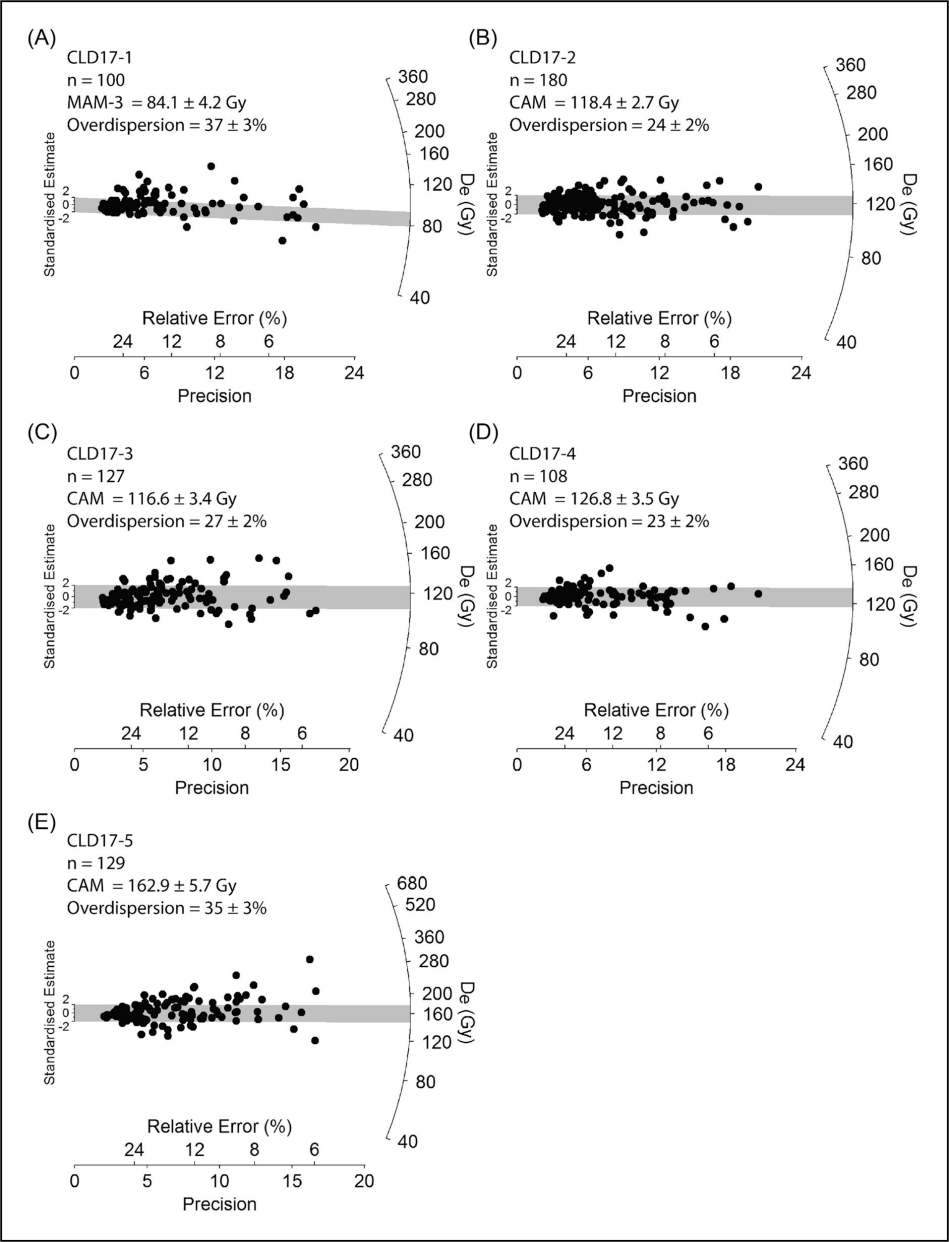
Radial plots showing the OSL samples’ single-grain D_e_ distributions. The grey bar is centred on the age model that provides the optimum statistical fit for each distribution (bold values in S1D Table in S1 File).

The samples collected from layers L, M, and N (CLD17-2, CLD17-3, and CLD17-4) exhibit the most homogeneous single-grain D_e_ (equivalent dose) distributions (Fig 16B–16D). These D_e_ datasets are characterised by low-to-moderate dose dispersion, D_e_ scatter that is reasonably well-represented by the weighted mean value (as indicated by the large proportions of grains lying within the 2σ grey bands), and low overdispersion values of 23 ± 2% to 27 ± 2% (S1D Table in S1 File). These overdispersion values are similar to those typically obtained for ideal (well-bleached and unmixed) single-grain D_e_ datasets (cf. the global average overdispersion value of 20 ± 1% reported in [60]). Sample CLD17-5 from layer O exhibits similar D_e_ distribution characteristics (Fig 16E), though it has a moderate overdispersion value of 35 ± 3% (S1D Table in S1 File). None of these four D_e_ datasets are considered significantly positively skewed according to the weighted skewness test outlined in [61, 62] (S1D Table in S1 File). Application of the maximum log likelihood (*L*_*max*_) test [60] indicates for all four D_e_ datasets that the CAM (central age model) is statistically favoured over the three- or four-parameter MAM (minimum age model) (MAM-3 or MAM-4) [63]. Collectively, these single-grain OSL D_e_ characteristics suggest that the samples from layers L, M, and N do not suffer from major extrinsic D_e_ scatter related to insufficient bleaching prior to burial (e.g., [64, 65]) or to widespread post-depositional sediment mixing between units (e.g., [65]). It is therefore likely that the low to moderate overdispersion observed for these samples is attributable to intrinsic experimental scatter not captured by the dose recovery test—e.g., grain-to-grain variations in luminescence responses due to the fixed SAR- (single-aliquot regenerative) dose conditions [66]; or to extrinsic field-related scatter associated with beta-dose spatial heterogeneity (e.g., [67]). Consequently, the single-grain OSL ages for CLD17-2 to CLD17-5 were obtained using the weighted mean (CAM) D_e_ estimate, in accordance with their *L*_*max*_ test results, as outlined in [60].

Sample CLD17-1 (layer K) exhibits a more complex D_e_ distribution, characterised by moderate dose dispersion, a larger proportion of individual D_e_ values lying outside of the weighted mean (CAM) burial dose 2σ range, and a more prominent leading-edge of low D_e_ values (Fig 16A). Unlike the site’s other OSL samples, CLD17-1 is considered to be significantly positively skewed when compared with its 2σ critical skewness score ([61, 62]). The overdispersion value for this sample (37 ± 3%; S1D Table in S1 File) does not overlap with the “best case scenario” (baseline) estimates of overdispersion for well-bleached and unmixed samples from this site (i.e., 19–31% based on the 2σ overdispersion ranges of samples CLD17-2, CLD17-3, and CLD17-4), though it is similar to that of CLD17-5 (35 ± 3%). Application of the *L*_*max*_ test [60] indicates that the MAM-3 age is statistically favoured over the CAM age for this D_e_ dataset (S1D Table in S1 File). These various D_e_ characteristics suggest that dose dispersion originating from field-related sources has exerted a more significant influence on CLD17-1. We interpret the more heterogeneous D_e_ distribution characteristics of this sample as primarily reflecting a locally complex depositional history for layer K; namely, the incorporation of grains from pre-existing deposits within the Back Chamber, without them seeing much, if any, external light (an issue we come back to in the Discussion section below). Such complex syn-depositional mixing processes have the potential to give rise to single-grain D_e_ distributions that are outwardly similar to those commonly reported for heterogeneously bleached single-grain OSL samples (e.g.,[64, 68]), as has been reported for several analogous cave infill sequences (e.g., [69–71]). The final age of sample CLD17-1 has therefore been calculated using the MAM-3, in accordance with sedimentary considerations (see the Discussion below) and the *L*_*max*_ test results.

### 2.2. Entrance

**Fig 17:**
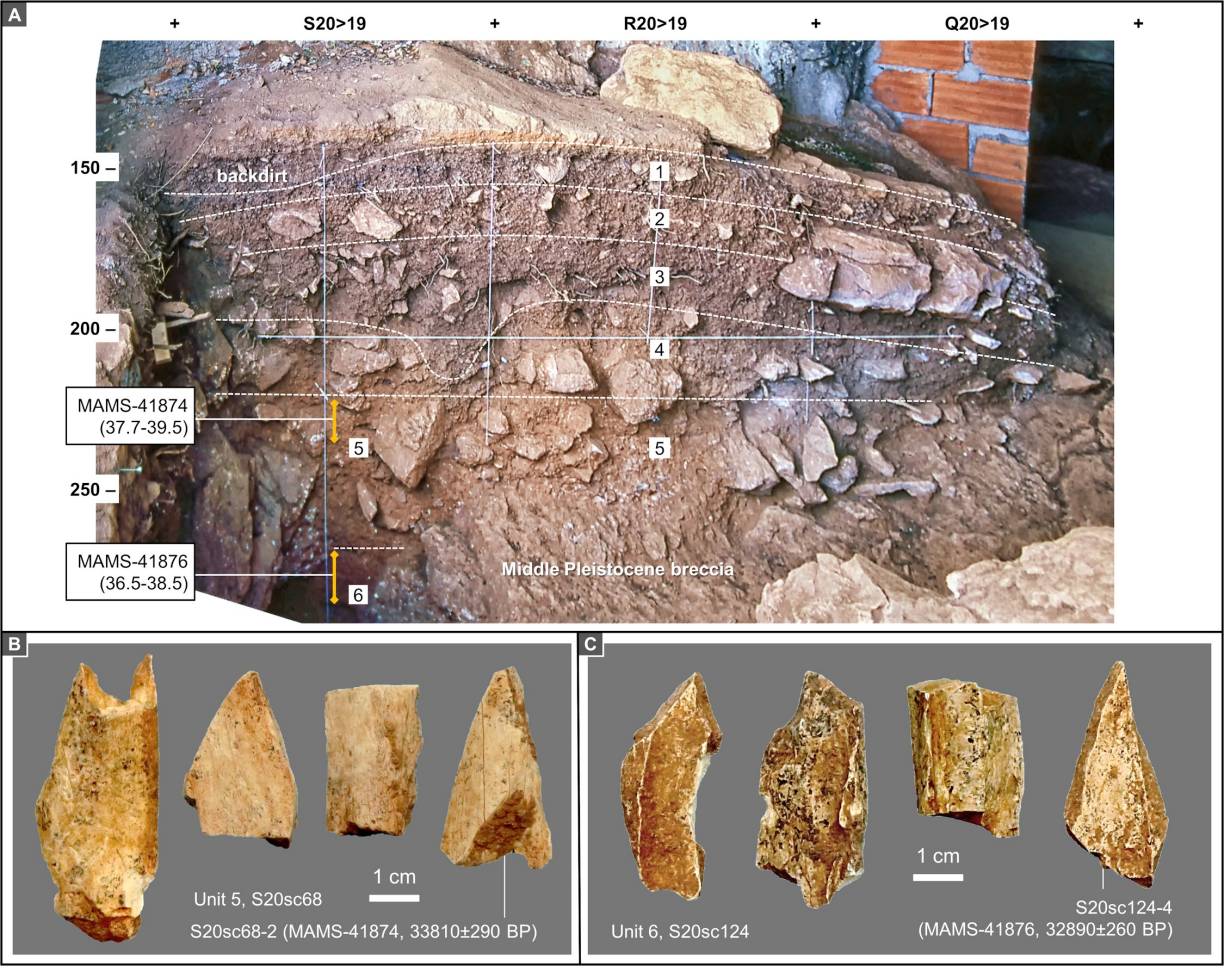
Entrance trench: Stratigraphy and dating. **A.** Orthorectified photo of the S-Q20>19 profile, and stratigraphic interpretation (the dark pocket reaching the base of unit 4 is a disturbance feature); the 95.4% probability intervals of the calendar ages (ka BP) for units 5 and 6 in Tables 7 and 8 are indicated; elevations are in cm below datum. **B, C.** Non-plotted shaft fragments from spits F10 of unit 5 (**B**) and F16 of unit 6 (**C**); the specimens that yielded enough collagen are associated with age result plus inventory and lab numbers.

Fig 17A illustrates the stratigraphic profile extant in 1988, the last field season. The samples that were successfully radiocarbon dated are illustrated in Fig 17B and 17C, and their composition, provenance, and analytical data are reported in Tables 7 and 8.

**Table 7:** AMS radiocarbon dating in the entrance trench: samples.

Culture	Layer	Sample #	x	y	z	Spit	Material	Taxon	Description	Result received	Reference
Mousterian	Unit 5	S20sc68-2	–	–	–	F10	bone	–	shaft fragment	05/10/2019	This work
	Unit 6	S20sc124-4	–	–	–	F16	bone	–	shaft fragment	05/10/2019	This work

Provenience, description and referencing.

**Table 8:** AMS radiocarbon dating in the entrance trench: Results.

Sample #	Lab #	Sample mass (mg)	Collagen mass (mg)	% collagen	δ^13^C	δ^15^N	%C	%N	C:N	^14^C Age (BP)	cal BP (a)	cal BP (b)	Notes
S20sc68-2	MAMS-41874	631	6.6	1.0	-20.5	3.8	43.3	15.5	3.3	33810 ± 290	38357–39345	37665–39500	(c)
S20sc124-4	MAMS-41876	664	5.5	0.8	-20.1	4.9	43.4	15.3	3.3	32890 ± 260	36880–37666	36536–38465	(c)

Age measurements. Calibration was carried out with Calib 8.1 for Windows using the IntCal20 Northern Hemisphere curve [41, 43]

(a) ultrafiltration pre-treatment

(b) 68.3% confidence interval

(c) 95.4% confidence interval.

The Entrance trench was excavated in two steps. Squares P-Q/20-21 were taken down to bedrock in 1983–84. Squares R-S/20 were excavated in 1986–87. The basal Middle Pleistocene breccia was reached in R20 but not in S20, where the excavation had to stop at 305 cm below datum because, starting *c*. 1 m below the surface, the excavatable area became ever more restricted between (a) an outward-sloping step of the breccia, (b) the truncated cave wall, and (c) a large, protruding flowstone mass occupying most of the S>T20 profile (Figs 2C, 2D, 3, and 17A).

The limited thickness of the deposit explains the lower resolution of the succession, especially in its upper 50 cm, which is affected by the development of a Holocene soil. Unit 1 is a dry, dark brown, loose, silty sand (often aggregating as rootlet-supported crumbs) and yielded modern potsherds. Unit 2 is a finer and more compact, lighter reddish-brown earth with abundant *Helix* remains. Unit 3 has a similar matrix but is richer in limestone éboulis that, as in unit 4, probably reflect the gradual recession of the cliff face. Units 4 and 5 have a silty sand matrix of distinctively orange-red colour (in the case of unit 4, paler, and, inwardly, whitish, or light brown) but are separated by a significant discontinuity. In square R20, their boundary appeared as an extensively cemented surface found under a lens of fine gravel and small pebbles. This surface could be followed into S20 where, however, the underlying deposit (i.e., unit 5) was less compact. At both ends of the profile (in S20, against the southern wall of the trench; in Q20, against the outcropping bedrock), the contact between units 4 and 5 appears somewhat disturbed by wall effects and the presence of roots. The lower boundary to unit 6—a darker red, clayey deposit—was marked by a horizon of large, 20–30 cm éboulis lying atop the interface.

**Fig 18:**
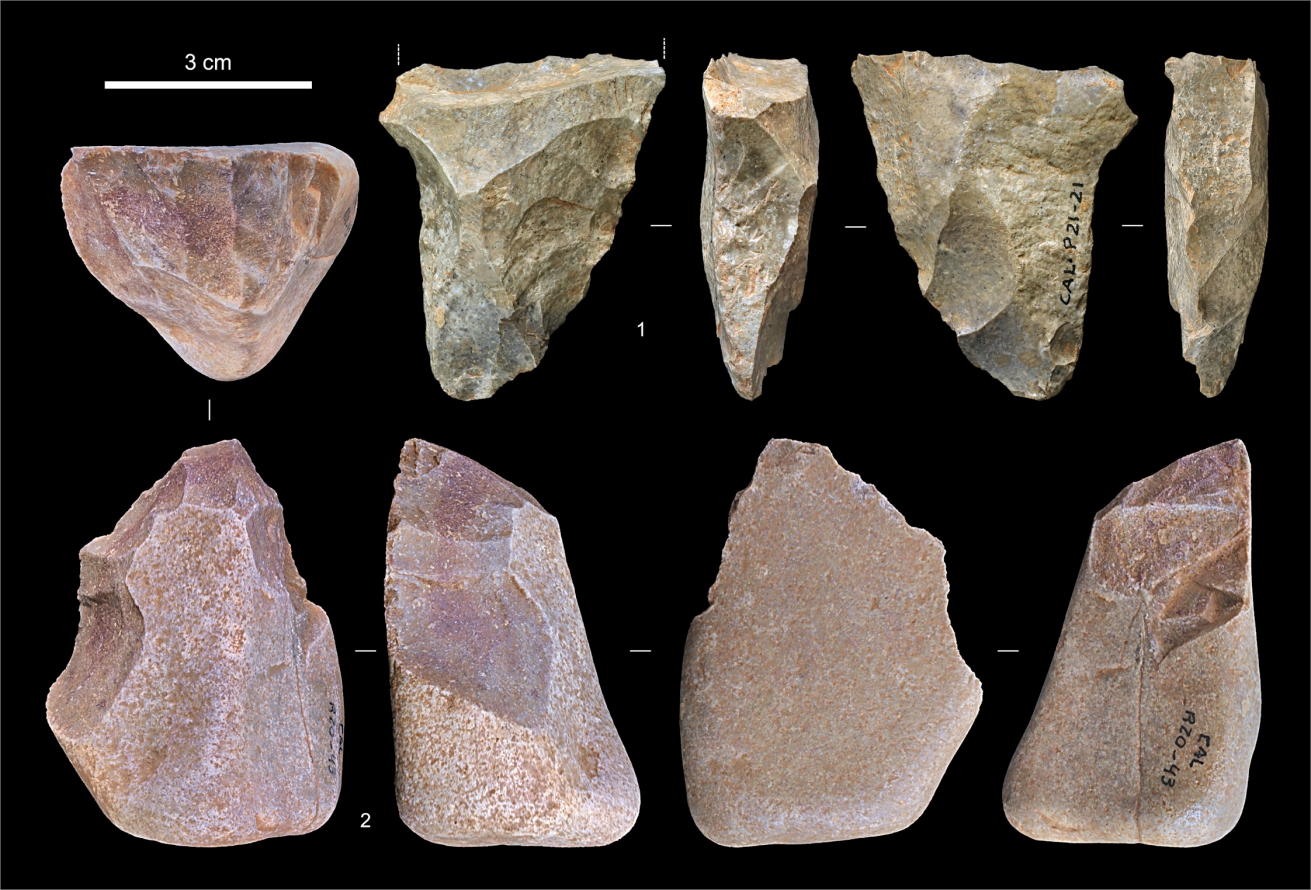
Entrance trench: Upper Palaeolithic artefacts. **1.** Bifacial preform fragment, chert (P21-21, Unit 2). **2.** Carinated scraper/core, quartzite (R20-43, Unit 4).

**Fig 19:**
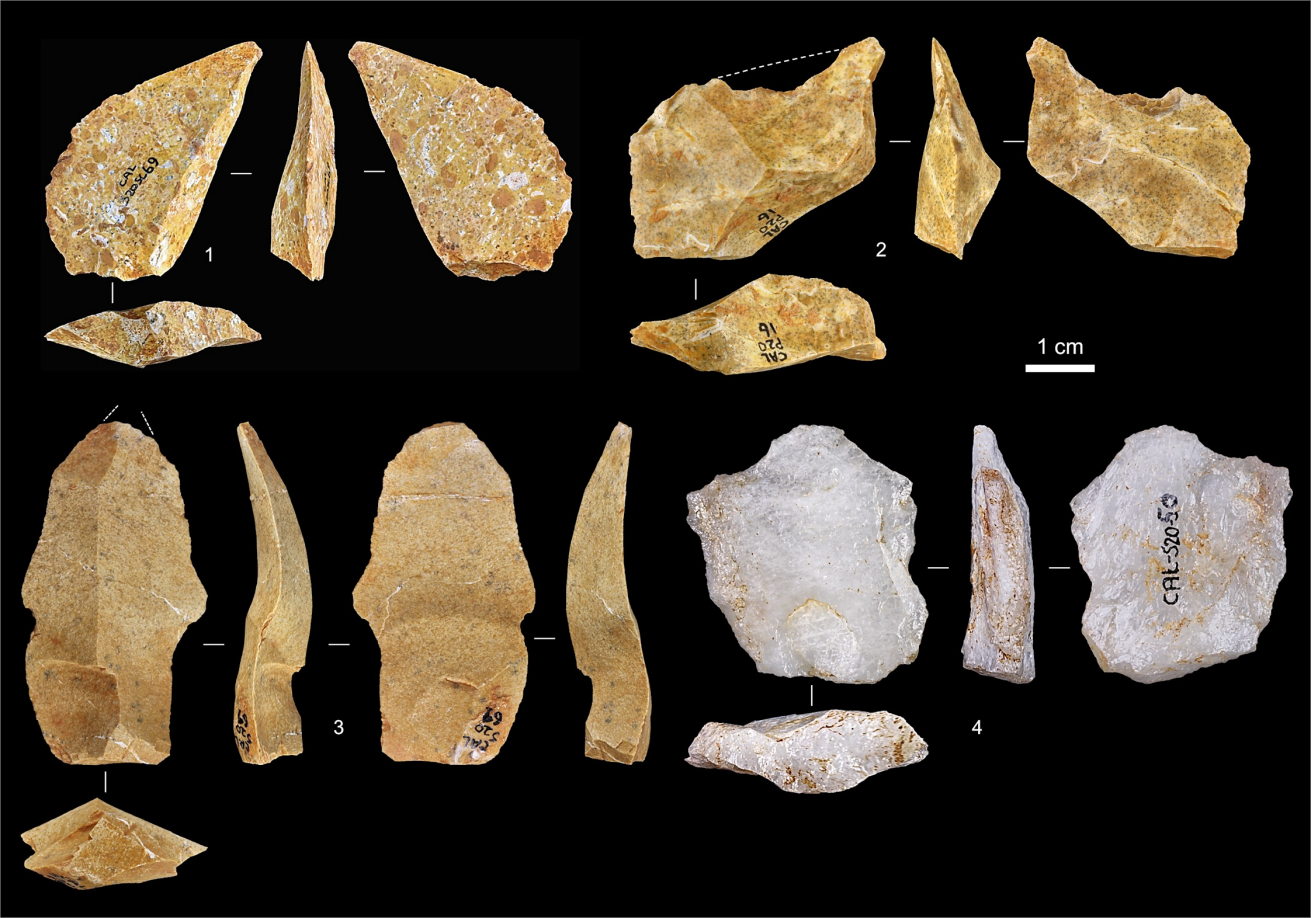
Entrance trench: Middle Palaeolithic debitage. **1.** Kombewa flake, silcrete (S20sc69). **2.** Pseudo-Levallois point, silcrete (P20-16; excavation-damaged; the dashed line indicates the outline of the item’s original edge). **3.** Laminar flake, silcrete (S20-61). **4.** Levallois flake, quartz (S20-50). Nos. 1–2 are from Unit 5, nos. 3–4 from Unit 6.

**Fig 20:**
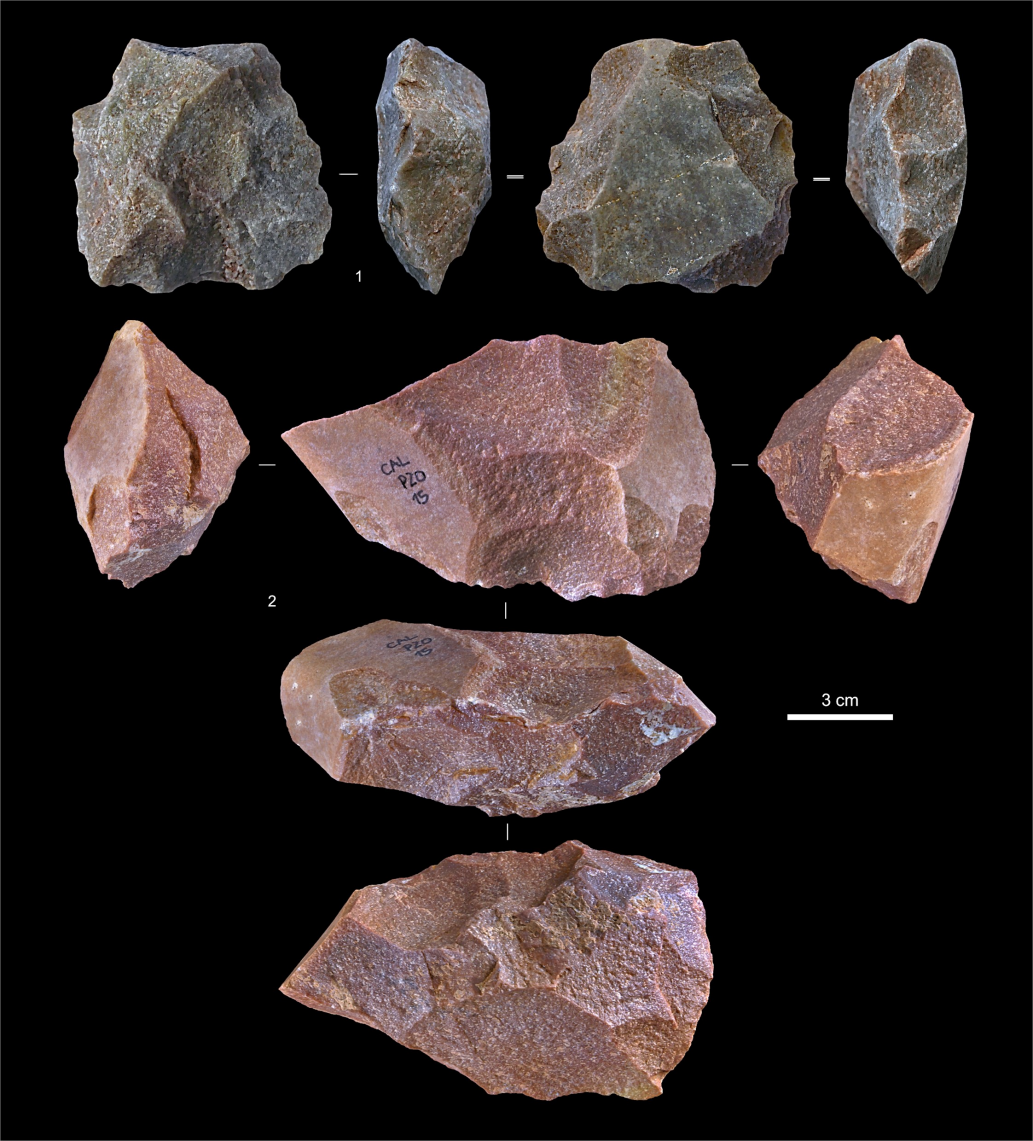
Entrance trench: Middle palaeolithic quartzite cores. **1.** Centripetal Levallois core, exhausted (Unit 6, S20sc130). **2.** Discoidal core, initial phase (set-up of the reduction surface) (Unit 5, P20-15).

Below unit 1, the undisturbed sediment yielded no items characteristic of the periods of Holocene archaeology represented in the Back Chamber and the Corridor (e.g., ceramics, ornaments, stone or metal tools, glass). The herbivore taxa are the same as those making up the vast majority of the faunal assemblage from the Pleistocene succession of the cave’s interior: ibex, red deer, and horse. The stone tool assemblages are small and dominated by the expedient reduction of quartzite cobbles (Table 9). However, they contain a few technologically diagnostic items that enable correlation with the interior and a measure of culture-stratigraphic assignment: unit 2 yielded a bifacial preform fragment (Fig 18, no. 1), and a small bifacial thinning flake was found in unit 3 against the wall (in Q21); unit 4 yielded a characteristically Aurignacian carinated scraper/core made on a small quartzite cobble (Fig 18, no. 2); unit 5 yielded a Kombewa flake, a pseudo-Levallois point, and a quartzite core discarded in the initial stages of the configuration of two organised surfaces (Fig 19, nos. 1 and 2, and Fig 20, no. 2, respectively); unit 6 yielded a hard hammer-extracted laminar blank, a Levallois flake on quartz, and an exhausted, centripetal Levallois core on quartzite (Fig 19, nos. 3 and 4, and Fig 20, no. 1).

**Table 9:** Entrance lithics.

		Chert	Quartz	Quartzite	Hyaline Quartz	Other	Total
Unit 4	Core (a)	–	–	2	–	–	1
	Flake	2	1	–	–	–	3
	Flake fragment	–	1	1	–	–	2
	Chippage	–	–	1	–	–	1
	Chunk	–	–	1	–	–	1
	Manuport	–	–	1	–	–	1
	Total	2	2	6	–	–	10
Unit 5	Core	–	–	1	–	–	1
	Levallois core	–	–	1	–	–	1
	Bladelet	1	–	–	–	–	1
	Flake	4	2	1	–	–	7
	Pseudo-Levallois point	1	–	–	–	–	1
	Small flake	1	1	–	–	–	2
	Flake fragment	10	3	6	–	1	20
	Retouched tool (b)	1	–	–	–	–	1
	Chippage	1	5	3	–	–	9
	Chunk	–	1	1	–	–	2
	Total	19	12	13	–	1	45
Unit 6	Levallois core	–	1	1	–	–	2
	Flake	4	2	5	–	–	11
	Levallois flake	–	1	–	–	–	1
	Flake fragment	5	6	6	1	–	18
	Chippage	–	4	4	–	–	8
	Total	9	14	16	1	–	40

Inventory per techno-economic category

(a) carinated scraper/core

(b) flake with inverse marginal semi-abrupt retouch on left edge.

Based on this archaeological content, the following correlation with the cave’s interior can be proposed: unit 2 with layer Eb; unit 3 with layers Fa-Fc; unit 4 with the H-K sequence; units 5–6 with the L-N sequence. This correlation is supported by the presence of juvenile horse teeth and astragali corroded by digestive acids among the faunal remains found below unit 3. In the interior sequence, such finds occur alongside the remains of hyaena and, like these, are exclusive to the Early Upper and Middle Palaeolithic levels.

One batch of bone fragments from unit 4, three from unit 5, and another three from unit 6, each composed of four unidentified shaft fragments, were submitted for radiocarbon dating. Enough collagen was found in only two of these 28 samples. The following ages were obtained: 33,810 ± 290 BP (MAMS-41874; 37.7–39.5 ka) for unit 5, and 32,890 ± 260 BP (MAMS-41876; 36.5–38.5 ka) for unit 6 (Table 8). The uncalibrated radiocarbon results overlap, but minimally; their comparison yields a z-score of 2.36, i.e., they differ by more than twice the pooled standard deviations and so ought to be considered statistically distinct and represent a case of stratigraphic inversion. Yet, they are consistent with (a) correlation of the unit 4/unit 5 discontinuity with the K/L boundary, and (b) the notion that these two interfaces mark the point in time after which a Middle Palaeolithic technology no longer existed in the region.

## 3. Discussion

### 3.1. Chronology of the upper palaeolithic sequence

#### 3.1.1. Younger-than-expected ages

Two directly dated human remains from layer Fb turned out to be Neolithic intrusions related to funerary usage of the Back Chamber by people of the Cardial culture. However, they ought not to be counted as chrono-stratigraphic anomalies; they simply show that the excavation was not 100% effective in the identification of post-depositional disturbance at the Eb/Fa boundary. In the Back Chamber, the contrast between a fine, reddish, and massive Fa and a sandy, brownish, looser, and Cardial-intruded Eb, was in general rather stark, even though less noticeable in places, especially along the walls. With the benefit of hindsight, we can posit that the Fb1 cut-and-fill lens of Fig 5 is quite probably a manifestation of the so-called “wall effect” [72]. Such effects often entail the downward migration of small items, and it can be hardly coincidental that Neolithic intrusions into apparently intact areas of layers Fa and Fb were only found along the north wall of the cave in O12 and P12 (i.e., adjacent to the Fb1 feature).

Two other younger-than-expected results exist in the Fa-K sequence. OxA-8670 was obtained on a burnt bone sample and came with a lab warning that the result “should be treated as a minimum age only because the charred bone was of poor quality and the yield was very low.” OxA-5541 (18,060 ± 140 BP) dates P13-403, a deer metapodial fragment from the base of layer Jb (S1K Fig in S1 File). This sample was submitted alongside P13-491, a *Capra pyrenaica* left scapula from layer K dated by OxA-5521 to 23,040 ± 340 BP. As part of the same batch, both were pre-treated alike, i.e., with the standard, Longin-derived collagen extraction method used in the 1990s. Subsequent re-dating of P13-491 using ultrafiltration produced a result that is fully consistent with its stratigraphic position: 26,790 ± 260 BP (OxA-22020). Clearly, the younger-than-expected result for P13-491 obtained in the 1990s is a by-product of incomplete sample decontamination; the parsimonious explanation for P13-403 must therefore be the same, and even more so because its collagen yield was extremely low (0.2%; Table 5). As with OxA-8670 and OxA-5521, OxA-5541 cannot be considered a valid radiocarbon age by today’s standards.

#### 3.1.2. The *semicassis saburon* shell

The *Semicassis saburon* from uppermost layer K (Fig 13, no. 4) is ten millennia older than indicated by the layer’s other dated samples. Interpreting this result requires that we consider the lag between the dated event (death of the animal) and human collection (of the animal’s beached shell). Three hypotheses can be entertained: (a) the shell belongs in layer K’s Early Gravettian context (which implies a duration of minimally seven-and-a-half millennia for the lag); (b) the shell is part of a diffuse, hitherto stratigraphically non-differentiated, Evolved Aurignacian context (if the lag were in the range of five millennia); (c) the shell was upwardly displaced from Middle Palaeolithic layers L-N (if the lag can be deemed negligible).

It has been shown that bivalve thanatocoenoses in extant Mediterranean beaches contain shells that are several millennia-old—up to 4500 years for *Glycymeris* accumulations in Israeli beaches [73]. In the Atlantic coast of Portugal, however, the high wave energy militates against the possibility that beached shells could survive lengthy periods of exposure. Apparent ages of up to 3000 years were found in a study that used radiocarbon to date shells collected in the late 19th and early 20th centuries [74]; however, such extreme values were obtained for samples from a lagoon context (a *Cerithium vulgatum* and an oyster valve), and they can be explained to a significant extent by the large reservoir effect characteristic of inland and brackish waters. The apparent age of all the other historical specimens dated in that study was significantly lower, between 400 and 800 years. These latter values are in the range of the offset caused by the marine reservoir effect observed through the Holocene of Portugal via dating of associated pairs of bone/charcoal and shell samples from a number of archaeological contexts [74, 75]. The results of that marine reservoir study support the notion that Portugal’s active seashores are unlikely to yield shells that, at the time of collection, are already many centuries or even millennia old.

At first glance, the Holocene evidence would therefore seem to rule out that layer K’s *Semicassis saburon* ornament is an *in situ* find. Yet, we believe the opposite must be the case. This is because of the significant drop in sea level that occurred towards the end of MIS 3 and especially between 35,000 and 30,000 years ago: from *c*. 70 to *c*. 125 m below modern, i.e., about 11 m per millennium [76]. As a consequence, people living through that interval would have been able to procure marine shells for body ornamentation not just in then-extant beaches but also in the staircase of raised ones formed as the seashore receded. Such raised beaches have since been submerged by the sea level rise of the Holocene but, if the fact that they once existed is duly borne in mind, they provide a solution for the conundrum: the dated *Semicassis saburon* shell from layer K is a subfossil specimen collected not on the Early Gravettian seashore but on a raised beach deposit. Thus, despite *in situ* and, to the best of our knowledge, accurately dated, this sample and corresponding age measurement (OxA-22301) must be excluded from consideration when assessing the chronology of the succession.

#### 3.1.3. Solutrean and protosolutrean

The radiocarbon age obtained for layer Fa is older than that for layer Fc, and it falls in the same range as the radiocarbon ages obtained for samples from layer H taken almost 1 m lower down in the same P11 square. The radiocarbon age for layer I falls in the Late Gravettian range and thus is at odds with this layer’s stratigraphic position between Middle Solutrean layer H and Protosolutrean layer Ja. The latter is dated by a sample that yielded an even earlier age. How can these three observations be reconciled with the otherwise high degree of stratigraphic integrity displayed by the Back Chamber sequence?

Under the premise that the Fa sample (OxA-1938) is *in situ*, layers Fa-H would date to >23.9 ka. However, the stratigraphically consistent dating results for layers H and Fc show that they contain material that can be no older than 23.8 ka (e.g., the OxA-2510 and OxA-X-2786-13 samples). In addition, the rich, well-stratified, and precision-dated sequence of Lower, Middle and Upper Solutrean occupations of the Rambla Perea rock-shelter of La Boja (Murcia, Spain; Fig 1, no. 12) [16] shows that the transition from the Middle to the Upper Solutrean—represented at Caldeirão by layers H and Fc, respectively—occurs no earlier than *c*. 24.0 ka ago. The interpretation of the Fa sample as a reworked item brought up from layer H brings the dating of the sequence in line with regional chronostratigraphic patterns and is therefore the parsimonious view of the evidence (Fig 15).

**Fig 21:**
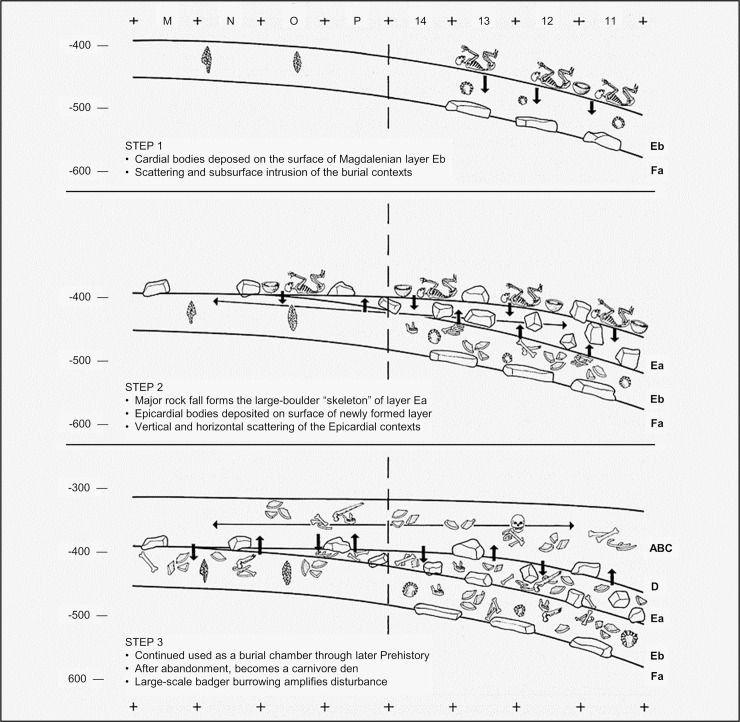
Site formation process in the holocene. Processes of sedimentary accumulation and post-depositional disturbance as inferred from stratigraphy, refitting, and spatial distributions (modified after Fig 3.11 of [1]).

**Fig 22:**
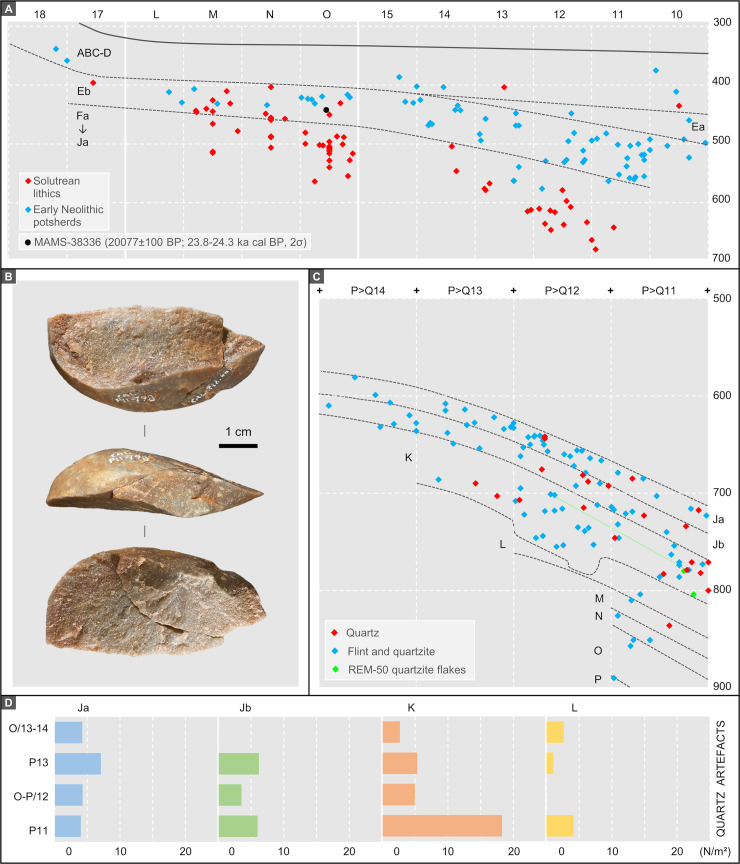
Back chamber artefact distributions illustrative of site formation process. **A.** y- over z-axis (columns 10–15 and 17–18) and x- over z-axis (rows L-O) projection of piece-plotted Early Neolithic potsherds (N = 72), Solutrean diagnostics (N = 70; includes 29 sieve items assigned to the centroid of the square/spit field provenience), and upwardly displaced Solutrean horse tooth O13sc91. **B.** Refit unit REM-50 (quartzite flakes from layer K, P11-798 and P12-665). **C.** y- over z-axis projection of piece-plotted lithics from layers Ja-P. **D.** Density of quartz artefacts (total number per area unit of excavation, manuports excluded) across layers Ja-L. Elevations are in cm below datum.

The contradiction between this reworking interpretation of OxA-1938 and the high level of integrity of the Fa-H sequence revealed by stratigraphic observations and the composition of stone tool assemblages is apparent rather than real. To understand why, we need to bear in mind the dynamics of sedimentation, erosion, and disturbance relative to the topography of the site and the geometry of the infill. To do that, it is useful to start by examining the situation either side of the Eb/Fa boundary (Figs 21 and 22A).

As described above and discussed at greater length in publications dealing with the Neolithic archaeology of Caldeirão [1, 6, 30], the Solutrean deposit of the Corridor features significant evidence of burrowing (Fig 4E). The disturbed areas were emptied prior to the excavation of the adjacent intact areas (except in the case of squares O/13-15, where they were emptied in parallel, but with finds and sieved sediment being processed and logged in separately). No pottery or other Neolithic proxies were found in such burrow features, but the 1980s conventional radiocarbon dating of a bulk sample of rabbit and unidentified herbivore shaft fragments from Corridor squares of layer Fa yielded a result of 15,170 ± 740 BP (ICEN-69) [2]. This younger-than-expected age must result from sample heterogeneity; specifically, the presence of Magdalenian-aged material. The fact that, due to size requirements, the sample was made up of bones from squares O13, O14, and N14 that mixed intact (one third) and disturbed (two thirds) provenances, is consistent with this view.

This first phase of the Corridor’s disturbance began toward the end of the accumulation of layer Fa and continued through the intervening sedimentation hiatus into the initial stages of the accumulation of layer Eb. Vertically, the attendant displacement of items was significant; horizontally, however, the scattering was limited. The dated deer metatarsal from layer Fa of the Back Chamber (OxA-1938) is among the few examples of horizontal displacement. Examples of vertical displacement are: (a) the Solutrean tools previously displaced from underlying layers Fa-Ja that layer Eb incorporated as it built-up; (b) the subsurface intrusion of Magdalenian bone as the hollows left by burrowing were filled-up and further reworked, exemplified by the ICEN-69 bulk radiocarbon age; and (c) the Solutrean age obtained for the O13sc91 horse tooth from layer Eb—20,077 ± 100 BP (MAMS-38336; 23.8–24.3 ka) (Tables 4 and 5; Fig 22A; S1I Fig in S1 File). In the course of a second phase of disturbance, during Late Neolithic and post-Neolithic times, when layer ABC-D was forming, limited amounts of potsherds and human bone originating in such later occupations penetrated subsurface into the upper part of layer Eb of the Corridor, mostly along the walls (Fig 21).

In the Back Chamber, potsherds, and other proxies for the Cardial Neolithic, but no Solutrean items, were found inside layer Eb (sometimes at the very bottom of the large burrows that penetrated deep into layers Fa-Fb; Figs 4D and 22A). These observations indicate that, in this part of the cave, the localised affection of Fa-Fb by non-human agent(s) of disturbance occurred during and immediately after the first phase of Early Neolithic funerary usage. As sedimentation resumed and the accumulation of layer ABC-D began, the subsurface activity of the burrowing agent (in all likelihood, badgers) became more conspicuous. The significant number of juvenile rabbit bones in layer ABC-D further shows that, in the recent Holocene, the cave harboured a warren. However, through the process, the Pleistocene sequence became ever more deeply buried, and the densely packed éboulis forming the skeleton of layer Ea provided additional protection against disturbance by large burrowers. That must be the reason why the upper part of layer Eb contained the odd sherd of post-Cardial pottery in the Corridor but not in the Back Chamber. Potsherd refitting does show, however, that, in later Neolithic and post-Neolithic times, significant back-and-forth displacement was happening across the length of the cave’s interior. It must have been in this way, i.e., via second-order reworking, that some Solutrean material derived from layer Eb of the Corridor eventually made its way into layer ABC-D of the Back Chamber (Figs 21 and 22A).

The above shows that, either side of a stratigraphic boundary involving a long hiatus in sedimentation and otherwise affected by cut-and-fill features, it is not unexpected that the odd item be found out of place significantly above its original locus of deposition, even when the intervening deposit is apparently intact. This will be the more so when the stratification presents a significant angle of dip and the thickness of each of its different units decreases upslope, as is the case with Caldeirão’s Pleistocene deposit. If the Corridor had not been excavated, i.e., based on the Back Chamber sequence alone, we would have lacked a proper understanding of site formation across the site as a whole, and we would be somewhat at a loss to explain how a few Solutrean items had managed to make their way into layer ABC-D whereas none existed in the up to 1.5 m-thick deposit separating the base of that layer from the Eb/Fa boundary. This happens because burrowing in the Corridor, even if only to a limited depth, sufficed to bring up material that is (a) significantly older and (b) liable to gravitate inward (i.e., downslope) and end up incorporated in apparently intact deposits of the Back Chamber found stratigraphically well above the layer of origin. Likewise, this process explains why the Back Chamber’s Solutrean sequence features the appearance, in the correct order, of the index fossils of its different phases despite the capping layer (Fa) containing one animal bone (OxA-1938) whose dating is at odds with expectations derived from stone tool typology.

Bearing in mind the Eb/Fa model, let us now turn to the layer I anomaly. Firstly, recall that the lithic assemblage in layer Ja not only clearly fits the Protosolutrean but, among the unretouched material, contains no typological or technological diagnostics of the Gravettian. Its MAMS-38337 age (23,437 ± 140 BP; 27.3–27.8 ka) is therefore at least two millennia too old. As already noted, layer 2 of Lapa do Anecrial is comparable [2, 44]; this few cm-thick unit featured a characteristic Terminal Gravettian lithic assemblage scattered around a well-preserved hearth and refitted into raw material blocks that brought together >90% of the finds. Despite this apparently Pompeii-like preservation, single-item AMS dating showed that it consisted of a palimpsest of two occupations. The main Terminal Gravettian one, dated to 21,560 ± 220 BP (OxA-5526; 25.3–26.3 ka) by a piece of *Erica* sp. charcoal from the hearth, had taken place directly atop an earlier floor defined by a small assemblage of somewhat patinated lithic and faunal remains, including two of the nine refit units and a cut-marked pelvis of *Capra pyrenaica* dated to 23,410 ± 170 BP (OxA-11235; 27.3–27.8 ka). This earlier age implies that the surface extant at the time of the Terminal Gravettian occupation had remained exposed for one to two-and-a-half millennia.

Such sedimentation hiatuses being climate-driven, one would expect them to impact regional sites in like manner and, therefore, that one ought to exist at Caldeirão too. Indeed, the notion that the accumulation of layer Ja was both preceded and followed by palimpsest-generating sedimentation hiatuses is supported by two lines of evidence. Firstly, there is the difference in age between layer Jb, bounded by dating to the 28.7–31.0 ka interval, and the 25.5–26.0 ka temporal horizon for the beginning of the Protosolutrean technocomplex that we find represented in immediately overlying layer Ja (Table 5; Fig 15). Secondly, there is the fact that the MAMS-38337 result for Ja is nearly identical to that for the *Capra* material in layer 2 of Anecrial. Like the latter, layer Ja must therefore be seen as a multi-component stratigraphic unit comprising archaeological remains discarded through a period of at least two-and-a-half millennia prior to the onset, *c*. 25 ka of the LGM. In these circumstances, the geometry of the Caldeirão deposit predicts the presence of residual material in the stratigraphic unit formed once sedimentation resumed.

To sum up: much as layer Fa of the Back Chamber incorporates earlier finds brought up by coeval burrowing taking place upslope, in the Corridor, so layer I can be expected to incorporate items derived in like manner from the underlying Ja palimpsest. Alternatively, the layer I sample could represent a specimen whose derivation was syn- rather than post-depositional, i.e., a bone from the Late Gravettian-to-Protosolutrean deposit that gravitated downslope from an area closer to the porch of the cave where that palimpsest remained exposed (and eventually became incorporated in the unit coevally forming inwardly, in the Back Chamber—layer I).

#### 3.1.4. The early upper palaeolithic

It is in light of the above that the comparable issues affecting Early Upper Palaeolithic layer K must be looked at. The small size of both excavated areas and stone tool assemblages makes it difficult to assess the stratigraphic integrity of this part of the sequence using lithic taphonomy. It is therefore highly significant that, despite the low probability of success, two quartzite flakes from layer K have been found to refit—the single refit so far identified in the site’s Transition levels. The two flakes are horizontally separated by 1.40 m and vertically by 78 cm, which makes for a connection link that closely follows the stratification’s angle of dip (Fig 22B and 22C).

This evidence brings additional support to the notion that, as otherwise indicated by the dated samples, layer K is significantly intact and chronologically homogeneous, at least with regards to its upper part (the K1 lens). Nonetheless, recall that (a) its basal spits (the K2 lens; Fig 5) contained quartz debitage and quartz tools that are characteristically Middle Palaeolithic (Fig 9, no. 3; Fig 11, no. 2), and (b) conversely, the uppermost spit of the immediately underlying layer L yielded a Dufour bladelet that would be at home in an Evolved Aurignacian context (Fig 10, no. 2) but is at odds with the layer’s large, typically Middle Palaeolithic quartz sidescraper (Fig 11, no. 1). Also recall that these anomalies are mirrored in the inversions that complicate the interpretation of the radiocarbon results obtained for layer L (Table 5; Fig 15): MAMS-41872 is of the same age as samples from overlying layer K; MAMS-41871 is older than underlying layer M’s MAMS-33905.

Which is the parsimonious interpretation of these data? Like the bone samples that yielded the stratigraphically anomalous results for layers Fa and I, residual or inherited material could be present in layer K. This possibility is supported by the distribution of quartz artefacts relative to the cut-and-fill disturbance feature found at the K/L boundary: the enrichment seen in layer K of P11 is matched by the absence in layer L of P12 (Fig 22C and 22D). As predicted by the Eb/Fa site formation model, burrowing of layer L during the K/L sedimentation hiatus would have brought up finds from layers L-N, which in turn would have gravitated downslope and eventually incorporate the deposit formed—higher-up stratigraphically, but lower down topographically—once sedimentation resumed. Under these premises, sediment derived from underlying units would have contributed to the build-up of layer K and, indeed, the single-grain D_e_ distribution of layer K’s OSL sample (CLD17-1) is consistent with such a locally complex depositional history (Table 6). The evidence that P11 was enriched with artefacts from the cut-and-filled parts of layers L-M upslope in square P12 supports our interpretation that the enhanced and asymmetric D_e_ scatter of CLD17-1 (collected from P11) is most likely related to minor syn-depositional mixing of externally derived (well-bleached) sediments with older generations of pre-existing cave deposits during transportation through the chamber.

A corollary of the above is that quartz may well have been quite important in the site’s latest Middle Palaeolithic, especially if we bear in mind that, in O/13-14 and P13, layer L was exposed but not excavated, and so the ratio of finds per area unit of excavation for these squares is a significant underestimation (Fig 22D). In the case of the Dufour bladelet, however, we are probably dealing with excavation error rather than post-depositional displacement. Recall that this item is a sieve find from the uppermost spit of layer L in P11 and that this square was excavated as a deep, one-square-meter test trench where the décapage of newly exposed stratigraphic units was of necessity approximate only (the more so due to the K/L boundary’s significant angle of dip, as well as, against the north wall of the cave, particular geometry; Figs 4–6 and 15). A plausible, and we believe parsimonious view here is that, like the carinated scraper/core found at the base of Unit 4 of the Entrance, this Dufour bladelet stands for fleeting usage of the site during the Aurignacian.

A potential objection to the Aurignacian being represented at Caldeirão by finds made at the elevation of the Middle/Upper Palaeolithic boundary is that the corresponding chronological signal has not been picked up by the set of layer K bone samples successfully dated so far. Possibly, this is because layer K is *c*. 50 cm-thick but the three dated samples all come from its upper 30 cm, those forming the K1 lens (Fig 5). The lower part of layer K, which corresponds to a stratigraphically distinct lens (K2), could well be of an earlier age, one bounded by (a) the set of bone ages for layer K and (b) the radiocarbon results for layers L-M. That interval is 33.3–42.0 ka, which overlaps with the range of the Aurignacian in south-western Iberia [16].

### 3.2. Age of the middle palaeolithic

The archaeological and geo-stratigraphic observations summarised above show that the L-O sequence is made up of deposits that retain their original organisation (Fig 5). Crucially, the OSL results corroborate that such is the case and rule out interpreting the reversals seen in the radiocarbon record as due to large scale reworking. Therefore, décapage error and minor, localised disturbance are the possible explanations that must be explored.

For the layer L radiocarbon sample that returned a layer K-like result (Fig 14E), excavation error is most likely (as with layer L’s Dufour bladelet). The other sample from layer L (Fig 14D; MAMS-41871) is older than underlying layer M’s (Fig 14F and 14G; MAMS-33905) but, in a deposit formed downslope of cut-and-fill features—as is the case, in square P11, with layer L—it is to be expected that inherited material will be present (as indeed observed in layers Fa and I). Under the assumption that incorporation of reworked items is the primary cause of the Back Chamber’s few dating anomalies, it is also legitimate to view in like manner the sample from unit 5 of the Entrance, which turned out to be somewhat older, statistically, than the sample from unit 6 (Fig 17). The plausibility of these interpretations is strengthened when the size of the bone fragments used in the dating of layers L-M and units 5–6 is considered; all were rather small and thereby susceptible to vertical mobility in the context of localised, limited disturbance. Based on these premises, the lower boundary of the 95.4% probability ranges of the results for unit 6 and layer M provide a *terminus post quem* of 38.5 ka for the end of Caldeirão’s last Middle Palaeolithic occupation (Figs 15 and 17).

It must be noted here that the D_e_ distributions of the OSL samples from layers L, M, and N—close to the ideal for single-grain dating—are not inconsistent with the presence therein of inherited bone finds. Heterogeneity in the ages of individual grains from a given OSL sample would only be expected in the case of layer K, whose accumulation followed a long hiatus and was associated with mobilisation and downslope redeposition of sediment from the cave floor extant upslope. This process would have entailed generalised syn-depositional mixing of sedimentary particles at the bottom of the slope, in P11, as indeed inferred from this square’s artefact content (Fig 22C and 22D) and revealed by the enhanced and asymmetric D_e_ scatter of its OSL sample (CLD17-1; Fig 16A). In a situation such as layer K’s, single-grain OSL dating would detect heterogeneity in the ages-of-last-exposure of individual quartz grains regardless of where exactly in P11 the point of sample acquisition had been positioned. In contrast, for layers L, M, and N, where post-depositional disturbance is limited to localised intrusion, single-grain OSL dating would only have picked up significant inter-grain D_e_ heterogeneity if the sampling had been carried out in disturbance-affected areas; as explained in the S1 File, due care was taken to avoid such areas.

Major stratigraphic discontinuities can also be associated with the presence in the layer below the boundary of intrusive items that are younger than the time of sedimentary accumulation. At Caldeirão, examples thereof are (a) the bone remains of Magdalenian age whose presence in the bulk sample from layer Fa must be the explanation for the unexpectedly young, post-Solutrean conventional result ICEN-69 (15,170 ± 740 BP; see above [2]), and (b) the directly dated human bones of Early Neolithic age that made their way into Solutrean layer Fb (Fig 15). Thus, rather than excavation error, the Dufour bladelet and the bone fragment from layer L whose age turned out to be in the range of layer K’s (the MAMS-41872 sample) could represent intrusions, and ditto for the bone fragment from layer M (the MAMS-33905 sample). If so, the best assessment of the age of Caldeirão’s latest Middle Palaeolithic would be given by the youngest of the other two results (MAMS-41874 for unit 5, and MAMS-41871 for layer L), i.e., no earlier than 39.5 ka (Tables 5 and 8; Figs 15 and 17).

A caveat to bear in mind here is that, when working close to the limit of applicability of the radiocarbon method, failure to remove even very small amounts of contaminating material will produce dating results younger, often much younger, than a sample’s true age [77]. This is the more so in the case of burnt bone, the 35.6–38.6 ka results for layer 8 of Gruta da Oliveira (Torres Novas, Portugal; Fig 1, no. 5), now robustly dated to MIS 5a by U-series and luminescence, being a case in point [19, 78]. Radiocarbon dating of bone samples treated with the standard, Longin-derived bulk collagen-extraction method has a record of underestimating the true age of Middle Palaeolithic samples, and results based on single amino-acid dating have recently shown that underestimation may also happen, although rarely and to a lesser extent, even when, as is the case here, ultrafiltration was used (see Materials and Methods for details) [79, 80]. Conceivably, it could therefore be the case that all of Caldeirão’s Middle Palaeolithic radiocarbon results represent minimum ages only; however, they are not close to the limit of applicability of the method, are consistent with the luminescence results, were obtained by the same laboratory that produced the most recent batch of results for the Transition levels of nearby Lapa do Picareiro (Alcanena, Portugal; 25 km WSW of Caldeirão; Fig 1, no. 4) [81], and that laboratory has successfully pre-treated and dated bone samples that yielded age measurements >45 ka [82]. There is, therefore, little reason to assume that dating error must be the primary explanation of the age-depth anomalies.

Another caveat is that the dated bone samples bore no signs of anthropogenic modification and came from faunal assemblages accumulated by carnivores. However, to contest the persistence of the Middle Palaeolithic at Caldeirão beyond 38.5–39.5 ka based on an agency argument requires positing that, with a single possible exception (the layer L sample that yielded the MAMS-41871 age of 40.9–42.0 ka), the carnivore activity reflected by the dated bones occurred after the time of human occupation and sediment deposition. In such a scenario, the site’s dating record might be reconciled with the time horizon for the Transition in northern Spain’s, i.e., *c*. 41.5 ka; but one would still need to explain why four of the dated bones would be intrusive (but not the fifth), and one would also have to make a case for ruling out the equally likely possibility that said carnivore activity predated or was coeval with the human visits documented by the artefacts.

Another problem with using MAMS-41871 to argue that Caldeirão’s Middle Palaeolithic must predate 41–42 ka is the length of the hiatus that, if so, would separate layer L from layer K: eight millennia, if we use the time range indicated by available radiocarbon dates, four to six millennia if we account for the possibility that the undated K2 lens is of Evolved or Late Aurignacian age (see above). Conceivably, erosion could be the answer, given that the angle of dip of the stratification suggests deposition along an unimpeded slope until the end of the Solutrean. However, bear in mind that, in P11, Upper Palaeolithic layers Fa-K are *c*. 2.5 m-thick and span some ten millennia (Fig 15); assuming that the deposit putatively lost to erosion formed at a comparable rate, we would be dealing with the removal of one meter, minimally, of the original accumulation. However, nothing even remotely suggests that the high-energy processes required to bring about such an outcome were in operation at any point during the accumulation of the site’s Upper Pleistocene infilling.

The long hiatus required to make layer L predate 41–42 ka would therefore have to be one generated primarily by an arrest in sedimentary deposition. An analogue might be the hiatus that does exist at the site higher-up in the succession, between the end of the Magdalenian and the resumption of sediment build-up in Epicardial times. From available dating results—e.g., OxA-1037 (5970 ± 120 BP) for layer Ea, and ICEN-72 (10,700 ± 380 BP) for the upper part of layer Eb of the Corridor area [1, 2]—we can estimate the duration of that hiatus to be in the range of four millennia. In this instance, a good explanation for the arrest is provided by the stabilisation of exterior soils resulting from a dense, Early Holocene forest cover. However, no interval of climate amelioration of similar intensity and duration existed during MIS 3, much less one lasting almost twice as long.

To sum up: (a) based on the five radiocarbon dates for layers L-M and units 5–6, the *terminus post quem* for the K/L boundary falls in the 38.5–39.5 ka interval; (b) although it cannot be ruled out that the age/depth inversions seen in that set of results reflect incomplete decontamination of the samples from layer M and unit 6, they are more likely to reflect post-depositional disturbance or excavation error; (c) the luminescence ages for layers L-N are consistent with these conclusions.

### 3.3. Correlation with global records

**Fig 23:**
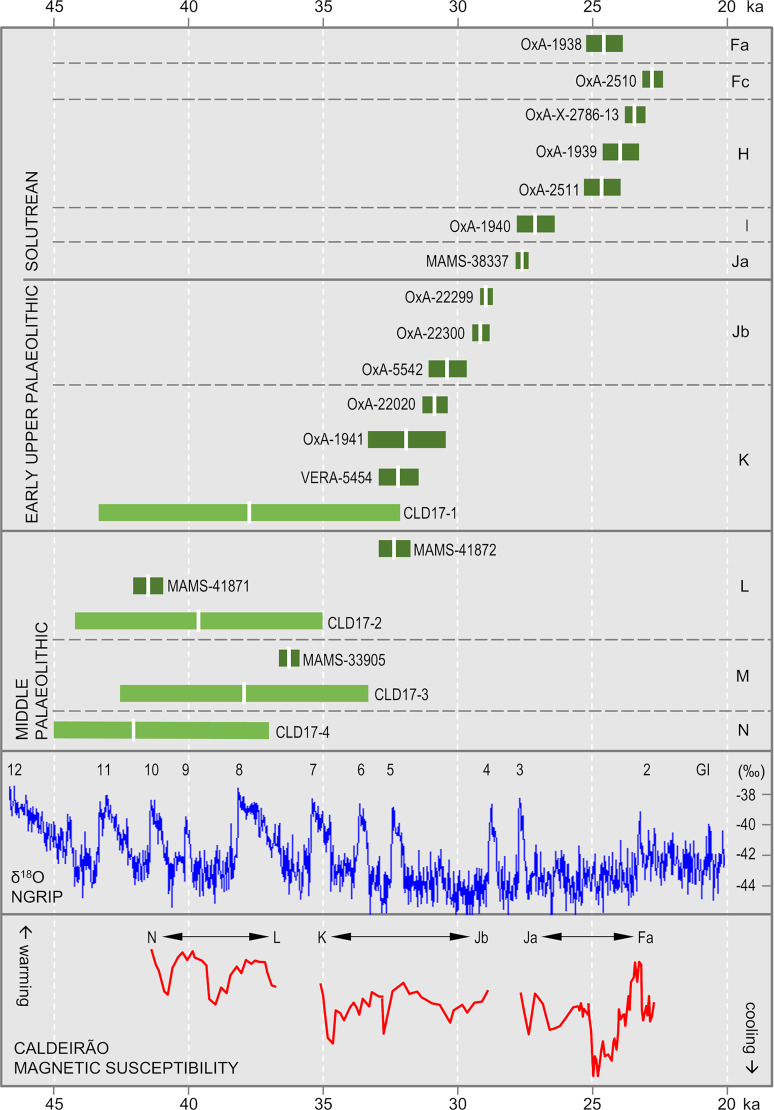
Chronology and palaeoenvironmental context of the transition at Gruta do Caldeirão (back chamber). The bars indicate the 95.4% probability intervals of the calendar ages in Tables 5 and 6. OxA-37728 and OxA-37729 (which date intrusive human bone from the overlying Cardial context), OxA-5521, OxA-5541 and OxA-8670 (which reflect incomplete decontamination and are minimum ages only), and OxA-22301 (which is from a subfossil marine shell) have been excluded. Plotting of the NGRIP climate curve used CalPal (version 2017.5) [83]. The magnetic susceptibility curve is from Fig 6A, here sectioned into segments whose length has been adjusted to account for the hiatuses and the dating.

Fig 23 plots the dating results for the Back Chamber against (a) the NGRIP oxygen isotope curve and (b) the MS curve in Fig 6A (sectioned to account for the hiatuses and adjusted to fit the dating evidence as interpreted in the preceding discussion). For clarity, we excluded the samples that could be identified as intrusive or subfossil, as well as those with younger-than-expected ages whose chemistry or associated dating laboratory’s “health warning” unambiguously point to an error caused by incomplete decontamination (cf. Fig 15).

At the latitude of Portugal and in limestone terrain, soils are rich in maghemite, and so the alternation of cool/wet and dry/hot periods produces significant variation in iron mineral content and, hence, in MS values (lower during the former, and higher during the latter periods). In caves, the rationale for using the MS signal as a climate proxy is that (a) sedimentary infillings are made up of particles derived from the erosion of the karst’s coeval soil cover, and (b) the stable, unlit interior environment precludes further pedogenesis. Based on these premises, cave sequences with long and continuous clay-rich deposits and minimal bioturbation provide reliable archives of regional climate change.

The Caldeirão sequence provides good illustration of the relationship between global climate and MS [2, 28, 29]: the values for pre-LGM layers I-K are intermediate, the lowest values are in layers Fc-H, in agreement with their LGM age, and the highest values are found in layers L-N. Under these premises, layers L-N must belong in an unusually mild interstadial period. We therefore correlate them with the long interval of predominantly interstadial conditions that prevailed in Iberia between GI 8 and GI 12, which only the brief cold spell of GS 9 (38,220–39,900 years ago), associated with HS (Heinrich Stadial) 4, punctuated in any significant manner (as indicated by the pollen evidence from deep sea cores drilled off the Portuguese coast. [84]). Compared with the average picture observed through that interval, tree cover was already significantly diminished by *c*. 29–33 ka [85], fully in line with the intermediate MS values obtained for layers Jb-K.

The high MS values in layers L-N are consistent with the bracketing OSL dating results, which constrain the accumulation of these deposits to the *c*. 35–47 ka interval, i.e., to between the onset of GI 12 and the end of GI 7 [28]. Taking in due consideration the age of layer K and the major hiatus existent at the K/L boundary, it therefore makes sense to align the three MS peaks found in layers L-N with the GI 8-GI 9-GI 10 sequence. Following the same logic, the climatic amelioration implied by the increased water dripping and attendant cementation of layer O can be correlated with the long period of interstadial conditions comprised between the end of GI 11 and the onset of GI 12 (42.2–46.9 ka). This reasoning supports the radiocarbon-based *terminus post quem* of 38.5–39.5 ka for the Middle Palaeolithic of Caldeirão and implies an age in the GS 13-GS 16 interval (46.9–58.2 ka) for the deposition of layer O, consistent with its OSL age of 58.4 ± 3.8 ka. If so, layer P below, as yet undated, may well be of MIS 4 age.

Following the same logic, the episodes of flowstone development capping layers Fa and H can be correlated with short intervals of enhanced humidity—likely, the minor and very short episodes of climate amelioration that punctuated the LGM, GI 2.1 (22,900–23,020 years ago), and GI 2.2 (23,220–23,340 years ago [28]. Further down, the interpretation of layer Ja as a Late Gravettian-to-Protosolutrean palimpsest implies a significant slowdown of the accumulation at that point in time, while the set of available radiocarbon results reveals that a millennium-long hiatus would seem to exist between layers Ja and Jb. Given the dating, that hiatus would seem to coincide with GS 4 (27.8–28.6 ka).

Based on these correlations, the rather thick Fa-Fc ensemble would have accumulated quite rapidly—between *c*. 23.4 ka (the beginning of GI 2.2) and *c*. 22.9 ka (the end of GI 2.1), i.e., over a duration of four to five centuries. This inference is consistent with (a) the characteristics of the deposit, a chaotic-like slope sediment (see above), (b) its homogeneous Upper Solutrean content, and (c) the well-defined chronological boundaries of this assemblage-type (as recently illustrated at La Boja, where the Upper Solutrean dates to *c*. 23 ka and the younger limits of the 95.4% probability intervals of the results obtained for it are all >22.5 ka) [16, 86].

Regionally, these interpretations of the Caldeirão record are also consistent with the evidence from Picareiro [87, 88]. Here (a) concordant ages have been obtained for Upper Solutrean levels T-upper, S, and R, (b) sedimentation rates have been found to increase markedly at the time of the LGM, and (c) radiocarbon ages falling in the GS 4 stadial are missing from the cave’s detailed dating record, suggesting that, across the karst areas of central Portugal, this stadial may well have corresponded to a phase of generalised erosion.

### 3.4. Bayesian modelling of the succession

**Fig 24:**
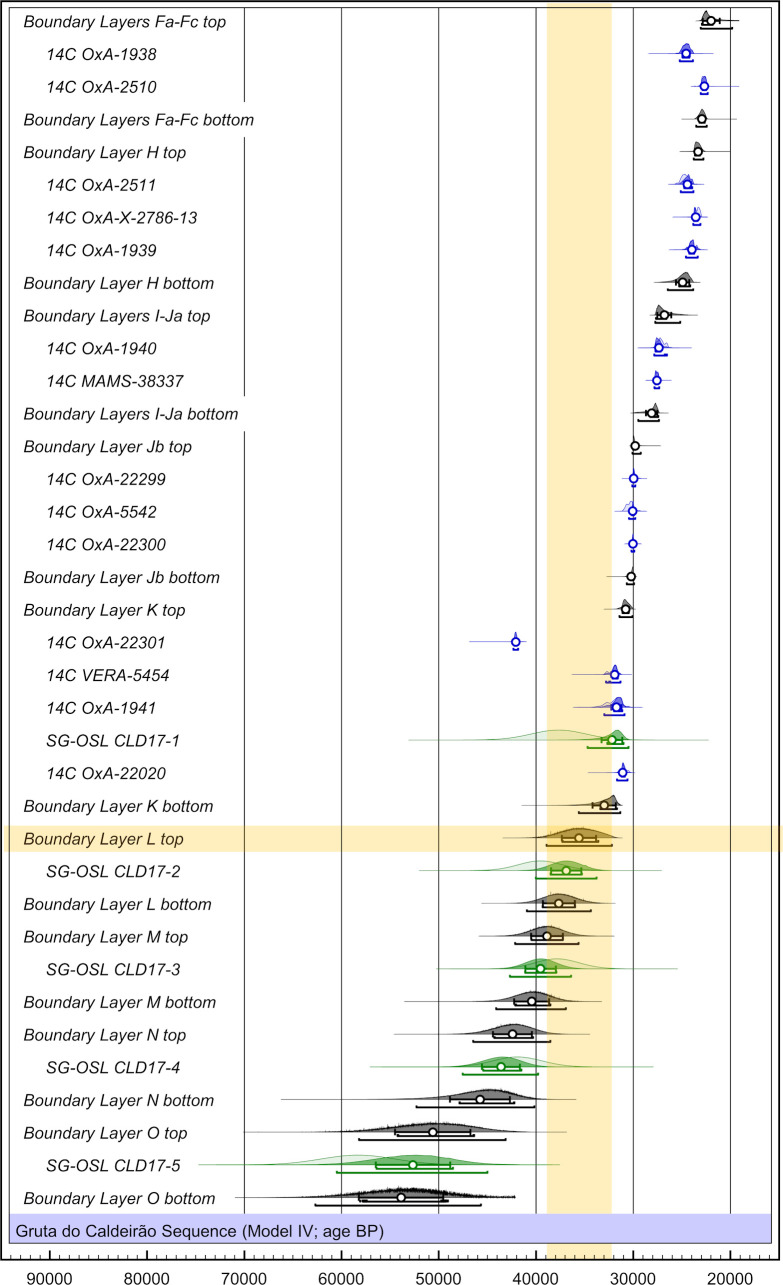
Bayesian modelling (back chamber). Model IV (cf. Table J for a summary of priors and likelihoods, and Table N for posteriors and statistical parameters). The prior age distributions for the dating determinations (likelihoods) are shown as light coloured probability density functions (PDFs): blue = radiocarbon determinations; green = single-grain OSL determinations. The modelled posterior distributions for the dating determinations and stratigraphic unit boundaries are shown as dark coloured and grey PDFs, respectively. Unmodelled and modelled ages are shown on a calendar year timescale, and both are expressed in years before AD1950. The white circles and associated error bars represent the mean ages and 1σ uncertainty ranges of the PDFs. The 68.3% and 95.4% ranges of the highest posterior probabilities are indicated by the horizontal bars underneath the PDFs.

**Fig 25:**
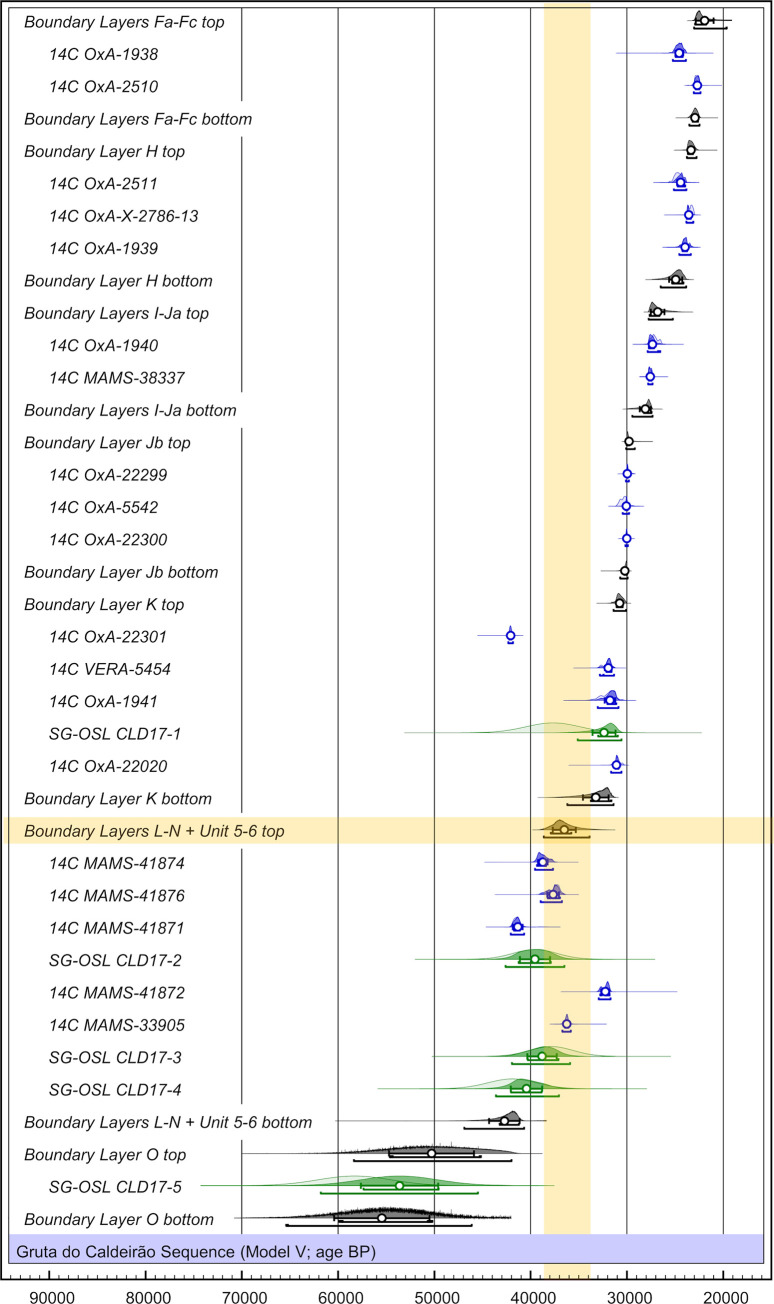
Bayesian modelling (back chamber and entrance). Model V (cf. Table J for a summary of priors and likelihoods, and Table O for posteriors and statistical parameters). The prior age distributions for the dating determinations (likelihoods) are shown as light coloured probability density functions (PDFs): blue = radiocarbon determinations; green = single-grain OSL determinations. The modelled posterior distributions for the dating determinations and stratigraphic unit boundaries are shown as dark coloured and grey PDFs, respectively. Unmodelled and modelled ages are shown on a calendar year timescale, and both are expressed in years before AD1950. The white circles and associated error bars represent the mean ages and 1σ uncertainty ranges of the PDFs. The 68.3% and 95.4% ranges of the highest posterior probabilities are indicated by the horizontal bars underneath the PDFs.

Whether observed or inferred, the Back Chamber’s succession features discontinuities that preclude the use of age/depth modelling. To test the chronostratigraphic outline above, we therefore proceeded within a Bayesian framework, using the OxCal software tools [89, 90] and the set of radiocarbon and luminescence ages in Fig 23. We ran five models in which layers I-Ja, and Fa-Fc were treated as single units to mitigate against palimpsest effects and the presence of reworked items (S1J Table in S1 File). For these reasons, OxA-1938 for Fa and MAMS-41872 for L were considered as maximum and minimum ages, input with the *After* and *Before* commands, respectively; for completeness, we also considered the OxA-22301 result for the *Semiccassis saburon* shell from K as a maximum age, likewise input with the *After* command. Model outcomes are listed in Tables K-O and plotted in Figs 24 and 25 and M-O.

Within the Upper Palaeolithic, the boundaries and maximum durations calculated for the different stratigraphic units and ensembles remain quite stable across the different models. A maximum duration of between three and five millennia is returned for layer K. This variation is an artefact of the imprecision introduced by the lack of ages for the basal part of the unit, which affects the calculation of the lower limit; interestingly, Model III estimates a maximum beginning age of 35.3 ka, consistent with the hypothesis that a component of Late or even Evolved Aurignacian age may well exist in said basal part. Layer Jb is estimated to last no more than one millennium, within an interval bracketed by the extremes of 30.1–30.5 ka and 29.2–29.6 ka. Its accumulation would thus have ended just before the onset of GS 4, as suggested above based on the correlation between the Greenland isotope evidence and the site’s MS record.

Layer Ja is estimated to begin no later than 28.8–29.1 ka, while estimates for how late the end of layer I may have been vary (depending on whether it is considered together with, or separately from Ja) between 25.5 and 26.0 ka. These estimates remain consistent with the notion that a hiatus coinciding with GS 4 separates layers Ja and Jb, but the end date returned for layer I is older than expected based on archaeological content. This contradiction would probably be less apparent if OxA-1940 for layer I had been input with the *After* command, in accordance with our interpretation of the sample as a reworked item, but we decided against constraining the models beyond what was strictly necessary to account for the obvious anomalies.

This decision impacted the estimation of the time when layer H began to accumulate, which all models extend to possibly as early as between 25.3 and 25.5 ka. Conversely, all models produce an overlap between the youngest possible age for H (23.0–23.1 ka) and the oldest possible age for the beginning of Fa-Fc (23.4 ka). Compared with the known duration of the Middle Solutrean, the two-and-a-half millennia allowed for the duration of H are three to five times in excess of what might be expected. However, the <23.4 ka constraint returned for Fa-Fc is in good accord with the placement of these units between GI 2.2 and GI 2.1 (22.9–23.4 ka); the overlap with the earlier parts of GS 2 implied by the younger limit of the maximum duration interval (20.8–21.3 ka) must reflect the lack of a reliable radiocarbon age constraint for Fa.

With regards to the Middle Palaeolithic, we focused on the extent to which model outcomes were impacted by assumptions concerning accuracy and stratigraphic association. The difference between Models I and II is that, in order to account for reworking, layers L-N were considered separately in Model I and treated as a single phase in Model II. In Model III, we additionally constrained MAMS-33905 for layer M with the *Before* command (i.e., treated it as a minimum age only).

The set of assumptions in Model II is consistent with the site formation analysis and the chronostratigraphic framework outlined in the preceding sections. In contrast, by placing a boundary between layers L and M, or by leaving open the possibility that the result for layer M be too young, Models I and III give more weight to MAMS-41871 (which we left unconstrained in all models); they therefore represent a worst case scenario for the hypothesis that the Middle Palaeolithic persisted at the site beyond 39 ka. Not unexpectedly, the results obtained for the age of the K/L boundary were significantly impacted by the changes made to the priors: within the 95.4% probability intervals of 31.9–41.4 ka and 34.5–41.8 ka for Models I and III, respectively, and of 32.6–36.6 ka for Model II—i.e., a difference of five millennia in *terminus post quem* terms.

To test the models’ sensitivity to the radiocarbon ages we then ran Model IV, which only considered the OSL ages and did so in stratigraphic order. For the K/L boundary, a *terminus post quem* of 38.9 ka was returned (Table N; Fig 24). This outcome showed that the worst case scenarios (Models I and III) ought to be considered unrealistic and that modelling the Middle Palaeolithic units as a single phase best accounts for the uncertainties created by the issues of reworking and accuracy involved. This conclusion was corroborated by the running of Model V, which is the same as Model III but assumes that units 5–6 of the Entrance are coeval with layers L-N and adds them to the calculation of the boundaries for such a single, site-wide Middle Palaeolithic phase. For the K/L boundary, doing so returns essentially the same *terminus post quem* (38.6 ka) as obtained with Model IV and this despite the inherent downweighting of the MAMS-33905 result for layer M (Table O; Fig 25).

Based on the above, we conclude that the parsimonious explanation for the age/depth anomalies found in the set of radiocarbon ages for the Middle Palaeolithic levels of Caldeirão is indeed minor, localised post-depositional disturbance, not dating error. Using those ages within a single-phase framework, as in Model V, accounts for the inversions and provides a reliable chronology. The estimate returned for the K/L boundary is consistent with (a) the notion that the boundary corresponds to a hiatus in sedimentation representing a local manifestation of the impact that GI 8 (36.6–38.2 ka) had on the karst environments of western Iberia, and (b) persistence of the Middle Palaeolithic at the site beyond 39 ka and into GI 8, i.e., into the time range of the Early Aurignacian of northern Spain and southern France. The estimate returned for the boundary between layers N and O—within the 95.4% probability interval of 40.7–46.9 ka—is in turn consistent with it being a local manifestation of GI 11-GI 12 (42.2–46.9 ka), as above proposed based on the MS data. In short, the chronostratigraphic interpretation of Caldeirão proposed in Fig 23 passes Bayes’ test.

### 3.5. Site function and palaeoenvironmental inferences

The dating work makes it clear that the Back Chamber’s Pleistocene succession hides much variation in rates of sedimentation (Table 10). In caves, site-specific factors, such as the opening of new sediment entry points, the development of chimneys, or the emergence of blockages can impact sedimentary dynamics. However, (a) our excavation trenches have allowed us to follow the cave’s morphology across >90% of its length, revealing that it corresponds to a simple volume developed along a single, linear conduit, (b) the matrix of layers Fa-P shows that we are dealing with correlative deposits reflecting variation in the intensity of soil-sediment inwash, (c) in squares P/11-12, at the bottom end of the Back Chamber, the Pleistocene succession does not contain any boulders, and (d) it is only after the LGM, in connection with the deposition of layers Eb, Ea, and ABC-D, that the inferred cluttering-up of the conduit beyond the Back Chamber and the upward development of roof chimneys significantly changed the dynamics of the accumulation (see above).

**Table 10:** Data for the estimation of rates of sedimentation and density of finds.

Layer	Maximum duration (years)*	Thickness (in P11 at y = 80) (cm)**	Minimum sedimentation rate (cm/10^3^ years)***	Area (m^2^)**	Volume (m^3^)**	Fauna (g)**	Chert (piece-plotted) (N)	Chert (N)	Chert (g)
**N**	2770	21	7.6	0.89	0.180	250	1	2	24.5
**M**	2770	21	7.6	0.94	0.190	220	1	1	3.7
**L**	2770	33	11.9	1.77	0.410	700	–	3	1.4
**K**	4020	50	12.4	3.31	1.783	6520	3	14	49.5
**Jb**	980	26	26.5	4.68	1.136	3270	8	19	302.0
**Ja**	1405	27	19.2	6.25	1.298	4070	28	41	269.7
**I**	1405	17	12.1	6.61	0.944	3070	15	31	166.3
**H**	2430	13	5.3	7.82	0.950	3300	83	–	–
**Fc**	830	13	15.7	10.11	1.080	4620	59	–	–
**Fb**	830	49	59.0	10.50	2.280	6180	119	–	–
**Fa**	830	43	51.8	9.04	2.720	6100	240	–	–

Per stratigraphic unit (Back Chamber and Corridor)

*based on the difference between the upper and lower boundaries of the 95.4% probability intervals returned for each unit by Model V (cf. Table O; the maximum durations for L, I, Ja, Fc, Fb and Fa are obtained dividing in equal parts those for L-N, I/Ja and Fa/Fc)

**after [2] and unpublished data

***thickness in P11 divided by maximum duration of the unit.

The fact that sedimentation rates vary in tandem with the MS values (which are lowest in layers L-N, intermediate in layers I-K, and highest in layers Fc-H; Fig 6A) suggests that the local impact of global climate change is the main driver of the variation. The increased inwash reflected in the thickness of the Upper Solutrean deposit must stem from a much sparser plant cover of the limestone massif, with attendant greater exposure of exterior soils to erosion. Conversely, the slower process of sedimentary build-up seen inside the cave during the accumulation of layers Jb-K, prior to the LGM, is consistent with a denser plant cover, making for less erosion, and less inwash, when less harsh climatic conditions prevailed. The composition of the small assemblage of charcoal fragments from Caldeirão whose stratigraphic provenance in layer H is secure—mostly, Scots pine (*Pinus sylvestris*)—supports these inferences, as it suggests an LGM landscape of open pineland and heath (as otherwise reliably inferred on the basis of the large charcoal assemblage from coeval layer TP09 of the Lagar Velho rock-shelter [91, 92]).

The zooarchaeological analysis of the faunal assemblages indicates that human presence was infrequent and incidental during the accumulation of the basal layers of the succession. In the Middle Palaeolithic, the cave functioned primarily as a hyaena den, as revealed by the mandible retrieved in layer L and the large number of coprolites found in immediately underlying layer M (Fig 4H and 4I). Carnivores (lynx, wolf, bear, hyaena, and lion) are also well represented in the overlying pre-LGM units; they continued to be the primary agent of accumulation through layers K, Jb, and Ja. However, the faunal assemblages from the Last Glacial Maximum (LGM) and the Tardiglacial are mostly anthropogenic [32, 33].

The distances between Caldeirão and the closest known sources of chert and other high-quality siliceous rocks are significant (≥10 km, and even ≥30 km for some; [93]). In contrast, quartz and quartzite cobbles are locally and abundantly available—in the alluvium of the Nabão, in the gravelly beds of its terrace staircase, and in the Miocene detrital cover of the karst. This sourcing evidence parsimoniously explains why lithic assemblages are dominated by the products and by-products of the expedient exploitation of quartz and quartzite (Tables 2 and 9; Figs 8 and 9). That factor also explains well why the reduction sequences are, for chert, incomplete and biased towards the final stages of the *chaîne opératoire* (retouching of imported blanks; repurposing, or discard of broken, damaged, and worn-out tools), which is especially apparent in the case of the Middle and the Early Upper Palaeolithic stone tool assemblages.

**Fig 26:**
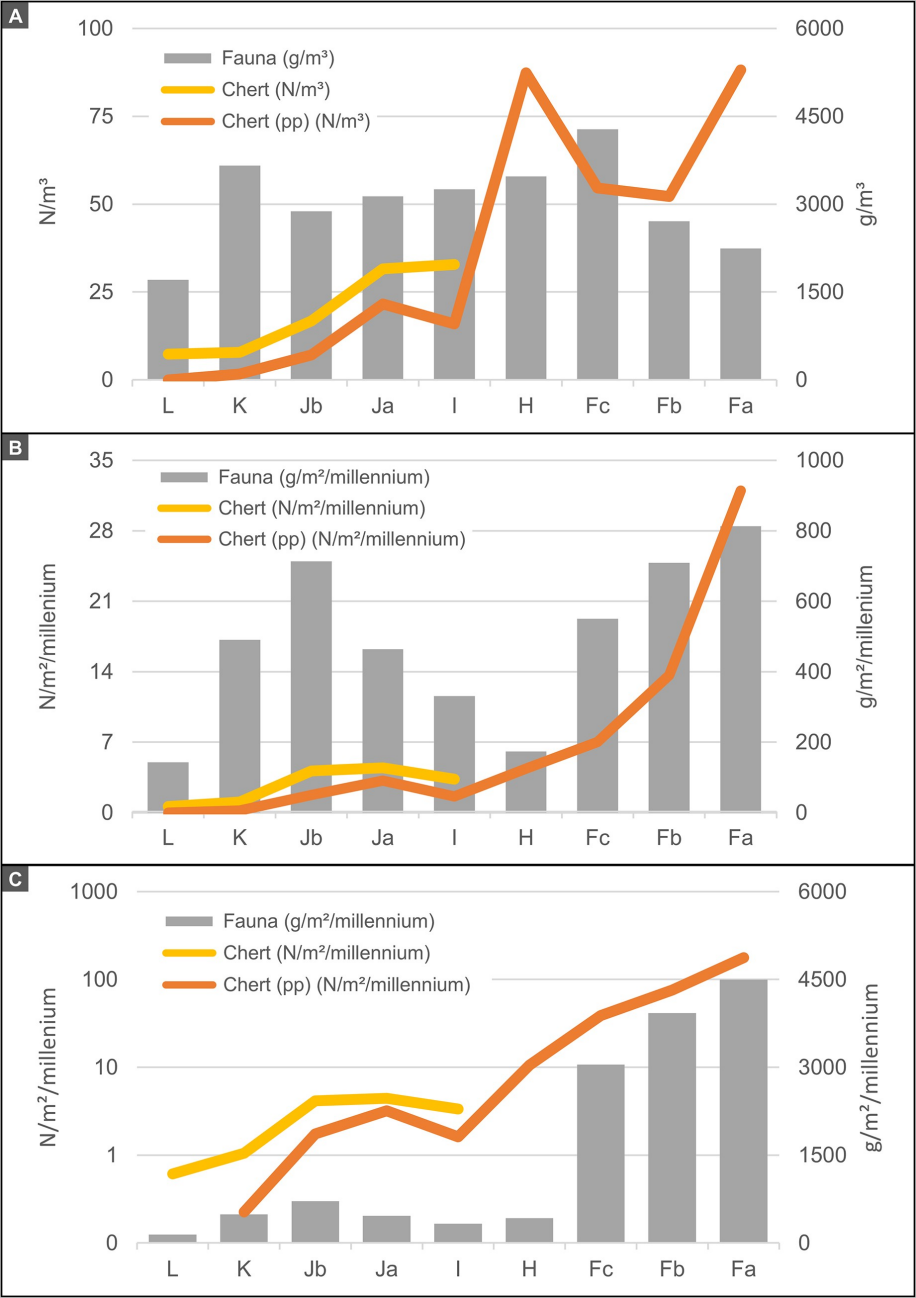
Stratigraphic variation in the rate of accumulation of archaeological remains. A. Per unit volume of excavated sediment. B. Per unit area of excavation and maximum duration based on Model V. C. Per unit area of excavation and maximum duration; maximum duration as in (B) for layers I-L but based on chronostratigraphic reasoning for layers Fa-Fc (half a millennium) and H (one millennium). The lines represent item counts (manuports excluded), and the columns represent weight counts. N = number of finds, pp = piece-plotted items.

Given distance to sources, variation in how much chert was discarded at the site is therefore the best proxy for the frequency and intensity of human visitation and usage. To enable comparison across the sequence we used the variation in the number of piece-plotted chert items because, for stone tools, only layers I-L have been weighed and counted (Table 10); excavation protocols remained the same throughout and in the units for which we have full data piece-plotted chert counts follow the same trend as total counts. There is therefore every reason to believe that the variation in the abundance of chert items is reliably tracked by the piece-plotted proxy: gradually increasing as one moves up the stratigraphy, skyrocketing in the Upper Solutrean (Fig 26).

When estimated in terms of excavated volume (Fig 26A), the amount of animal bone more than doubles from Middle Palaeolithic layer L to Early Upper Palaeolithic layer K and then remains rather stable until the Upper Solutrean, where a decrease from Fc to Fa is apparent. Yet, quite a distinct trend—as much animal bone accumulating at the site during the Early Upper Palaeolithic as during the Upper Solutrean—is revealed when duration is considered and used to calculate rates of accumulation through time and per area unit of excavation (Fig 26B). However, we must also bear in mind that the values plotted in Fig 26B are based on Model V’s maximum interval lengths (Tables 10 and O), which overestimate by a factor of five the actual duration of the time periods represented by layers Fc, Fb and Fa. The real pattern, therefore, is one where chert and faunal remains undergo a very significant co-increase in the Upper Solutrean (Fig 26C; and recall that, at this point in the sequence, the agents primarily involved in the accumulation of animal bone are no longer the carnivores but the humans).

Collectively, these data leave little doubt that people visited Caldeirão much more frequently during the Solutrean, and especially the Upper Solutrean. Their stone tool diagnostics are the kinds of items that one expects to see in logistical contexts: mostly, hunting weaponry. However, endscrapers and other domestic implements are also well represented in layers Fc-H, as is the evidence that both the *façonnage* of foliates and the production of blanks for shouldered points took place on site (Table 1). This evidence is consistent with occupations of a more residential nature than before, which is supported by the fact the surrounding area was at this time frequented by children, not just adults (as shown by O12-84, the directly dated human mandibular fragment from layer H, which belonged to a 10–12 year-old youngster; Fig 14A [2, 94]).

### 3.6. The evidence in context

Elsewhere in Portugal, and in line with the general Iberian pattern, the Upper Solutrean is the period best represented in cave sequences that span the Upper Palaeolithic [2, 95, 96]. It has been suggested that this pattern has demographic underpinnings, reflecting population growth in a refugium to which the hunter-gatherer peoples of south-western Europe retreated as a deteriorating climate covered the central European plains with uninhabitable tundra landscapes [97]. One might therefore be tempted to argue that the more frequent and intensive usage of Caldeirão seen during the Upper Solutrean is consistent with such models in that it can be taken as a proxy for a regional increase in population numbers.

Against the palaeoenvironmental backdrop, however, the peak in the rate of accumulation of archaeological remains seen in layers Fa-Fc can be parsimoniously explained by behavioural changes in the realms of subsistence and territoriality. The open landscapes of the period would have made for karst landscapes with an increased ungulate biomass, ones that would have been both richer in food resources and easier to travel across. Rather than indicating that more people were using the same amount of countryside, we therefore suggest that Caldeirão’s Upper Solutrean data reflect that karst areas were being visited and explored more often than during preceding Early Upper and Middle Palaeolithic times. The enhanced visibility (higher resolution) and protection against erosion (burial depth) offered by the sediment packages enveloping the remains discarded through the Solutrean in the caves of central Portugal further contribute to understand the period’s striking conspicuousness. The parsimonious explanation is that the archaeological contexts formed at that time are easier to detect and explore [98].

For the critical stratigraphic units found either side of the layer K/layer L and unit 4/unit 5 boundaries, both excavated areas and artefact assemblages are small. The identification of those boundaries as separating the Middle from the Upper Palaeolithic is therefore open to criticism. The fact that the dating record is imperfect in terms of age-depth consistency is another complicating factor. Yet, when considering the evidence in context, correlating those two boundaries with the Transition remains fully supported.

In Iberia, carinated scrapers/cores and Dufour bladelets have never been found in Middle Palaeolithic stone tool assemblages. Conversely, Levallois debitage and sidescrapers disappear altogether in the Early Upper Palaeolithic. In the Mula basin of Murcia (Spain), La Boja and the adjacent rock-shelter of Finca Doña Martina (Fig 1, no. 12) have provided large Mousterian, Aurignacian, and Early Gravettian assemblages that are clear-cut examples of both rules [16]. These criteria for the assessment of whether we are on the Upper or the Middle Palaeolithic side of the Transition are qualitative, not quantitative. They are widely accepted, even by authors claiming that the Aurignacian of southern and western Iberia is the same age as elsewhere in Europe: their claims are based on carinated or nosed scraper/cores from level Bj/13 of Cueva Bajondillo (Malaga, Spain; Fig 1, no. 13) [99] and level GG of Lapa do Picareiro [81] that, in both cases, are the assemblages’ single diagnostic or would-be diagnostic items.

In the Mula basin, the chronology of the Transition is based on high-quality ABOx charcoal ages taken in contexts of exceptional stratigraphic integrity excavated at two different sites located <2 km apart: the two Rambla Perea rock-shelters; and the fluvial terrace-in-shelter of Cueva Antón (Fig 1, no. 11). Here, the IntCal13 curve constrained to 36.5–37.1 ka the interval during which Middle Palaeolithic technological systems were replaced by Upper Palaeolithic ones [16]. However, application of the updated IntCal20 curve narrows that interval down and shifts it back by some five centuries, to 37.0–37.4 ka [41, 100]. In the Côa Valley of Portugal, at the Cardina/Salto do Boi site, there is a long, consistently luminescence-dated Middle Palaeolithic sequence preserved in fluviatile sediments whose uppermost unit, GFU 5/UA 11, is chronologically bounded by a *terminus post quem* of 39.5 ± 1.8 ka (for GFU 5/UA 12 below) and a *terminus ante quem* of 33.6 ± 2.0 ka (for the earliest Upper Palaeolithic with Dufour bladelets in GFU 5/UA 10 above) [101]. Thus, both the Mula basin and the Côa Valley data are fully consistent with the Middle Palaeolithic persisting at Caldeirão beyond 39 ka.

Palimpsest effects and post-depositional disturbance at the Middle/Upper Palaeolithic interface are ubiquitous features of Iberian cave deposits, with implications for sample mobility, décapage error, and dating by association. Even though archaeologists have all too often failed to acknowledge these kinds of issues, they are widespread and must be borne in mind at all times [18]. Caldeirão is a case in point, and it is not a particularly problematic site. Indeed, Cueva Bajondillo and Lapa do Picareiro are also good examples of the rule.

Bajondillo levels Bj/13-Bj/12 have been claimed to be Aurignacian and date to 40.6–44.8 ka [99]. However, prior to obtaining the dating results underpinning the claim, the excavator of the site had consistently referred to those levels as containing a solifluction-generated mix of Middle Palaeolithic and “Upper Palaeolithic-like” (sic) material [102]. In fact, as others have subsequently shown [103, 104], Bj/13-Bj/12 contain nothing that might be considered diagnostically Upper Palaeolithic, let alone Early Aurignacian or even Aurignacian *lato sensu*.

The suggestion that an Early Aurignacian dating to 41.1–38.1 ka exists at Picareiro is based on the radiocarbon ages for levels GG, HH, and II and their Bayesian modelling [81]. At the base of this sequence, level II is posited to represent the earliest phase, dated by two samples that imply a *terminus ante quem* of 41.0 ka: MAMS-42282 (36,670 ± 220 BP; 41.2–42.0 ka), and MAMS-42278 (36,390 ± 210 BP; 41.0–41.8 ka). However, the Aurignacian diagnostics all come from level GG; levels HH and II yielded no artefacts.

As discussed in-depth elsewhere [105], only the five GG results should be considered of relevance to establish the chronology of the Aurignacian at Picareiro. Those results cluster into two statistically distinct groups, strongly suggesting a palimpsest situation: the earlier cluster, represented by three results in the 37.9–39.5 ka range, may well relate to carnivore activity; the younger cluster, represented by two results in the 35.6–38.8 ka range, overlaps with those for La Boja and the Aurignacian sequence of Cova de Malladetes (Valencia, Spain; Fig 1, no. 10) [106] and may well be that which reliably reflects Aurignacian human activity at the site. Under this alternative view, Picareiro remains consistent with the Middle Palaeolithic persisting at nearby Caldeirão into the timespan of the Early Aurignacian.

## 4. Conclusions

The Caldeirão sequence sheds light on two key moments of the Upper Pleistocene prehistory of Portugal and Spain: the Transition, and the LGM. In both cases, the site provides good illustration of how purely natural, mostly geological processes may impact the generation or illusory archaeological patterns whose interpretation requires a critical, taphonomy-oriented approach.

In Iberia, the *c*. 36–41 ka time interval was one of extreme climate variability. During Heinrich Stadial (HS) 4, which lasted for a few centuries around 40 ka, aridity was extreme, and semi-desert environments expanded across the Mesetan hinterland and the badlands of the middle and upper Ebro basin. Conversely, the GI 8 interstadial was a period of milder, wetter climatic conditions during which, below 40° N, mountain forests and wooded landscapes underwent significant expansion [85, 107–110]. These oscillations must have impacted human settlement patterns as much as the geological archives of the corresponding archaeological evidence. One would predict such to be especially the case with regards to cave and rock-shelter sites, where those five millennia are bound to have witnessed the rapid alternation of periods of erosion and periods of non-accumulation, favouring the scarring of deposits and the formation of complex palimpsests. It is no wonder, therefore, that elucidating the technological and human population processes of the Transition in Iberia remains such an empirical challenge.

Conversely, increased sediment inwash at the time of the LGM facilitated the preservation of a rich record of the Upper Solutrean at Caldeirão. However, a major stratigraphic discontinuity marks the end of this phase of massive accumulation at the site. That discontinuity is associated with localised but significant post-depositional disturbance and the overlying deposit corresponds to a long period during which sedimentation was slowed down or altogether halted. Given the lack of rich, well-defined, stratigraphically intact Solutreogravettian and Magdalenian contexts in almost all known cave sites of Portugal, this pattern must have regional validity and reflect the prevalence of palaeoenvironmental conditions favouring an early, post-LGM development of forests across the low elevation karst areas of the region. As shown by the number and richness of the Gravettian and Magdalenian open-air sites currently known in the country, neither demography nor adaptation explain the fact that, in caves, the latter is hard to come by and the former is mostly represented by contexts that associate small numbers of artefacts with faunal assemblages primarily accumulated by carnivores.

The few age/depth anomalies seen in the radiocarbon dating record of Caldeirão can be explained by well-known and well-understood processes of post-depositional disturbance. For the end of the Middle Palaeolithic, the radiocarbon and luminescence ages set a clear *terminus post quem* of 39 ka. Pending the results of the new fieldwork planned for 2021–22, the evidence presented here supports the notion that, in Portugal, the Middle Palaeolithic persisted as long as in the Mula basin of Spain, i.e., for some four millennia longer than in Catalonia and the Cantabrian strip [16]. The evidence from the other side of the Transition remains consistent with the notion that, south of the Ebro basin, the earliest Upper Palaeolithic is an Evolved Aurignacian emerging no earlier than 37.5 ka. Represented at nearby Picareiro and in the open-air site of Gato Preto, this phase is also quite possibly represented at Caldeirão by the diagnostics (Dufour bladelet, carinated scraper) found at the interface between layers K and L of the Back Chamber and units 4 and 5 of the Entrance.

It remains entirely possible that this Ebro Frontier pattern is an artefact of insufficient data, or dating error, and there is no question that advances in dating techniques have led to a significant decrease in the number of cave sites supporting a late persistence of the Middle Palaeolithic in eastern, central, southern and western Iberia [111]. Still, no convincing case can be made for humans to have become extinct in those regions *c*. 41.5 ka, as some have proposed [112]. The technocomplexes that in Catalonia, the Cantabrian strip, and southern France occupy the subsequent chrono-stratigraphic slot are the Protoaurignacian and the Early Aurignacian [113, 114]. Yet, representative assemblages of these phases of the Aurignacian, or even isolated finds of their index fossils remain stubbornly absent from the archaeological records of Iberian regions located to the south of the Ebro drainage basin. With current evidence, there is no reason to think that the chronology of the Transition in central Portugal would have been out of phase with that in south-eastern Spain.

The Ebro Frontier model tries to make sense of these facts and is not contradicted by the new dating work reported here. Caldeirão continues to support the hypothesis that the people living in Portugal through the few millennia of poor archaeological visibility between 37.5 and 41.5 ka were bearers of a Middle Palaeolithic technology, not of an Upper Palaeolithic one.

## 5. Materials and methods

The finds from the 1979–88 excavation of Gruta do Caldeirão, accompanied by all the original field records and other documentation, are in storage at the National Museum of Archaeology, in Lisbon, Portugal, which is also the authority that issues the permits for the study of the collections it owns. All necessary permits were obtained for the described study, which complied with relevant regulations. The individuals shown in Fig 2 are volunteers who gave fully informed consent for the photographs to be taken and used to illustrate the excavation work for academic purposes, including research and publication.

The 1979–88 excavation of the site followed standard practice. Finds were piece-plotted using a one square meter grid for (x,y) coordinates and an arbitrary datum, set at 133.4 m asl, for (z) elevations. The deposit was water-sieved on site, and sieve residues were lab-sorted by hand for the recovery of small finds, namely the microfaunal remains. Recognised layers were subdivided into arbitrary spits whose boundaries followed the natural dip of the stratification and whose thickness varied as a function of the deposit’s texture (5 cm as a rule, 10 to 20 cm when mostly made-up of dense accumulations of éboulis). Exceptionally, at the time of initial testing of the Solutrean and pre-Solutrean sequence in square P11 and adjacent square P12, this *modus operandi* had to be adjusted. Because of the constrained space and the significant E→W dip of the stratification, the spits had to be horizontal, which implied the risk, knowingly taken on, that 10–15 cm intervals of mixed content would be obtained whenever layer boundaries were crossed. To minimize the risk, and taking advantage of the fact that, through the Solutrean and Early Upper Palaeolithic sequence, no appreciable N→S dip existed, a protocol of systematic piece-plotting was followed, and P11 was excavated in two half-square subdivisions, E and W. This system allowed for correct assignment, via projection onto the S profile when in doubt, of the vast majority of the finds retrieved in the course of that initial, testing phase. In the Entrance trench, the natural dip of the stratification was also followed. However, the spatial disconnection with the interior sequence led, after some experimentation, to the adoption of a separate system to designate the different layers (and the spits into which they were subdivided). Eventually, based on the study of the S-P20>19 profile, it was concluded that only six main “units” could clearly be defined, and the spits/layers discriminated at the time of excavation were assigned to each such unit based on décapage plans and associated descriptions and elevations.

Sample P11-783 was radiocarbon dated at the VERA laboratory. Two subsamples, VERA-5454 and VERA-5454_2, of this specimen were treated with the laboratory’s standard pre-treatment used for bones and independently dated. The C and N content as well as the δ^13^C and δ^15^N values of the extracted gelatin, usually measured with EA-IRMS systems (elemental analyser coupled to a stable isotope ratio mass spectrometer) in sample splits of the ^14^C sample, were not determined. Further independent age determinations of two ultrafiltrated sub-samples from the P11-783 tooth were performed. In the first trial, only the <30 kDa (VERA-5454UF2) could be measured, whereas from the second ultrafiltrated subsample both the >30kDa (VERA-5454_2UF1) and the <30 kDa (VERA-5454_2UF2) fraction could be dated. The chemical procedure for the ultrafiltration is, apart from some modifications, the method described in [15, 115, 116].

The post-2010 radiocarbon dating of bone samples at the Oxford Radiocarbon Accelerator Unit was carried out following a routine acid-base-acid (coded AG) collagen extraction procedure described previously [115, 116]. Sample surfaces were initially cleaned by air abrasion with aluminium oxide powder, and *c*. 250-500mg of bone was removed for chemical pretreatment. After the AG procedure, the extracted collagen was weighed into tin capsules (*c*. 5mg) for the measurement of elemental values and stable isotope (δ^13^C and δ^15^N) values, and for CO_2_ collection using a Carlo-Erba NA 2000 combustion elemental analyser coupled with a Sercon 20/20 isotope ratio mass spectrometer. ^13^C/^12^C and ^15^N/^14^N values are reported as delta per mil values relative to international standards (VPDB and AIR respectively). 98% of the CO_2_ was diverted via a splitter for cryogenic collection and subsequent graphitisation and target production following protocols outlined in [117]. The collagen samples met a number of quality control criteria prior to AMS dating; namely a 1%wt or greater yield, a measured %C value of between 30–50%, and an elemental C:N ratio indicative of well-preserved collagen (2.9–3.5) [118]. Samples that do not meet these criteria are usually considered to have failed. Successful samples were dated using the ORAU HVEE AMS system [115]. In-house standards of background age were measured in order to assess pre-treatment backgrounds [119]. One Solutrean sample, O12-84, was reported with an OxA-X assignation. This sample was pre-treated following the routine AG protocol but exhibited a collagen yield of 0.7% and a C:N value of 3.5. As such, it should be noted that this date may be less reliable.

All bone samples (except P11-705) dated at the Oxford Radiocarbon Accelerator Unit prior to 2010 underwent continuous-flow ABA collagen extraction procedures outlined in [120]. Powdered bone samples were demineralised using a continuous-flow system and the resulting collagen gelatinised. The gelatinised collagen was then purified using BioRad AGMP-50 ion exchange resin (coded AI), or as an additional step, subsequently hydrolysed, and the amino acids further purified using BioRad 50W-X8 ion exchange resin (coded AC). Samples were then combusted, graphitised for target production and subsequently AMS dated following methods described previously [121]. The charred bone sample P11-799, dated in 1989, was pre-treated following a reduced carbon protocol (coded RR) involving mild acid and purified water washes with ultrasonication, described in detail previously [116]. The radiocarbon dating of shell samples was also carried out at the Oxford Radiocarbon Accelerator Unit, following a phosphoric acid digestion protocol for fragile carbonates (coded OX) consisting of the cleaning and removal of the outer surface by air abrasion with aluminium oxide powder, followed by rinsing with ultrapure water (using ultrasonication if required), drying and rough crushing. Approximately 20–50 mg of crushed shell was then reacted with 85% phosphoric acid and the CO_2_ collected following methods described previously [116].

Seven bones/teeth were dated at MAMS from several layers, but more were pre-treated and failed to yield collagen. The pre-treatment was carried out in the Department of Human Evolution of the Max Planck Institute for Evolutionary Anthropology, using previously described collagen extraction and ultrafiltration protocols [122, 123]. Stable isotopic (δ^13^C and δ^15^N) and elemental values (C%, N%, C:N) of the collagen extracts (*c*. 0.5 mg) were obtained using a ThermoFinnigan Flash elemental analyser (EA) coupled to a Thermo Delta plus XP isotope ratio mass spectrometer (IRMS). Stable carbon isotope ratios were expressed relative to VPDB (Vienna PeeDee Belemnite), and stable nitrogen isotope ratios were measured relative to AIR (atmospheric N_2_) using the delta notation (δ) in parts per thousand (‰). Repeated analysis of internal and international standards indicates an analytical error of 0.2‰ (1σ) for δ^13^C and δ^15^N. Samples with a collagen yield of 0.8% or higher were deemed suitable for dating as their elemental and stable isotopic values fell within accepted ranges of well-preserved collagen (Tables 5 and 8; [118]). The collagen extracts (*c*. 3–5 mg) were weighed into pre-cleaned tin cups and sent to the Klaus-Tschira-AMS facility in Mannheim (lab code: MAMS) for graphitisation and dating using the MICADAS AMS [124]. Data reduction was performed using BATS software [125]. Errors were calculated from blanks and standards measured in the same magazine, with an additional 1‰ included in the final error calculation, as per the standard practice at MAMS. A background bone (>50,000 BP) was pre-treated and dated alongside the samples to monitor lab-based contamination.

For detailed explanation of the methodology used in the OSL dating of the Back Chamber sequence and the identification of the colorants found on the site’s Early Upper Palaeolithic shell beads, see the S1 File.

## Supporting information

S1 File Additional data and information on the excavation of the site and the analytical approaches used. (PDF)

## References

[1] Zilhão J. Gruta do Caldeirão. O Neolítico Antigo. Lisboa: Instituto Português do Património Arquitectónico e Arqueológico; 1992. 326 p.

[2] Zilhão J. O Paleolítico Superior da Estremadura portuguesa. Lisboa: Colibri; 1997. 1159 p.

[3] Hahn J. Das Geissenklösterle I. Stuttgart: Konrad Theiss; 1988.

[4] Mellars PA, Bricker HM, Gowlett JJ, Hedges RM. Radiocarbon Accelerator Dating of French Upper Paleolithic Sites. Current Anthropology. 1987;28(1):128–33.

[5] Ryan WBF, Carbotte SM, Coplan JO, O’Hara S, Melkonian A, Arko R, et al. Global Multi-Resolution Topography synthesis. Geochemistry, Geophysics, Geosystems. 2009;10(3):Q03014. doi: 10.1029/2008gc002332

[6] Zilhão J. The Spread of Agro-Pastoral Economies across Mediterranean Europe: A View from the Far West. Journal of Mediterranean Archaeology. 1993;6(1):5–63. doi: 10.1558/jmea.v6i1.5

[7] Zilhão J. From the Mesolithic to the Neolithic in the Iberian Peninsula. In: Price TD, editor. Europe’s First Farmers. Cambridge: Cambridge University Press; 2000. p. 144–82.

[8] Zilhão J. Radiocarbon evidence for maritime pioneer colonization at the origins of farming in west Mediterranean Europe. Proceedings of the National Academy of Sciences of the United States of America. 2001;98(24):14180–5. doi: 10.1073/pnas.241522898 WOS:000172328100126. 11707599 PMC61188

[9] Straus LG. El Solutrense vasco-cantabrico: una nueva perspectiva. Madrid: Centro de Investigación y Museo de Altamira; 1983.

[10] Smith P. Le Solutréen en France. Bordeaux: Delmas; 1966.

[11] Delibrias G, Guillier M-T, Labeyrie J. Gif natural radiocarbon measurements X. Radiocarbon. 1986;28(1):9–68.

[12] Zilhão J. Le passage du Paléolithique moyen au Paléolithique supérieur dans le Portugal. In: Cabrera-Valdés V, editor. El orígen del hombre moderno en el Suroeste de Europa. Madrid: Universidad Nacional de Educación a Distancia; 1993. p. 127–45. doi: 10.1111/j.1365-2958.1993.tb01201.x

[13] Zilhão J. The Ebro frontier: a model for the late extinction of Iberian Neanderthals. In: Finlayson C, editor. Neanderthals on the Edge: 150th anniversary conference of the Forbes’ Quarry discovery, Gibraltar. Oxford: Oxbow Books; 2000. p. 111–21.

[14] Zilhão J. The Ebro Frontier Revisited. In: Camps M, Szmidt C, editors. The Mediterranean from 50 000 to 25 000 BP: Turning Points and New Directions. Oxford: Oxbow Books; 2009. p. 293–312.

[15] Zilhão J, Davis SJM, Duarte C, Monge Soares AM, Steier P, Wild EM. Pego do Diabo (Loures, Portugal): Dating the emergence of anatomical modernity in Westernmost Eurasia. PLoS ONE. 2010;5(1):e8880. doi: 10.1371/journal.pone.0008880 WOS:000274114800006. 20111705 PMC2811729

[16] Zilhão J, Anesin D, Aubry T, Badal E, Cabanes D, Kehl M, et al. Precise dating of the Middle-to-Upper Paleolithic transition in Murcia (Spain) supports late Neandertal persistence in Iberia. Heliyon. 2017;3(11):e00435. doi: 10.1016/j.heliyon.2017.e00435 29188235 PMC5696381

[17] Zilhão J, Pettitt P. On the new dates for Gorham’s Cave and the late survival of Iberian Neanderthals. Before Farming. 2006;2006(3):95–122. doi: 10.3828/bfarm.2006.3.3

[18] Zilhão J. Chronostratigraphy of the Middle-to-Upper Paleolithic transition in the Iberian Peninsula. Pyrenae. 2006;37:7–84.

[19] Hoffmann DL, Pike AWG, Wainer K, Zilhão J. New U-series results for the speleogenesis and the Palaeolithic archaeology of the Almonda karstic system (Torres Novas, Portugal). Quaternary International. 2013;294:168–82. doi: 10.1016/j.quaint.2012.05.027 WOS:000318379900013.

[20] d’Errico F, Zilhão J, Julien M, Baffier D, Pelegrin J. Neanderthal acculturation in Western Europe? A critical review of the evidence and its interpretation. Current Anthropology. 1998;39(S1):S1–S44. doi: 10.1086/204689

[21] Zilhão J, d’Errico F. The chronology and taphonomy of the earliest Aurignacian and its implications for the understanding of Neandertal extinction. Journal of World Prehistory. 1999;13(1):1–68. doi: 10.1023/A:1022348410845 WOS:000081862400001.

[22] Zilhão J. The Aurignacian of Portugal: a reappraisal. Zona arqueológica. 2006;7:373–94.

[23] Zilhão J, Trinkaus E, editors. Portrait of the Artist as a Child. The Gravettian Human Skeleton from the Abrigo do Lagar Velho and its Archaeological Context. Lisboa: Instituto Português de Arqueologia; 2002.

[24] Almeida F, Moreno-García M, Angelucci DE. From under the bulldozer’s claws: the EE15 Late Gravettian occupation surface of the Lagar Velho rock-shelter. World Archaeology. 2009;41(2):242–61.

[25] Zilhão J, Cardoso J, Pike A, Weninger B. Gruta Nova da Columbeira (Bombarral, Portugal): Site stratigraphy, age of the Mousterian sequence, and implications for the timing of Neanderthal extinction in Iberia. Quartär. 2011;58:93–112. doi: 10.7485/QU58_05

[26] López-García JM, Póvoas L, Zilhão J. Nota sobre la taxonomía de Microtus (Iberomys) (Arvicolinae, Rodentia) del Pleistoceno superior de la Gruta do Caldeirão (Tomar, Portugal) e interpretación paleoclimática de la asociación de roedores. Estudios Geológicos. 2020;76(1):e128. 10.3989/egeol.43622.542.

[27] Pereira T. A exploração do quartzito na faixa atlântica peninsular durante o final do Plistocénico: Universidade do Algarve; 2010.

[28] Rasmussen SO, Bigler M, Blockley SP, Blunier T, Buchardt SL, Clausen HB, et al. A stratigraphic framework for abrupt climatic changes during the Last Glacial period based on three synchronized Greenland ice-core records: refining and extending the INTIMATE event stratigraphy. Quaternary Science Reviews. 2014;106:14–28. doi: 10.1016/j.quascirev.2014.09.007

[29] Ellwood BB, Zilhão J, Harrold FB, Balsam W, Burkart B, Long GJ, et al. Identification of the last glacial maximum in the Upper Paleolithic of Portugal using magnetic susceptibility measurements of Caldeirão Cave sediments. Geoarchaeology. 1998;13(1):55–71. doi: 10.1002/(sici)1520-6548(199801)13:1&lt;55::aid-gea4&gt;3.3.co;2–8

[30] Zilhão J. New evidence from Galeria da Cisterna (Almonda) and Gruta do Caldeirão on the phasing of Central Portugal’s Early Neolithic. Open Archaeology. 2021;7(1):747–64. doi: 10.1515/opar-2020-0163

[31] Lloveras L, Moreno-García M, Nadal J, Zilhão J. Who brought in the rabbits? Taphonomical analysis of Mousterian and Solutrean leporid accumulations from Gruta do Caldeirão (Tomar, Portugal). Journal of Archaeological Science. 2011;38(9):2434–49. doi: 10.1016/j.jas.2011.05.012 WOS:000293551200042.

[32] Davis SJM, Robert I, Zilhão J. Caldeirão cave (Central Portugal)—Whose home? Hyaena, man, bearderd vulture. CFS Courier Forschungsinstitut Senckenberg 2007. p. 213–26.

[33] Davis SJM. The mammals and birds from the Gruta do Caldeirão, Portugal. Revista Portuguesa de Arqueologia. 2002;5(2):29–98.

[34] Bertran P, Texier J-P. Facies and microfacies of slope deposits. CATENA. 1999;35(2):99–121. 10.1016/S0341-8162(98)00096-4.

[35] Pericot L. La cueva del Parpalló (Gandia). Madrid: Consejo Superior de Investigaciones Cientificas; 1942.

[36] Peyrony D, Peyrony E. Laugerie-Haute près des Eyzies (Dordogne): Masson; 1938.

[37] Renard C. Continuity or discontinuity in the Late Glacial Maximum of south-western Europe: the formation of the Solutrean in France. World Archaeology. 2011;43(4):726–43.

[38] Verpoorte A, Cosgrove R, Wood R, Petchey F, Lenoble A, Chadelle J-P, et al. Improving the chronological framework for Laugerie-Haute Ouest (Dordogne, France). Journal of Archaeological Science: Reports. 2019;23:574–82. 10.1016/j.jasrep.2018.11.017.

[39] Zilhão J, Aubry T. La pointe de Vale Comprido et les origines du Solutréen. L’Anthropologie. 1995;99(1):125–42.

[40] Aubry T, Detrain L, Kervazo B. Les niveaux intermédiaires entre le Gravettien et le Solutréen de l’Abri Casserole (Les Eyzies de Tayac): Mise en évidence d’un mode de production original de microlithes et implications. Bulletin de la Société préhistorique française 1995;92(3):296–301. 10.3406/bspf.1995.10030.

[41] Reimer PJ, Austin WEN, Bard E, Bayliss A, Blackwell PG, Bronk Ramsey C, et al. The IntCal20 Northern Hemisphere Radiocarbon Age Calibration Curve (0–55 cal kBP). Radiocarbon. 2020;62(4):725–57. Epub 2020/08/12. doi: 10.1017/RDC.2020.41

[42] Heaton TJ, Köhler P, Butzin M, Bard E, Reimer RW, Austin WEN, et al. Marine20—The Marine Radiocarbon Age Calibration Curve (0–55,000 cal BP). Radiocarbon. 2020;62(4):779–820. Epub 2020/08/12. doi: 10.1017/RDC.2020.68

[43] Stuiver M, Reimer PJ. Extended 14C data base and revised CALIB 3.0 14C age calibration program. Radiocarbon. 1993;35:215–30.

[44] Almeida F, Brugal J-P, Zilhão J, Plisson H. An Upper Paleolithic Pompeii: Technology, Subsistence and Paleoethnography at Lapa do Anecrial. From the Mediterranean basin to the Portuguese Atlantic Shore: Papers in Honor of Anthony Marks Actas do IV Congresso de Arqueologia Peninsular. Promontoria Monográfica Faro 2007. p. 119–39.

[45] Zazzo A. Bone and enamel carbonate diagenesis: A radiocarbon prospective. Palaeogeography, Palaeoclimatology, Palaeoecology. 2014;416:168–78. 10.1016/j.palaeo.2014.05.006.

[46] Arnold LJ, Demuro M, Ruiz MN. Empirical insights into multi-grain averaging effects from ‘pseudo’ single-grain OSL measurements. Radiation Measurements. 2012;47(9):652–8. 10.1016/j.radmeas.2012.02.005.

[47] Duval M, Arnold LJ. Field gamma dose-rate assessment in natural sedimentary contexts using LaBr3(Ce) and NaI(Tl) probes: A comparison between the “threshold” and “windows” techniques. Applied Radiation and Isotopes. 2013;74:36–45. doi: 10.1016/j.apradiso.2012.12.006 23353090

[48] Bøtter-Jensen L, Mejdahl V. Assessment of beta dose-rate using a GM multicounter system. International Journal of Radiation Applications and Instrumentation Part D Nuclear Tracks and Radiation Measurements. 1988;14(1):187–91. 10.1016/1359-0189(88)90062-3.

[49] Mejdahl V. Thermoluminescence dating: beta-dose attenuation in quartz grains. Archaeometry. 1979;21(1):61–72. doi: 10.1111/j.1475-4754.1979.tb00241.x

[50] Brennan BJ. Beta doses to spherical grains. Radiation Measurements. 2003;37(4):299–303. doi: 10.1016/S1350-4487(03)00011-8

[51] Prescott JR, Hutton JT. Cosmic ray contributions to dose rates for luminescence and ESR dating: Large depths and long-term time variations. Radiation Measurements. 1994;23(2):497–500. doi: 10.1016/1350-4487(94)90086-8

[52] Mejdahl V. Internal radioactivity in quartz and feldspar grains. Ancient TL 1987;5(2):10–7.

[53] Bowler J, Johnston H, Olley J, R. Prescott J, G. Roberts R, Shawcross W, et al. New ages for human occupation and climatic change at Lake Mungo, Australia. Nature. 2003;421:837–40. doi: 10.1038/nature01383 12594511

[54] Jacobs Z, Duller G, Wintle AG. Interpretation of single grain De distributions and calculation of De. Radiation Measurements. 2006;41:264–77. doi: 10.1016/j.radmeas.2005.07.027

[55] Pawley SM, Bailey RM, Rose J, Moorlock BSP, Hamblin RJO, Booth SJ, et al. Age limits on Middle Pleistocene glacial sediments from OSL dating, north Norfolk, UK. Quaternary Science Reviews. 2008;27(13):1363–77. 10.1016/j.quascirev.2008.02.013.

[56] Lewis RJ, Tibby J, Arnold LJ, Barr C, Marshall J, McGregor G, et al. Insights into subtropical Australian aridity from Welsby Lagoon, north Stradbroke Island, over the past 80,000 years. Quaternary Science Reviews. 2020;234:106262. 10.1016/j.quascirev.2020.106262.

[57] Rees-Jones J. Optical dating of young sediments using fine-grain quartz. Ancient TL. 1995;13(2):9–14.

[58] Rees-Jones J, Tite MS. Optical Dating Results for British Archaeological Sediments. Archaeometry. 1997;39(1):177–87. doi: 10.1111/j.1475-4754.1997.tb00797.x

[59] Arnold LJ, Roberts RG. Stochastic modelling of multi-grain equivalent dose (De) distributions: Implications for OSL dating of sediment mixtures. Quaternary Geochronology. 2009;4:204–30. doi: 10.1016/j.quageo.2008.12.001

[60] Arnold LJ, Roberts RG, Galbraith RF, DeLong SB. A revised burial dose estimation procedure for optical dating of young and modern-age sediments. Quaternary Geochronology. 2009;4(4):306–25. doi: 10.1016/j.quageo.2009.02.017

[61] Bailey RM, Arnold LJ. Statistical modelling of single grain quartz De distributions and an assessment of procedures for estimating burial dose. Quaternary Science Reviews. 2006;25(19):2475–502. doi: 10.1016/j.quascirev.2005.09.012

[62] Arnold LJ, Roberts RG. Paper I–Optically stimulated luminescence (OSL) dating of perennially frozen deposits in north-central Siberia: OSL characteristics of quartz grains and methodological considerations regarding their suitability for dating. Boreas. 2011;40(3):389–416. 10.1111/j.1502-3885.2011.00209.x.

[63] Galbraith RF, Roberts RG, Laslett GM, Yoshida H, Olley JM. Optical dating of single and multiple grains of quartz from Jinmium rock shelter, northern Australia: Part I, Experimental design and statistical models. Archaeometry. 1999;41(2):339–64. doi: 10.1111/j.1475-4754.1999.tb00987.x

[64] Arnold LJ, Roberts RG, MacPhee RDE, Willerslev E, Tikhonov AN, Brock F. Optical dating of perennially frozen deposits associated with preserved ancient plant and animal DNA in north-central Siberia. Quaternary Geochronology. 2008;3(1–2):114–36. doi: 10.1016/j.quageo.2007.09.002

[65] Arnold LJ, Demuro M, Navazo M, Benito Calvo A, Pérez-González A. OSL dating of the Middle Paleolithic Hotel California site, Sierra de Atapuerca, north-central Spain. Boreas. 2013;42(2):285–305. doi: 10.1111/j.1502-3885.2012.00262.x

[66] Demuro M, Arnold LJ, Froese DG, Roberts RG. OSL dating of loess deposits bracketing Sheep Creek tephra beds, northwest Canada: Dim and problematic single-grain OSL characteristics and their effect on multi-grain age estimates. Quaternary Geochronology. 2013;15:67–87. doi: 10.1016/j.quageo.2012.11.003

[67] Nathan RP, Thomas PJ, Jain M, Murray AS, Rhodes EJ. Environmental dose rate heterogeneity of beta radiation and its implications for luminescence dating: Monte Carlo modelling and experimental validation. Radiation Measurements. 2003;37(4):305–13. 10.1016/S1350-4487(03)00008-8.

[68] Arnold LJ, Bailey RM, Tucker GE. Statistical treatment of fluvial dose distributions from southern Colorado arroyo deposits. Quaternary Geochronology. 2007;2(1):162–7. doi: 10.1016/j.quageo.2006.05.003

[69] Arnold LJ, Demuro M, Spooner NA, Prideaux GJ, McDowell MC, Camens AB, et al. Single-grain TT-OSL bleaching characteristics: Insights from modern analogues and OSL dating comparisons. Quaternary Geochronology. 2019;49:45–51. 10.1016/j.quageo.2018.01.004.

[70] Ruiz MN, Benito-Calvo A, Alonso-Alcalde R, Alonso P, Fuente Hdl, Santamaría M, et al. Late Neanderthal subsistence strategies and cultural traditions in the northern Iberia Peninsula: Insights from Prado Vargas, Burgos, Spain. Quaternary Science Reviews. 2021;254:106795. 10.1016/j.quascirev.2021.106795.

[71] David B, Arnold LJ, Delannoy J-J, Fresløv J, Urwin C, Petchey F, et al. Late survival of megafauna refuted for Cloggs Cave, SE Australia: Implications for the Australian Late Pleistocene megafauna extinction debate. Quaternary Science Reviews. 2021;253:106781. 10.1016/j.quascirev.2020.106781.

[72] Deschamps M, Zilhão J. Assessing site formation and assemblage integrity through stone tool refitting at Gruta da Oliveira (Almonda karst system, Torres Novas, Portugal): A Middle Paleolithic case study. PLoS ONE. 2018;13(2):e0192423. doi: 10.1371/journal.pone.0192423 29451892 PMC5815586

[73] Sivan D, Potasman M, Almogi-Labin A, Bar-Yosef Mayer DE, Spanier E, Boaretto E. The Glycymeris query along the coast and shallow shelf of Israel, southeast Mediterranean. Palaeogeography, Palaeoclimatology, Palaeoecology. 2006;233(1):134–48. 10.1016/j.palaeo.2005.09.018.

[74] Soares AMM. Variabilidade do “Upwelling” costeiro durante o Holocénico nas Margens Atlânticas Ocidental e Meridional da Península Ibérica: Universidade do Algarve; 2005.

[75] Soares AMM, Dias JMA. Coastal Upwelling and Radiocarbon—Evidence for Temporal Fluctuations in Ocean Reservoir Effect off Portugal During the Holocene. Radiocarbon. 2006;48(1):45–60. Epub 2016/07/18. doi: 10.1017/S0033822200035384

[76] Lambeck K, Rouby H, Purcell A, Sun Y, Sambridge M. Sea level and global ice volumes from the Last Glacial Maximum to the Holocene. Proceedings of the National Academy of Sciences. 2014;111(43):15296. doi: 10.1073/pnas.1411762111 25313072 PMC4217469

[77] Wood R. From revolution to convention: the past, present and future of radiocarbon dating. Journal of Archaeological Science. 2015;56:61–72. 10.1016/j.jas.2015.02.019.

[78] Zilhão J, Angelucci DE, Arnold LJ, Demuro M, Hoffmann DL, Pike AWG. A revised, Last Interglacial chronology for the Middle Palaeolithic sequence of Gruta da Oliveira (Almonda karst system, Torres Novas, Portugal). Quaternary Science Reviews. 2021;258:106885. 10.1016/j.quascirev.2021.106885.

[79] Marom A, McCullagh JSO, Higham TFG, Sinitsyn AA, Hedges REM. Single amino acid radiocarbon dating of Upper Paleolithic modern humans. Proceedings of the National Academy of Sciences. 2012;109(18):6878. doi: 10.1073/pnas.1116328109 22517758 PMC3344984

[80] Devièse T, Abrams G, Hajdinjak M, Pirson S, De Groote I, Di Modica K, et al. Reevaluating the timing of Neanderthal disappearance in Northwest Europe. Proceedings of the National Academy of Sciences. 2021;118(12):e2022466118. doi: 10.1073/pnas.2022466118 33798098 PMC7999949

[81] Haws JA, Benedetti MM, Talamo S, Bicho N, Cascalheira J, Ellis MG, et al. The early Aurignacian dispersal of modern humans into westernmost Eurasia. Proceedings of the National Academy of Sciences. 2020:202016062. doi: 10.1073/pnas.2016062117 32989161 PMC7568277

[82] Hublin J-J, Sirakov N, Aldeias V, Bailey S, Bard E, Delvigne V, et al. Initial Upper Palaeolithic Homo sapiens from Bacho Kiro Cave, Bulgaria. Nature. 2020;581(7808):299–302. doi: 10.1038/s41586-020-2259-z 32433609

[83] Weninger B, Jöris O. A 14C age calibration curve for the last 60 ka: the Greenland-Hulu U/Th timescale and its impact on understanding the Middle to Upper Paleolithic transition in Western Eurasia. Journal of Human Evolution. 2008;55(5):772–81. doi: 10.1016/j.jhevol.2008.08.017 18922563

[84] Goñi MaFS, Turon J-L, Eynaud F, Gendreau S. European Climatic Response to Millennial-Scale Changes in the Atmosphere–Ocean System during the Last Glacial Period. Quaternary Research. 2000;54(3):394–403. 10.1006/qres.2000.2176.

[85] Sánchez Goñi MF, Landais A, Cacho I, Duprat J, Rossignol L. Contrasting intrainterstadial climatic evolution between high and middle North Atlantic latitudes: A close-up of Greenland Interstadials 8 and 12. Geochemistry, Geophysics, Geosystems. 2009;10(4). 10.1029/2008GC002369.

[86] Banks WE, Zilhão J, d’Errico F, Kageyama M, Sima A, Ronchitelli A. Investigating links between ecology and bifacial tool types in Western Europe during the Last Glacial Maximum. Journal of Archaeological Science. 2009;36(12):2853–67. doi: 10.1016/j.jas.2009.09.014 WOS:000271796600028.

[87] Haws JA, Benedetti MM, Cascalheira JM, Bicho NF, Carvalho MC, Zinsious BK, et al. Human Occupation during the Late Pleniglacial at Lapa do Picareiro. In: Schmidt IC, João, Bicho N, Weniger G-C, editors. Human Adaptations to the Last Glacial Maximum: The Solutrean and its Neighbors. Newcastle upon Tyne: Cambridge Scholars Publishing; 2019. p. 188–213.

[88] Benedetti MM, Haws JA, Bicho NF, Friedl L, Ellwood BB. Late Pleistocene site formation and paleoclimate at Lapa do Picareiro, Portugal. Geoarchaeology. 2019;34(6):698–726. doi: 10.1002/gea.21735

[89] Bronk Ramsey C. Dealing with Outliers and Offsets in Radiocarbon Dating. Radiocarbon. 2009;51(3):1023–45. Epub 2016/07/18. doi: 10.1017/S0033822200034093

[90] Bronk Ramsey C. Bayesian Analysis of Radiocarbon Dates. Radiocarbon. 2009;51(1):337–60. Epub 2016/07/18. doi: 10.1017/S0033822200033865

[91] Badal E, Carrión Y, Figueiral I, Rodríguez-Ariza MO. Pinares y enebrales: El paisaje solutrense en Iberia. Espacio, Tiempo y Forma, Serie I, Prehistoria y Arqueología. 2012;5:259–71.

[92] Queiroz PF, Mateus JE, Van Leeuwaarden W. The Paleovegetational Context. Portrait of the Artist as a Child The Gravettian Human Skeleton from the Abrigo do Lagar Velho and its Archeological Context. Trabalhos de Arqueologia. Lisboa: Instituto Português de Arqueologia; 2002.

[93] Matias H, Aubry T, Zilhão J. Raw-material provenience of the Solutrean diagnostics from Gruta do Caldeirão (Tomar, Portugal). In: Schmidt IC, João; Bicho Nuno F.; Weniger Gerd-Christian, editor. Human Adaptations to the Last Glacial Maximum: The Solutrean and its Neighbors. Newcastle: Cambridge Scholars Publishing; 2019. p. 302–16.

[94] Trinkaus E, Bailey SE, Zilhão J. Upper Paleolithic human remains from the Gruta do Caldeirão, Tomar, Portugal. Revista Portuguesa de Arqueologia. 2001;4(2):5–17.

[95] Aubry T, Zilhão J. Entre Atlantique et Méditerranée. Origine et modèle évolutif du Solutréen du Portugal. In: Otte M, editor. Les Solutréens. Paris: Éditions Errance; 2018. p. 57–70.

[96] Straus LG. El Paleolítico Superior de la península ibérica. Trabajos de Prehistoria. 2018;75(1):9–51. doi: 10.3989/tp.2018.12202

[97] Straus LG, Bicho N, Winegardner AC. The Upper Palaeolithic settlement of Iberia: first-generation maps. Antiquity. 2000;74(285):553–66. Epub 2015/01/02. doi: 10.1017/S0003598X00059913

[98] Zilhão J, Almeida F. The Archaeological Framework. In: Zilhão J, Trinkaus E, editors. Portrait of the Artist as a Child The Gravettian Human Skeleton from the Abrigo do Lagar Velho and its Archeological Context. Trabalhos de Arqueologia. Lisboa: Instituto Português de Arqueologia; 2002. p. 13–27.

[99] Cortés-Sánchez M, Jiménez-Espejo FJ, Simón-Vallejo MD, Stringer C, Lozano-Francisco MC, García-Alix A, et al. An early Aurignacian arrival in southwestern Europe. Nature Ecology & Evolution. 2019;3(2):207–12. doi: 10.1038/s41559-018-0753-6 30664696

[100] Bard E, Heaton TJ, Talamo S, Kromer B, Reimer RW, Reimer PJ. Extended dilation of the radiocarbon time scale between 40,000 and 48,000 y BP and the overlap between Neanderthals and &lt;em&gt;Homo sapiens&lt;/em&gt. Proceedings of the National Academy of Sciences. 2020;117(35):21005. doi: 10.1073/pnas.2012307117 32817536 PMC7474600

[101] Aubry T, Dimuccio LA, Barbosa AF, Luís L, Santos AT, Silvestre M, et al. Timing of the Middle-to-Upper Palaeolithic transition in the Iberian inland (Cardina-Salto do Boi, Côa Valley, Portugal). Quaternary Research. 2020:1–21. Epub 2020/06/19. doi: 10.1017/qua.2020.43

[102] Cortés-Sánchez M. Cueva Bajondillo (Torremolinos). Secuencia cronocultural y paleoambiental del Cuaternario reciente en la Bahía de Málaga. Málaga: Centro de Ediciones de la Diputación Provincial de Málaga; 2007. 544 p.

[103] de la Peña P. Dating on its own cannot resolve hominin occupation patterns. Nature Ecology & Evolution. 2019;3(5):712. doi: 10.1038/s41559-019-0886-2 30988497

[104] Anderson L, Reynolds N, Teyssandier N. No reliable evidence for a very early Aurignacian in Southern Iberia. Nature Ecology & Evolution. 2019;3(5):713. doi: 10.1038/s41559-019-0885-3 30988496

[105] Zilhão J. The late persistence of the Middle Palaeolithic and Neandertals in Iberia: A review of the evidence for and against the “Ebro Frontier” model. Quaternary Science Reviews. 2021;270:107098. 10.1016/j.quascirev.2021.107098.

[106] Villaverde V, Sanchis A, Badal E, Bel MÁ, Bergadà MM, Eixea A, et al. Cova de les Malladetes (Valencia, Spain): New Insights About the Early Upper Palaeolithic in the Mediterranean Basin of the Iberian Peninsula. Journal of Paleolithic Archaeology. 2021;4(1):5. doi: 10.1007/s41982-021-00081-w

[107] Fletcher WJ, Sánchez Goñi MF, Allen JRM, Cheddadi R, Combourieu-Nebout N, Huntley B, et al. Millennial-scale variability during the last glacial in vegetation records from Europe. Quaternary Science Reviews. 2010;29(21):2839–64. 10.1016/j.quascirev.2009.11.015.

[108] Fletcher WJ, Sánchez Goñi MF. Orbital- and sub-orbital-scale climate impacts on vegetation of the western Mediterranean basin over the last 48,000 yr. Quaternary Research. 2008;70(3):451–64. 10.1016/j.yqres.2008.07.002.

[109] Sánchez-Goñi MF, Landais A, Fletcher WJ, Naughton F, Desprat S, Duprat J. Contrasting impacts of Dansgaard–Oeschger events over a western European latitudinal transect modulated by orbital parameters. Quaternary Science Reviews. 2008;27(11):1136–51. 10.1016/j.quascirev.2008.03.003.

[110] Sepulchre P, Ramstein G, Kageyama M, Vanhaeren M, Krinner G, Sánchez-Goñi M-F, et al. H4 abrupt event and late Neanderthal presence in Iberia. Earth and Planetary Science Letters. 2007;258(1):283–92. 10.1016/j.epsl.2007.03.041.

[111] Wood RE, Barroso-Ruíz C, Caparrós M, Jordá-Pardo JF, Galván-Santos B, Higham TF. Radiocarbon dating casts doubt on the late chronology of the Middle to Upper Palaeolithic transition in southern Iberia. Proceedings of the National Academy of Sciences. 2013;110(8):2781–6. doi: 10.1073/pnas.1207656110 23382220 PMC3581959

[112] Galván B, Hernández CM, Mallol C, Mercier N, Sistiaga A, Soler V. New evidence of early Neanderthal disappearance in the Iberian Peninsula. Journal of Human Evolution. 2014;75:16–27. doi: 10.1016/j.jhevol.2014.06.002 25016565

[113] Banks WE, d’Errico F, Zilhão J. Human–climate interaction during the Early Upper Paleolithic: testing the hypothesis of an adaptive shift between the Proto-Aurignacian and the Early Aurignacian. Journal of Human Evolution. 2013;64(1):39–55. doi: 10.1016/j.jhevol.2012.10.001 23245623

[114] Banks WE, d’Errico F, Zilhão J. Revisiting the chronology of the Proto-Aurignacian and the Early Aurignacian in Europe: A reply to Higham et al.’s comments on Banks et al. (2013). Journal of Human Evolution. 2013;65(6):810–7. doi: 10.1016/j.jhevol.2013.08.004 WOS:000328721100011. 24095637

[115] Bronk Ramsey C, Higham T, Leach P. Towards High-Precision AMS: Progress and Limitations. Radiocarbon. 2004;46(1):17–24. Epub 2016/07/18. doi: 10.1017/S0033822200039308

[116] Brock F, Higham T, Ditchfield P, Ramsey CB. Current Pretreatment Methods for AMS Radiocarbon Dating at the Oxford Radiocarbon Accelerator Unit (ORAU). Radiocarbon. 2010;52(1):103–12. doi: 10.1017/S0033822200045069

[117] Dee M, Bronk Ramsey C. Refinement of graphite target production at ORAU. Nuclear Instruments and Methods in Physics Research Section B: Beam Interactions with Materials and Atoms. 2000;172(1):449–53. 10.1016/S0168-583X(00)00337-2.

[118] van Klinken GJ. Bone Collagen Quality Indicators for Palaeodietary and Radiocarbon Measurements. Journal of Archaeological Science. 1999;26(6):687–95. 10.1006/jasc.1998.0385.

[119] Wood RE, Ramsey CB, Higham TFG. Refining Background Corrections for Radiocarbon Dating of Bone Collagen at Orau. Radiocarbon. 2010;52(2):600–11. Epub 2016/07/18. doi: 10.1017/S003382220004563X

[120] Law IA, Hedges REM. A Semi-Automated Bone Pretreatment System and the Pretreatment of Older and Contaminated Samples. Radiocarbon. 1989;31(3):247–53. Epub 2016/07/18. doi: 10.1017/S0033822200011759

[121] Hedges REM, Law IA, Bronk CR, Housley RA. THE OXFORD ACCELERATOR MASS SPECTROMETRY FACILITY: TECHNICAL DEVELOPMENTS IN ROUTINE DATING. Archaeometry. 1989;31(2):99–113. 10.1111/j.1475-4754.1989.tb01007.x.

[122] Fewlass H, Tuna T, Fagault Y, Hublin JJ, Kromer B, Bard E, et al. Pretreatment and gaseous radiocarbon dating of 40–100 mg archaeological bone. Scientific Reports. 2019;9(1):5342. doi: 10.1038/s41598-019-41557-8 30926822 PMC6440986

[123] Talamo S, Richards M. A Comparison of Bone Pretreatment Methods for AMS Dating of Samples >30,000 BP. Radiocarbon. 2011;53(3):443–9. Epub 2016/07/18. doi: 10.1017/S0033822200034573

[124] Kromer B, Lindauer S, Synal H-A, Wacker L. MAMS–A new AMS facility at the Curt-Engelhorn-Centre for Achaeometry, Mannheim, Germany. Nuclear Instruments and Methods in Physics Research Section B: Beam Interactions with Materials and Atoms. 2013;294:11–3. 10.1016/j.nimb.2012.01.015.

[125] Wacker L, Christl M, Synal HA. Bats: A new tool for AMS data reduction. Nuclear Instruments and Methods in Physics Research Section B: Beam Interactions with Materials and Atoms. 2010;268(7):976–9. 10.1016/j.nimb.2009.10.078.

