# Supplementary material for: Revisiting the Middle and Upper Palaeolithic archaeology of Gruta do Caldeirão (Tomar, Portugal)
Source: PLoS One. 2021 Oct 27;16(10):e0259089. doi: 10.1371/journal.pone.0259089 (PMC8550450; doi:10.1371/journal.pone.0259089)
Supplement: S1 File — (PDF) [file pone.0259089.s001.pdf]

Revisiting the Middle and Upper Palaeolithic archaeology of Gruta do Caldeirão (Tomar, Portugal)

João ZILHÃO
Diego E. ANGELUCCI
Lee J. ARNOLD
Francesco d'ERRICO
Laure DAYET
Martina DEMURO
Marianne DESCHAMPS
Helen FEWLASS
Luís GOMES
Beth LINSCOTT
Henrique MATIAS
Alistair W. G. PIKE
Peter STEIER
Sahra TALAMO
Eva M. WILD

Supplementary Information

Luminescence dating experimental procedures and results

Pigment analysis procedures and results

Stratigraphic provenance of key finds

Bayesian age modelling of the succession

Bayesian model CQL code

Luminescence dating experimental procedures and results

Procedure

Luminescence dating has been used to provide direct estimates of when the Gruta do Caldeirão infill deposits were last exposed to light prior to burial. In this study, we have focussed on optically stimulated luminescence (OSL) dating of quartz because of the abundance of this mineral type at the site and the expected Late Pleistocene age range of the archaeological deposits (Zilhão et al., 1997). Our luminescence dating study employs single-grain quartz OSL analyses rather than conventional (multi-grain) OSL measurements in order to gain improved insights into any potential methodological complications that could affect dating reliability in this cave setting; particularly the presence of insufficiently bleached grain populations (e.g., Arnold et al., 2007, 2009), contaminant grains associated with syn- or post-depositional mixing (e.g., Arnold et al., 2011, 2013, 2019), or aberrant grains displaying inherently unsuitable luminescence properties (e.g., Demuro et al., 2008, 2013).

Sample collection and preparation

In total, five OSL dating samples were collected from the sedimentary infill sequence exposed in the back chamber (square P11) of Gruta do Caldeirão. The units sampled encompass the Middle Palaeolithic to Upper Palaeolithic transition, as well as the underlying Middle Palaeolithic deposits. One sample was collected from each of the following units: unit K (sample CLD17-1), unit L (sample CLD17-2), unit M (sample CLD17-3), unit N (sample CLD17-4) and unit O (sample CLD17-5). During sampling, care was taken to avoid areas showing sediment heterogeneity and bioturbation, focusing instead on sections of homogeneous, fine grained deposits that were unaffected by post-depositional disturbance. Owing to the consolidated nature of the target sedimentary horizons, OSL samples were carefully hand-collected from cleaned, refreshed profiles under filtered red LED lighting after sealing off the cave chamber from external light contamination. Upon extraction, the hand-collected samples were immediately sealed with black plastic and duct tape to avoid exposure to daylight. Additional bulk sediment was collected from the surrounding 1 cm of each OSL sample position for water content analysis and beta dosimetry evaluation.

Purified coarse-grained quartz fractions were extracted from the luminescence samples under safe light (dim red LED) conditions at the University of Adelaide and prepared for burial dose estimation using standard preparation procedures (Aitken, 1998). The sediment samples were initially sieved to isolate the fine sand fraction (90-300 µm). Organics and carbonates were then eliminated using concentrated (30%) hydrogen peroxide (H_2O_2) and hydrochloric (HCl) acid digestion. Quartz grains were isolated using heavy liquid (LST lithium heteropolytungstate) density ranges of 2.62 g/cm^3 to 2.72 g/cm^3. The 212-250 µm quartz fractions were then sieved and etched with hydrofluoric (HF) acid to remove the alpha-irradiated external layers (48% HF digestion for 40 min). The etched grains were subsequently washed in 30% hydrochloric acid to remove any precipitated fluorides and re-sieved using a 63 µm sieve to eliminate any disaggregated grains.

Dose rate estimation

Environmental dose rates have been calculated using a combination of low-level beta counting and *in situ* gamma spectrometry (**Table 6**). Field gamma spectrometry measurements were made with a Canberra NaI:Tl detector and analysed using the 'energy windows' method to determine individual K, U and Th elemental concentrations (Arnold et al., 2012a; Duval and Arnold, 2013). External beta dose rates have been calculated from measurements made on a Risø GM-25-5 beta counter, using homogenised sediment sub-samples collected from the main

luminescence dating sample positions. The conversion factors of Guérin et al. (2011) have been used to derive gamma and beta dose rates from the measured radionuclide concentrations and specific activities. Cosmic-ray dose rates have been calculated as described in Prescott and Hutton (1994) after taking into consideration site altitude, geomagnetic latitude, and density, thickness and geometry of sediment and bedrock overburden. The beta, gamma and cosmic-ray dose rates have been corrected for long-term sediment moisture contents (Aitken, 1985; Readhead, 1987), which are taken to be equivalent to the present-day measured water contents (i.e., 10-12% of dry sediment weight) as the cave environment has remained sufficiently well-protected from major variations in external atmospheric conditions. A relative uncertainty of 20% has been assigned to the long-term water content values to accommodate any minor variations in hydrologic conditions during burial.

High-resolution gamma spectrometry (HRGS) measurements were additionally made on the homogenised bulk sediment samples to assess the presence of secular equilibrium in the ^{238}U and ^{232}Th decay series (**Table A**). Daughter-parent isotopic ratios for ^{238}U, ^{226}Ra, ^{210}Pb, ^{228}Ra and ^{228}Th are consistent with unity at either 1σ or 2σ for all samples, confirming that the ^{238}U and ^{232}Th decay series are in equilibrium. **Table A** also includes the corresponding beta dose rates obtained using the HRGS results, which have been calculated after taking into consideration the fractional beta dose rate contributions of different isotopes in the ^{238}U decay series (Stokes et al., 2003; Guérin et al., 211). For all five samples, the final beta dose rates derived using HRGS are in agreement, at either 1 or 2σ, with those obtained using beta counting (**Table 6**).

Equivalent dose (D_e) estimation

Multi-grain and single-grain OSL measurements have been made using a Risø TL/OSL-DA-20 reader equipped with blue LEDs (470 nm, maximum power 102 mW/cm^2), infrared LEDs (peak emission 850 nm, maximum power of 302 mW/cm^2), and a 10 mW Nd:YVO_4 (532 nm) single-grain laser attachment (maximum power of *c.* 50 W/cm^2). Ultraviolet OSL signals were detected using Electron Tubes PDM 9107B photomultiplier tubes fitted with 7.5 mm-thick Hoya U-340 filters. Samples were irradiated with a mounted $^{90}Sr/^{90}Y$ beta source that had been calibrated to administer known doses to multi-grain aliquots and single-grain discs (average single-grain dose rate at the time of measurement = 0.095 Gy/s). Multi-grain OSL measurements (used for dose recovery tests only; see below) were made by mounting monolayers of quartz grains on 9.7 mm stainless steel discs using silicon oil spray (Silkospray). Single-grain D_e measurements were made by manually loading individual 212-250 µm grains onto standard single-grain aluminium discs drilled with a 10 x 10 array of 300 µm diameter holes to ensure true single-grain resolution during D_e evaluation (Arnold et al., 2012b).

Multi-grain dose recovery tests and single-grain D_e measurements were undertaken using modified versions of the single-aliquot regenerative-dose (SAR) protocol described in Murray and Wintle (2000), as shown in **Table B**. Multi-grain D_e values measured as part of the dose recovery test experiments were calculated by integrating the first 0.4 s of stimulation and subtracting a late-light background from the last 10 s. Single-grain OSL D_e values were calculated by integrating the first 0.09 s of stimulation and subtracting a late-light background from the last 0.25 s. The sensitivity-corrected SAR dose-response curves were fitted with a single saturating exponential function. The uncertainty ranges of each individual D_e value include three sources of error: (i) a random uncertainty term arising from photon counting statistics for each OSL measurement, calculated using Eq. 3 of Galbraith (2002); (ii) an empirically determined instrument reproducibility uncertainty of 1.9% for each single-grain measurement (calculated for the specific Risø reader used in this study according to the

approach outlined in Jacobs et al., 2006); and (iii) a dose-response curve fitting uncertainty determined using 1000 iterations of the Monte Carlo method described by Duller (2007) and implemented in Analyst.

Single-grain OSL D_e values were excluded from final age calculations when: (i) the net intensity of the natural test dose signal, T_n, was not >3σ above the late-light background signal; (ii) the low-dose (plus high-dose in the case of single-grain OSL) recycling ratios (i.e., sensitivity-corrected luminescence responses (L_x/T_x) for two identical regenerative doses) were not consistent with unity at 2σ; (iii) the OSL IR depletion ratio of Duller (2003) was not consistent with unity at 2σ (i.e., the ratio of the L_x/T_x values obtained for two identical regenerative doses measured with and without prior IR stimulation, designed to detect feldspar contamination or inclusions); (iv) the recuperation ratio, calculated as the ratio of the sensitivity-corrected 0 Gy dose point (L_0/T_x) to the sensitivity-corrected natural (L_n/T_n), was >5%; (v) the net T_n signal had a relative error of >30%; (vi) the sensitivity-corrected natural signal (L_n/T_n) did not intercept the sensitivity-corrected dose-response curve; (vii) the dose-response curve displayed anomalous properties (i.e., zero or negative response with increasing dose) or very scattered L_x/T_x values that could not be successfully fitted with the Monte Carlo procedure and, hence, did not yield finite D_e values and uncertainty ranges; (viii) the L_n/T_n value intercepted the saturated part of the dose-response curve (L_n/T_n values were equal to the I_{max} saturation limit of the dose-response curve at 2σ).

SAR D_e validation tests

Multi-grain dose recovery tests were initially undertaken on 160-grain aliquots of sample CLD17-2 to assess the suitability of the SAR protocol and determine optimal preheat combination for bulk grain fractions of the Caldeirão samples. Five batches of four aliquots were each bleached twice at room temperature for 1000 s using blue LEDs (with an intervening 10,000 s pause to ensure complete decay of any phototransferred charge in the 110 °C TL trap), after which a known laboratory dose of 50 Gy was administered using the calibrated beta source. The surrogate natural dose of each aliquot was then measured using protocol A shown in **Table B.** A series of different preheat combinations were applied to each batch of four aliquots, as follows: regenerative-dose preheat (PH1) of 200, 220, 240 or 260°C for 10 s in combination with a test-dose preheat (PH2) of 160°C for 10 s; and a PH1 of 220°C for 10 s in combination with a PH2 of 200°C for 10 s. The two preheat combinations that produced multi-grain dose recovery ratios closest to unity (PH1 = 200°C for 10 s and PH2 = 160°C for 10 s; PH1 = 240°C for 10 s and PH2 160°C for 10 s) (**Fig A, panel A**) were further tested via single-grain dose recovery tests. These additional dose recovery tests were performed on 200-300 quartz grains of sample CLD17-2 (**Table C**) after bleaching their natural signals using the same procedure described above and administering a surrogate natural dose of 75 Gy. The single-grain OSL dose recovery test results indicate that a PH2 of 240 °C for 10 s and PH1 of 160°C for 10 s is optimal for single-grain OSL burial dose estimation. This preheat combination yielded a mean measured-to-given dose ratio of 1.00 ± 0.02 (*n* = 55 accepted grains) and an overdispersion value of 10 ± 4% (**Fig B, panel A**).

Results

Between 800 and 1100 grains per sample were measured for single-grain OSL D_e estimation. Approximately 50% of measured grains produced detectable OSL signals (**Table C**), with the brightest 1-5% of grains having 150-14,000 net counts / Gy in the first 0.09 s stimulation (**Fig C, panel A**). The measured OSL signals were bright and fast-decaying, and they were generally

depleted by *c.* 90% within the first 0.25 s of stimulation. After applying the SAR rejection criteria, 11-16% of the measured grains were considered suitable for D_e estimation (**Table C**). An example of a sensitivity-corrected dose-response and OSL decay curve for a moderately bright grain that passed the rejection criteria is shown in **Fig C, panel B**.

D_e distributions and the final single-grain OSL ages are presented and discussed in the Main Text (**section 2.1.4.**, **Fig 16**, and **Table 6**) and **Table D**.

References

Aitken, M.J., 1985. Thermoluminescence dating. Academic Press, London.

Aitken, M.J., 1998. An introduction to optical dating: the dating of Quaternary sediments by the use of photon-stimulated luminescence. Oxford University Press, Oxford.

Arnold, L.J., Bailey, R.M., Tucker, G.E., 2007. Statistical treatment of fluvial dose distributions from southern Colorado arroyo deposits. Quaternary Geochronology 2, 162-167.

Arnold, L.J., Roberts, R.G., Galbraith, R.F., DeLong, S.B., 2009. A revised burial dose estimation for optical dating of young and modern-age sediments. Quaternary Geochronology 4, 306-325.

Arnold, L.J., Roberts, R.G., 2009. Stochastic modelling of multi-grain equivalent dose (D_e) distributions: Implications for OSL dating of sediment mixtures. Quaternary Geochronology 4, 204-230.

Arnold, L.J., Roberts, R.G., 2011. Paper I – Optically stimulated luminescence (OSL) dating of perennially frozen deposits in north-central Siberia: OSL characteristics of quartz grains and methodological considerations regarding their suitability for dating. Boreas 40, 389-416.

Arnold, L.J., Duval, M., Falguères, C., Bahain, J.-J., Demuro, M. 2012a. Portable gamma spectrometry with cerium-doped lanthanum bromide scintillators: Suitability assessments for luminescence and electron spin resonance dating applications. Radiation Measurements 47, 6-18.

Arnold, L.J., Demuro, M., Navazo, M., 2012b. Empirical insights into multi-averaging effects from 'pseudo' single-grain OSL measurements. Radiation Measurements 47, 652-658.

Arnold, L. J., Roberts, R. G., MacPhee, R. D. E., Haile, J. S., Brock, F., Möller, P., Froese, D. G., Tikhonov, A. N., Chivas, A. R., Gilbert, M. T. P., Willerslev, E. 2011. Paper II – Dirt, dates and DNA: OSL and radiocarbon chronologies of perennially frozen sediments in Siberia and their implications for sedimentary ancient DNA studies. Boreas 40, 417-445.

Arnold, L.J., Demuro, M., Navazo, M., Benito-Calvo, A., Pérez-González, A., 2013. OSL dating of Middle Palaeolithic Hotel California site, Sierra de Atapuerca, north-central Spain. Boreas 42, 285-305.

Arnold, L.J., Demuro, M., Spooner, N.A., Prideaux, G.J., McDowell, M.C., Camens, A.B., Reed, E.H., Parés, J.M., Arsuaga, J.L., Bermúdez de Castro, J.M., Carbonell, E., 2019. Single-grain TT-OSL bleaching characteristics: Insights from modern analogues and OSL dating comparisons. Quaternary Geochronology, 49, 45-51.

Bailey, R.M., Arnold, L.J., 2006. Statistical modelling of single grain quartz D_e distributions and an assessment of procedures for estimating burial dose. Quaternary Science Reviews 25, 2475-2502.

Demuro, M., Roberts, R.G., Froese, D.G., Arnold, L.J., Brock, F., Bronk Ramsey, C., 2008. Optically stimulated luminescence dating of single and multiple grains of quartz from perennially frozen loess in western Yukon Territory, Canada: comparison with radiocarbon chronologies for the late Pleistocene Dawson tephra. Quaternary Geochronology 3, 346-364.

Demuro, M., Arnold, L.J., Froese, D.G., Roberts, R.G., 2013. OSL dating of loess bracketing Sheep Creek tephra beds, northwest Canada: dim and problematic single-grain OSL characteristics and their effect on multi-grain age estimates. Quaternary Geochronology 15, 67-87.

Duller, G.A.T., 2003. Distinguishing quartz and feldspar in single grain luminescence measurements. Radiation Measurements 37, 161-165.

Duller, G.A.T., 2007. Assessing the error on equivalent dose estimates derived from single aliquot regenerative dose measurements. Ancient TL 25, 15-24.

Duval, M., Arnold, L., 2013. Field gamma dose-rate assessment in natural sedimentary contexts using $LaBr_3$(Ce) and NaI(Tl) probes: a comparison between the "threshold" and "windows" techniques. Applied Radiation and Isotopes 74, 36-45

Galbraith, R.F., Roberts, R.G., Laslett, G.M., Yoshida, H., Olley, J.M., 1999. Optical dating of single and multiple grains of quartz from Jinmium rock shelter, northern Australia: Part I, experimental design and statistical models. Archaeometry 41, 339-364.

Galbraith, R., 2002. A note on the variance of a background-corrected OSL count. Ancient TL 20, 49-51.

Guérin, G., Mercier, M., Adamiec, G., 2011. Dose-rate conversion factors: update. Ancient TL 29, 5-8.

Jacobs, Z., Duller, G.A.T., Wintle, A.G., 2006. Interpretation of single grain D_e distributions and calculation of D_e. Radiation Measurements 41, 264-277.

Murray, A.S., Wintle, A., 2000. Luminescence dating of quartz using an improved single-aliquot regenerative-dose protocol. Radiation Measurements 32, 57-73.

Prescott, J.R., Hutton, J.T., 1994. Cosmic ray contributions to dose rates for luminescence and ESR dating: large depths and long-term time variations. Radiation Measurements 23, 497-500.

Readhead, M.L., 2002. Absorbed dose fraction for ^{87}Rb β particles. Ancient TL 20, 25-28.

Stokes, S., Ingram, S., Aitken, M.J., Sirocko, F., Anderson, R., Leuschner, D., 2003. Alternative chronologies for Late Quaternary (Last Interglacial-Holocene) deep sea sediments via optical dating of silt-sized quartz. Quaternary Science Reviews 22, 925-941.

Zilhão J., 1997. O Paleolítico Superior da Estremadura portuguesa. Lisboa: Colibri, 1159 p.

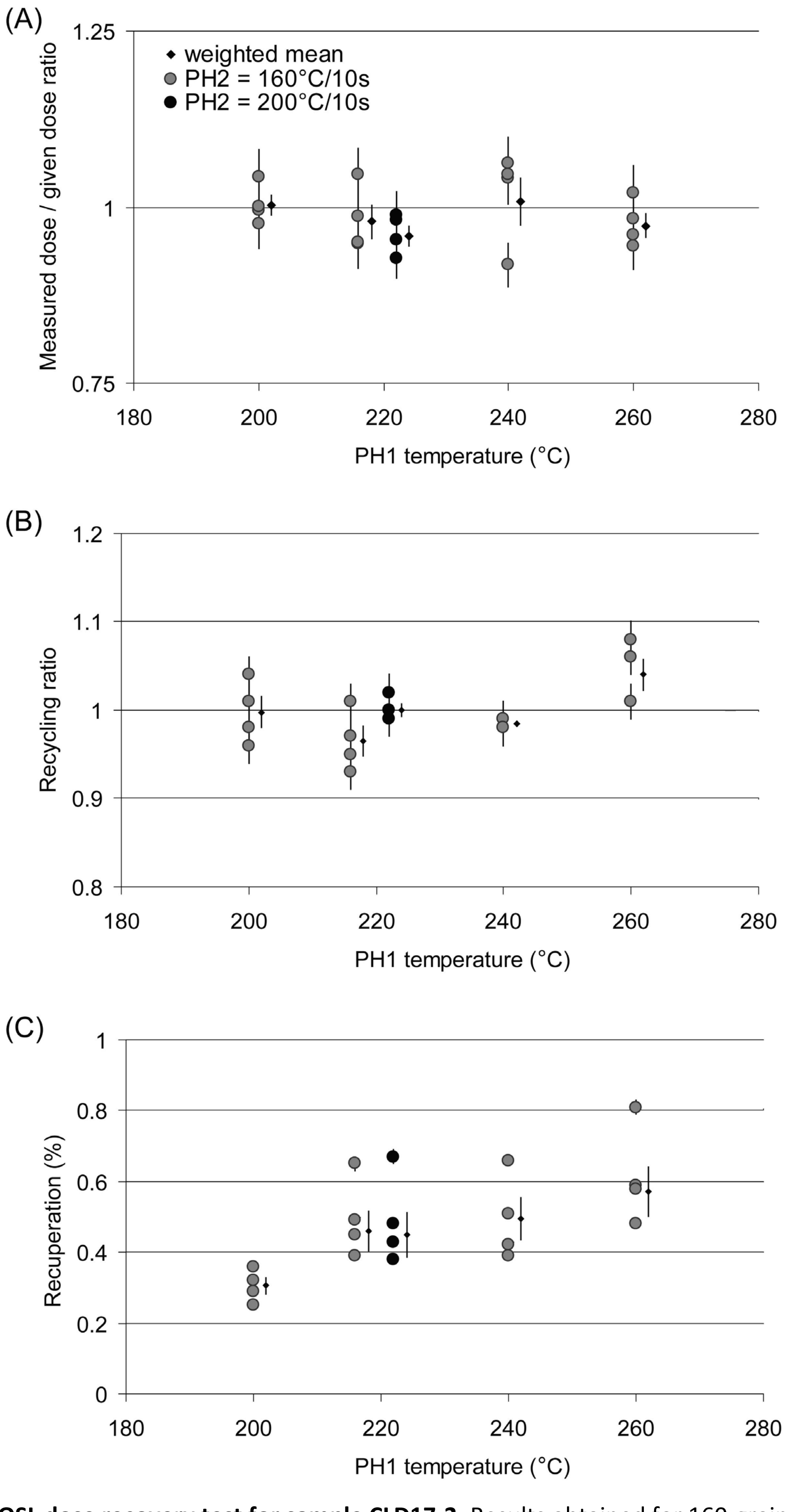

Fig A. OSL dose recovery test for sample CLD17-2. Results obtained for 160-grain aliquots using the SAR protocol A in **Table C**. Showing measured to given dose ratios (**A**), recycling ratios (**B**) and recuperation values (**C**). A known dose of 50 Gy was administered to each aliquot as part of these multi-grain dose recovery tests.

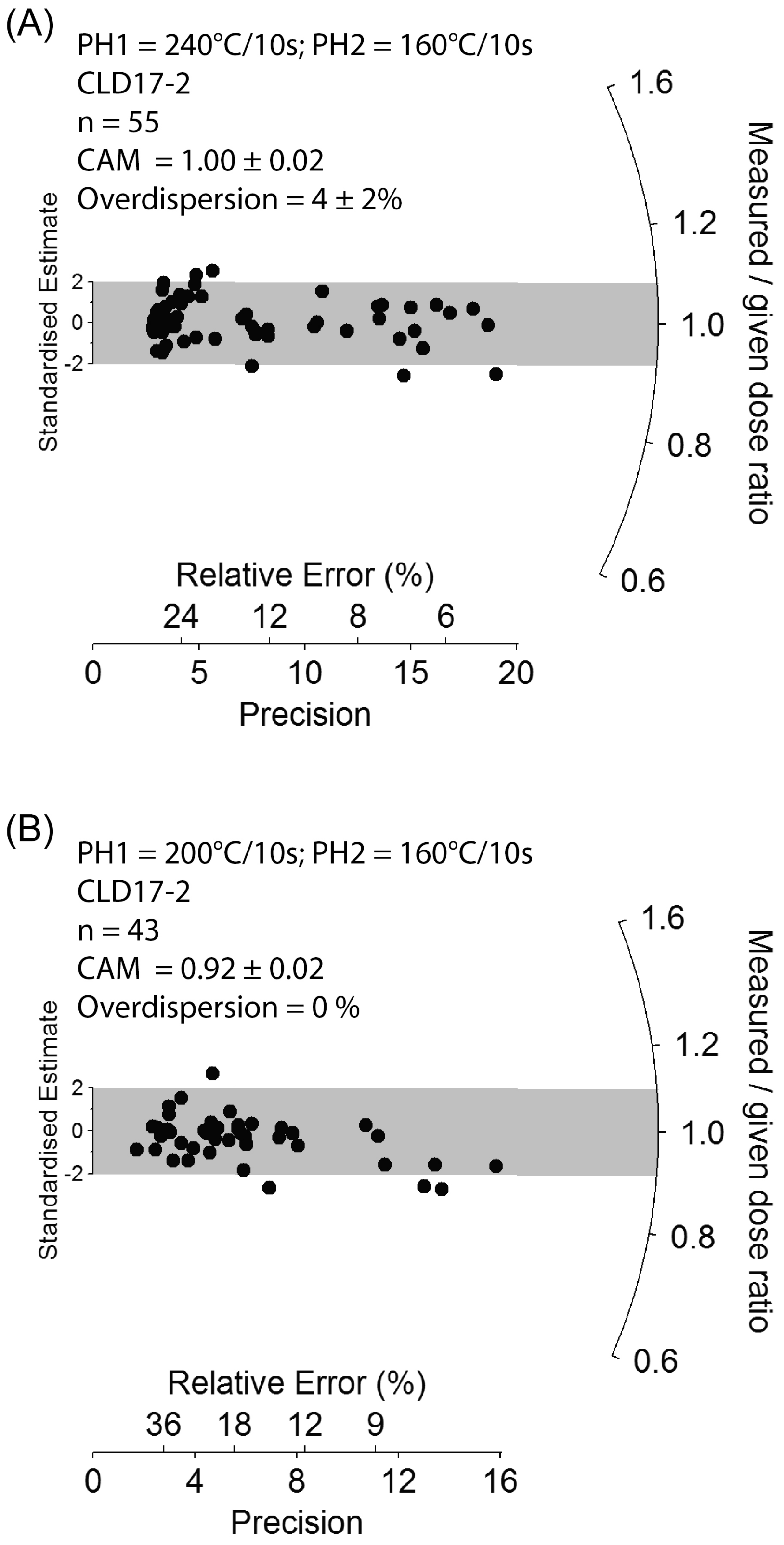

Fig B. Radial plot showing single-grain OSL dose recovery test results obtained sample CLD17-2 using the SAR protocol B in Table B. The grey shaded region is centred on a dose recovery ratio of 1. Individual values that fall within the shaded region are consistent with the administered dose at 2σ. **(A)** Data obtained using a regenerative-dose preheat (PH1) of 240°C / 10 s and test-dose preheat (PH2) of 160°C / 10 s. **(B)** Data obtained using a PH1 of 200°C / 10 s and PH2 of 160°C / 10 s.

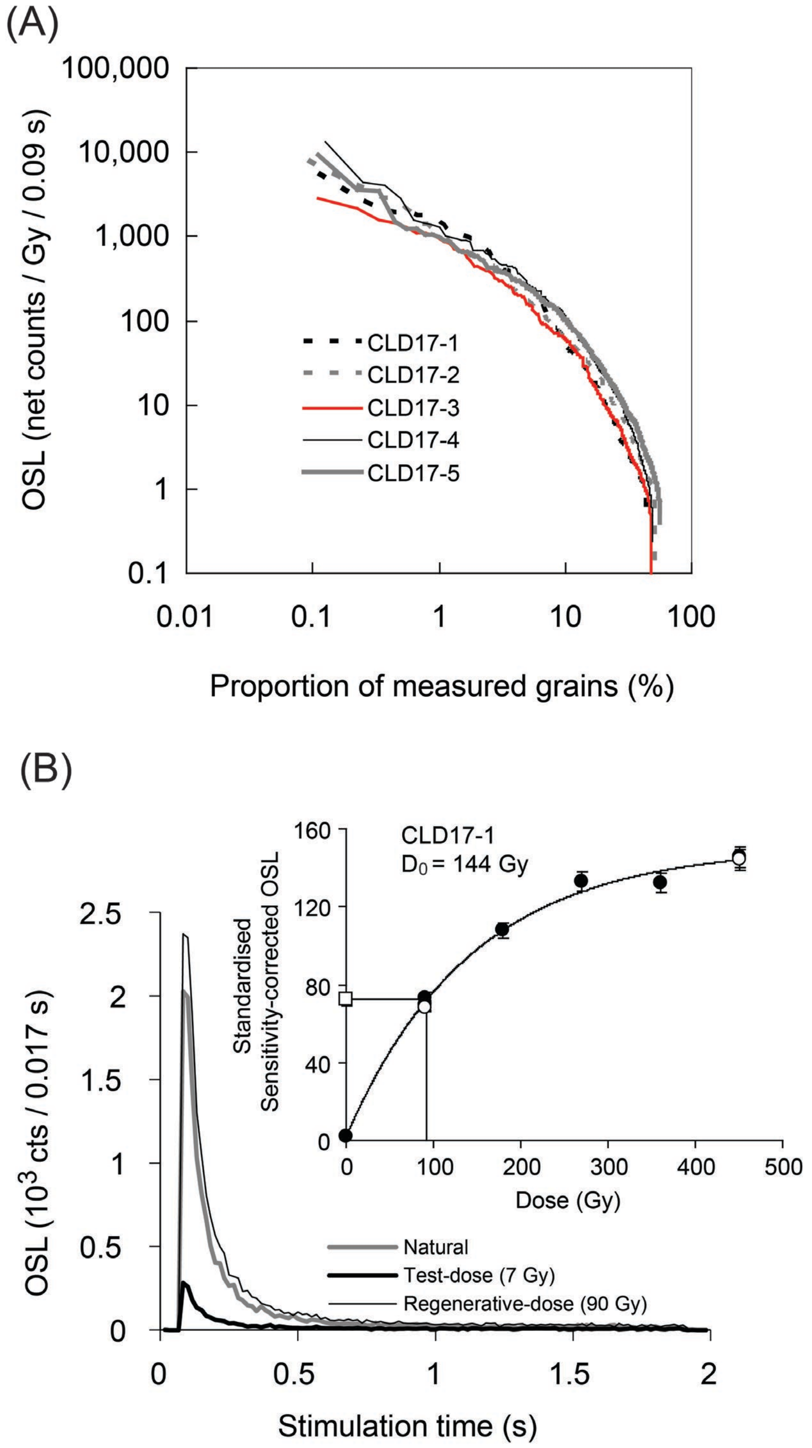

Fig C. OSL signal brightness and decay curves. (**A**) OSL signal brightness plot showing absolute net intensities expressed as counts / Gy / 0.09 s. The data shown are for single-grain OSL measurements made using the 212-250 µm quartz fraction for all samples from Gruta do Caldeirão. (**B**) Examples of OSL decay curves and sensitivity-corrected dose-response curve (inset) for a representative quartz grain of sample CLD17-1. White square denotes the sensitivity-corrected natural OSL signal; filled circles denote the sensitivity-corrected regenerative dose OSL signals; white circles denote the repeated regenerative dose points used to calculate the recycling ratios. The D_0 value characterises the rate of signal saturation with respect to administered dose and equates to the dose value for which the saturating exponential dose-response curve slope is 1/*e* (or *c.* 0.37) of its initial value.

Table A. High-resolution gamma spectrometry (HRGS). Results for OSL samples collected from Gruta do Caldeirão. The beta dose rates shown in the final column have been calculated using the long-term water content corrections, beta attenuation factors and dose rate conversation factors detailed for each sample in **Table 6**

Sample	Radionuclide specific activities (Bq/kg) [a, b]						Daughter:parent isotopic ratio			Beta dose rate (Gy/ka
	^{238}U	^{226}Ra	^{210}Pb	^{228}Ra	^{228}Th	^{40}K	^{226}Ra:^{238}U	^{210}Pb:^{226}Ra	^{228}Th:^{228}Ra	
CLD17-1	35.76 ± 3.40	34.24 ± 0.73	33.46 ± 3.79	44.13 ± 1.53	46.76 ± 1.21	380 ± 12	0.96 ± 0.09	0.98 ± 0.11	1.06 ± 0.05	1.29 ± 0.06
CLD17-2	39.21 ± 3.18	44.58 ± 0.79	45.13 ± 4.01	65.52 ± 1.58	65.48 ± 1.43	494 ± 13	1.14 ± 0.09	1.01 ± 0.09	1.00 ± 0.03	1.67 ± 0.07
CLD17-3	40.97 ± 2.72	44.72 ± 0.78	39.95 ± 4.07	62.77 ± 1.57	64.59 ± 1.39	491 ± 13	1.09 ± 0.08	0.89 ± 0.09	1.03 ± 0.03	1.64 ± 0.07
CLD17-4	43.44 ± 3.74	47.42 ± 0.92	45.99 ± 4.71	74.93 ± 1.97	77.76 ± 1.73	518 ± 15	1.09 ± 0.10	0.97 ± 0.10	1.04 ± 0.04	1.78 ± 0.08
CLD17-5	42.62 ± 3.78	42.82 ± 0.76	36.54 ± 4.55	68.15 ± 1.58	70.69 ± 1.48	434 ± 12	1.00 ± 0.09	0.85 ± 0.11	1.04 ± 0.03	1.51 ± 0.07

[a] Measurements made on dried and powdered sediment sub-samples of *c.* 7 g
[b] Mean ± total uncertainty (68% confidence interval), calculated as the quadratic sum of the random and systematic uncertainties

Table B. SAR protocols used in this study to undertake dose recovery tests on multi-grain aliquots (protocol A) and to obtain single-grain quartz OSL ages (protocol B). L_n and L_x refer to the natural and regenerative-dose OSL signal measurements, respectively. T_n and T_x refer to the test dose OSL signals measured after the L_n and L_x OSL signals, respectively. Each of these SAR measurement cycles was repeated for the natural dose, five different sized regenerative doses and a 0 Gy regenerative-dose (to measure OSL signal recuperation). Both the smallest and largest non-zero regenerative-dose cycles were repeated at the end of the SAR procedure to assess the suitability of the test-dose sensitivity correction. For protocol B, the smallest regenerative-dose cycle was also repeated a second time with the inclusion of step 2 to check for the presence of feldspar contaminants using the OSL IR depletion ratio of Duller (2003)

**A**	**Multi-grain OSL SAR protocol**	
**Step**	Treatment	Symbol
1[a]	Give dose	
2	Stimulate with infrared diodes at 50ºC for 60 s at 90% power	
3	Preheat to either 200, 220, 240 or 260ºC for 10 s	
4	Stimulate with blue LEDs at 125ºC for 60 s	L_n or L_x
5	Give test dose	
6	Stimulate with infrared diodes at 50ºC for 60 s at 90% power	
7	Preheat to 160 or 200ºC for 10 s	
8	Stimulate with blue LEDs at 125ºC for 60 s	T_n or T_x
9	Return to 1	

**B**	**Single-grain OSL SAR protocol**	
**Step**	Treatment	Symbol
1[a]	Give dose	
2[b]	Stimulate with infrared diodes at 50ºC for 60 s at 90% power	
3	Preheat to 240ºC for 10 s	
4	Stimulate with green laser at 125ºC for 2 s (90% power)	L_n or L_x
5	Give test dose	
6	Preheat to 160ºC for 10 s	
7	Stimulate with green laser at 125ºC for 2 s (90% power)	T_n or T_x
8	Return to 1	

[a] Step omitted when measuring the natural signal (L_n).
[b] Step added only when measuring the IR depletion ratio described in Duller (2003).

Table C. Single-grain OSL classification statistics for the natural D_e measurements. The proportion of grains that were rejected from final D_e estimation after applying the various SAR quality assurance criteria are shown in columns 3-11. These criteria were applied to each single-grain measurement in the order listed. Also shown are the single-grain OSL classification statistics for the dose recovery tests undertaken using different preheating conditions, where “240/160” equates to a PH1 of 240°C for 10 s and a PH2 of 160°C for 10 s, and “200/160” equates to a PH1 of 200°C for 10 s and a PH2 of 160°C for 10 s. T_n = natural test dose signal response; L_n/T_n = sensitivity-corrected natural signal response. BG = background. DRC = dose response curve

**CLD sample**	**17-1**	**17-2**	**17-3**	**17-4**	**17-5**	**17-2 (dose recovery test 240/160)**	**17-2 (dose recovery test 200/160)**
Grains measured (N)	900	1100	900	800	900	300	200
**Rejected grains (%)**	**56**	**49**	**52**	**51**	**44**	**56**	**54**
T_n signal <3xBG	8	6	8	9	11	4	7
Poor low recycling ratio	4	5	3	5	4	6	3
Poor high recycling ratio	4	5	4	3	4	3	2
IR depletion ratio	<1	<1	<1	<1	0	0	0
Recuperation >5%	9	8	9	6	9	4	4
Net T_n error >30%	1	2	1	3	3	<1	0
L_n/T_n not intercepting DRC	3	5	4	5	6	4	6
Anomalous dose-response curve	3	4	4	5	5	4	5
Saturated	56	49	52	51	44	56	54
**Accepted grains (%)**	**11**	**16**	**14**	**14**	**14**	**18**	**22**

Table D. Single-grain OSL D_e summary statistics, dose rates and OSL ages. The preferred age of each sample is highlighted in bold. For these samples, the preferred age has been derived using the statistical age model that yielded the optimum L_{max} score, following the criterion outlined in footnote [d] and Arnold et al. (2009)

Sample	Sample depth (cm)	Grain size (μm)	Total dose rate (Gy / ka)	Accepted/ Measured	Over-dispersion (%)	Weighted skewness	Critical skewness (95% C.I.) [a]	Critical skewness (68% C.I.) [a]	Age Model [b,c]	L_{max} score [d]	D_e(Gy) [e]	Age (ka) [e,f]
CLD17-1	393	212-250	2.23 ± 0.11	100/900	37 ± 3	0.905	0.489	0.244	CAM	-55.694	98.6 ± 4.1	44.2 ± 3.1
									MAM-3	-47.863	84.1 ± 4.2	**37.7 ± 2.8**
									MAM-4	-47.716	85.5 ± 4.2	38.4 ± 2.8
CLD17-2	459	212-250	2.99 ± 0.15	180/1100	24 ± 2	0.020	0.365	0.183	CAM	-48.832	118.4 ± 2.7	**39.6 ± 2.3**
									MAM-3	-48.635	112.2 ± 3.9	37.5 ± 2.4
									MAM-4	-48.510	112.4 ± 31.3	37.6 ± 10.7
CLD17-3	469	212-250	3.08 ± 0.15	127/900	27 ± 2	0.122	0.435	0.217	CAM	-41.452	116.6 ± 3.4	**37.9 ± 2.3**
									MAM-3	-40.409	102.8 ± 10.4	33.4 ± 3.8
									MAM-4	-40.212	106.0 ± 10.5	34.5 ± 3.9
CLD17-4	513	212-250	3.02 ± 0.15	108/800	23 ± 2	0.034	0.471	0.236	CAM	-24.530	126.8 ± 3.5	**42.0 ± 2.5**
									MAM-3	-23.339	122.2 ± 4.3	40.5 ± 2.6
									MAM-4	-23.189	122.1 ± 4.2	40.4 ± 2.6
CLD17-5	525	212-250	2.79 ± 0.14	129/900	35 ± 3	-0.103	0.431	0.216	CAM	-63.595	162.9 ± 5.7	**58.4 ± 3.8**
									MAM-3	-65.695	127.9 ± 5.8	45.8 ± 3.2
									MAM-4	-64.107	118.0 ± 10.8	42.3 ± 4.5

[a] Weighted skewness scores have been calculated on log-transformed D_e values using Eq. 7-8 of Arnold and Roberts (2009). Critical skewness scores have been calculated using Eq. 16 of Bailey and Arnold (2006). Critical skewness values are taken to be equivalent to twice the standard error of skewness score (95% C.I.) for single-grain D_e datasets, following the results of sensitivity analyses performed by Bailey and Arnold (2006) and Arnold et al. (2007).

[b] CAM = central age model; MAM-3 = 3-parameter minimum age model; MAM-4 = 4-parameter minimum age model (Galbraith et al., 1999).

[c] D_e estimates have been calculated after adding, in quadrature, a relative error of 20% to each individual D_e measurement error to approximate the underlying dose overdispersion observed in 'ideal' (well-bleached and unmixed) sedimentary samples from this site (CLD17-2, CLD17-3, CLD17-4), the single-grain dose-recovery tests performed on the Caldeirão samples (CLD17-2) and from global overdispersion datasets (Arnold and Roberts, 2009).

[d] Maximum log likelihood score of the CAM, MAM-3 or MAM-4 fit. For a given sample, the L_{max} score of the MAM-3 is expected to be substantially higher (i.e. at least 1.92 greater) than that of the CAM when the addition of the extra model parameter improves the fit to the data. Likewise, the L_{max} score of the MAM-4 is expected to be significantly greater than that of the MAM-3 (by at least 1.92 when compared with the 95% C.I. of a X^2 distribution) when the addition of the extra model parameter improves the fit to the data. If the extra parameter of the MAM-3 (or MAM-4) is not supported by the data, then its L_{max} score will be similar to (i.e. within 1.92 of) the CAM (or MAM-3) L_{max} score, indicating that the simpler age model explains the data equally well (Arnold et al., 2009).

[e] Mean ± total uncertainty (68% confidence interval), calculated as the quadratic sum of the random and systematic uncertainties. Total uncertainty includes a systematic component of ±2% associated with laboratory beta-source calibration.

[f] The preferred age for each sample is shown in bold. For these samples, the preferred age has been derived using the statistical age model that yielded the optimum L_{max} score, following the criterion outlined in footnote [d] and Arnold et al. (2009).

Pigment analysis procedures and results

Methodology

We employed three techniques to characterise the elemental and mineralogical composition of sediment samples and of residues adhering to three directly dated marine shells from Gruta do Caldeirão: P13sc491 (**Fig 12, no. 1**; OxA-22299) and P13-402 (**Fig 12, no. 2**; OxA-22300), from layer Jb (henceforth Shell 1 and Shell 2, respectively); P11sc968 (**Fig 12, no. 4**; OxA-22301), from layer K (henceforth Shell 3). Raman spectroscopy was applied to residues and uncoated areas of the three shells. A portable X-ray fluorescence (pXRF) analyser was used for sediment samples. Scanning electron microscopy (SEM) coupled with energy dispersive X-ray spectrometry (EDS) was used for the analysis of Shell 3 and sediment samples. The latter were also analysed by means of X-ray micro-diffraction (µXRD).

Raman analyses were conducted with a Raman Senterra (BRUKER) device equipped with a 532 nm laser and using an illumination intensity of 2 mW. Scattered light was collected through a 50× objective.

The SEM-EDS instrument was a PHILIPS XL30 ESEM model with an electron gun LaB6 coupled with Si(Li) EDS. The samples were observed and analysed without any preparation, in controlled pressure mode (pressure of 10^{-4} Torr). The acceleration voltage was set to 20 kV.

µXRD analysis were carried out on a dedicated, laboratory-made device using a Rigaku monochromatic source (λ=1.54186 Å) and a 200 µm collimator. The maximum voltage and current were set at 45 kV and a 660 µA, respectively. The incident beam was positioned to form a grazing angle with the surface of the sample. The analysed area was about 1 mm². A 2D Rigaku imaging plate detector (R-AXIS IV++) and a motorized X,Y,Z,ϕ positioning system with an independent θ axis were coupled to the XRD equipment. Acquisition time was set at 3 minutes. The circular diffractograms were calibrated in 2θ and transformed into linear ones through the software Fit2D v.12.077, developed by Andy Hammersley (European Synchrotron Radiation Facility, Grenoble, France). Data treatment was performed with the EVA© software (Bruker).

pXRF measurements were carried out with a SPECTRO xSORT (AMETEK) instrument, equipped with a silicon drift detector (SDD) and a low power W X-ray tube with an excitation source of 40 kV. Measurements were acquired in the air with a constant working distance by using a lead receptacle to which the spectrometer is fixed. Light elements such as Na, Mg, and Al are not detected with this technique. An area of 8 mm in diameter was analysed. Spectra acquisition times were set to 60 s. The spectrometer is internally calibrated by an automated measure of the contents of a standard metal shutter. Data treatment was realised using standard materials and after two-time calibration of the results.

Results

Observation under a reflected light optical microscope identified remnants of a red coating and whitish residues on Shell 1, and red, orange, and white residues on Shell 2 (**Figs D and E**). Three superimposed deposits of different colour were detected on the surface of Shell 3 (**Figs F-H**): the first, adhering to the shell's test, is bright red in colour and composed of fine, sorted particles; the second is a thicker and coarser orange/reddish layer that, in places, covers the first; the third is an even thicker, whitish layer that covers both and can also be seen in the fill of the shell's aperture, indicating that it must relate to the sedimentary matrix.

SEM observation confirms the differences in texture between the bright red and orange/reddish layers of Shell 3 (**Fig H**): most particles composing the inner bright red layer are <1 µm and those in the order of 5 µm are rare, which indicates a clayey texture. However, the elements composing both layers are the same (Si, Al, Ca, K, Fe, P), albeit in different proportions (P is substantially more abundant in the outer orange/reddish layer).

Raman spectroscopy (**Table E; Fig D**) identified the presence of hematite and calcite in, respectively, the red and the whitish residues coating Shell 1. Hematite was also identified in the red residues found on the surface of Shell 2, whose pale reddish coating was dominated by calcite with traces of hematite; the analysis of the shell's test reveals diagenetically unmodified aragonite (**Fig E**). The bright red layer adhering to the surface of Shell 3 is composed of hematite, possibly associated with ferrihydrite, and calcite, while the overlying orange/reddish layer contains either hematite associated with calcite or hematite (and, possibly, magnetite) associated with phosphates. In Shell 3, however, the hematite spectra identified in the bright red and orange/reddish layers differ from reference spectra in the form of line shift, change in relative intensities, and absence of bands beyond 400 cm-1 (**Table F)**. These anomalies can be due to excessive laser power (2 mW, wavelength 532 nm), and are not necessarily related to the crystallinity of the hematite (Faria et al., 1997).

µXRD analysis of the bright red layer of Shell 3 identified quartz, calcite, aragonite, clays of the illites/glauconites family, and kaolinite (**Fig G; Table G**). Hematite was not detected, but the main peak of this mineral coincides with an aragonite peak, and a secondary peak coincides with a kaolinite one. In light of the Raman results, the failure of µXRD to detect hematite must be due to method limitations. Indeed, µXRD similarly failed when analysing red residues found in the overlying orange/reddish layer. The latter was found to be mainly composed of calcite and aragonite, while the bright red layer also showed traces of illite/glauconite and kaolinite.

The pXRF analysis of sediments from layers Jb and K identified a notable proportion (4-5%) of iron oxides, as intimated by their reddish colour (**Table H**). However, iron oxides were not detected by µXRD (**Table I**), which probably implies that, in the sediment, such oxides are found in poorly crystalline form only. Otherwise, both layers have a similar mineralogical composition: quartz, calcite, feldspar (microcline or other), calcium phosphate (hydroxyapatite family), and illite/muscovite are present in both; kaolinite is the single mineral found in one layer only (layer Jb).

Synthesis

The layer of residue adhering to the surface of Shell 3 differs from the overlying orange/reddish layer in colour (bright red), grain size (clayey), and composition (less calcium phosphate). The composition of the orange/reddish layer is intermediate between the underlying bright red layer and the sedimentary matrix of layer K, where the shell was retrieved. The bright red and the orange/reddish layer also feature a higher proportion of clay minerals (illite/muscovite and kaolinite), which is consistent with the clayey grain size of the bright red layer. The layer's matrix contains a small proportion of iron, but µXRD failed to identify hematite. Iron compounds (oxy-hydroxides or oxides) seem to be present in the sediment in poorly crystalline form, whereas in the residues found on all the shells they appear as hematite (although this difference would need to be confirmed by Raman analysis of the sediment).

Overall, these patterns are consistent with two different interpretations. The first is that the bright red layer seen on Shell 3 represents the remnant of a hematite-rich compound that (a) covered the shell during its use as an ornamental object, and (b) was still present on its surface at the time of loss (or discard) and eventual incorporation in the deposit. The second hypothesis is that said bright red layer (a) corresponds to the finest fraction of the sediment making up archaeological layer K, and (b) represents post-depositional accumulation. Although it cannot formally ruled out, this second hypothesis is unparsimonious and indeed rather unlikely, as it requires us to postulate an unknown mechanism by which, prior to its eventual deposition on the shell's surface, the fine fraction would have been segregated, with some of the minerals that make it up being eliminated in the process. Provided we interpret the reddish deposits found in Shells 1 and 2 alike the orange/reddish layer of Shell 3, i.e., as remnants of a pigmentatious compound diluted in a calcitic

matrix, we can conclude that, originally, the three shells were coated with a red, hematite-rich colouring mixture.

References

Cuscó, R., Guitián, F., Aza, S.d., Artús, L., 1998. Differentiation between hydroxyapatite and β-tricalcium phosphate by means of μ-Raman spectroscopy. Journal of the European Ceramic Society 18, 1301-1305.

de Faria, D.L.A., Venâncio Silva, S., de Oliveira, M.T., 1997. Raman microspectroscopy of some iron oxides and oxyhydroxides. Journal of Raman Spectroscopy 28, 873-878.

Froment, F., Tournié, A., Colomban, P., 2008. Raman identification of natural red to yellow pigments: ochre and iron-containing ores. Journal of Raman Spectroscopy 39, 560-568.

Gillet, P., Biellmann, C., Reynard, B., McMillan, P., 1993. Raman spectroscopic studies of carbonates part I: High-pressure and high-temperature behaviour of calcite, magnesite, dolomite and aragonite. Physics and Chemistry of Minerals 20, 1-18.

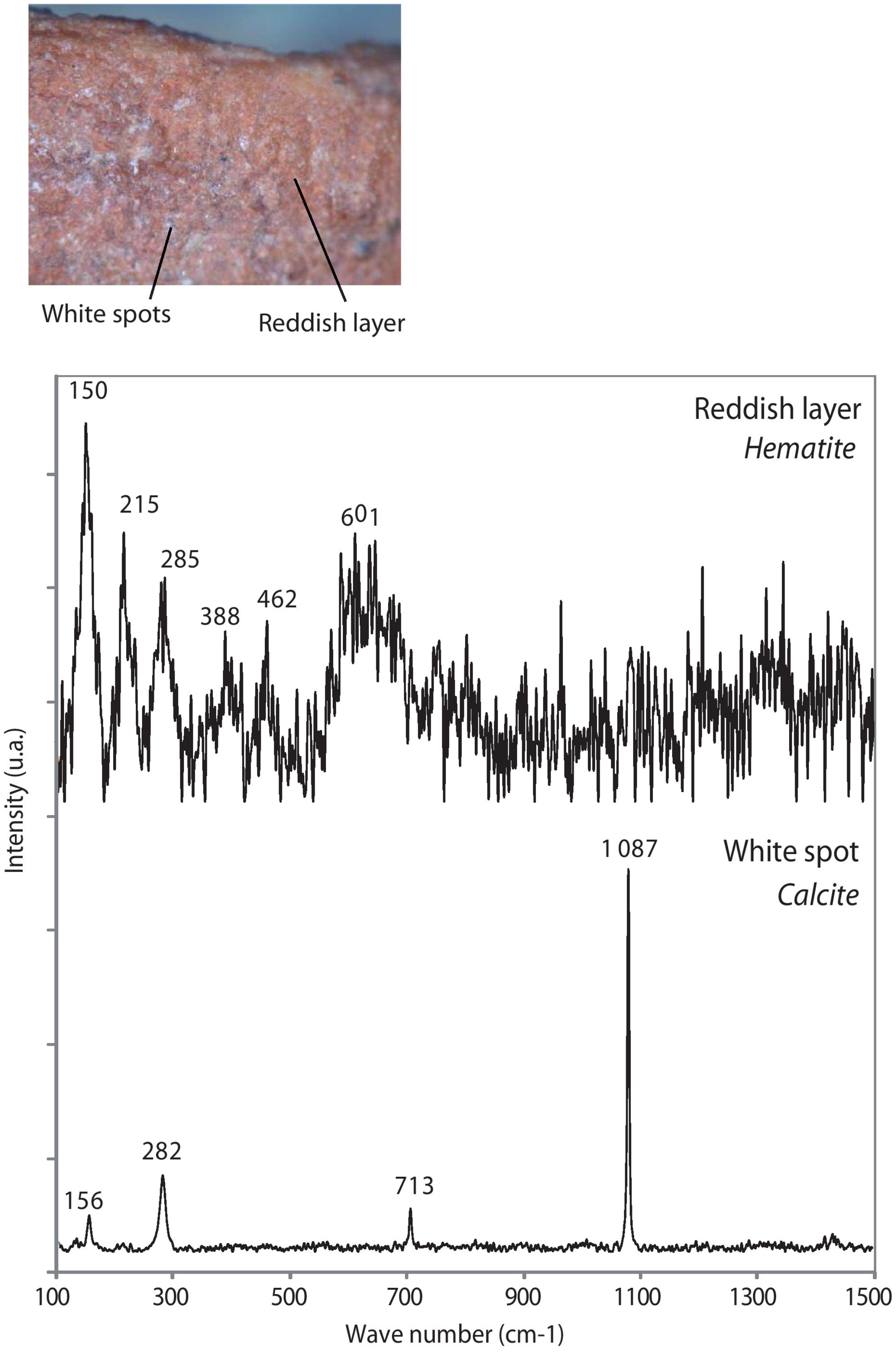

Fig D. Shell 1 (P13sc491; layer Jb). Detail of the reddish deposit covering the shell and Raman spectra of that deposit compared to a white spot of the shell's test itself.

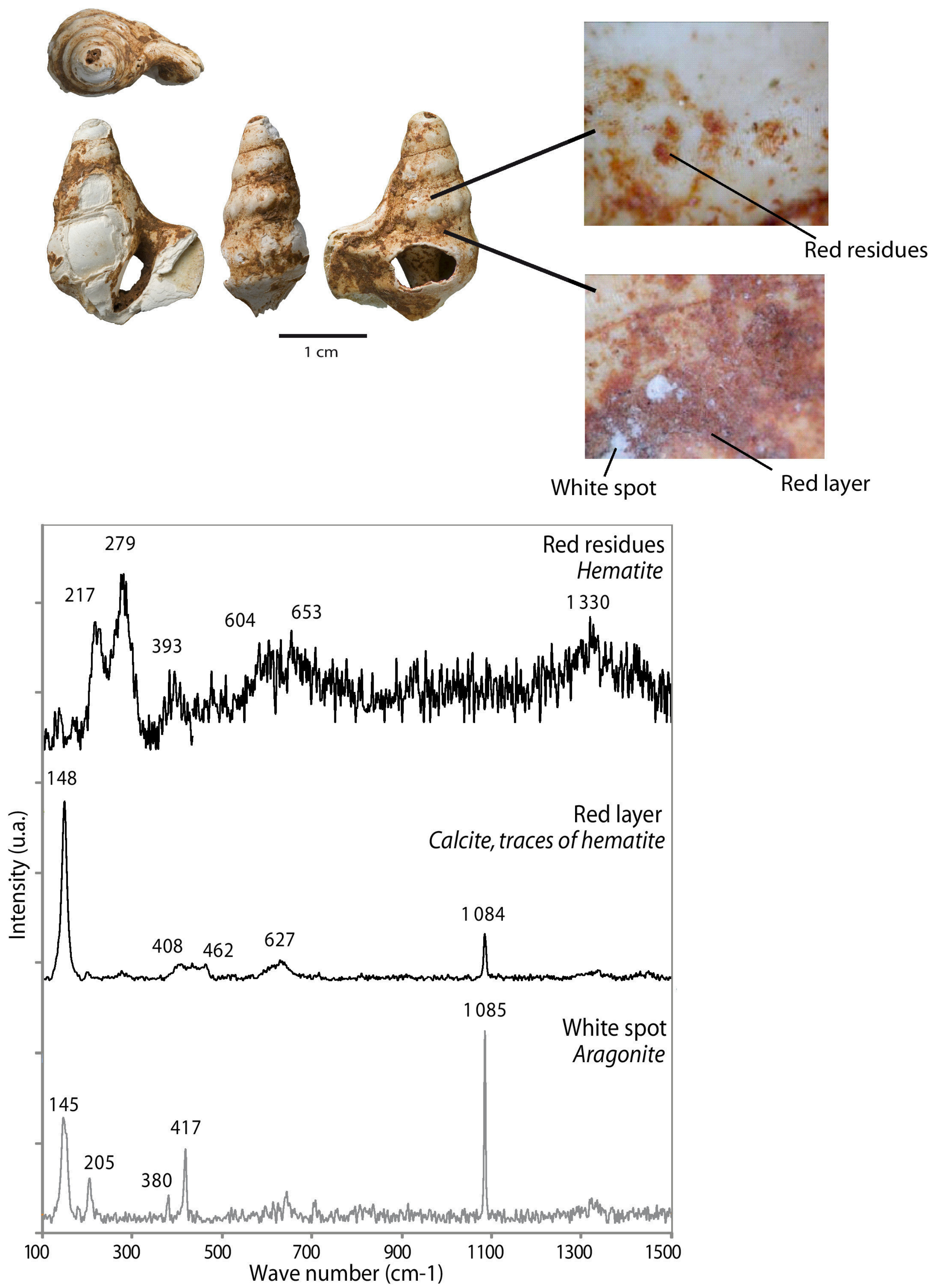

Fig E. Shell 2 (P13-402; layer Jb). Details of the reddish deposit covering the shell and Raman spectra of that deposit compared to a white spot of the shell's test itself.

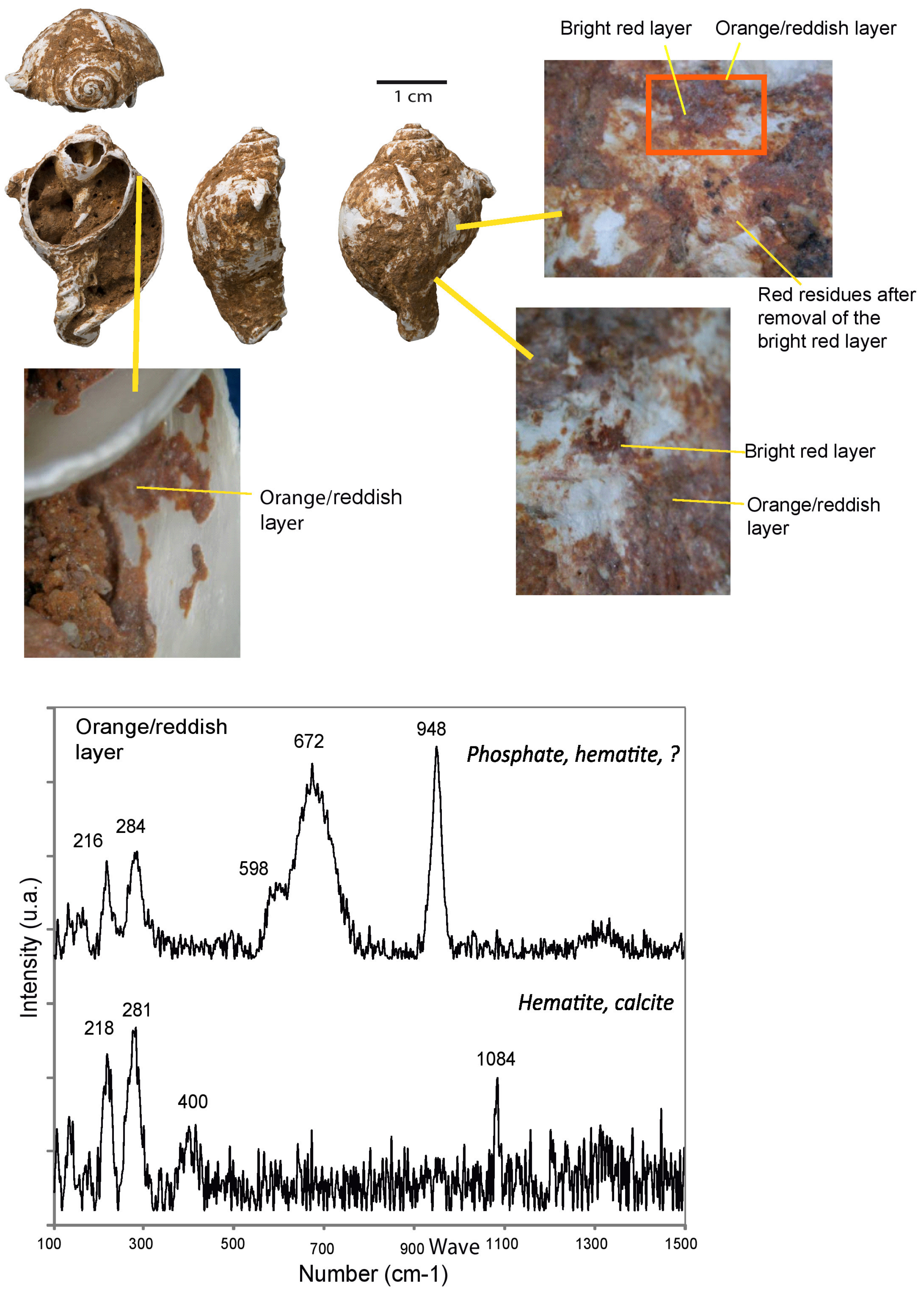

Fig F. Shell 3 (P11sc968; layer K). Details of the orange/reddish and bright red deposits covering the shell and Raman spectra of those deposits compared to a white spot of the shell's test itself.

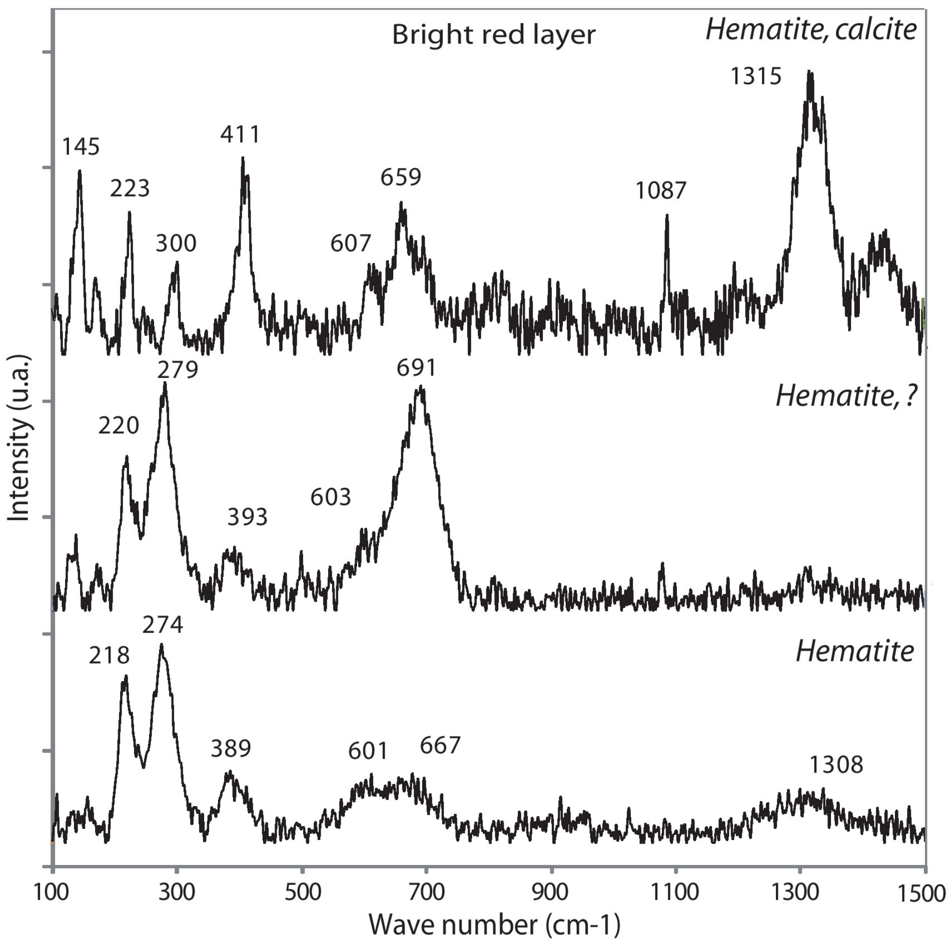

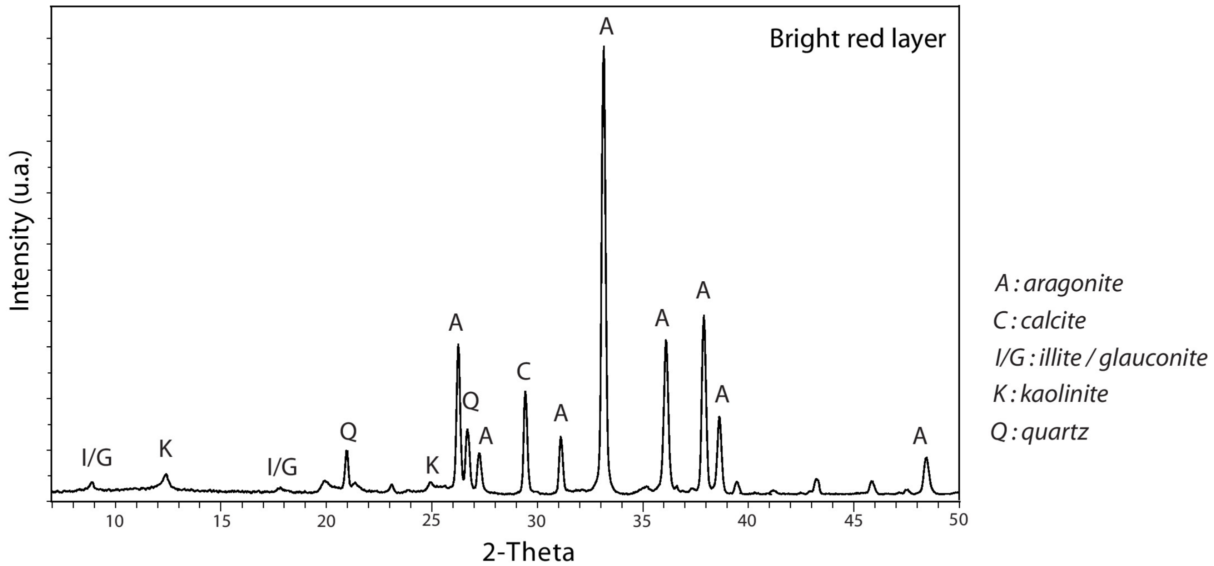

Fig G. Shell 3 (P11sc968; layer K). Raman spectra and X-ray diffraction pattern of the bright red layer.

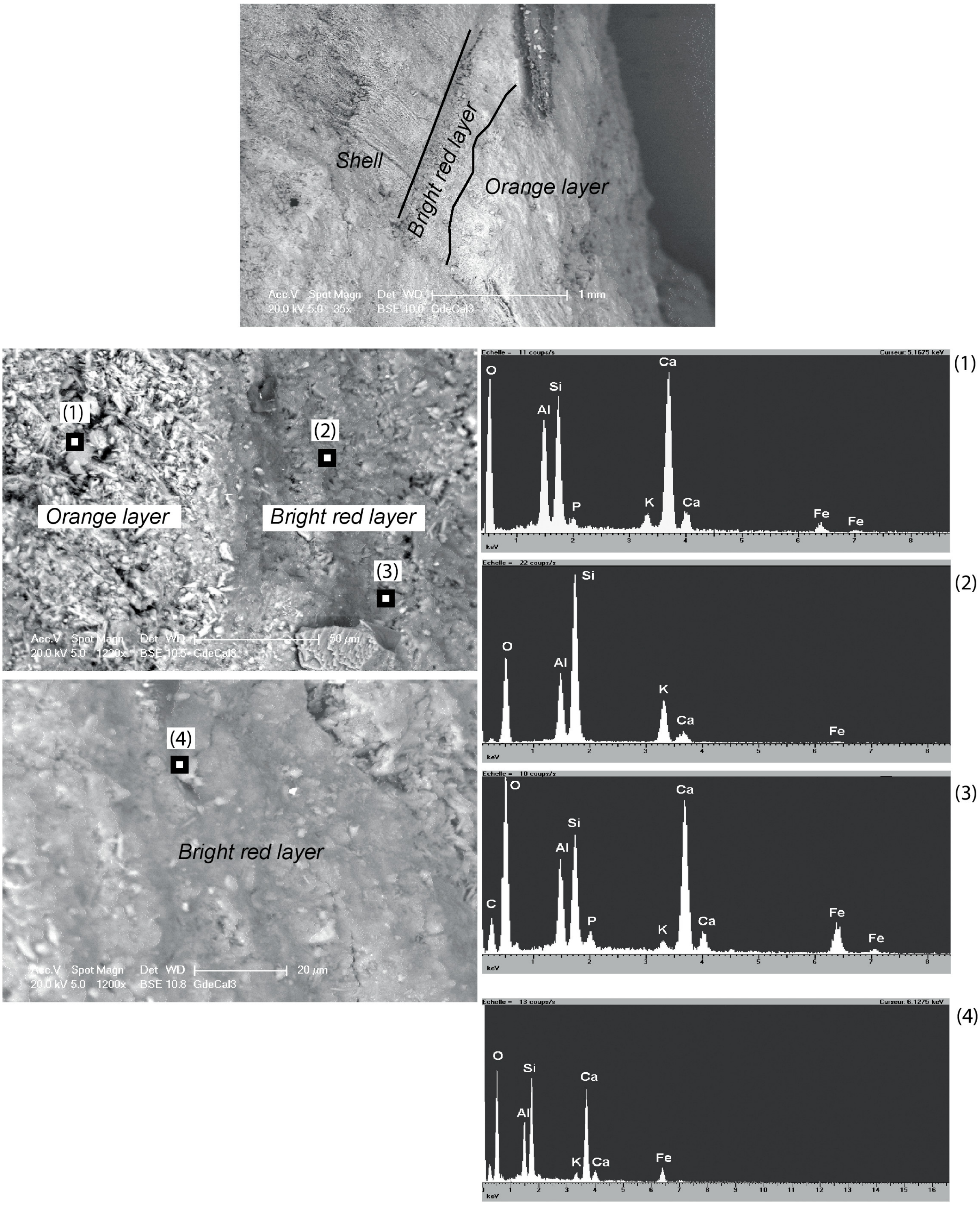

Fig H. Shell 3 (P11sc968; layer K). SEM back scattered images and EDS results of the orange/reddish and bright red deposits.

Table E. Results of the Raman analyses on three marine shells from Caldeirão

Shell	Layer/Area	Minerals identified	Spectra (N)
1	Red layer	Hematite	1
	White area	Calcite	1
2	Red residues	Hematite	1
	Orange/reddish layer	Calcite, traces of hematite	1
	White area	Aragonite	1
3	Bright red layer	Hematite (plus magnetite?); calcite	10
	Orange/reddish layer	Hematite, traces of calcite; hematite, phosphates (plus magnetite?)	3

Table F. Reference Raman bands for the minerals identified on Caldeirão samples

Minerals	Reference Raman bands (cm-1)	References
Hematite	225 vs, 240 sh, 290 vs, 410 m, 490 w, 610 w 1320 vs	Faria et al., 1997<br>Froment et al., 2008
Calcite	150 m, 280 m, 710 m, 1085 vs	Gillet et al., 1993
Aragonite	150, 210, 280, 700 m, 1085 vs	Gillet et al., 1993
Phosphates	400-490 w, 570-620 w, 970 vs	Cuscó et al., 1998
Magnetite	300 w, 550 w, 670 vs	Faria et al., 1997<br>Froment et al., 2008

Table G. Results of µXRD analyses conducted on Shell 3 from Caldeirão

Layer/Area	Calcite	Aragonite	Quartz	Illite/glauconite	Kaolinite
Bright red layer	+	+++	++	-	-
Red residues in the bright red layer	++	+++	+	+	+
Orange/reddish layer	+++	++	+	-	-

Table H. Results of the X-ray fluorescence analysis of Caldeirão sediments [a]

		SiO_2 (%)	P_2O_5 (%)	SO_3 (%)	K_2O (%)	CaO (%)	TiO_2 (%)	MnO (%)	Fe_2O_3 (%)	Analyses (N)
Sediment Jb	Average	48	10.7	1.6	1.95	33	0.45	0.168	4.6	4
	sd	1	1.7	–	0.08	1	0.03	0.005	0.1	
Sediment K	Average	64	7.4	1.2	2.18	25.0	0.53	0.215	5.6	3
	sd	3	–	–	0.03	0.6	0.03	0.004	0.0	

[a] except for phosphorus, element concentration was controlled by the use of standards

Table I. Results of the X-ray diffraction analysis of Caldeirão sediments

	Calcite	Quartz	Illite/glauconite	Kaolinite	Hydroxyapatite	Feldspar
Layer Jb	+++	++	-	-	+	+
Layer Jb <200 µm	++	+++	+	+	+	+
Layer K	++	+++	+	-	+	+

Stratigraphic provenance of key finds

O13sc91 (MAMS-38336)

The specimen is a non-plotted horse tooth retrieved in spit E4 of square O13 (see **Fig 3** for the location of this grid unit in the Corridor area of the site). The décapage plans illustrating the excavation of that spit, carried out in 1983 between August 26 and August 31, are reproduced in **Fig I**. The hand-written annotations provide elevations of both the sediment and the upper and lower surfaces of the blocks exposed at the base of each spit. Note the large animal burrows and the linear disturbance features, which denote root paths. The description emphasises the large number of rabbit bones retrieved, especially in and around the larger burrow feature against the cave wall.

O13 was the first grid unit to be opened in order to extend the Back Chamber trench towards the Corridor. At the time, it was assumed that the latter would feature the same stratigraphic succession and, therefore, that the first spits of the reddish-brown deposit below layer ABC-D would correspond to layer Ea. The décapage descriptions reflect that assumption. Only subsequently, with further outward expansion of the excavation trench, was it possible to recognise that layer Ea wedged out at the transition between Back Chamber and Corridor, and that spits E1-E4 belonged in the upper part of layer Eb, not in layer Ea.

O12-84 (OxA-X-2786-13)

The specimen is a human left mandibular fragment. It belonged to an early adolescent: the Caldeirão 2 individual, as described in Trinkaus et al. (2001). The dm_2 is preserved in its socket. The fossil was retrieved in a small burrow against the cave wall, during the excavation of spit H1 of grid unit O12 (**Fig J**). The latter corresponds to a triangular surface created by the site's gridding against the north wall of the Back Chamber (see **Fig 3** for its location on the site plan). Here, layers Fa-Jb were excavated between July 7 and August 5, 1986, i.e., after the surface of layer K in the adjacent P row had already been reached (during the previous field season, in 1985). The idea was to double-check, by careful décapage, the E→W dip of the stratification suggested by observation of the P>O/11-12 profile, and to verify, by comparison with the opposite profile (P>Q/11-13), that the N→S dip was indeed negligible, as the excavation of the P row had suggested.

The hand-written annotations provide elevations of the sediment at the base of each spit. They also contain summary descriptions of matrix and clasts, reflecting how the Fc/H interface was first thought to correspond to the base of spit F8, with continued excavation showing that a few cm remained before the surface of H was truly exposed, which was the case at the base of spit F9. Note that the burrow only appeared as the surface of H was reached. This evidence suggests that the disturbance was a small scale one and that the finds made in the burrow are reworked from layer H itself, not intrusive form layer Fc above.

P13-403 (OxA-5541)

The specimen is a distal metapodial of red deer from spit J6 of square P13, at the base of which the Jb/K interface was reached in most of the square (**Fig K**). In this part of the cave (squares O-P/13-14), at the 90° angle between Back Chamber and Corridor, controlling for the presence of a double dip (E→W in the former, N→S in the latter) was hindered by the relative homogeneity displayed by the matrix through the succession of layers Fa-L. Even though often aided by such clues as the presence of stone lines, incrustation lenses, or flat-bottomed slabs denoting the actual disposition of past cave floors, the décapage of stratigraphic interfaces in P13 was always rather approximate.

This difficulty may explain the erroneous assignment of P13-403 to "layer K-top" that appeared in previously published reports on the site's dating (e.g., Zilhão, 1997). As shown by the décapage plans reproduced in **Fig K**, P13-403 was found at the same elevation and adjacent to the retouched flint knife and directly dated *Aporrhais pespelecani* shell illustrated in **Figs 10 and 12**.

When their position is assessed against a virtual surface reconstructed from the elevations found in the more reliable excavation records — the topography of the Jb/K interface in O/13-14, and its elevation along the P>Q/11-13 profile — the three items lay at, or just above the base of layer Jb. Indeed, this exercise shows that, at the base of spit J6 of P13, the surface of layer K (a) had yet to be reached in the square's SW corner, and (b) conversely, due to the heavy induration of the deposit, which hindered a precise décapage of layer boundaries, it had been somewhat undercut in the square's SE corner (without consequence, however, as that corner was entirely devoid of finds). Note the root burrow along the wall in O13, which was not detected when, a month before (July 31, 1986), the same surface had been exposed in P13.

O13-361

The specimen is a large quartz sidescraper (**Fig 11, no. 1**) retrieved in square O13 at the surface of layer L. In previous publications (e.g., Zilhão, 1997), it was assigned to layer K, which we correct here.

The following reasons explain the original misassignment: (a) the excavation of squares O/13-14 and P13 down to the surface of layer L was carried out at the very end of the project (September 12 and 14, 1988, respectively), and it stopped at the elevation of that boundary; (b) no subsequent field assessment of the stratigraphic accuracy of the assignment of finds then made was therefore possible; (c) through the excavation of the site, all finds made during the last, fine-décapage stage of the exposure of stratigraphic interfaces were by convention recorded as belonging in the unit above the interface. Following this convention dictated that O13-361 be recorded as "K," even though this was the first artefact found in the excavation of O/13-14 since the quartzite flake O13-346, which lay 30 cm higher-up, at the top of layer Jb. In addition, at the time, layer K was thought to belong in the Middle Palaeolithic. Whether this item came from K or L was therefore not regarded as hugely significant, and whether the convention ought to be ignored in this case was not considered to be an issue of chronostratigraphic importance.

As shown by the décapage plans reproduced in **Fig L**, the elevation of O13-361 clearly places it at the very top of layer L, not in layer K. The plan also shows that, due to the same "double-dip" problems mentioned in relation to the P13-403 radiocarbon sample, the K/L interface was significantly undercut in the NE part of P13. The same happened in its SE corner, due to induration. Elsewhere along the P>Q13 profile, however, induration had the opposite effect, i.e., the décapage could not proceed to the exact interface and remained a few cm above it.

References

Trinkaus, E., Bailey, S.E., Zilhão, J., 2001. Upper Paleolithic human remains from the Gruta do Caldeirão, Tomar, Portugal. Revista Portuguesa de Arqueologia 4, 5-17.

Zilhão, J., 1997. O Paleolítico Superior da Estremadura portuguesa. Colibri, Lisboa.

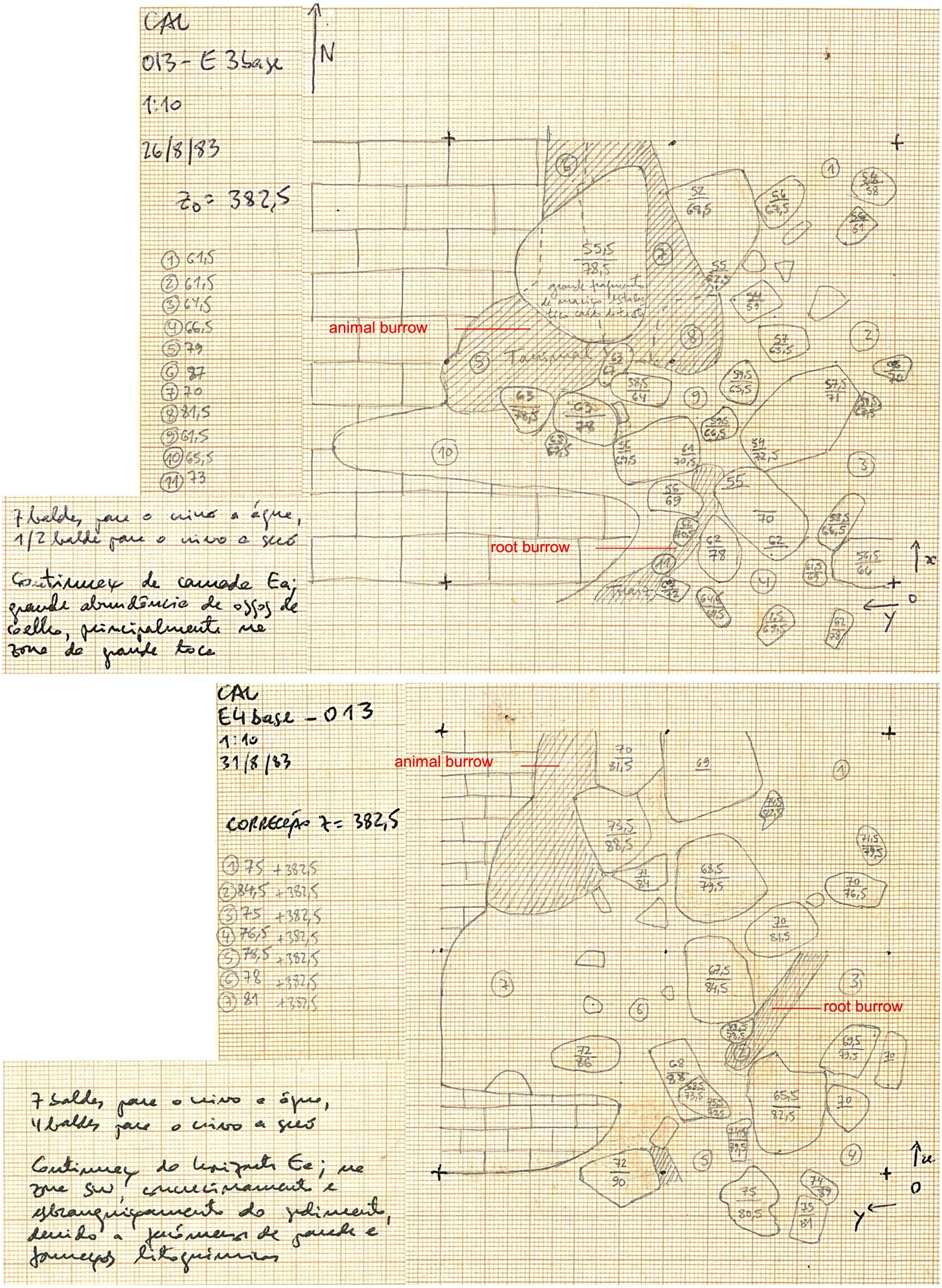

Fig I. O13, field records for spits E3-E4. Description and elevation of the surfaces delimiting the thickness of deposit that yielded the O13sc91, non-plotted horse tooth; its radiocarbon age (20,077 ± 100 BP; MAMS-38336) shows that this an upwardly moved find derived from the underlying Solutrean deposit.

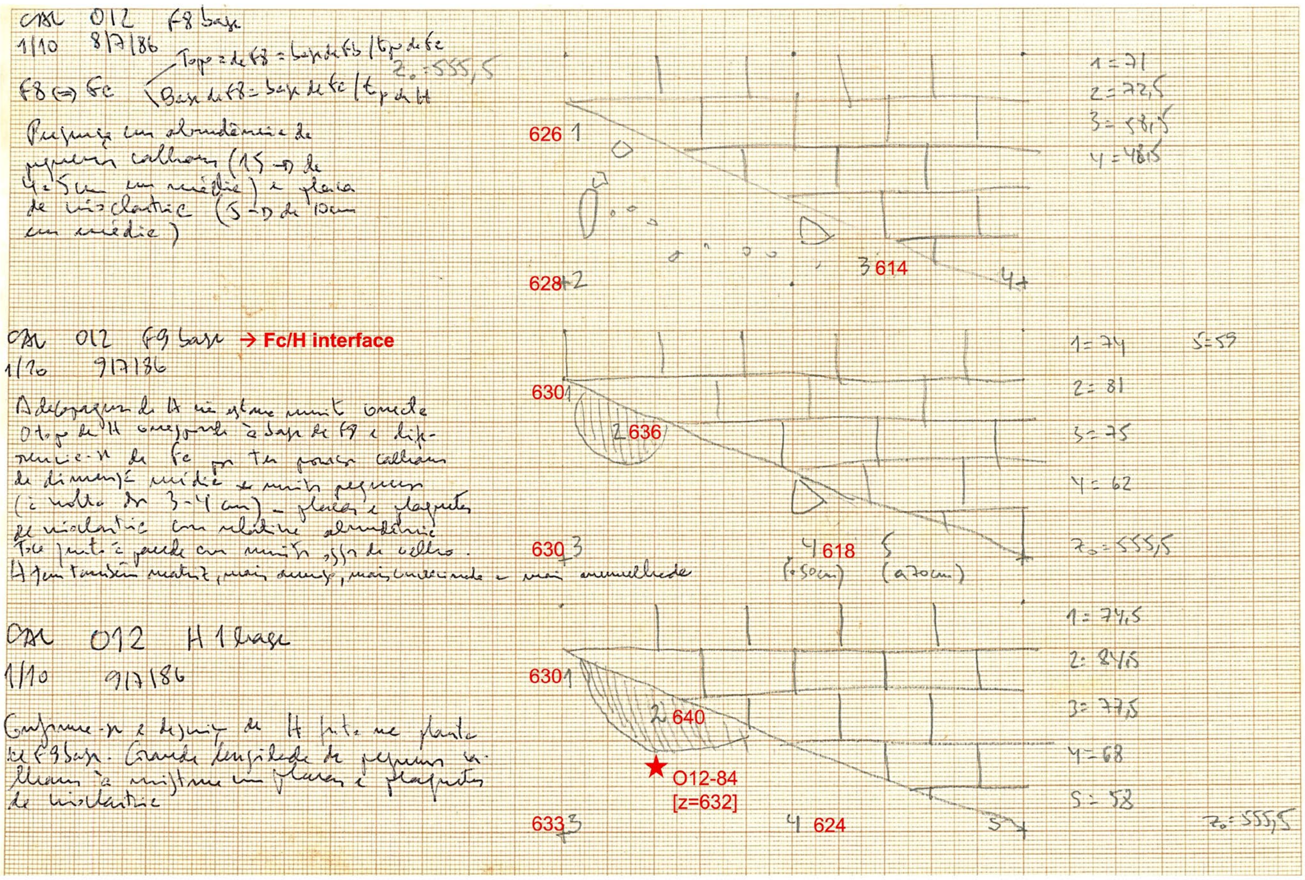

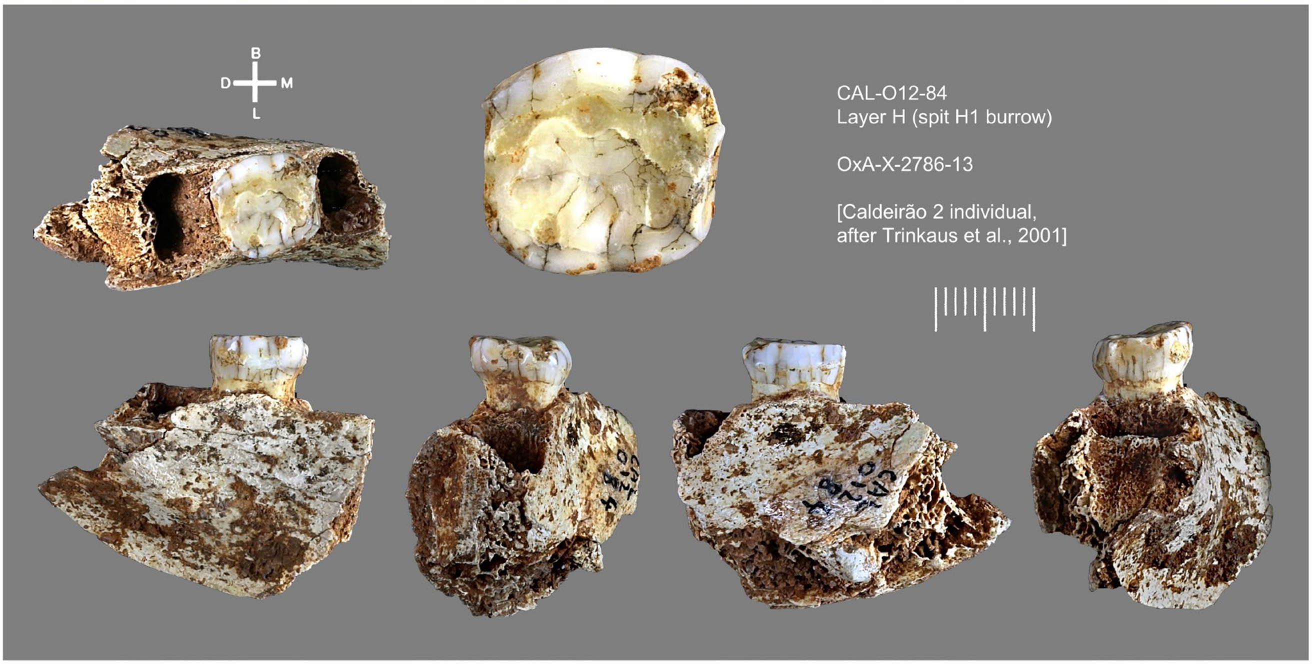

Fig J. O12, field records for spits F8-H1. Description and elevation of the surfaces delimiting the thickness of deposit across which the Fc/H interface was exposed and excavated in grid unit O12, and the O12-84 human fossil (whose [x,y] coordinates are indicated by the star) retrieved in the small burrow exposed at that interface and radiocarbon dated to 19,400 ± 150 BP (OxA-X-2786-13).

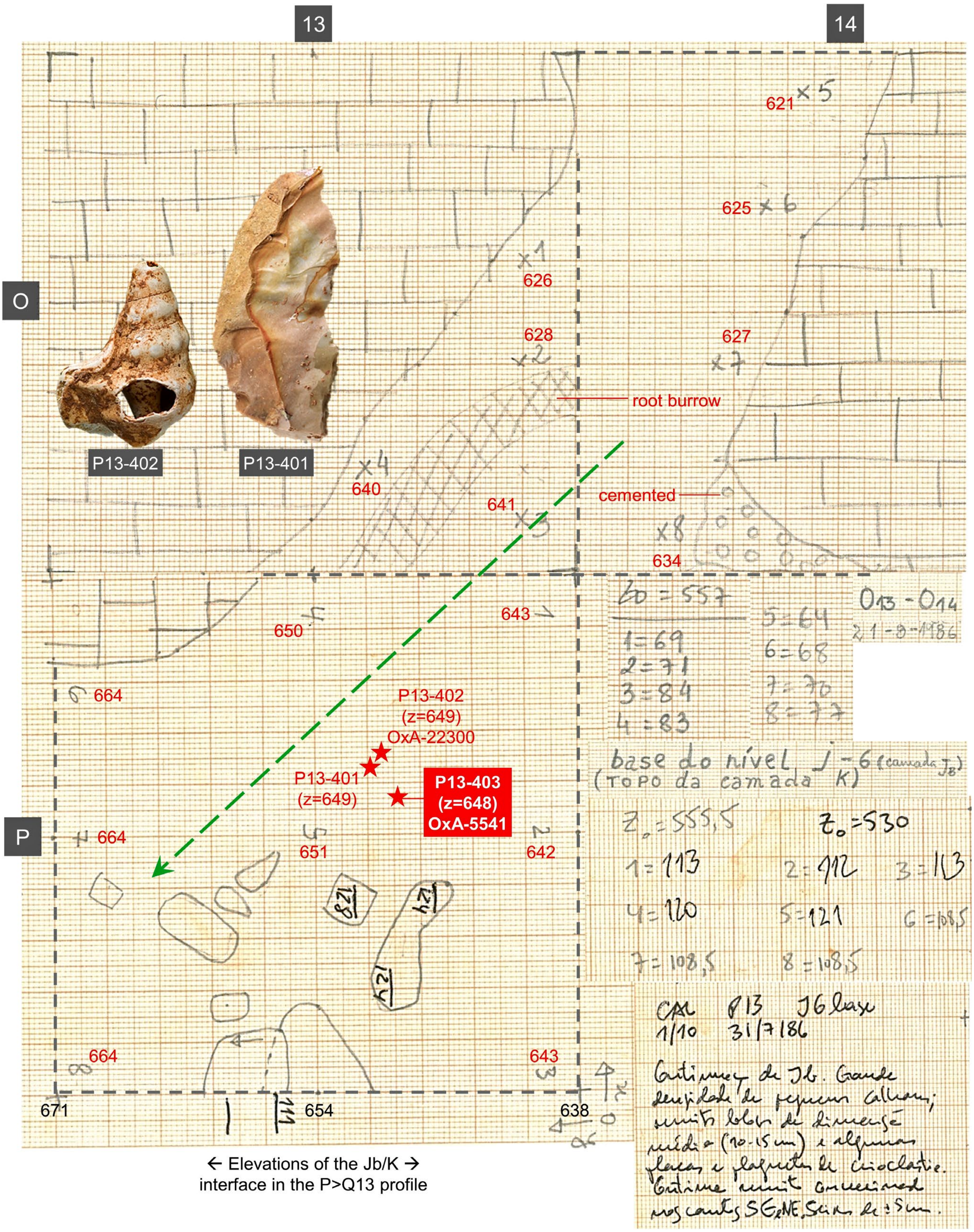

Fig K. Field records for spit J6 of squares O/13-14 and P13. Description and elevation of the décapage surface approximately corresponding to the interface between layers Jb and K. The arrow indicates the dip of the stratification. The stars mark the [x,y] coordinates of the P13-401, 402 and 403 finds. Their elevations are indicated, and show that all three belong in layer Jb.

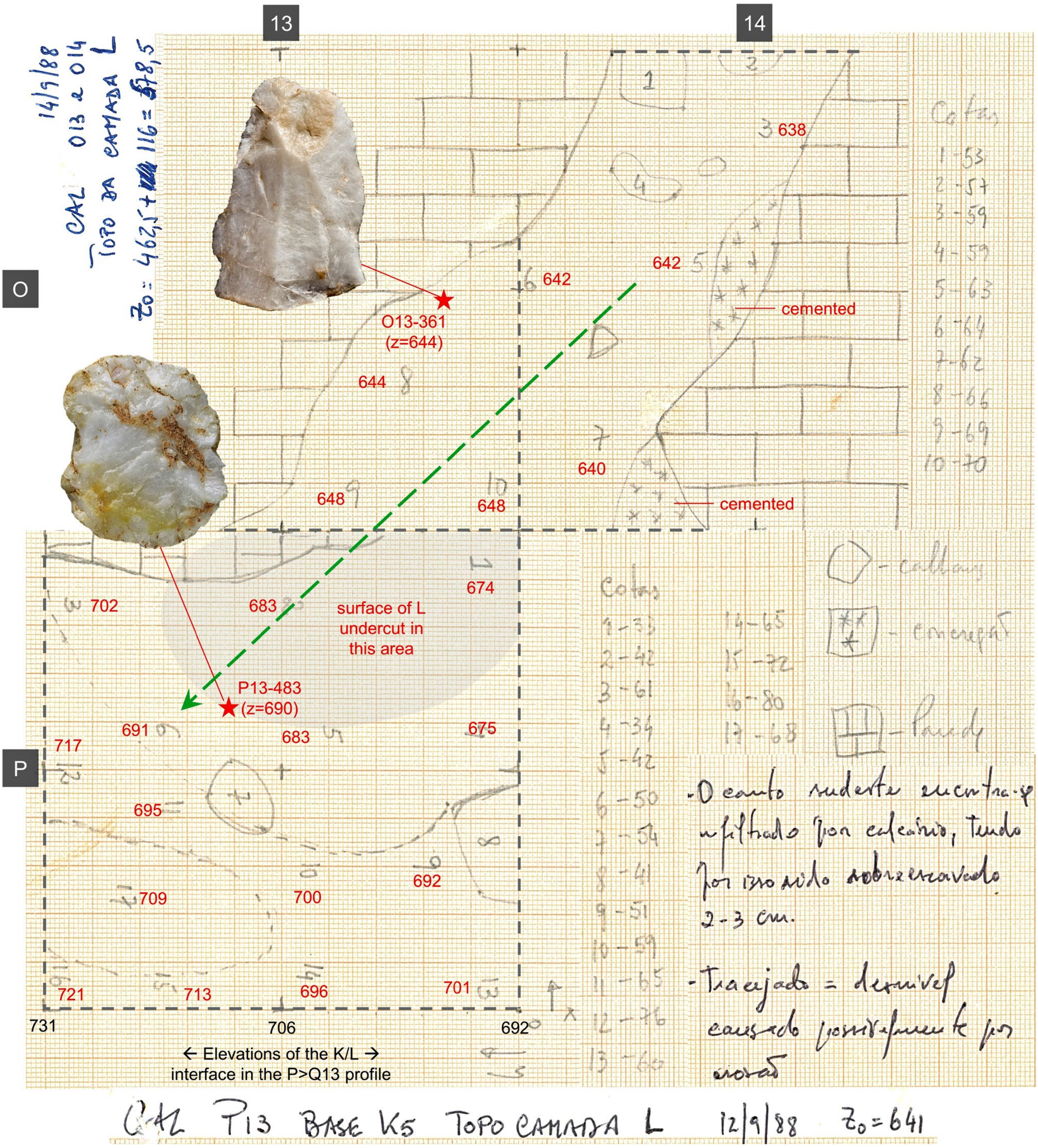

Fig L. Field records for spits K1 of O/13-14 and K5 of P13. Description and elevation of the décapage surface approximately corresponding to the interface between layers Jb and K (in O/13-14, excavated as a single spit). The arrow indicates the dip of the stratification. The stars mark the [x,y] coordinates of the O13-361 and P13-483 finds. Their elevations are indicated, showing that both items belong in layer L.

Bayesian age modelling of the succession

Methods

Bayesian modelling was undertaken using OxCal v4.4 (Bronk Ramsey, 2009a), following the general approach outlined in Demuro et al. (2019, 2020). The sedimentary sequence has been modelled using a *Sequence* depositional model, incorporating stratigraphic units in ordered succession and separated by associated *boundaries*. The Gruta do Caldeirão Bayesian model focuses on the eleven layers comprising the pre-Magdalenian archaeological sequence excavated in the Back Chamber (layers Fa-O), plus the two Middle Palaeolithic layers from the Entrance Trench (Units 5-6). The dating determinations for individual units are represented as a grouped set of likelihoods (*Phase*) within the *Sequence* model. *Boundaries* have been used to delineate the beginning and end of each stratigraphic unit, and to specify that all likelihoods or events included in these groupings have a uniform prior likelihood of occurrence. Separate rather than shared *boundaries* have been used to delineate the beginning and end of each stratigraphic unit to ensure the model is able to accommodate potential depositional hiatuses or erosional discontinuities between successive layers.

The single-grain OSL dating likelihoods have been input into the model as calendar ages before year of sample collection, together with their associated 1σ uncertainty ranges, using the *date* command. The Bayesian model was run using the general *outlier* function (Bronk Ramsey, 2009b), with prior outlier probabilities of 5% assigned to all dating samples. Likelihood estimates that yielded posterior outlier probabilities >5% were not excluded from the final model but were proportionally down-weighted in the iterative Markov Chain Monte Carlo runs (Bronk Ramsey, 2009b).

To examine the sensitivity of the modelling outcomes to different assumptions about stratigraphic priors and dating likelihoods, we have run five different versions of the Gruta do Caldeirão Bayesian model (Models I to V). The structure of these models, and the main differences in representation of individual stratigraphic layers and dating determinations, are summarised in **Table J**. In brief, Model I is set up with separate stratigraphic units defined for each individual layer, with the exception of layers Fa-Fc, which are grouped as a single unit. Model I includes all age determinations depicted in **Fig 23**, together with the radiocarbon determination obtained on the *Semicassis saburon* ornament from layer K (OxA-22301). The radiocarbon determinations for OxA-1938 and OxA-22301 are assumed to represent maximum age estimates for layers Fa-Fc and K, respectively, and have therefore been input into the model using the *After* command. The radiocarbon determination for MAMS-41872 is assumed to represent a minimum age estimate for layer L and has thus been input into the model using the *Before* command. Model II is the same as Model I, except that layers I-Ja and layers L-N are represented as combined stratigraphic units rather than defined as individual units. Model III is the same as Model II, except that the radiocarbon determination for MAMS-33905 is considered as a minimum age estimate for layers L-N and has therefore been input into the model using the *Before* command. Model IV is equivalent to Model III, but all radiocarbon determinations have been removed from the Middle Palaeolithic units (layers L, M, N) to test the extreme assumption that they all suffer from methodological or stratigraphic reliability issues. Layers L, M and N are also represented as separate stratigraphic units rather than as a single combined grouping in Model IV. Model V is the same as Model III but includes the two radiocarbon determinations from the Entrance Trench (MAMS-41874 and MAMS-41876), and additionally adopts a single stratigraphic grouping for layers L, M, N (Back Chamber), and the Middle Palaeolithic layers from the Entrance Trench (Units 5-6). The CQL codes used to construct Models I to V are provided in the next section.

The results obtained for Models I to V are summarised in **Tables K-O** and **Figs M-O, 24-25**. All modelled age ranges have been rounded to the nearest 10 years and are reported as the 68.3 % and 95.4 % highest probability density function (PDF) ranges, as well as the mean and 1σ uncertainty ranges of the modelled posterior distributions. The posterior probabilities of the upper and lower (top and bottom) boundaries have been used to constrain the beginning and end periods for each layer. For comparison, **Tables K-O** also show the modelled age range of each stratigraphic unit, calculated from the modelled posterior probabilities of the lower and upper unit boundaries using the *date* command. **Tables K-O** summarise the convergence

integrals, posterior outlier probabilities and agreement indices for all individual posterior distributions. The agreement indices, including the A_{model} and $A_{overall}$ values, are included for completeness but are of limited diagnostic value as all models have been run with the general *outlier* function.

References

Bronk Ramsey, C., 2009a. Bayesian analysis of radiocarbon dates. Radiocarbon 51, 337–360.

Bronk Ramsey, C., 2009b. Dealing with offsets and outliers in radiocarbon dating. Radiocarbon 51, 1023–1045.

Demuro, M., Arnold, L.J., Aranburu, A., Sala, N., Arsuaga, J.L., 2019. New bracketing luminescence ages constrain the Sima de los Huesos hominin fossils (Atapuerca, Spain) to MIS 12. Journal of Human Evolution 131, 76–95.

Demuro, M., Arnold, L.J., Duval, M., Méndez-Quintas, E., Santonja, M., Pérez-González, A., 2020. Refining the chronology of Acheulean deposits at Porto Maior in the river Miño basin (Galicia, Spain) using a comparative luminescence and ESR dating approach. Quaternary International 556, 96-112.

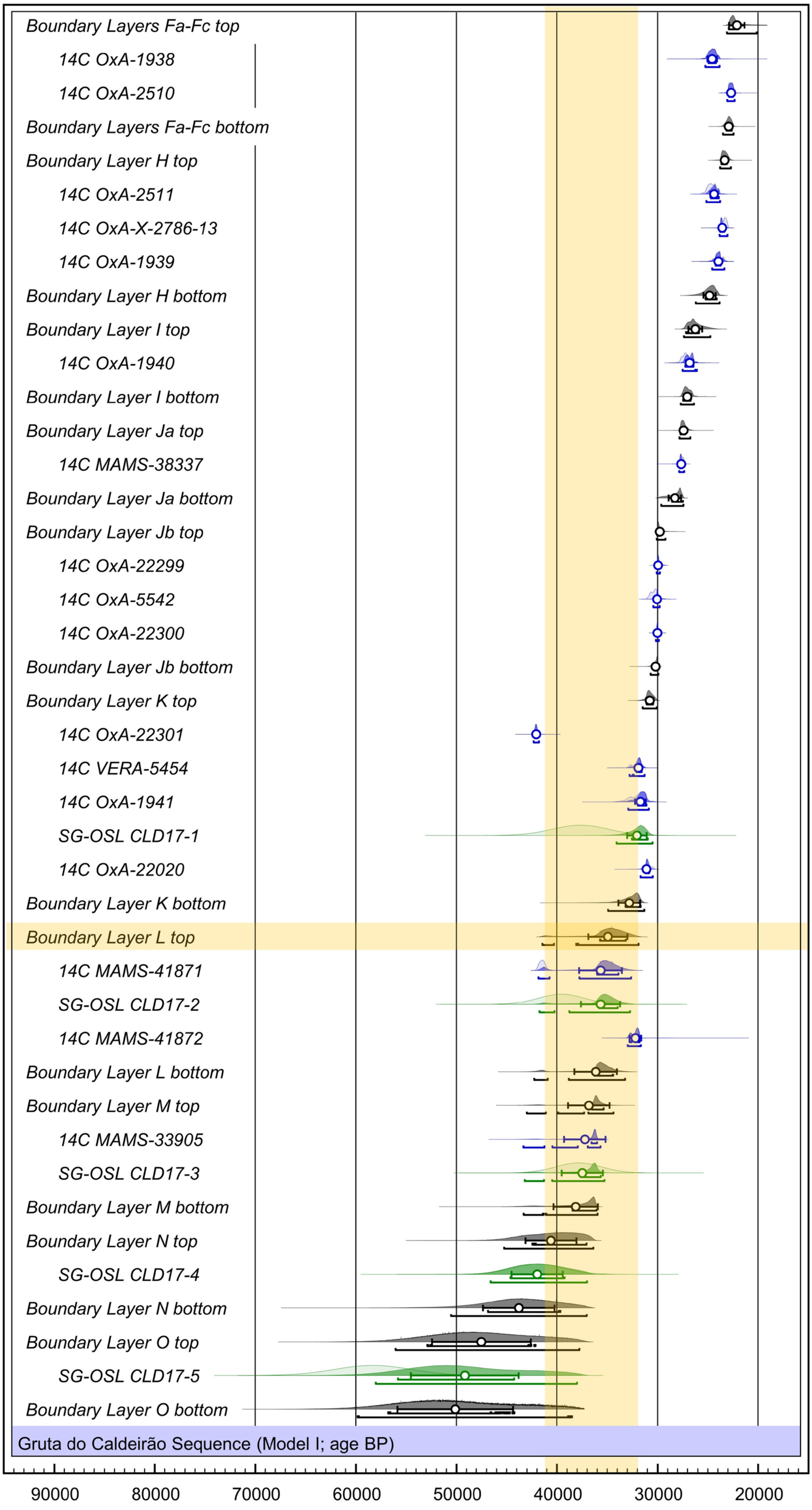

Fig M. OxCal plot output for Model I. The prior age distributions for the dating determinations (likelihoods) are shown as light coloured probability density functions (PDFs): blue = radiocarbon determinations; green = single-grain OSL determinations. The modelled posterior distributions for the dating determinations and stratigraphic unit boundaries are shown as dark coloured and grey PDFs, respectively. Unmodelled and modelled ages are shown on a calendar year timescale, and both are expressed in years before AD1950. The white circles and associated error bars represent the mean ages and 1σ uncertainty ranges of the PDFs. The 68.3% and 95.4% ranges of the highest posterior probabilities are indicated by the horizontal bars underneath the PDFs. The light yellow rectangles highlight the 95.4% interval that the model returns for the end of the Middle Palaeolithic at the site.

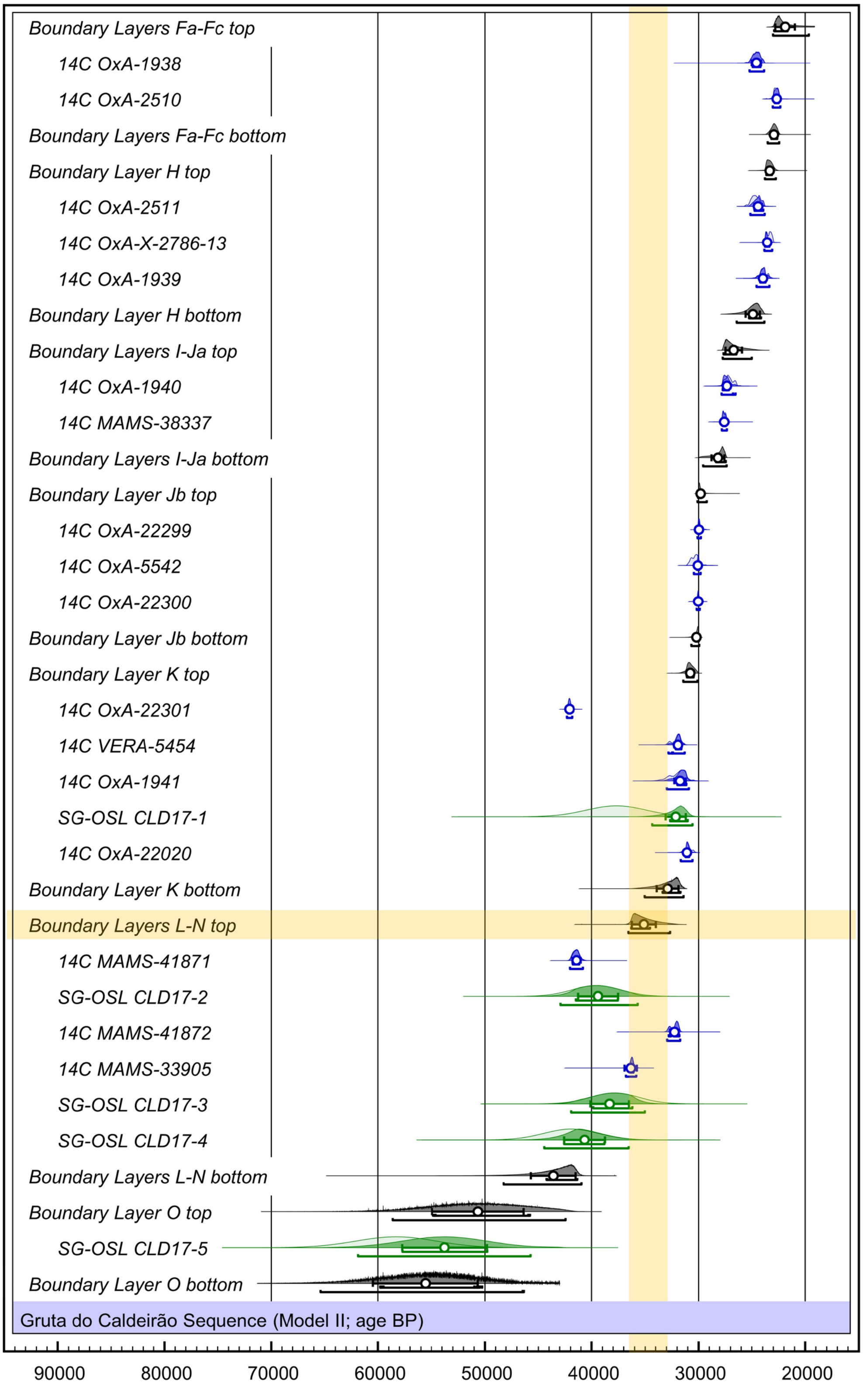

Fig N. OxCal plot output for Model II. The prior age distributions for the dating determinations (likelihoods) are shown as light coloured probability density functions (PDFs): blue = radiocarbon determinations; green = single-grain OSL determinations. The modelled posterior distributions for the dating determinations and stratigraphic unit boundaries are shown as dark coloured and grey PDFs, respectively. Unmodelled and modelled ages are shown on a calendar year timescale, and both are expressed in years before AD1950. The white circles and associated error bars represent the mean ages and 1σ uncertainty ranges of the PDFs. The 68.3% and 95.4% ranges of the highest posterior probabilities are indicated by the horizontal bars underneath the PDFs. The light yellow rectangles highlight the 95.4% interval that the model returns for the end of the Middle Palaeolithic at the site.

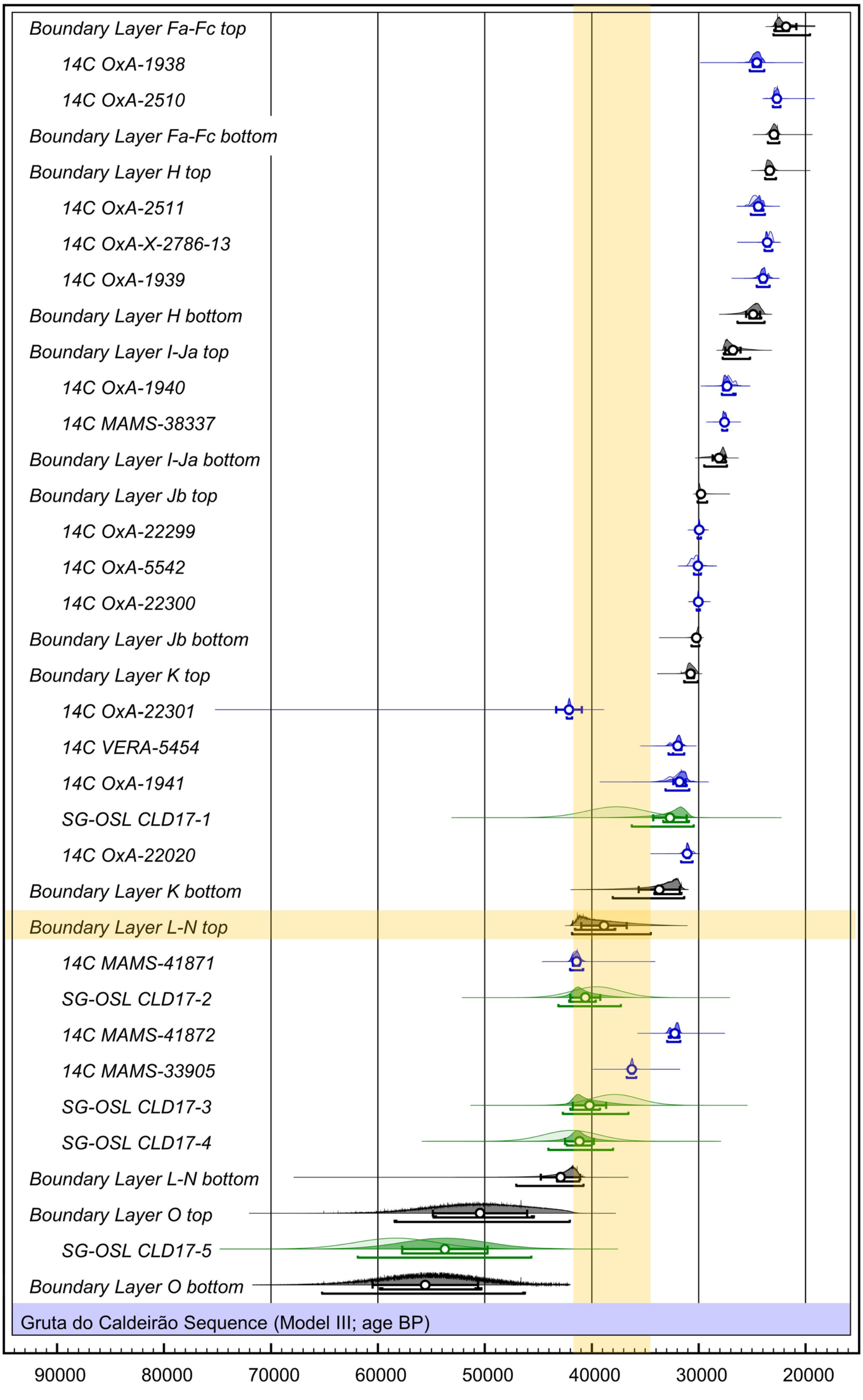

Fig O. OxCal plot output for Model III. The prior age distributions for the dating determinations (likelihoods) are shown as light coloured probability density functions (PDFs): blue = radiocarbon determinations; green = single-grain OSL determinations. The modelled posterior distributions for the dating determinations and stratigraphic unit boundaries are shown as dark coloured and grey PDFs, respectively. Unmodelled and modelled ages are shown on a calendar year timescale, and both are expressed in years before AD1950. The white circles and associated error bars represent the mean ages and 1σ uncertainty ranges of the PDFs. The 68.3% and 95.4% ranges of the highest posterior probabilities are indicated by the horizontal bars underneath the PDFs. The light yellow rectangles highlight the 95.4% interval that the model returns for the end of the Middle Palaeolithic at the site.

Table J. Summary of the different priors and likelihoods used to construct Bayesian Models I-V. The individual units, stratigraphic groupings, radiocarbon determinations and OSL determinations included in each model are shown, together with any additional constraints used to account for methodological or stratigraphic complications with individual likelihoods (see main text for further details). The 95.4% calibrated age ranges of the radiocarbon determinations and the ±2σ age ranges of the OSL determinations are shown for reference.

Model I

Unit	Sample	age (-2σ)	(+2σ)
Fa-Fc	OxA-1938	23874	25178
	OxA-2510	22360	23111
H	OxA-X-2786-13	23036	23759
	OxA-1939	23234	24603
	OxA-2511	23922	25331
I	OxA-1940	26379	27789
Ja	MAMS-38337	27816	27343
Jb	OxA-22299	28659	29160
	OxA-22300	28786	29447
	OxA-5542	29696	31038
K	OxA-22020	30346	31272
	OxA-1941	30446	33311
	CLD17-1	32100	43300
	VERA-5454	31481	32893
	OxA-22301	40916	41682
L	MAMS-41872	31721	32903
	CLD17-2	35000	44200
	MAMS-41871	40886	42028
M	MAMS-33905	35844	36669
	CLD17-3	42500	33300
N	CLD17-4	47000	37000
O	CLD17-5	66000	50800

Model II

Unit	Sample	age (-2σ)	(+2σ)
Fa-Fc	OxA-1938	23874	25178
	OxA-2510	22360	23111
H	OxA-X-2786-13	23036	23759
	OxA-1939	23234	24603
	OxA-2511	23922	25331
I-Ja	OxA-1940	26379	27789
	MAMS-38337	27816	27343
Jb	OxA-22299	28659	29160
	OxA-22300	28786	29447
	OxA-5542	29696	31038
K	OxA-22020	30346	31272
	OxA-1941	30446	33311
	CLD17-1	32100	43300
	VERA-5454	31481	32893
	OxA-22301	40916	41682
L-N	MAMS-41872	31721	32903
	CLD17-2	35000	44200
	MAMS-41871	40886	42028
	MAMS-33905	35844	36669
	CLD17-3	42500	33300
	CLD17-4	47000	37000
O	CLD17-5	66000	50800

Model III

Unit	Sample	age (-2σ)	(+2σ)
Fa-Fc	OxA-1938	23874	25178
	OxA-2510	22360	23111
H	OxA-X-2786-13	23036	23759
	OxA-1939	23234	24603
	OxA-2511	23922	25331
I-Ja	OxA-1940	26379	27789
	MAMS-38337	27816	27343
Jb	OxA-22299	28659	29160
	OxA-22300	28786	29447
	OxA-5542	29696	31038
K	OxA-22020	30346	31272
	OxA-1941	30446	33311
	CLD17-1	32100	43300
	VERA-5454	31481	32893
	OxA-22301	40916	41682
L-N	MAMS-41872	31721	32903
	CLD17-2	35000	44200
	MAMS-41871	40886	42028
	MAMS-33905	35844	36669
	CLD17-3	42500	33300
	CLD17-4	47000	37000
O	CLD17-5	66000	50800

Model IV

Unit	Sample	age (-2σ)	(+2σ)
Fa-Fc	OxA-1938	23874	25178
	OxA-2510	22360	23111
H	OxA-X-2786-13	23036	23759
	OxA-1939	23234	24603
	OxA-2511	23922	25331
I-Ja	OxA-1940	26379	27789
	MAMS-38337	27816	27343
Jb	OxA-22299	28659	29160
	OxA-22300	28786	29447
	OxA-5542	29696	31038
K	OxA-22020	30346	31272
	OxA-1941	30446	33311
	CLD17-1	32100	43300
	VERA-5454	31481	32893
	OxA-22301	40916	41682
L	CLD17-2	35000	44200
M	CLD17-3	42500	33300
N	CLD17-4	47000	37000
O	CLD17-5	66000	50800

Model V

Unit	Sample	age (-2σ)	(+2σ)
Fa-Fc	OxA-1938	23874	25178
	OxA-2510	22360	23111
H	OxA-X-2786-13	23036	23759
	OxA-1939	23234	24603
	OxA-2511	23922	25331
I-Ja	OxA-1940	26379	27789
	MAMS-38337	27816	27343
Jb	OxA-22299	28659	29160
	OxA-22300	28786	29447
	OxA-5542	29696	31038
K	OxA-22020	30346	31272
	OxA-1941	30446	33311
	CLD17-1	32100	43300
	VERA-5454	31481	32893
	OxA-22301	40916	41682
L-N/Units 5-6	MAMS-41872	31721	32903
	CLD17-2	35000	44200
	MAMS-41871	40886	42028
	MAMS-33905	35844	36669
	MAMS-41874	37665	39500
	MAMS-41876	36536	38465
	CLD17-3	42500	33300
	CLD17-4	47000	37000
O	CLD17-5	66000	50800

minimum age ('Before' command)
maximum age ('After' command)

Table K. Summary of Bayesian modelling results for Model I. The likelihood (unmodelled) and posterior (modelled) age ranges are presented for each of the numerical dating samples. Posterior (modelled) ranges are also shown for the boundaries and age of each stratigraphic layer. Posterior ages are presented as the 68.3% and 95.4% highest probability density ranges. The mean and 1σ uncertainty ranges of the modelled posterior distributions are shown for comparison (assuming a normally distributed probability density function). The unmodelled and modelled age estimates have been rounded to the nearest 10 years.

Unit /	Dating	Unmodelled age (years)			Modelled age (years)			Agreement index	Posterior outlier probability	Convergence integral
boundary parameter	sample	68.3% range	95.4% range	Mean ± 1σ	68.3% range	95.4% range	Mean ± 1σ	(A_i) (%)	(%)	(%)
Layer Fa-Fc age (a)					23030 – 22370	23390 – 21260	22540 ± 540			99.9
Boundary Layer Fa-Fc top					22930 – 22030	23110 – 20140	22130 ± 780			99.6
	14C OxA-1938 (b)	24930 – 24210	25200 – 23870	24570 ± 350	24940 – 24200	25260 – 23830	24580 ± 470	102.6	2.4	99.9
	14C OxA-2510	22940 – 22540	23140 – 22360	22750 ± 210	22910 – 22520	23080 – 22350	22700 ± 230	105.9	1.3	99.9
Boundary Layer Fa-Fc bottom					23170 – 22630	23510 – 22450	22940 ± 290			99.9
Layer H age (a)					24490 – 23460	25300 – 23060	24090 ± 570			100
Boundary Layer H top					23660 – 23130	23790 – 22730	23350 ± 290			100
	14C OxA-2511	25050 – 24320	25520 – 23940	24710 ± 360	24710 – 23930	25160 – 23790	24400 ± 360	86.4	3.4	99.9
	14C OxA-X-2786-13	23720 – 23140	23760 – 23040	23400 ± 210	23780 – 23360	23850 – 23060	23590 ± 250	99.4	4	100
	14C OxA-1939	24270 – 23440	24640 – 23240	23960 ± 340	24240 – 23730	24570 – 23360	23970 ± 290	112.6	0.8	100
Boundary Layer H bottom					25230 – 24140	26190 – 23840	24840 ± 600			99.8
Layer I age (a)					27270 – 26360	27550 – 25490	26650 ± 530			99.9
Boundary Layer I top					27160 – 25880	27370 – 24750	26250 ± 690			99.8
	14C OxA-1940	27670 – 26550	27820 – 26380	27140 ± 400	27240 – 26440	27500 – 26090	26820 ± 370	90.9	2.4	100
Boundary Layer I bottom					27440 – 26730	27690 – 26380	27040 ± 370			99.9
Layer Ja age (a)					28050 – 27350	29060 – 27050	27850 ± 480			99.9
Boundary Layer Ja top					27710 – 27260	27820 – 26750	27410 ± 310			99.9
	14C MAMS-38337	27760 – 27470	27820 – 27340	27600 ± 130	27790 – 27530	27860 – 27340	27650 ± 210	105.2	2.2	100
Boundary Layer Ja bottom					28570 – 27490	29640 – 27420	28290 ± 630			99.8
Layer Jb age (a)					30140 – 29880	30490 – 29550	30010 ± 210			99.9
Boundary Layer Jb top					30030 – 29780	30090 – 29240	29800 ± 260			99.8
	14C OxA-22299	30030 – 29840	30100 – 29520	29880 ± 150	30040 – 29920	30100 – 29790	29960 ± 80	118.3	0.4	99.9
	14C OxA-5542	30730 – 30030	31050 – 29770	30360 ± 330	30150 – 29930	30440 – 29800	30070 ± 150	102.5	0.7	99.9
	14C OxA-22300	30110 – 29970	30230 – 29890	30050 ± 80	30080 – 29960	30170 – 29900	30030 ± 70	113.4	0.2	100
Boundary Layer Jb bottom					30270 – 29980	30690 – 29930	30210 ± 210			99.7
Layer K age (a)					32220 – 30890	33570 – 30390	31800 ± 860			99.9
Boundary Layer K top					31100 – 30460	31480 – 30090	30790 ± 350			100
	14C OxA-22301 (b)	42210 – 41990	42310 – 41850	42090 ± 120	42210 – 41980	42330 – 41810	42080 ± 240	102.7	2.3	99.9

Unit / boundary parameter	Dating sample	Unmodelled age (years) 68.3% range	95.4% range	Mean ± 1σ	Modelled age (years) 68.3% range	95.4% range	Mean ± 1σ	Agreement index (A_i) (%)	Posterior outlier probability (%)	Convergence integral (%)
	14C VERA-5454	32230 – 31610	32910 – 31490	32060 ± 370	32110 – 31600	32790 – 31300	31910 ± 340	108.2	1.6	99.9
	14C OxA-1941	32770 – 31090	33600 – 30790	31940 ± 720	31970 – 31130	32920 – 30890	31720 ± 520	122.2	1.1	99.9
	SG-OSL CLD17-1	40570 – 34790	43300 – 32070	37680 ± 2810	32550 – 30980	34070 – 30500	32060 ± 960	21.8	10.3	99.9
	14C OxA-22020	31180 – 30800	31300 – 30350	30930 ± 240	31220 – 30860	31690 – 30490	31110 ± 310	103.4	4.1	100
Boundary Layer K bottom					33180 – 31690	34930 – 31340	32810 ± 1080			99.6
Layer L age (a)					36030 – 33840	41810 – 32530	35560 ± 2000			92.2
Boundary Layer L top					35750 – 33160	41430 – 31910	34950 ± 1940			93.3
	14C MAMS-41871	41800 – 41200	42040 – 40910	41480 ± 290	36040 – 33880	41870 – 32640	35680 ± 2140	14	90.5	84.5
	SG-OSL CLD17-2	41890 – 37220	44090 – 35020	39550 ± 2270	35830 – 33970	41770 – 32750	35680 ± 1950	36	9.9	93.1
	14C MAMS-41872 (c)	32780 – 31790	32910 – 31720	32240 ± 350	32790 – 31790	32970 – 31680	32220 ± 570	101.9	3	99.8
Boundary Layer L bottom					36280 – 34430	42300 – 33240	36170 ± 2110			89.8
Layer M age (a)					37510 – 35680	43270 – 35230	37500 ± 2090			94.6
Boundary Layer M top					36530 – 35390	43020 – 34390	36840 ± 2060			95.6
	14C MAMS-33905	36410 – 36090	36690 – 35860	36250 ± 190	36580 – 36040	43350 – 35690	37240 ± 2060	81.9	20.9	98
	SG-OSL CLD17-3	40180 – 35530	42370 – 33340	37860 ± 2260	37670 – 35680	43220 – 35290	37500 ± 2030	108.5	3.4	94.4
Boundary Layer M bottom					38470 – 36070	43330 – 35970	38160 ± 2200			93.2
Layer N age (a)					44820 – 38680	47730 – 36780	42210 ± 2980			93.3
Boundary Layer N top					42460 – 37070	45260 – 36390	40610 ± 2530			90.6
	SG-OSL CLD17-4	44530 – 39290	47000 – 36820	41910 ± 2540	44630 – 39260	46610 – 37020	41980 ± 2540	100.7	3.6	93.5
Boundary Layer N bottom					46850 – 39690	50550 – 37050	43800 ± 3560			95.1
Layer O age (a)					54920 – 43540	57940 – 38160	48830 ± 5280			97.4
Boundary Layer O top					52890 – 42150	56060 – 37790	47530 ± 4910			96.6
	SG-OSL CLD17-5	62160 – 54460	65860 – 50760	58310 ± 3780	55840 – 44280	58030 – 38010	49170 ± 5340	34.4	33.2	98.1
Boundary Layer O bottom					56790 – 44260	59820 – 38530	50130 ± 5750			88.3
								A_{model} = 26.1		
								$A_{overall}$ = 35.2		

(a) Modelled age ranges of individual layers have been calculated from the posterior probabilities of the upper and lower (top and bottom) boundaries of each stratigraphic layer using the *date* query function in OxCal v4.4.

(b) Modelled as a maximum age estimate using the *After* command in OxCal v4.4.

(c) Modelled as a minimum age estimate using the *Before* command in OxCal v4.4.

Table L. Summary of Bayesian modelling results for Model II. The likelihood (unmodelled) and posterior (modelled) age ranges are presented for each of the numerical dating samples. Posterior (modelled) ranges are also shown for the boundaries and age of each stratigraphic layer. Posterior ages are presented as the 68.3% and 95.4% highest probability density ranges. The mean and 1σ uncertainty ranges of the modelled posterior distributions are shown for comparison (assuming a normally distributed probability density function). The unmodelled and modelled age estimates have been rounded to the nearest 10 years.

Unit /	Dating	Unmodelled age (years)			Modelled age (years)			Agreement index	Posterior outlier probability	Convergence integral
boundary parameter	sample	68.3% range	95.4% range	Mean ± 1σ	68.3% range	95.4% range	Mean ± 1σ	(A_i) (%)	(%)	(%)
Layer Fa-Fc age (a)					23060 – 22250	23380 – 20870	22420 ± 630			99.9
Boundary Layer Fa-Fc top					22930 – 21690	23020 – 19660	21900 ± 930			99.7
	14C OxA-1938 (b)	24930 – 24210	25200 – 23870	24570 ± 350	24940 – 24200	25220 – 23860	24570 ± 430	100.2	4.8	99.9
	14C OxA-2510	22940 – 22540	23140 – 22360	22750 ± 210	22880 – 22490	23060 – 22350	22690 ± 220	102.5	4.5	100
Boundary Layer Fa-Fc bottom					23180 – 22640	23520 – 22460	22950 ± 290			100
Layer H age (a)					24520 – 23460	25420 – 23050	24130 ± 600			100
Boundary Layer H top					23660 – 23140	23780 – 22770	23340 ± 270			100
	14C OxA-2511	25050 – 24320	25520 – 23940	24710 ± 360	24740 – 23940	25130 – 23810	24420 ± 360	85.5	5.5	100
	14C OxA-X-2786-13	23720 – 23140	23760 – 23040	23400 ± 210	23780 – 23370	23830 – 23100	23580 ± 220	96.9	6.4	100
	14C OxA-1939	24270 – 23440	24640 – 23240	23960 ± 340	24240 – 23740	24570 – 23370	23970 ± 290	109.5	4	100
Boundary Layer H bottom					25280 – 24150	26410 – 23850	24910 ± 660			99.9
Layer I-Ja age (a)					27980 – 26960	28950 – 25840	27440 ± 670			100
Boundary Layer I-Ja top					27650 – 26430	27730 – 25020	26700 ± 770			99.9
	14C OxA-1940	27670 – 26550	27820 – 26380	27140 ± 400	27710 – 27150	27820 – 26530	27330 ± 330	104.6	4.1	100
	14C MAMS-38337	27760 – 27470	27820 – 27340	27600 ± 130	27730 – 27440	27810 – 27330	27580 ± 150	99.7	4.1	100
Boundary Layer I-Ja bottom					28390 – 27430	29580 – 27370	28180 ± 620			99.9
Layer Jb age (a)					30140 – 29880	30480 – 29550	30000 ± 210			100
Boundary Layer Jb top					30030 – 29780	30090 – 29240	29800 ± 260			99.9
	14C OxA-22299	30030 – 29840	30100 – 29520	29880 ± 150	30040 – 29920	30100 – 29790	29960 ± 80	114.7	3.7	100
	14C OxA-5542	30730 – 30030	31050 – 29770	30360 ± 330	30150 – 29930	30440 – 29800	30070 ± 150	99.1	4	99.9
	14C OxA-22300	30110 – 29970	30230 – 29890	30050 ± 80	30080 – 29960	30170 – 29900	30030 ± 70	110.1	3.3	100
Boundary Layer Jb bottom					30270 – 29980	30680 – 29930	30210 ± 210			99.9
Layer K age (a)					32260 – 30870	33700 – 30400	31830 ± 850			99.9
Boundary Layer K top					31100 – 30460	31420 – 30090	30780 ± 340			99.9
	14C OxA-22301 (b)	42210 – 41990	42310 – 41850	42090 ± 120	42210 – 41990	42320 – 41840	42090 ± 130	100.8	4.2	100

Unit / boundary parameter	Dating sample	Unmodelled age (years) 68.3% range	95.4% range	Mean ± 1σ	Modelled age (years) 68.3% range	95.4% range	Mean ± 1σ	Agreement index (A_i) (%)	Posterior outlier probability (%)	Convergence integral (%)
	14C VERA-5454	32230 – 31610	32910 – 31490	32060 ± 370	32120 – 31600	32800 – 31300	31920 ± 340	104.9	4.5	100
	14C OxA-1941	32770 – 31090	33600 – 30790	31940 ± 720	31990 – 31130	32950 – 30890	31730 ± 530	117.9	4.2	100
	SG-OSL CLD17-1	40570 – 34790	43300 – 32070	37680 ± 2810	32670 – 31010	34320 – 30560	32130 ± 940	23	5.9	100
	14C OxA-22020	31180 – 30800	31300 – 30350	30930 ± 240	31210 – 30870	31660 – 30580	31090 ± 260	101.5	6	100
Boundary Layer K bottom					33360 – 31680	35060 – 31400	32890 ± 1010			99.8
Layer L-N age (a)					41680 – 36180	44800 – 34150	39360 ± 2790			99.9
Boundary Layer L-N top					36310 – 34540	36560 – 32650	35120 ± 1130			99.9
	14C MAMS-41871	41800 – 41200	42040 – 40910	41480 ± 290	41770 – 41150	42040 – 40820	41420 ± 390	99.1	5.1	99.9
	SG-OSL CLD17-2	41890 – 37220	44090 – 35020	39550 ± 2270	41480 – 37550	42930 – 35720	39400 ± 1860	108.7	4.7	100
	14C MAMS-41872 (c)	32780 – 31790	32910 – 31720	32240 ± 350	32780 – 31790	32930 – 31710	32250 ± 400	100.2	4.8	99.9
	14C MAMS-33905	36410 – 36090	36690 – 35860	36250 ± 190	36430 – 36090	36810 – 35820	36350 ± 580	97.8	6.2	99.9
	SG-OSL CLD17-3	40180 – 35530	42370 – 33340	37860 ± 2260	39860 – 36200	41930 – 35020	38320 ± 1810	109.3	4.7	100
	SG-OSL CLD17-4	44530 – 39290	47000 – 36820	41910 ± 2540	42590 – 38800	44450 – 36540	40660 ± 1920	105.7	4.8	100
Boundary Layer L-N bottom					44220 – 41320	48250 – 40930	43590 ± 2100			99.8
Layer O age (a)					57200 – 48370	61810 – 44280	53110 ± 4450			97.2
Boundary Layer O top					54840 – 45780	58620 – 42450	50660 ± 4290			96.6
	SG-OSL CLD17-5	62160 – 54460	65860 – 50760	58310 ± 3780	57730 – 49800	61880 – 45720	53780 ± 3970	69.4	6.1	99.9
Boundary Layer O bottom					59800 – 50270	65380 – 46340	55570 ± 4900			92.6
								A_{model} = 76.3		
								$A_{overall}$ = 77.8		

(a) Modelled age ranges of individual layers have been calculated from the posterior probabilities of the upper and lower (top and bottom) boundaries of each stratigraphic layer using the *date* query function in OxCal v4.4.

(b) Modelled as a maximum age estimate using the *After* command in OxCal v4.4.

(c) Modelled as a minimum age estimate using the *Before* command in OxCal v4.4.

Table M. Summary of Bayesian modelling results for Model III. The likelihood (unmodelled) and posterior (modelled) age ranges are presented for each of the numerical dating samples. Posterior (modelled) ranges are also shown for the boundaries and age of each stratigraphic layer. Posterior ages are presented as the 68.3% and 95.4% highest probability density ranges. The mean and 1σ uncertainty ranges of the modelled posterior distributions are shown for comparison (assuming a normally distributed probability density function). The unmodelled and modelled age estimates have been rounded to the nearest 10 years.

Unit /	Dating	Unmodelled age (years)			Modelled age (years)			Agreement index	Posterior outlier probability	Convergence integral
boundary parameter	sample	68.3% range	95.4% range	Mean ± 1σ	68.3% range	95.4% range	Mean ± 1σ	(A_i) (%)	(%)	(%)
Layer Fa-Fc age (a)					23070 – 22210	23380 – 20770	22400 ± 660			94.2
Boundary Layer Fa-Fc top					22940 – 21580	23010 – 19580	21830 ± 960			91.4
	14C OxA-1938 (b)	24930 – 24210	25200 – 23870	24570 ± 350	24940 – 24200	25220 – 23860	24570 ± 390	100.3	4.7	99.9
	14C OxA-2510	22940 – 22540	23140 – 22360	22750 ± 210	22880 – 22490	23060 – 22350	22690 ± 220	102.3	4.6	97.4
Boundary Layer Fa-Fc bottom					23190 – 22620	23530 – 22460	22960 ± 290			96.6
Layer H age (a)					24520 – 23460	25410 – 23060	24130 ± 600			100
Boundary Layer H top					23670 – 23150	23780 – 22770	23350 ± 270			99.9
	14C OxA-2511	25050 – 24320	25520 – 23940	24710 ± 360	24740 – 23940	25130 – 23810	24420 ± 360	85.6	5.5	99.9
	14C OxA-X-2786-13	23720 – 23140	23760 – 23040	23400 ± 210	23780 – 23370	23840 – 23090	23580 ± 220	96.7	6.5	100
	14C OxA-1939	24270 – 23440	24640 – 23240	23960 ± 340	24240 – 23740	24570 – 23370	23970 ± 290	109.5	4.1	100
Boundary Layer H bottom					25280 – 24140	26360 – 23840	24910 ± 650			99.8
Layer I-Ja age (a)					27930 – 27040	28840 – 25990	27450 ± 610			99.8
Boundary Layer I-Ja top					27660 – 26590	27740 – 25190	26800 ± 720			99.6
	14C OxA-1940	27670 – 26550	27820 – 26380	27140 ± 400	27710 – 27180	27830 – 26540	27350 ± 320	104.6	4.2	99.9
	14C MAMS-38337	27760 – 27470	27820 – 27340	27600 ± 130	27730 – 27440	27810 – 27330	27570 ± 140	99.5	4.1	99.9
Boundary Layer I-Ja bottom					28270 – 27430	29470 – 27350	28110 ± 580			99.7
Layer Jb age (a)					30150 – 29880	30490 – 29530	30000 ± 220			99.9
Boundary Layer Jb top					30030 – 29770	30090 – 29200	29790 ± 280			99.8
	14C OxA-22299	30030 – 29840	30100 – 29520	29880 ± 150	30040 – 29920	30100 – 29790	29960 ± 80	114.6	3.7	100
	14C OxA-5542	30730 – 30030	31050 – 29770	30360 ± 330	30150 – 29930	30440 – 29790	30070 ± 150	99.3	4.1	99.9
	14C OxA-22300	30110 – 29970	30230 – 29890	30050 ± 80	30080 – 29960	30170 – 29900	30030 ± 70	109.7	3.4	99.9
Boundary Layer Jb bottom					30280 – 29980	30680 – 29930	30210 ± 210			99.8
Layer K age (a)					32590 – 30780	35340 – 30300	32220 ± 1390			97.8
Boundary Layer K top					31080 – 30440	31360 – 30070	30750 ± 330			99.3
	14C OxA-22301 (b)	42210 – 41990	42310 – 41850	42090 ± 120	42210 – 41990	42320 – 41830	42130 ± 1210	100.1	4.8	99.8

Unit / boundary parameter	Dating sample	Unmodelled age (years) 68.3% range	95.4% range	Mean ± 1σ	Modelled age (years) 68.3% range	95.4% range	Mean ± 1σ	Agreement index (A_i) (%)	Posterior outlier probability (%)	Convergence integral (%)
	14C VERA-5454	32230 – 31610	32910 – 31490	32060 ± 370	32150 – 31610	32820 – 31350	31960 ± 350	104.2	4.5	99.3
	14C OxA-1941	32770 – 31090	33600 – 30790	31940 ± 720	32060 – 31110	33090 – 30880	31790 ± 580	114.1	4.3	98.9
	SG-OSL CLD17-1	40570 – 34790	43300 – 32070	37680 ± 2810	33310 – 30900	36260 – 30470	32680 ± 1570	34.9	5.7	99.9
	14C OxA-22020	31180 – 30800	31300 – 30350	30930 ± 240	31200 – 30870	31640 – 30560	31080 ± 260	103.3	5.7	99.6
Boundary Layer K bottom					34140 – 31620	38040 – 31350	33690 ± 1920			96.8
Layer L-N age (a)					42360 – 39600	44570 – 36280	40880 ± 1870			94.5
Boundary Layer L-N top					41580 – 37820	41840 – 34490	38850 ± 2120			92.8
	14C MAMS-41871	41800 – 41200	42040 – 40910	41480 ± 290	41750 – 41140	42020 – 40810	41420 ± 340	99.4	4.7	98.7
	SG-OSL CLD17-2	41890 – 37220	44090 – 35020	39550 ± 2270	42090 – 39620	43110 – 37280	40600 ± 1400	110.2	4.7	99.7
	14C MAMS-41872 (c)	32780 – 31790	32910 – 31720	32240 ± 350	32780 – 31790	32930 – 31710	32240 ± 390	100.2	4.8	99.9
	14C MAMS-33905 (c)	36410 – 36090	36690 – 35860	36250 ± 190	36410 – 36080	36720 – 35830	36250 ± 250	100.2	4.8	99.9
	SG-OSL CLD17-3	40180 – 35530	42370 – 33340	37860 ± 2260	42000 – 39220	42700 – 36580	40210 ± 1550	76	5	99.7
	SG-OSL CLD17-4	44530 – 39290	47000 – 36820	41910 ± 2540	42340 – 40000	44070 – 38010	41150 ± 1350	122.5	4.7	99.8
Boundary Layer L-N bottom					43260 – 41170	47050 – 40780	42910 ± 1840			94.8
Layer O age (a)					57200 – 48250	61820 – 44130	53010 ± 4510			98.4
Boundary Layer O top					54800 – 45420	58430 – 42060	50450 ± 4400			97.8
	SG-OSL CLD17-5	62160 – 54460	65860 – 50760	58310 ± 3780	57730 – 49760	61880 – 45640	53740 ± 3990	68.9	6	99.9
Boundary Layer O bottom					59800 – 50320	65220 – 46250	55570 ± 4930			94.1
								A_{model} = 78.2		
								$A_{overall}$ = 81.1		

(a) Modelled age ranges of individual layers have been calculated from the posterior probabilities of the upper and lower (top and bottom) boundaries of each stratigraphic layer using the *date* query function in OxCal v4.4.

(b) Modelled as a maximum age estimate using the *After* command in OxCal v4.4.

(c) Modelled as a minimum age estimate using the *Before* command in OxCal v4.4.

Table N. Summary of Bayesian modelling results for Model IV. The likelihood (unmodelled) and posterior (modelled) age ranges are presented for each of the numerical dating samples. Posterior (modelled) ranges are also shown for the boundaries and age of each stratigraphic layer. Posterior ages are presented as the 68.3% and 95.4% highest probability density ranges. The mean and 1σ uncertainty ranges of the modelled posterior distributions are shown for comparison (assuming a normally distributed probability density function). The unmodelled and modelled age estimates have been rounded to the nearest 10 years.

Unit / boundary parameter	Dating sample	Unmodelled age (years) 68.3% range	Unmodelled age (years) 95.4% range	Unmodelled age (years) Mean ± 1σ	Modelled age (years) 68.3% range	Modelled age (years) 95.4% range	Modelled age (years) Mean ± 1σ	Agreement index (A_i) (%)	Posterior outlier probability (%)	Convergence integral (%)
Layer Fa-Fc age (a)					23050 – 22300	23390 – 21030	22470 ± 590			99.9
Boundary Layer Fa-Fc top					22930 – 21830	23060 – 19820	21990 ± 870			99.7
	14C OxA-1938 (b)	24930 – 24210	25200 – 23870	24570 ± 350	24940 – 24210	25220 – 23860	24570 ± 380	100.4	4.6	99.9
	14C OxA-2510	22940 – 22540	23140 – 22360	22750 ± 210	22890 – 22500	23060 – 22350	22700 ± 210	102.7	4.3	100
Boundary Layer Fa-Fc bottom					23180 – 22640	23510 – 22460	22950 ± 280			99.9
Layer H age (a)					24530 – 23460	25450 – 23050	24140 ± 610			100
Boundary Layer H top					23660 – 23140	23780 – 22770	23340 ± 270			100
	14C OxA-2511	25050 – 24320	25520 – 23940	24710 ± 360	24750 – 23950	25130 – 23810	24430 ± 360	85.9	5.4	100
	14C OxA-X-2786-13	23720 – 23140	23760 – 23040	23400 ± 210	23780 – 23370	23830 – 23100	23580 ± 220	97	6.4	100
	14C OxA-1939	24270 – 23440	24640 – 23240	23960 ± 340	24240 – 23740	24570 – 23370	23970 ± 290	109.5	4	100
Boundary Layer H bottom					25300 – 24140	26460 – 23840	24930 ± 680			99.9
Layer I-Ja age (a)					27920 – 27040	28840 – 25980	27460 ± 610			99.9
Boundary Layer I-Ja top					27650 – 26590	27740 – 25180	26810 ± 720			99.8
	14C OxA-1940	27670 – 26550	27820 – 26380	27140 ± 400	27710 – 27180	27840 – 26540	27350 ± 320	104.6	4.1	99.9
	14C MAMS-38337	27760 – 27470	27820 – 27340	27600 ± 130	27730 – 27440	27810 – 27330	27570 ± 140	99.6	4.1	100
Boundary Layer I-Ja bottom					28260 – 27430	29480 – 27350	28100 ± 580			99.9
Layer Jb age (a)					30140 – 29880	30480 – 29540	30000 ± 210			99.9
Boundary Layer Jb top					30030 – 29770	30090 – 29230	29800 ± 270			99.8
	14C OxA-22299	30030 – 29840	30100 – 29520	29880 ± 150	30040 – 29920	30100 – 29790	29960 ± 80	114.7	3.6	100
	14C OxA-5542	30730 – 30030	31050 – 29770	30360 ± 330	30150 – 29930	30440 – 29790	30070 ± 150	99.1	4	99.9
	14C OxA-22300	30110 – 29970	30230 – 29890	30050 ± 80	30080 – 29960	30170 – 29900	30030 ± 70	110.1	3.3	100
Boundary Layer Jb bottom					30270 – 29980	30680 – 29930	30210 ± 210			99.8
Layer K age (a)					32280 – 30860	33940 – 30360	31880 ± 940			100
Boundary Layer K top					31100 – 30460	31420 – 30090	30780 ± 340			100
	14C OxA-22301 (b)	42210 – 41990	42310 – 41850	42090 ± 120	42210 – 41980	42320 – 41830	42090 ± 200	100.3	4.6	99.9

Unit / boundary parameter	Dating sample	Unmodelled age (years) 68.3% range	Unmodelled age (years) 95.4% range	Unmodelled age (years) Mean ± 1σ	Modelled age (years) 68.3% range	Modelled age (years) 95.4% range	Modelled age (years) Mean ± 1σ	Agreement index (A_i) (%)	Posterior outlier probability (%)	Convergence integral (%)
	14C VERA-5454	32230 – 31610	32910 – 31490	32060 ± 370	32120 – 31600	32800 – 31310	31920 ± 340	104.7	4.6	99.9
	14C OxA-1941	32770 – 31090	33600 – 30790	31940 ± 720	31990 – 31120	32970 – 30890	31730 ± 530	117.6	4.2	100
	SG-OSL CLD17-1	40570 – 34790	43300 – 32070	37680 ± 2810	32680 – 31000	34700 – 30490	32190 ± 1060	24.7	5.9	100
	14C OxA-22020	31180 – 30800	31300 – 30350	30930 ± 240	31210 – 30870	31660 – 30580	31090 ± 270	101.6	5.9	99.9
Boundary Layer K bottom					33400 – 31660	35610 – 31340	32980 ± 1200			99.8
Layer L age (a)					38490 – 34870	39890 – 33320	36630 ± 1670			99.7
Boundary Layer L top					37310 – 33600	38910 – 32200	35590 ± 1770			99.5
	SG-OSL CLD17-2	41890 – 37220	44090 – 35020	39550 ± 2270	38490 – 35290	40010 – 33770	36890 ± 1550	73.4	5.2	99.9
Boundary Layer L bottom					39320 – 35990	40930 – 34370	37660 ± 1640			99.7
Layer M age (a)					41260 – 37940	43040 – 36330	39670 ± 1670			99.7
Boundary Layer M top					40490 – 37240	42150 – 35620	38880 ± 1630			99.6
	SG-OSL CLD17-3	40180 – 35530	42370 – 33340	37860 ± 2260	41120 – 37910	42700 – 36400	39540 ± 1560	97	4.8	100
Boundary Layer M bottom					42140 – 38540	44110 – 36930	40450 ± 1800			99.5
Layer N age (a)					46000 – 41360	49270 – 39400	44100 ± 2490			99.8
Boundary Layer N top					44300 – 40310	46480 – 38520	42430 ± 2000			99.8
	SG-OSL CLD17-4	44530 – 39290	47000 – 36820	41910 ± 2540	45450 – 41500	47530 – 39770	43600 ± 1950	99.1	4.9	100
Boundary Layer N bottom					47880 – 42230	52290 – 40180	45770 ± 3080			99.4
Layer O age (a)					55990 – 47950	60430 – 44400	52250 ± 4020			99.1
Boundary Layer O top					54240 – 46370	58200 – 43120	50620 ± 3880			98.6
	SG-OSL CLD17-5	62160 – 54460	65860 – 50760	58310 ± 3780	56410 – 48550	60490 – 45000	52660 ± 3830	57.1	6.6	99.9
Boundary Layer O bottom					58090 – 49060	62690 – 45660	53890 ± 4350			94.7
								A_{model} = 63.1		
								$A_{overall}$ = 65.7		

(a) Modelled age ranges of individual layers have been calculated from the posterior probabilities of the upper and lower (top and bottom) boundaries of each stratigraphic layer using the *date* query function in OxCal v4.4.

(b) Modelled as a maximum age estimate using the *After* command in OxCal v4.4.

(c) Modelled as a minimum age estimate using the *Before* command in OxCal v4.4.

Table O. Summary of Bayesian modelling results for Model V. The likelihood (unmodelled) and posterior (modelled) age ranges are presented for each of the numerical dating samples. Posterior (modelled) ranges are also shown for the boundaries and age of each stratigraphic layer. Posterior ages are presented as the 68.3% and 95.4% highest probability density ranges. The mean and 1σ uncertainty ranges of the modelled posterior distributions are shown for comparison (assuming a normally distributed probability density function). The unmodelled and modelled age estimates have been rounded to the nearest 10 years.

Unit /	Dating	Unmodelled age (years)			Modelled age (years)			Agreement index	Posterior outlier probability	Convergence integral
boundary parameter	sample	68.3% range	95.4% range	Mean ± 1σ	68.3% range	95.4% range	Mean ± 1σ	(A_i) (%)	(%)	(%)
Layer Fa-Fc age [a]					23050 – 22280	23370 – 20880	22440 ± 630			99.9
Boundary Layer Fa-Fc top					22930 – 21760	23030 – 19630	21930 ± 930			99.7
	14C OxA-1938 [b]	24930 – 24210	25200 – 23870	24570 ± 350	24940 – 24210	25220 – 23860	24570 ± 400	100.3	4.7	99.9
	14C OxA-2510	22940 – 22540	23140 – 22360	22750 ± 210	22880 – 22500	23060 – 22350	22690 ± 210	102.5	4.4	100
Boundary Layer Fa-Fc bottom					23170 – 22630	23510 – 22450	22950 ± 280			99.9
Layer H age [a]					24540 – 23450	25470 – 23040	24140 ± 620			100
Boundary Layer H top					23660 – 23130	23780 – 22760	23340 ± 280			100
	14C OxA-2511	25050 – 24320	25520 – 23940	24710 ± 360	24750 – 23950	25140 – 23810	24430 ± 360	86	5.5	100
	14C OxA-X-2786-13	23720 – 23140	23760 – 23040	23400 ± 210	23780 – 23360	23830 – 23090	23580 ± 220	97	6.4	100
	14C OxA-1939	24270 – 23440	24640 – 23240	23960 ± 340	24240 – 23740	24570 – 23370	23970 ± 290	109.3	4.1	100
Boundary Layer H bottom					25310 – 24140	26500 – 23850	24940 ± 680			99.9
Layer I-Ja age [a]					27920 – 27050	28820 – 26010	27460 ± 600			99.9
Boundary Layer I-Ja top					27650 – 26610	27740 – 25220	26820 ± 710			99.8
	14C OxA-1940	27670 – 26550	27820 – 26380	27140 ± 400	27710 – 27180	27840 – 26550	27360 ± 310	104.6	4.1	100
	14C MAMS-38337	27760 – 27470	27820 – 27340	27600 ± 130	27730 – 27440	27810 – 27330	27570 ± 140	99.6	4.1	100
Boundary Layer I-Ja bottom					28250 – 27430	29440 – 27350	28090 ± 570			99.9
Layer Jb age [a]					30150 – 29880	30500 – 29520	30000 ± 220			99.9
Boundary Layer Jb top					30030 – 29760	30090 – 29180	29790 ± 280			99.8
	14C OxA-22299	30030 – 29840	30100 – 29520	29880 ± 150	30040 – 29910	30100 – 29790	29960 ± 90	114.6	3.7	100
	14C OxA-5542	30730 – 30030	31050 – 29770	30360 ± 330	30160 – 29930	30450 – 29790	30070 ± 150	99.5	4	99.9
	14C OxA-22300	30110 – 29970	30230 – 29890	30050 ± 80	30080 – 29960	30170 – 29900	30030 ± 70	109.7	3.4	100
Boundary Layer Jb bottom					30280 – 29980	30690 – 29930	30210 ± 210			99.9
Layer K age [a]					32420 – 30820	34380 – 30360	32000 ± 1050			99.8
Boundary Layer K top					31090 – 30450	31390 – 30080	30760 ± 330			99.9
	14C OxA-22301 [b]	42210 – 41990	42310 – 41850	42090 ± 120	42210 – 41980	42320 – 41830	42090 ± 170	100.5	4.5	99.9

Unit / boundary parameter	Dating sample	Unmodelled age (years) 68.3% range	Unmodelled age (years) 95.4% range	Unmodelled age (years) Mean ± 1σ	Modelled age (years) 68.3% range	Modelled age (years) 95.4% range	Modelled age (years) Mean ± 1σ	Agreement index (A_i) (%)	Posterior outlier probability (%)	Convergence integral (%)
	14C VERA-5454	32230 – 31610	32910 – 31490	32060 ± 370	32140 – 31600	32810 – 31330	31950 ± 350	104.5	4.5	99.9
	14C OxA-1941	32770 – 31090	33600 – 30790	31940 ± 720	32030 – 31120	33040 – 30880	31770 ± 560	115.5	4.2	99.9
	SG-OSL CLD17-1	40570 – 34790	43300 – 32070	37680 ± 2810	32970 – 30950	35110 – 30560	32380 ± 1190	28.4	5.8	100
	14C OxA-22020	31180 – 30800	31300 – 30350	30930 ± 240	31210 – 30870	31650 – 30560	31090 ± 270	102.4	5.9	99.9
Boundary Layer K bottom					33750 – 31640	36190 – 31390	33240 ± 1340			99.5
Layer L-N + Unit 5-6 age [a]					41590 – 37560	43790 – 35480	39620 ± 2090			99.9
Boundary Layer L-N + Unit 5-6 top					37900 – 35780	38640 – 33880	36520 ± 1190			99.8
	14C MAMS-41874	39350 – 38370	39520 – 37670	38730 ± 500	39350 – 38390	39540 – 37680	38750 ± 510	101.7	4.6	99.9
	14C MAMS-41876	37690 – 36880	38530 – 36550	37430 ± 480	38230 – 36960	38950 – 36760	37680 ± 600	86	5.2	99.9
	14C MAMS-41871	41800 – 41200	42040 – 40910	41480 ± 290	41740 – 41110	42060 – 40690	41340 ± 510	96	6.6	99.9
	SG-OSL CLD17-2	41890 – 37220	44090 – 35020	39550 ± 2270	41250 – 37920	42610 – 36490	39550 ± 1570	116.4	4.7	100
	14C MAMS-41872 [c]	32780 – 31790	32910 – 31720	32240 ± 350	32790 – 31790	32930 – 31710	32240 ± 420	100.1	4.8	99.8
	14C MAMS-33905 [c]	36410 – 36090	36690 – 35860	36250 ± 190	36410 – 36090	36710 – 35830	36250 ± 240	100.3	4.6	99.9
	SG-OSL CLD17-3	40180 – 35530	42370 – 33340	37860 ± 2260	40280 – 37120	41930 – 35900	38820 ± 1520	111.1	4.7	100
	SG-OSL CLD17-4	44530 – 39290	47000 – 36820	41910 ± 2540	42030 – 38850	43610 – 37070	40430 ± 1630	106.3	4.8	100
Boundary Layer L-N + Unit 5-6 bottom					43230 – 41210	46900 – 40680	42730 ± 1600			99.7
Layer O age [a]					57130 – 48100	61730 – 43960	52880 ± 4540			97.3
Boundary Layer O top					54640 – 45170	58370 – 41980	50300 ± 4410			96.8
	SG-OSL CLD17-5	62160 – 54460	65860 – 50760	58310 ± 3780	57350 – 49570	61810 – 45490	53630 ± 4030	67.9	6.2	99.9
Boundary Layer O bottom					59870 – 50220	65390 – 46130	55460 ± 4950			93.3
								A_{model} = 78.5		
								$A_{overall}$ = 80.5		

(a) Modelled age ranges of individual layers have been calculated from the posterior probabilities of the upper and lower (top and bottom) boundaries of each stratigraphic layer using the *date* query function in OxCal v4.4.

(b) Modelled as a maximum age estimate using the *After* command in OxCal v4.4.

(c) Modelled as a minimum age estimate using the *Before* command in OxCal v4.4.

Bayesian model CQL code

Model I

```
Plot()
 {
  Outlier_Model("General",T(5),U(0,4),"t");
  Sequence("Gruta do Caldeirão Sequence")
  {
   Boundary("Boundary Layer O bottom");
   Date("SG-OSL CLD17-5", N(2017-58376,3777))
   {
    Outlier("General", 0.05);
    color="green";
   };
   Boundary("Boundary Layer O top");
   Boundary("Boundary Layer N bottom");
   Date("SG-OSL CLD17-4", N(2017-41978,2544))
   {
    Outlier("General", 0.05);
    color="green";
   };
   Boundary("Boundary Layer N top");
   Boundary("Boundary Layer M bottom");
   Phase("Layer M")
   {
    Date("SG-OSL CLD17-3", N(2017-37921,2258))
    {
     Outlier("General", 0.05);
     color="green";
    };
    R_Date("14C MAMS-33905", 31900, 170)
    {
     Outlier("General", 0.05);
     color="mediumblue";
    };
   };
   Boundary("Boundary Layer M top");
   Boundary("Boundary Layer L bottom");
   Phase("Layer L")
   {
    Before()
    {
     R_Date("14C MAMS-41872", 28150, 160)
     {
      Outlier("General", 0.05);
      color="mediumblue";
     };
    };
    Date("SG-OSL CLD17-2", N(2017-39618,2268))
    {
     Outlier("General", 0.05);
     color="green";
    };
    R_Date("14C MAMS-41871", 36490, 390)
    {
     Outlier("General", 0.05);
     color="mediumblue";
    };
   };
   Boundary("Boundary Layer L top");
   Boundary("Boundary Layer K bottom");
```

```
Phase("Layer K")
{
 R_Date("14C OxA-22020", 26790, 260)
 {
  Outlier("General", 0.05);
  color="mediumblue";
 };
 Date("SG-OSL CLD17-1", N(2017-37747,2808))
 {
  Outlier("General", 0.05);
  color="green";
 };
 R_Date("14C OxA-1941", 27600, 600)
 {
  Outlier("General", 0.05);
  color="mediumblue";
 };
 R_Date("14C VERA-5454", 28000, 210)
 {
  Outlier("General", 0.05);
  color="mediumblue";
 };
 After()
 {
  R_Date("14C OxA-22301", 37500, 230)
  {
   Outlier("General", 0.05);
   color="mediumblue";
  };
 };
};
Boundary("Boundary Layer K top");
Boundary("Boundary Layer Jb bottom");
Phase("Layer Jb")
{
 R_Date("14C OxA-22300", 25750, 110)
 {
  Outlier("General", 0.05);
  color="mediumblue";
 };
 R_Date("14C OxA-5542", 26020, 320)
 {
  Outlier("General", 0.05);
  color="mediumblue";
 };
 R_Date("14C OxA-22299", 25560, 100)
 {
  Outlier("General", 0.05);
  color="mediumblue";
 };
};
Boundary("Boundary Layer Jb top");
Boundary("Boundary Layer Ja bottom");
R_Date("14C MAMS-38337", 23437, 140)
{
 Outlier("General", 0.05);
 color="mediumblue";
};
Boundary("Boundary Layer Ja top");
```

```
  Boundary("Boundary Layer I bottom");
  R_Date("14C OxA-1940", 22900, 380)
  {
   Outlier("General", 0.05);
   color="mediumblue";
  };
  Boundary("Boundary Layer I top");
  Boundary("Boundary Layer H bottom");
  Phase("Layer H")
  {
   R_Date("14C OxA-1939", 19900, 260)
   {
    Outlier("General", 0.05);
    color="mediumblue";
   };
   R_Date("14C OxA-X-2786-13", 19400, 150)
   {
    Outlier("General", 0.05);
    color="mediumblue";
   };
   R_Date("14C OxA-2511", 20530, 270)
   {
    Outlier("General", 0.05);
    color="mediumblue";
   };
  };
  Boundary("Boundary Layer H top");
  Boundary("Boundary Layer Fa-Fc bottom");
  Phase("Layer Fa-Fc")
  {
   R_Date("14C OxA-2510", 18840, 200)
   {
    Outlier("General", 0.05);
    color="mediumblue";
   };
   After()
   {
    R_Date("14C OxA-1938", 20400, 270)
    {
     Outlier("General", 0.05);
     color="mediumblue";
    };
   };
  };
  Boundary("Boundary Layer Fa-Fc top");
 };
};
```

Model II

```
Plot()
 {
  Outlier_Model("General",T(5),U(0,4),"t");
  Sequence("Gruta do Caldeirão Sequence")
  {
   Boundary("Boundary Layer O bottom");
   Date("SG-OSL CLD17-5", N(2017-58376,3777))
   {
    Outlier("General", 0.05);
    color="green";
   };
   Boundary("Boundary Layer O top");
   Boundary("Boundary Layer L-N bottom");
   Phase("Layer L-N")
   {
    Date("SG-OSL CLD17-4", N(2017-41978,2544))
    {
     Outlier("General", 0.05);
     color="green";
    };
    Date("SG-OSL CLD17-3", N(2017-37921,2258))
    {
     Outlier("General", 0.05);
     color="green";
    };
    R_Date("14C MAMS-33905", 31900, 170)
    {
     Outlier("General", 0.05);
     color="mediumblue";
    };
    Before()
    {
     R_Date("14C MAMS-41872", 28150, 160)
     {
      Outlier("General", 0.05);
      color="mediumblue";
     };
    };
    Date("SG-OSL CLD17-2", N(2017-39618,2268))
    {
     Outlier("General", 0.05);
     color="green";
    };
    R_Date("14C MAMS-41871", 36490, 390)
    {
     Outlier("General", 0.05);
     color="mediumblue";
    };
   };
   Boundary("Boundary Layer L-N top");
   Boundary("Boundary Layer K bottom");
   Phase("Layer K")
   {
    R_Date("14C OxA-22020", 26790, 260)
    {
     Outlier("General", 0.05);
     color="mediumblue";
```

```
  };
  Date("SG-OSL CLD17-1", N(2017-37747,2808))
  {
   Outlier("General", 0.05);
   color="green";
  };
  R_Date("14C OxA-1941", 27600, 600)
  {
   Outlier("General", 0.05);
   color="mediumblue";
  };
  R_Date("14C VERA-5454", 28000, 210)
  {
   Outlier("General", 0.05);
   color="mediumblue";
  };
  After()
  {
   R_Date("14C OxA-22301", 37500, 230)
   {
    Outlier("General", 0.05);
    color="mediumblue";
   };
  };
 };
 Boundary("Boundary Layer K top");
 Boundary("Boundary Layer Jb bottom");
 Phase("Layer Jb")
 {
  R_Date("14C OxA-22300", 25750, 110)
  {
   Outlier("General", 0.05);
   color="mediumblue";
  };
  R_Date("14C OxA-5542", 26020, 320)
  {
   Outlier("General", 0.05);
   color="mediumblue";
  };
  R_Date("14C OxA-22299", 25560, 100)
  {
   Outlier("General", 0.05);
   color="mediumblue";
  };
 };
 Boundary("Boundary Layer Jb top");
 Boundary("Boundary Layer I-Ja bottom");
 Phase("Layer I-Ja")
 {
  R_Date("14C MAMS-38337", 23437, 140)
  {
   Outlier("General", 0.05);
   color="mediumblue";
  };
  R_Date("14C OxA-1940", 22900, 380)
  {
   Outlier("General", 0.05);
   color="mediumblue";
  };
```

```
   };
   Boundary("Boundary Layer I-Ja top");
   Boundary("Boundary Layer H bottom");
   Phase("Layer H")
   {
    R_Date("14C OxA-1939", 19900, 260)
    {
     Outlier("General", 0.05);
     color="mediumblue";
    };
    R_Date("14C OxA-X-2786-13", 19400, 150)
    {
     Outlier("General", 0.05);
     color="mediumblue";
    };
    R_Date("14C OxA-2511", 20530, 270)
    {
     Outlier("General", 0.05);
     color="mediumblue";
    };
   };
   Boundary("Boundary Layer H top");
   Boundary("Boundary Layer Fa-Fc bottom");
   Phase("Layer Fa-Fc")
   {
    R_Date("14C OxA-2510", 18840, 200)
    {
     Outlier("General", 0.05);
     color="mediumblue";
    };
    After()
    {
     R_Date("14C OxA-1938", 20400, 270)
     {
      Outlier("General", 0.05);
      color="mediumblue";
     };
    };
   };
   Boundary("Boundary Layer Fa-Fc top");
  };
 };
```

Model III

```
Plot()
 {
  Outlier_Model("General",T(5),U(0,4),"t");
  Sequence("Gruta do Caldeirão Sequence")
  {
   Boundary("Boundary Layer O bottom");
   Date("SG-OSL CLD17-5", N(2017-58376,3777))
   {
    Outlier("General", 0.05);
    color="green";
   };
   Boundary("Boundary Layer O top");
   Boundary("Boundary Layer L-N bottom");
   Phase("Layer L-N")
   {
    Date("SG-OSL CLD17-4", N(2017-41978,2544))
    {
     Outlier("General", 0.05);
     color="green";
    };
    Date("SG-OSL CLD17-3", N(2017-37921,2258))
    {
     Outlier("General", 0.05);
     color="green";
    };
    Before()
    {
     R_Date("14C MAMS-33905", 31900, 170)
     {
      Outlier("General", 0.05);
      color="mediumblue";
     };
    };
    Before()
    {
     R_Date("14C MAMS-41872", 28150, 160)
     {
      Outlier("General", 0.05);
      color="mediumblue";
     };
    };
    Date("SG-OSL CLD17-2", N(2017-39618,2268))
    {
     Outlier("General", 0.05);
     color="green";
    };
    R_Date("14C MAMS-41871", 36490, 390)
    {
     Outlier("General", 0.05);
     color="mediumblue";
    };
   };
   Boundary("Boundary Layer L-N top");
   Boundary("Boundary Layer K bottom");
   Phase("Layer K")
   {
    R_Date("14C OxA-22020", 26790, 260)
```

```
 {
  Outlier("General", 0.05);
  color="mediumblue";
 };
 Date("SG-OSL CLD17-1", N(2017-37747,2808))
 {
  Outlier("General", 0.05);
  color="green";
 };
 R_Date("14C OxA-1941", 27600, 600)
 {
  Outlier("General", 0.05);
  color="mediumblue";
 };
 R_Date("14C VERA-5454", 28000, 210)
 {
  Outlier("General", 0.05);
  color="mediumblue";
 };
 After()
 {
  R_Date("14C OxA-22301", 37500, 230)
  {
   Outlier("General", 0.05);
   color="mediumblue";
  };
 };
};
Boundary("Boundary Layer K top");
Boundary("Boundary Layer Jb bottom");
Phase("Layer Jb")
{
 R_Date("14C OxA-22300", 25750, 110)
 {
  Outlier("General", 0.05);
  color="mediumblue";
 };
 R_Date("14C OxA-5542", 26020, 320)
 {
  Outlier("General", 0.05);
  color="mediumblue";
 };
 R_Date("14C OxA-22299", 25560, 100)
 {
  Outlier("General", 0.05);
  color="mediumblue";
 };
};
Boundary("Boundary Layer Jb top");
Boundary("Boundary Layer I-Ja bottom");
Phase("Layer I-Ja")
{
 R_Date("14C MAMS-38337", 23437, 140)
 {
  Outlier("General", 0.05);
  color="mediumblue";
 };
 R_Date("14C OxA-1940", 22900, 380)
 {
```

```
   Outlier("General", 0.05);
   color="mediumblue";
  };
 };
 Boundary("Boundary Layer I-Ja top");
 Boundary("Boundary Layer H bottom");
 Phase("Layer H")
 {
  R_Date("14C OxA-1939", 19900, 260)
  {
   Outlier("General", 0.05);
   color="mediumblue";
  };
  R_Date("14C OxA-X-2786-13", 19400, 150)
  {
   Outlier("General", 0.05);
   color="mediumblue";
  };
  R_Date("14C OxA-2511", 20530, 270)
  {
   Outlier("General", 0.05);
   color="mediumblue";
  };
 };
 Boundary("Boundary Layer H top");
 Boundary("Boundary Layer Fa-Fc bottom");
 Phase("Layer Fa-Fc")
 {
  R_Date("14C OxA-2510", 18840, 200)
  {
   Outlier("General", 0.05);
   color="mediumblue";
  };
  After()
  {
   R_Date("14C OxA-1938", 20400, 270)
   {
    Outlier("General", 0.05);
    color="mediumblue";
   };
  };
 };
 Boundary("Boundary Layer Fa-Fc top");
};
};
```

Model IV

```
Plot()
 {
  Outlier_Model("General",T(5),U(0,4),"t");
  Sequence("Gruta do Caldeirão Sequence")
  {
   Boundary("Boundary Layer O bottom");
   Date("SG-OSL CLD17-5", N(2017-58376,3777))
   {
    Outlier("General", 0.05);
    color="green";
   };
   Boundary("Boundary Layer O top");
   Boundary("Boundary Layer N bottom");
   Date("SG-OSL CLD17-4", N(2017-41978,2544))
   {
    Outlier("General", 0.05);
    color="green";
   };
   Boundary("Boundary Layer N top");
   Boundary("Boundary Layer M bottom");
   Date("SG-OSL CLD17-3", N(2017-37921,2258))
   {
    Outlier("General", 0.05);
    color="green";
   };
   Boundary("Boundary Layer M top");
   Boundary("Boundary Layer L bottom");
   Date("SG-OSL CLD17-2", N(2017-39618,2268))
   {
    Outlier("General", 0.05);
    color="green";
   };
   Boundary("Boundary Layer L top");
   Boundary("Boundary Layer K bottom");
   Phase("Layer K")
   {
    R_Date("14C OxA-22020", 26790, 260)
    {
     Outlier("General", 0.05);
     color="mediumblue";
    };
    Date("SG-OSL CLD17-1", N(2017-37747,2808))
    {
     Outlier("General", 0.05);
     color="green";
    };
    R_Date("14C OxA-1941", 27600, 600)
    {
     Outlier("General", 0.05);
     color="mediumblue";
    };
    R_Date("14C VERA-5454", 28000, 210)
    {
     Outlier("General", 0.05);
     color="mediumblue";
    };
    After()
```

```
  {
   R_Date("14C OxA-22301", 37500, 230)
   {
    Outlier("General", 0.05);
    color="mediumblue";
   };
  };
 };
 Boundary("Boundary Layer K top");
 Boundary("Boundary Layer Jb bottom");
 Phase("Layer Jb")
 {
  R_Date("14C OxA-22300", 25750, 110)
  {
   Outlier("General", 0.05);
   color="mediumblue";
  };
  R_Date("14C OxA-5542", 26020, 320)
  {
   Outlier("General", 0.05);
   color="mediumblue";
  };
  R_Date("14C OxA-22299", 25560, 100)
  {
   Outlier("General", 0.05);
   color="mediumblue";
  };
 };
 Boundary("Boundary Layer Jb top");
 Boundary("Boundary Layer I-Ja bottom");
 Phase("Layer I-Ja")
 {
  R_Date("14C MAMS-38337", 23437, 140)
  {
   Outlier("General", 0.05);
   color="mediumblue";
  };
  R_Date("14C OxA-1940", 22900, 380)
  {
   Outlier("General", 0.05);
   color="mediumblue";
  };
 };
 Boundary("Boundary Layer I-Ja top");
 Boundary("Boundary Layer H bottom");
 Phase("Layer H")
 {
  R_Date("14C OxA-1939", 19900, 260)
  {
   Outlier("General", 0.05);
   color="mediumblue";
  };
  R_Date("14C OxA-X-2786-13", 19400, 150)
  {
   Outlier("General", 0.05);
   color="mediumblue";
  };
  R_Date("14C OxA-2511", 20530, 270)
  {
```

```
   Outlier("General", 0.05);
   color="mediumblue";
  };
 };
 Boundary("Boundary Layer H top");
 Boundary("Boundary Layer Fa-Fc bottom");
 Phase("Layer Fa-Fc")
 {
  R_Date("14C OxA-2510", 18840, 200)
  {
   Outlier("General", 0.05);
   color="mediumblue";
  };
  After()
  {
   R_Date("14C OxA-1938", 20400, 270)
   {
    Outlier("General", 0.05);
    color="mediumblue";
   };
  };
 };
 Boundary("Boundary Layer Fa-Fc top");
};
};
```

Model V

```
Plot()
 {
  Outlier_Model("General",T(5),U(0,4),"t");
  Sequence("Gruta do Caldeirão Sequence")
  {
   Boundary("Boundary Layer O bottom");
   Date("SG-OSL CLD17-5", N(2017-58376,3777))
   {
    Outlier("General", 0.05);
    color="green";
   };
   Boundary("Boundary Layer O top");
   Boundary("Boundary Layer L-N + Unit 5-6 bottom");
   Phase("Layer L-N + Unit 5-6")
   {
    Date("SG-OSL CLD17-4", N(2017-41978,2544))
    {
     Outlier("General", 0.05);
     color="green";
    };
    Date("SG-OSL CLD17-3", N(2017-37921,2258))
    {
     Outlier("General", 0.05);
     color="green";
    };
    Before()
    {
     R_Date("14C MAMS-33905", 31900, 170)
     {
      Outlier("General", 0.05);
      color="mediumblue";
     };
    };
    Before()
    {
     R_Date("14C MAMS-41872", 28150, 160)
     {
      Outlier("General", 0.05);
      color="mediumblue";
     };
    };
    Date("SG-OSL CLD17-2", N(2017-39618,2268))
    {
     Outlier("General", 0.05);
     color="green";
    };
    R_Date("14C MAMS-41871", 36490, 390)
    {
     Outlier("General", 0.05);
     color="mediumblue";
    };
    R_Date("14C MAMS-41876", 32890, 260)
    {
     Outlier("General", 0.05);
     color="mediumblue";
    };
    R_Date("14C MAMS-41874", 33810, 290)
```

```
 {
  Outlier("General", 0.05);
  color="mediumblue";
 };
};
Boundary("Boundary Layer L-N + Unit 5-6 top");
Boundary("Boundary Layer K bottom");
Phase("Layer K")
{
 R_Date("14C OxA-22020", 26790, 260)
 {
  Outlier("General", 0.05);
  color="mediumblue";
 };
 Date("SG-OSL CLD17-1", N(2017-37747,2808))
 {
  Outlier("General", 0.05);
  color="green";
 };
 R_Date("14C OxA-1941", 27600, 600)
 {
  Outlier("General", 0.05);
  color="mediumblue";
 };
 R_Date("14C VERA-5454", 28000, 210)
 {
  Outlier("General", 0.05);
  color="mediumblue";
 };
 After()
 {
  R_Date("14C OxA-22301", 37500, 230)
  {
   Outlier("General", 0.05);
   color="mediumblue";
  };
 };
};
Boundary("Boundary Layer K top");
Boundary("Boundary Layer Jb bottom");
Phase("Layer Jb")
{
 R_Date("14C OxA-22300", 25750, 110)
 {
  Outlier("General", 0.05);
  color="mediumblue";
 };
 R_Date("14C OxA-5542", 26020, 320)
 {
  Outlier("General", 0.05);
  color="mediumblue";
 };
 R_Date("14C OxA-22299", 25560, 100)
 {
  Outlier("General", 0.05);
  color="mediumblue";
 };
};
Boundary("Boundary Layer Jb top");
```

```
  Boundary("Boundary Layer I-Ja bottom");
  Phase("Layer I-Ja")
  {
   R_Date("14C MAMS-38337", 23437, 140)
   {
    Outlier("General", 0.05);
    color="mediumblue";
   };
   R_Date("14C OxA-1940", 22900, 380)
   {
    Outlier("General", 0.05);
    color="mediumblue";
   };
  };
  Boundary("Boundary Layer I-Ja top");
  Boundary("Boundary Layer H bottom");
  Phase("Layer H")
  {
   R_Date("14C OxA-1939", 19900, 260)
   {
    Outlier("General", 0.05);
    color="mediumblue";
   };
   R_Date("14C OxA-X-2786-13", 19400, 150)
   {
    Outlier("General", 0.05);
    color="mediumblue";
   };
   R_Date("14C OxA-2511", 20530, 270)
   {
    Outlier("General", 0.05);
    color="mediumblue";
   };
  };
  Boundary("Boundary Layer H top");
  Boundary("Boundary Layer Fa-Fc bottom");
  Phase("Layer Fa-Fc")
  {
   R_Date("14C OxA-2510", 18840, 200)
   {
    Outlier("General", 0.05);
    color="mediumblue";
   };
   After()
   {
    R_Date("14C OxA-1938", 20400, 270)
    {
     Outlier("General", 0.05);
     color="mediumblue";
    };
   };
  };
  Boundary("Boundary Layer Fa-Fc top");
 };
};
```
